# Effects of different exercise prescription parameters on metabolic and inflammatory biomarkers in cancer patients: a systematic review, meta-analysis, and meta-regression

**DOI:** 10.3389/fimmu.2025.1663560

**Published:** 2025-08-14

**Authors:** Jingyu Wang, Yuxuan He, Ziqian Wang, Zhouluo Wang, Yongqi Miao, Jae-Young Choi

**Affiliations:** ^1^ Department of Sport Leisure, Sungshin Women’s University, Seoul, Republic of Korea; ^2^ School of Education and Arts, Jiujiang Polytechnic University of Science and Technology, Jiujiang, China; ^3^ School of Philosophy and Sociology, Jilin University, Changchun, China; ^4^ College of Physical Education, Jilin University, Changchun, China; ^5^ Department of Physical Education, College of Education, Korea University, Seoul, Republic of Korea

**Keywords:** cancer, metabolism, inflammation, exercise, exercise prescription

## Abstract

**Objective:**

This study aimed to systematically evaluate the effects of exercise interventions on metabolic and inflammatory biomarkers in cancer patients, and to identify potential dose–response relationships and modulatory mechanisms using Robust Variance Estimation (RVE) and MetaForest models.

**Methods:**

A systematic search of five databases was conducted from inception to March 6, 2025, based on the PICOS framework. Randomized controlled trials involving exercise interventions of ≥4 weeks in adults (≥18 years) with cancer were included. Effect sizes were pooled using RVE to estimate overall intervention effects. Risk of bias was assessed using the ROB2 tool, and the certainty of evidence was evaluated with the GRADE approach. Univariable RVE meta-regression was performed to examine the linear effects of each moderator. MetaForest was used to assess variable importance and to explore potential nonlinear relationships between moderators and intervention effects. Subgroup analyses were conducted by cancer type and intervention timing.

**Results:**

A total of 83 eligible articles were included, representing 74 distinct randomized controlled trials, from which data were extracted. Exercise significantly reduced insulin levels (ES = –0.24, SE = 0.08, p < 0.01, I² = 49%), representing a small but meaningful effect. TNF-α showed a small effect (ES = –0.22, SE = 0.13) but was not statistically significant (p = 0.10, I² = 74%). MetaForest modeling revealed that the most favorable changes in IL-6, adiponectin, and IGF-1 were associated with high-intensity aerobic exercise; TNF-α, IL-8, and IL-10 responded best to longer weekly exercise duration; and improvements in glucose, leptin, and CRP were most pronounced when exercise was combined with caloric restriction.

**Conclusion:**

Regular exercise confers modest but favorable effects on metabolic and inflammatory biomarkers in cancer patients. Meta-regression highlighted the importance of high-intensity aerobic exercise (HRR > 85%) in modulating IL-6, adiponectin, and IGF-1, as well as longer weekly exercise duration (>280 min/week) in improving TNF-α and IL-8. Mechanistically, high-intensity aerobic exercise may serve as a primary trigger for activating pathways that mediate metabolic and inflammatory improvements.

**Systematic review registration:**

https://www.crd.york.ac.uk/PROSPERO/view/CRD420251002676.

## 1 Introduction

For a long time, cancer has been regarded as a genetic disease (1). However, an increasing number of studies now suggest that cancer should also be considered a metabolic disease (1, 2). This shift in perspective is partly attributed to a deeper understanding of the mechanisms underlying changes in the tumor microenvironment (TME) (2). Persistent hypoxia, lactate accumulation, and energy competition within the TME can induce metabolic reprogramming in patients to meet the energy demands of tumor proliferation (3, 4). Metabolic reprogramming is characterized by alterations in host glucose and lipid metabolism that favor tumor growth and invasion (5). These tumor-centric metabolic changes may progressively extend throughout the body, leading to systemic metabolic disturbances (6). As a result, cancer patients often present with typical features of metabolic syndrome, such as insulin resistance and dyslipidemia (7–9). To date, numerous studies have shown that metabolic syndrome is associated with increased cancer risk and poor prognosis in various malignancies (10–12).

More complex still is the close interplay between metabolic dysregulation and inflammation (13, 14). Hypoxia and lactate accumulation within the TME impair the function of T cells and natural killer cells, thereby weakening antitumor immune responses (15). Metabolic disturbances can induce sustained secretion of pro-inflammatory cytokines in the TME, such as interleukin 6 (IL-6), tumor necrosis factor alpha (TNF-α), and IL-1β, which activate systemic inflammatory pathways and exacerbate metabolic dysfunction (13, 16). Importantly, accumulating evidence suggests that combining inflammatory markers with metabolic indicators offers a reliable approach for prognostic prediction in cancer populations (17–20). Thus, abnormalities in glucose metabolism, lipid metabolism, and inflammatory signaling have become a key framework for understanding systemic cancer progression and underscore the need for multi-targeted interventions (4, 5, 21).

Beyond pharmacological therapies, exercise has emerged as a non-pharmacological strategy with multi-target effects (22). Mechanistic studies have partially confirmed that regular physical activity may exert anticancer effects by modulating metabolic signaling pathways and reshaping the TME (23, 24). Recent animal studies also suggest a potential conflict between tumor metabolic plasticity and exercise-induced metabolic reprogramming in stromal cells (25). At the clinical level, previous meta-analyses have provided preliminary evidence that exercise can improve common metabolic disturbances in cancer patients, including blood glucose, insulin, and triglycerides, as well as inflammatory markers such as IL-6, C-reactive protein (CRP), and TNF-α (26, 27). However, existing studies have primarily focused on breast cancer survivors, with a lack of cross-cancer analyses and comprehensive integration of systemic metabolic and inflammatory indicators.

Moreover, maximizing the benefits of exercise interventions depends heavily on the precise tailoring of exercise prescription parameters, such as frequency, intensity, and duration (28, 29). Although previous studies using subgroup analyses have indicated that intervention duration (e.g., >12 weeks vs. <12 weeks) may moderate changes in IL-6 and CRP levels among breast cancer survivors, Bayesian network meta-analyses have identified high-intensity aerobic and resistance exercise as the most promising modalities for reducing inflammation, with total exercise volume influencing TNF-α concentrations (30, 31). Nonetheless, these approaches have inherent limitations. Subgroup analysis often relies on dichotomizing continuous variables, and Bayesian network meta-analysis is generally restricted to comparing intervention types. Therefore, in the context of limited head-to-head trials, there is a pressing need for more advanced statistical modeling to explore how different exercise prescription parameters regulate metabolic and inflammatory responses in cancer patients, thereby informing precision exercise strategies (24, 32).

To address these methodological limitations, recent meta-analyses have begun to adopt more flexible and robust modeling techniques. Robust Variance Estimation (RVE) offers a flexible random-effects meta-regression framework that accommodates complex data structures (33). Even when the underlying correlation structure is unknown, RVE can model correlated effect sizes within the same study (34, 35). MetaForest, a machine learning approach that integrates random forest algorithms into meta-analytic models, enables the detection of nonlinear relationships and complex interactions among moderator variables (36). While MetaForest has been used to explore moderators of intervention effects, it has not yet been applied to optimize exercise prescription parameters (37, 38).

Therefore, this study aimed to systematically evaluate the effects of exercise interventions on metabolic dysregulation and inflammatory biomarkers in cancer patients, using both RVE and MetaForest models to identify potential dose–response patterns and regulatory mechanisms. We conducted a meta-analysis and regression based on randomized controlled trials reporting exercise interventions of at least 4 weeks that targeted metabolic and inflammatory biomarkers in cancer populations. The specific objectives were (1): to summarize effect sizes using RVE to determine overall intervention effects (2); to perform univariable meta-regression with RVE to assess linear influences of moderator variables (3); to apply the MetaForest model to evaluate the relative importance of moderators and interpret their nonlinear associations with intervention effects; and (4) to conduct subgroup analyses by cancer type and intervention timing.

## 2 Methods

This systematic review was registered with PROSPERO (registration number: CRD420251002676) and conducted in accordance with the PRISMA guidelines (**Supplementary Information 1**) (39).

### 2.1 Data sources, search strategy, and eligibility criteria

A systematic literature search was conducted from database inception to March 6, 2025, across five databases: PubMed (MEDLINE), Embase, the Cochrane Central Register of Controlled Trials (CENTRAL), SPORTDiscus, and Web of Science. In addition, reference lists of included studies and relevant systematic reviews or meta-analyses were manually screened to identify additional eligible studies. The search strategy was developed based on the PICOS framework (**Supplementary Information 2**).

The inclusion criteria were as follows: middle-aged and elderly cancer patients; intervention: exercise interventions lasting at least 4 weeks, with clearly reported exercise parameters aimed at increasing physical activity; comparator: non-exercise control groups, including usual care, health education, or waitlist controls; outcomes: at least one obesity-related or inflammatory biomarker; study design: randomized controlled trials (RCTs).

The exclusion criteria were as follows: population: cancer patients under the age of 18; intervention: rehabilitation programs, mind–body exercises (e.g., yoga, tai chi), or exergaming; comparator: absence of a non-exercise control group; outcomes: surgery-related outcomes (e.g., perioperative indicators, tumor resection quality, postoperative recovery, or prognosis); study design: systematic reviews, narrative reviews, animal studies, or non-English publications. Studies were also excluded if the full text or relevant data could not be obtained after contacting the corresponding authors.

In this review, we distinguished between “physical activity (PA)” (any bodily movement produced by skeletal muscles that results in energy expenditure) and “exercise” (a subset of physical activity that is planned, structured, and repetitive, with the objective of improving or maintaining physical fitness) (40). Interventions were included only if they provided clearly defined parameters and aligned with structured exercise or prescribed physical activity protocols (e.g., walking >10,000 steps per day or 30 minutes of moderate-intensity activity), even if labeled as “PA” in the original study.

This study focused on the effects of long-term exercise interventions, rather than the acute effects of single or infrequent exercise sessions. “Regular exercise” was defined as structured programs delivered at a consistent frequency for at least 4 weeks, following established clinical exercise prescription guidelines for patient populations (41, 42). In addition, given that recent network meta-analyses have reported minimal effects of mind–body exercises on inflammatory biomarkers (31), and that such interventions lack quantifiable exercise prescription parameters (e.g., intensity), randomized controlled trials using mind–body exercises as the sole intervention were not included in this review.

### 2.2 Study selection and data extraction

Two authors (YXH and ZQW) independently screened the eligible studies to identify trials that met the inclusion criteria. Discrepancies were resolved by a third, experienced author (JYW). The following study characteristics were extracted: basic study information (e.g., author, registration number), outcome measures (excluding follow-up data), and moderator variables. For studies reporting data in formats other than mean ± SD (e.g., median, interquartile range, standard error), values were converted according to the Cochrane Handbook for Systematic Reviews of Interventions (43).

Outcomes were categorized into three domains: Glucose–Insulin Group, Lipid Group, and Inflammatory Group (**Supplementary Information 4**). A total of 25 potential moderator variables related to exercise interventions were prespecified. Moderators with more than 30% missing data were excluded, while those with 0% to 30% missingness were imputed using multiple imputation. Moderator variables were classified into two groups based on their functional role: (1) exercise prescription moderators (e.g., type, duration, frequency, session intensity, total volume), which were the primary focus of the analysis; and (2) background moderators (e.g., age, male ratio, BMI), which were used to account for demographic and design-related heterogeneity.

### 2.3 Risk of bias and quality of evidence assessment

The risk of bias in included RCTs was assessed using the Cochrane Risk of Bias tool (RoB2) (44). Two authors (YXH and ZQW) independently conducted the assessments, with disagreements resolved by a third reviewer (JYW). Inter-rater agreement was acceptable (Kappa = 0.87). Given the nature of exercise interventions, “blinding of participants and personnel” was considered to be at low risk. The GRADE (Grading of Recommendations, Assessment, Development, and Evaluation) framework was applied to evaluate the quality of evidence (45).

### 2.4 Data analysis

All statistical analyses were conducted using R (version 4.4.1). Cohen’s d and its variance were used to summarize effect sizes (46). A d of 0.2 indicated a small effect, 0.5 a medium effect, and 0.8 a large effect. All effect sizes were transformed so that negative values indicated beneficial outcomes. For studies without explicit descriptions of moderator variables, mean imputation was conducted based on comparable studies. Meta-regression models were fitted using the “metafor” package, and variance inflation factors (VIF) were calculated to assess multicollinearity. Moderator variables with VIF > 10 were excluded. Data visualization was performed using the “ggplot2” package.

The RVE method was implemented using the “robumeta” package. Regression models assumed a correlated effects structure with an intrastudy correlation of 0.5 (model weights = “CORR”, rho = 0.5). Sensitivity analyses were conducted by varying rho from 0.1 to 0.9 in increments of 0.05 (e.g., rho = 0.1, 0.15, 0.2). An RVE meta-regression model without covariates was used to estimate the overall mean effect size. Between-study heterogeneity was assessed using the I² statistic, with I² > 25% indicating low, > 50% moderate, and > 75% high heterogeneity. Egger’s test was performed using the “metafor” package to detect publication bias, and the trim-and-fill method was used to adjust for potential bias and generate corrected funnel plots. Subsequently, univariable RVE meta-regression models were fitted, and I² and R² values were recorded for each model. In RVE models, R² represents the proportion of heterogeneity explained by moderators. The model with R² closest to 1 was considered optimal (47).

The MetaForest model was constructed following the method proposed by Van Lissa (36). Random-effects weights (“whichweights” = “random”) were applied, and 20,000 decision trees were grown. Convergence plots were generated. Cross-validation and parameter tuning were performed using the “caret” package, with 100 bootstrap resampling iterations. Bootstrapping ensured model robustness, particularly in small-sample contexts. Tuned parameters included weight type (random, fixed, uniform), number of variables per split (mtry = 2, 4, 6), and minimum node size (min.node.size = 2, 4). Partial dependence plots and variable importance plots were generated. In MetaForest, R² reflects model prediction accuracy and generalizability. Subgroup MetaForest analyses were conducted based on intervention timing and cancer type, with model parameters held constant.

## 3 Results

**Figure 1:**
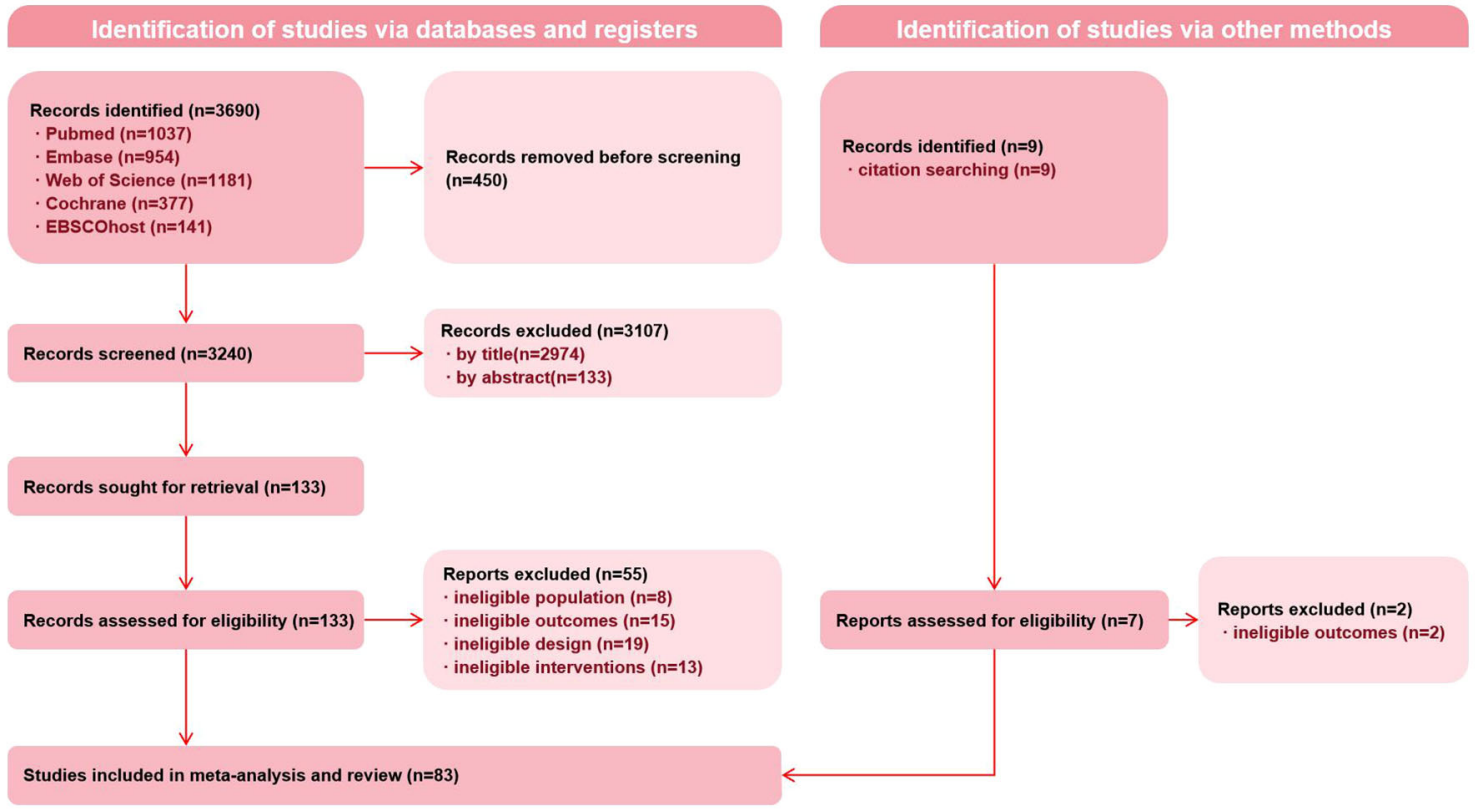
Preferred Reporting Items for Systematic Reviews and Meta-Analyses (PRISMA) flow diagram of record identification, screening, and selection processes.

A total of 3,690 records were initially identified. A total of 83 full-text articles corresponding to 74 unique RCTs were retrieved, as some trials were reported in more than one publication. Detailed information on the included RCTs and reported outcomes is provided in **Supplementary Information 3**. Some biomarkers (e.g., IL-1β, IL-12) were excluded from analysis due to an insufficient number of RCTs (<10) or effect sizes (<15). For RCTs reporting multiple time points or intervention arms, effect sizes were extracted separately. The full screening and selection process is illustrated in **Figure 1**.

### 3.1 Overall effects

A total of 74 RCTs involving 4,654 cancer patients were included. Detailed information of the 74 RCTs is recorded in **Supplementary Information 3**. Exercise-related adverse events mainly included pain (e.g., muscle soreness, joint pain, shin pain), flu-like symptoms, foot blisters, and injuries (e.g., joint or meniscus injuries) (48–55). No serious exercise-related adverse events were reported in any of the studies.

**Figure 2:**
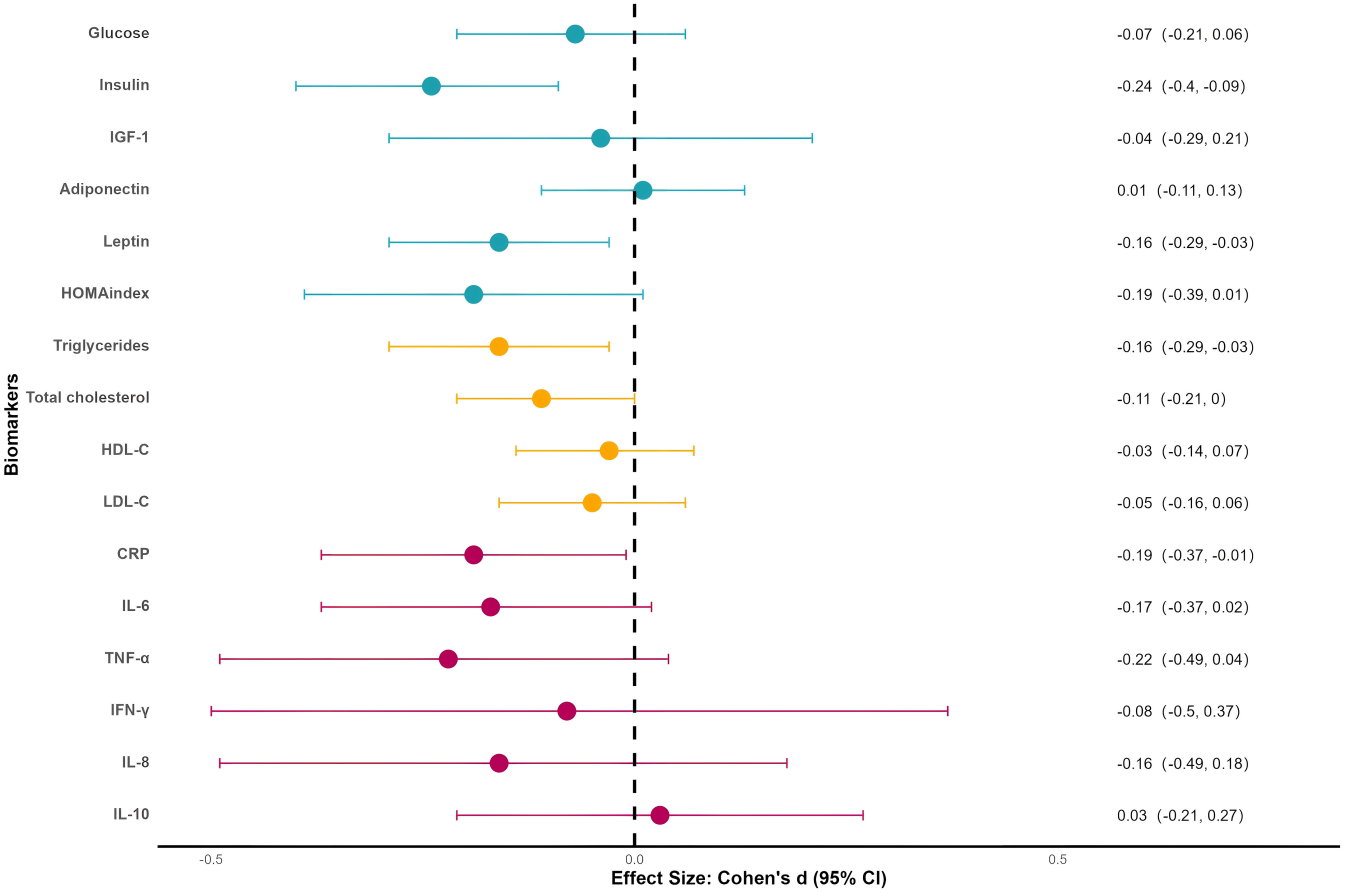
Forest plot for meta-analyses. IGF-1, insulin-like growth factor 1; HDL-C, high-density lipoprotein cholesterol; LDL-C, low-density lipoprotein cholesterol; CRP, C-reactive protein; IL-6, interleukin 6; TNF-α, tumor necrosis factor alpha; IFN-γ, interferon gamma; IL-8, interleukin 8; IL-10, interleukin 10.

**Figure 2** displays the effect sizes of 481 outcomes synthesized using the RVE model. Insulin showed a small but statistically significant effect (ES = –0.24, SE = 0.08, p < 0.01, I² = 49%). TNF-α reached a small effect size (ES = –0.22, SE = 0.13, p = 0.10, I² = 74%) but did not reach statistical significance. Leptin, HOMA index, triglycerides, CRP, IL-6, and IL-8 approached the threshold of small effects (ES > 0.15). Effect sizes for all other biomarkers are reported in **Supplementary Information 5**, **Supplementary Table S3**. Sensitivity analyses indicated that variations in the assumed rho value did not affect the stability of the model (**Supplementary Information 5**, **Supplementary Table S3**). Forest plots displaying the original effect sizes for each biomarker are presented in **Supplementary Information 5**, **Supplementary Figures S1–S16**.

### 3.2 Risk of bias and publication bias

Risk of bias was assessed using the ROB2 tool. The main sources of potential bias included lack of trial preregistration, absence of missing data handling descriptions, and insufficient details regarding exercise intervention protocols (**Supplementary Information 6**). **Supplementary Figure S17** presents the summary of risk of bias assessments, and **Supplementary Figure S18** shows the individual risk of bias ratings for each included study. Egger’s tests for 16 outcome indicators yielded p values greater than 0.05, indicating no significant publication bias (**Supplementary Information 6**, **Supplementary Figure S19**).

### 3.3 Moderator analysis results

A total of 19 moderator variables met the criteria for multiple imputation and multicollinearity diagnostics and were included in the final analysis. The extraction methods and descriptive statistics of all moderators are presented in **Supplementary Information 7**. Several univariable RVE models identified linear trends in moderator effects and explained heterogeneity in overall outcomes (**Supplementary Information 8**). For example, weekly exercise volume showed a significant linear association with IL-10 (p = 0.03), explaining 64% of the variance (R² = 0.64).

**Figure 3:**
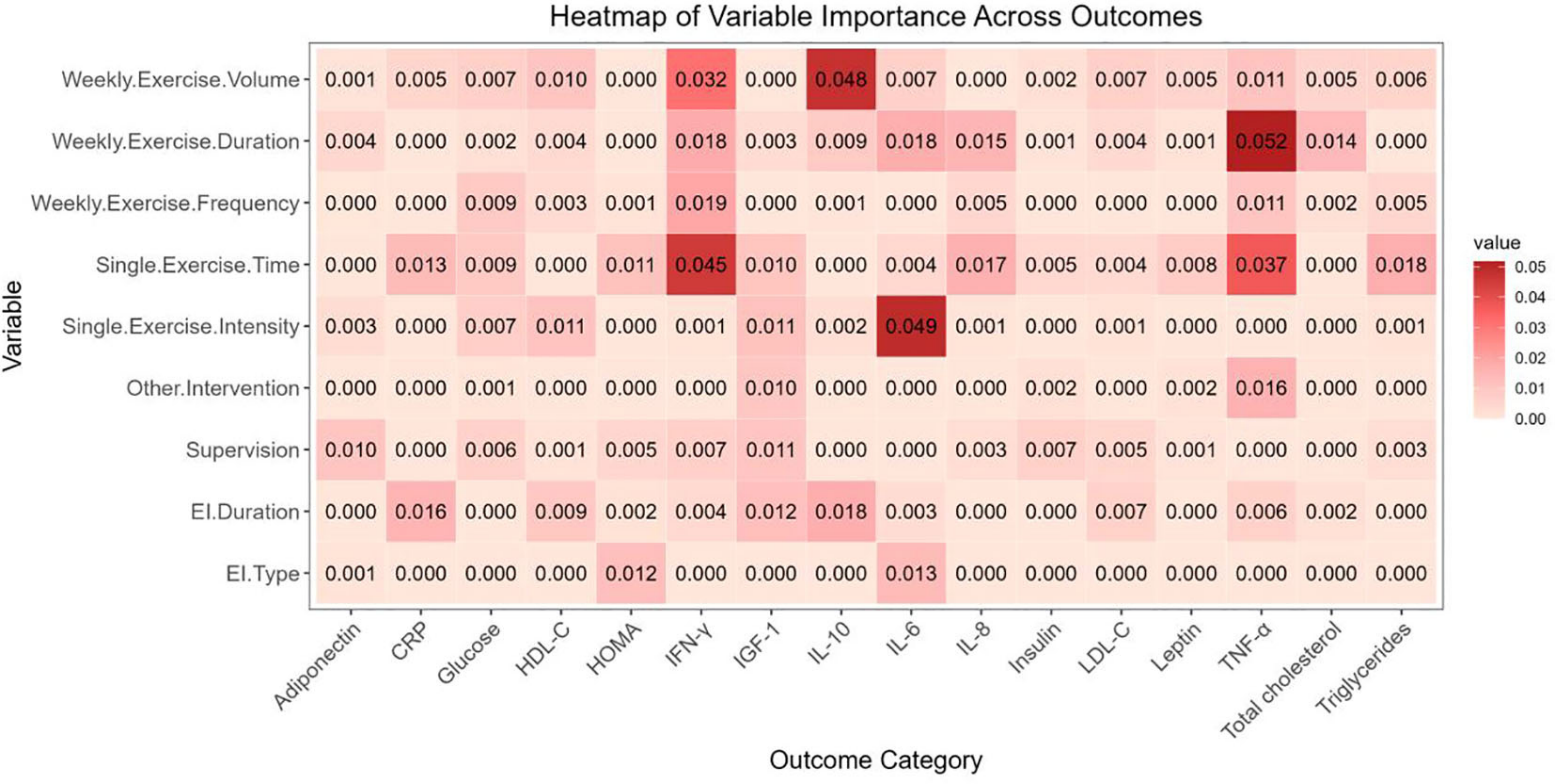
Variable importance heatmap generated by the MetaForest model. EI Duration, exercise intervention duration; EI Type, exercise intervention type.

**Supplementary Information 9** provides MetaForest model parameters (**Supplementary Tables S7, S8**) and convergence diagnostics (**Supplementary Figures S20, S37**), all of which indicated good model convergence. **Figure 3** presents the variable importance of exercise prescription moderators for 16 outcome indicators as identified by the MetaForest models.

**Table 1** summarizes the optimal effect ranges of each exercise prescription parameter derived from both RVE and MetaForest models (**Supplementary Figures S21–S36**). For categorical variables, RVE models identified significantly greater effects of aerobic exercise (AE) compared to resistance training (RT) for Adiponectin, HOMA, Total Cholesterol, and low-density lipoprotein cholesterol (LDL-C) (p < 0.05). For IL-6, AE also outperformed physical activity (PA) with statistical significance (p < 0.05). MetaForest models similarly identified AE as the most effective exercise type for IL-6, Adiponectin, and HOMA. Total cholesterol and LDL-C showed more favorable outcomes in studies with longer intervention durations (>20 weeks). CRP, glucose, leptin, and high-density lipoprotein cholesterol (HDL-C) demonstrated better improvements when combined with caloric restriction. No consistent pattern was found across outcomes regarding the mode of exercise supervision.

**Table 1:** Overview of results from RVE and MetaForest models.

Metabolic and inflammatory biomarkers	Type (AE/RT/PA/AE+RT)	Duration (week)	Intensity (MET)	Single time (min)	Frequency (times/week)	Weekly duration (min)	Volume (MET-min/week)	Other Intervention (Calorie restriction/No)	Supervision (Yes/Half/No)
(a) Glucose–Insulin Group									
Glucose	–	–	–	110-125	≤3	–	<2500	Calorie restriction	No
Insulin	–	–	–	110-125	–	150-250	1250-2000	No	Half
IGF-1	–	<12	≥8	<50	–	–	–	No*	Half
Adiponectin	AE #(RT ↓)	–	≥8#	20-80	–	100-175	500-1500	–	No*
Leptin	–	–	–	40-80	–	225-350	1000-2000	Calorie restriction	Yes
HOMA index	AE* #(RT ↓)	<12	–	>75	–	–	–	No	No
(b) Lipid Group									
Triglycerides	–	–	–	110-125	≤3	–	800-1400	–	No
Total cholesterol	#(RT ↓)	>25*	–	–	2-4	<200*	>1000	–	–
HDL-C	–	<20*	–	–	≤3	110-280	800-1500	Calorie restriction	No
LDL-C	#(RT ↓)	>20#	5-8	90-100	–	>300	>2000	–	Yes
(c) Inflammatory Group									
CRP	–	<20	–	<80	–	–	<2000#	Calorie restriction	–
IL-6	AE #(PA↓)	–	≥9	–	–	<150	>1500	–	–
TNF-α	–	<20	–	>75	–	>280	>2000	No	–
IFN-γ	–	<10	–	<55	≤5	<200	<2000	–	Yes
IL-8	–	–	–	>75	–	>280	>2000	–	Half
IL-10	–	<10	–	–	–	>280#	>2000*#	–	–

*indicates variable importance identified by the MetaForest model (p < 0.05). #indicates a significant association identified by the univariable RVE meta-regression (p < 0.05). – indicates negative variable importance or no clear nonlinear trend was observed. #(PA↓)/#(RT↓) means that the RVE regression model identified that PA or RT was significantly less effective than AE. IGF-1, insulin-like growth factor 1; HDL-C, high-density lipoprotein cholesterol; LDL-C, low-density lipoprotein cholesterol; CRP, C-reactive protein; IL-6, interleukin 6; TNF-α, tumor necrosis factor alpha; IFN-γ, interferon gamma; IL-8, interleukin 8; IL-10, interleukin 10; AE, aerobic exercise; RT, resistance training; PA, physical activity; AE+RT, combined aerobic and resistance training.

For continuous variables, the current guideline-recommended exercise dose, as calculated in this study, corresponds to approximately 1,500 MET-min/week (at least 150 minutes per week of moderate-intensity AE and 2–3 sessions of RT). The optimal ranges for IL-6, TNF-α, IL-10, LDL-C, and IL-8 all exceeded 1,500 MET-min/week. Specifically, TNF-α, IL-10, and IL-8 showed stronger effects at longer weekly exercise durations (>280 minutes/week), while IL-6 (≥9 METs) and LDL-C (5–9 METs) demonstrated optimal effects at specific intensity levels. Although the optimal total dose did not exceed 1,500 MET-min/week, high-intensity exercise (≥8 METs) was associated with greater effects on Adiponectin and insulin-like growth factor 1 (IGF-1). Additionally, while IL-8, insulin, and triglycerides showed some moderator effects under specific prescription parameters, they did not provide stronger explanatory value regarding exercise intensity, weekly duration, or total dose.

Additional results regarding background moderators identified by the MetaForest model are shown in **Supplementary Figure S38**, with partial dependence plots provided in **Supplementary Figures S39–S54**. Overall, patient baseline status, BMI, and age exhibited moderating effects on several biomarkers, including glucose, insulin, and CRP. Other background variables, such as cancer stage and cancer type, also showed some influence on specific outcomes.

### 3.4 Subgroup analysis

Subgroup analyses were conducted based on cancer type (breast cancer, colorectal cancer, and prostate cancer) and intervention timing (before treatment, during treatment, or survivorship) (**Supplementary Information 10, 11**). Due to insufficient effect sizes, MetaForest analyses for individual outcomes were only performed for the BC and survivor subgroups. The MetaForest models did not identify nonlinear trends that substantially contradicted the findings presented in **Table 1**.

Additional subgroup analyses were performed for BC during-treatment and survivorship subgroups (**Supplementary Information 12**). For the Glucose–Insulin and Inflammatory groups, post-treatment interventions yielded moderate effects and were more effective than during-treatment interventions. In contrast, for the Lipid group, during-treatment interventions showed stronger effects (ES = –0.43) compared to post-treatment interventions (ES = –0.10). According to the MetaForest models, the optimal exercise intensity during treatment was ≤6.5 METs for both the Glucose–Insulin and Lipid groups. For the Glucose–Insulin group, the optimal intensity shifted to >6.5 METs in the post-treatment phase, and interventions longer than 20 weeks yielded greater effects. For the Inflammatory group, optimal exercise intensity was ≥9 METs in both during- and post-treatment periods.

### 3.5 Quality of evidence assessment

The quality of evidence was evaluated using the GRADE framework. Summary results are presented in **Supplementary Information 13**. The overall certainty of evidence based on effect size synthesis ranged from moderate to very low (**Supplementary Table S22**). The certainty of evidence derived from MetaForest models ranged from low to very low (**Supplementary Table S23**).

## 4 Discussion

The aim of this systematic review and meta-analysis was to determine the effects of different exercise prescription parameters on metabolic and inflammatory biomarkers in cancer patients. The results showed that regular exercise interventions led to a small but significant improvement in insulin levels among cancer patients. Improvements in leptin, HOMA index, triglycerides, and CRP were also close to small in magnitude and statistically significant. IL-6, TNF-α, and IL-8 showed improvements that did not reach statistical significance, and the effects on other biomarkers were smaller. MetaForest modeling revealed that the most favorable changes in IL-6, adiponectin, and IGF-1 were associated with high-intensity aerobic exercise; TNF-α, IL-8, and IL-10 responded best to longer weekly exercise duration; and improvements in glucose, leptin, and CRP were most pronounced when exercise was combined with caloric restriction.

### 4.1 Effects and mechanisms of exercise on metabolic and inflammatory biomarkers in cancer patients

Our findings indicate that regular exercise significantly improved insulin levels and HOMA index in cancer patients, with a non-significant reduction in glucose. These results are consistent with previous studies that did not distinguish between cancer types and were limited to cancer survivors (26, 56). Among biomarkers related to glucose metabolism, leptin showed the most substantial and statistically significant improvement, aligning with findings from studies focused on breast cancer survivors (57–59). Triglyceride and total cholesterol levels were also significantly reduced, consistent with the majority of prior research (59, 60). CRP, a marker of systemic inflammation, showed a significant decrease. Concentrations of pro-inflammatory cytokines IL-6, IL-8, and TNF-α were also reduced, though not significantly. These patterns are in line with recent studies exclusively targeting breast cancer survivors (59, 61). Changes in anti-inflammatory cytokines such as IL-10 and IFN-γ were limited, consistent with two prior studies focused on breast cancer patients (58, 62). Overall, regular exercise appears to improve several metabolic and inflammatory biomarkers. These improvements are clinically meaningful, as elevated levels of such markers have been associated with poor prognosis, as outlined in the background section. However, the changes in IGF-1 and adiponectin were relatively small, which contradicts several previous findings that reported favorable responses in these biomarkers (26, 58, 63). Additionally, while prior research has shown that exercise can reduce LDL-C and increase HDL-C levels, our study did not observe significant changes in either (64, 65). These discrepancies may be attributed to the broader inclusion of cancer types and intervention timings in our sample. Therefore, the complex nature of these outcome variations warrants further exploration from the perspective of exercise-induced mechanisms in metabolic and inflammatory regulation.

In recent years, the regulation of AMP-activated protein kinase (AMPK) by exercise has been recognized as a key mechanism through which exercise improves metabolic health (66, 67). AMPK acts as a cellular energy sensor and plays a central role in maintaining energy homeostasis (68). Early animal studies demonstrated that both acute exercise and insulin promote the translocation of glucose transporter 4 (GLUT-4) to facilitate glucose uptake, operating through distinct yet additive mechanisms (69–71). Additionally, acute exercise-induced AMPK activation has been implicated in lipolysis regulation. Specifically, it suppresses lipogenic transcription factors while activating anti-lipogenic signaling (72, 73). In non-cancer animal models, regular exercise improves hepatic and visceral fat steatosis and corrects lipid metabolism disorders by activating AMPK-related pathways (74–76). It also enhances insulin sensitivity and ameliorates hyperglycemia, hyperlipidemia, and aging-related markers (77, 78). In tumor-bearing models, regular exercise has been shown to restore metabolic homeostasis and suppress tumor growth through AMPK pathway modulation (79, 80). These mechanisms have been partially validated in human studies. A 12-week exercise intervention in both heart failure patients and animal models demonstrated that exercise enhances aerobic glucose metabolism by promoting skeletal muscle secretion of meteorin-like protein, which in turn activates the AMPK–HDAC4 pathway in cardiac tissue (81). Furthermore, a trial involving cancer patients undergoing chemotherapy showed that 10 weeks of regular exercise may alleviate cancer- and treatment-induced metabolic stress in skeletal muscle by modulating the GLUT4 and FOXO3a pathways, potentially involving downstream signaling of AMPK (82).

In addition to AMPK, our previous research has explored the role of acute inflammatory responses and long-term adaptation as key mechanisms underlying the anti-inflammatory effects of regular exercise (31). Briefly, skeletal muscle contraction during acute exercise leads to a sharp rise in circulating IL-6, which in turn stimulates the release of anti-inflammatory cytokines such as IL-10 (31). Moreover, studies have shown that the acute elevation of IL-6 during exercise is a critical upstream regulator of AMPK activation and significantly contributes to its maximal stimulation (83). AMPK, in turn, positively regulates IL-10 and inhibits inflammatory signaling pathways activated by IL-1β, TNF-α, and IL-6 (84–86). These effects suggest that AMPK may exert anti-inflammatory actions through multiple regulatory routes. Importantly, the beneficial effects mediated by AMPK-related pathways are not isolated. For example, swimming exercise has been shown to activate the SIRT1/AMPK axis, which mediates both lipid metabolism and inflammatory modulation (87). In tumor-bearing models, AMPK activation has been linked to enhanced T cell survival and antitumor function (88). Regular exercise may also suppress cancer-associated cachexia driven by IL-6 signaling (89, 90). Overall, AMPK may serve as a central molecular link between metabolic homeostasis and inflammation control in exercise-induced systemic adaptations. However, these mechanistic inferences are primarily based on animal studies, and there is a lack of RCT evidence confirming the AMPK-mediated benefits of exercise in cancer patients.

Changes in adipose tissue composition induced by regular exercise may also represent a key mechanism. Excess white adipose tissue contributes to elevated levels of circulating free fatty acids and adipokines such as leptin, IL-6, and TNF-α, promoting a chronic inflammatory state that supports tumor progression (91, 92). This adipose tissue inflammation facilitates a phenotypic shift in macrophages from the anti-inflammatory M2 type to the pro-inflammatory M1 type (93). M1 macrophages secrete cytokines such as IL-6 and TNF-α, leading to glucose and lipid metabolism disorders and insulin resistance can mitigate inflammation not only by reducing adipose tissue volume but also by promoting the browning of white adipose tissue, which possesses anti-inflammatory properties (94, 95). This browning process inhibits M1 macrophage infiltration and alleviates pro-inflammatory conditions (95). Moreover, activated brown adipose tissue has been shown to improve insulin sensitivity, lipid profiles, and glucose homeostasis (96). Although CRP is widely used as a marker of systemic inflammation, numerous studies have shown a positive correlation between adiposity and CRP levels (97, 98). In cancer patients, RCTs have reported that reductions in leptin are highly sensitive to weight loss (99–101). A 16% reduction in fat mass has been associated with improvements in CRP and other biomarkers (102). Changes in obesity-related measures have also been correlated with changes in IL-6 and CRP (103, 104). Collectively, these adipose tissue–mediated effects may partially explain the improvements in metabolic and inflammatory biomarkers observed in our study.

Although the changes in IGF-1 and adiponectin were limited in this study, previous research suggests that both biomarkers may play important roles in regulating metabolic and inflammatory processes. IGF-1 is involved in maintaining insulin sensitivity, increasing glucose uptake, reducing plasma triglycerides, and modulating cholesterol levels (105). Recent animal studies have shown that exercise-induced alterations in IGF-1 signaling can improve skeletal muscle quality and metabolic function (106, 107). Some studies also suggest that IGF-1 and AMPK-related pathways may act in tandem or interact to exert synergistic effects (108, 109). Moreover, in murine models of skin cancer, regular exercise has been shown to suppress IGF-1 signaling, contributing to potential anticancer protection (110). Similarly, adiponectin is involved in lipid metabolism, energy regulation, immune responses, inflammation, and insulin sensitivity (111). Its metabolic actions are closely linked to cancer suppression (112, 113). Animal studies have demonstrated that adiponectin regulates the release of cytokines such as IL-6 and IL-10 during post-exercise metabolic and inflammatory responses through AMPK signaling (114). In addition to directly activating AMPK, adiponectin also exerts its metabolic benefits via calcium-dependent signaling cascades involving AdipoR1 (115). Activation of adiponectin signaling may be part of the molecular mechanism through which exercise alleviates muscle atrophy associated with cancer cachexia (116). Although numerous studies support the beneficial effects of exercise on metabolism and inflammation through IGF-1 and adiponectin pathways, there remains no strong consensus on whether acute or chronic exercise consistently alters their circulating concentrations (117, 118). Therefore, we speculate that the metabolic regulatory effects of exercise involving IGF-1 and adiponectin may be mediated through AMPK or downstream effectors, rather than through consistent changes in their serum levels.

### 4.2 Modulatory effects of exercise prescription parameters on metabolic and inflammatory biomarkers in cancer patients

We observed shared patterns in how different metabolic and inflammatory biomarkers responded to various exercise prescription parameters. For example, both IL-6 and adiponectin showed optimal improvements under AE and higher-intensity interventions. Previous studies have demonstrated that high-intensity acute AE can significantly increase IL-6 and IL-10 levels, whereas moderate-intensity AE does not elicit similar responses (119). Consistently, RCTs in cancer populations have shown that 12 weeks of high-intensity AE lead to greater improvements in IL-6 and IL-10 compared to moderate-intensity AE (120). It has also been hypothesized that moderate-intensity interventions may be insufficient to induce significant changes in adiponectin levels (121). Although our regression models did not identify AE as a significant moderator for IGF-1, the results suggested that the most favorable changes in IGF-1 also depended on high-intensity exercise. Furthermore, an RCT in breast cancer patients reported that a combined high-intensity AE and RT program produced greater inhibition of breast cancer cell proliferation when serum was collected after an acute exercise session than when resting serum was obtained following six months of training (122). A recent RCT among breast cancer patients undergoing chemotherapy also indicated that beneficial effects of exercise on tumor biology were driven primarily by acute responses to individual sessions that exceeded a certain intensity threshold, rather than by long-term systemic training effects (123). Together with prior discussions on the roles of IL-6, adiponectin, IGF-1, and AMPK-related pathways in metabolic and inflammatory regulation, these findings suggest that high-intensity AE may serve as the most direct trigger for activating mechanisms that underlie exercise-induced metabolic and inflammatory improvements.

Moreover, AE demonstrated greater improvements in HOMA index compared to RT. Early animal studies have shown that the enhancement of skeletal muscle glucose uptake during acute exercise is highly specific to muscle fiber type (124). More recent animal experiments indicate that fast-twitch fibers exhibit stronger responses to exercise stimuli, whereas slow-twitch fibers respond less robustly (125). Additionally, recent findings suggest that although both AE and RT can improve insulin sensitivity through AMPK activation, RT may also induce more prolonged and elevated activation of mTORC1, which could partially offset the insulin-sensitizing effects of RT (126). In human studies, research in healthy males has shown that AE, in comparison to RT, more significantly promotes the phosphorylation of key molecules involved in glucose uptake and glycogen synthesis, such as AMPK, AS160, and glycogen synthase (127). Further studies have reported that RT leads to a smaller reduction in blood glucose compared to AE or combined AE+RT interventions (128). However, these findings do not diminish the value of RT. One RCT demonstrated that AE combined with RT during cancer treatment led to greater reductions in IL-6 compared to high-intensity interval training (HIIT) alone (129). Two RCTs reported that RT during cancer treatment effectively limited increases in TNF-α (130–132). Excessive expression of TNF-α has been shown to contribute to the development of insulin resistance (133, 134). Moreover, RT may mitigate treatment-induced elevations in IL-6, thereby alleviating cancer-related fatigue and pain (135). Taken together, high-intensity AE appears to provide systemic benefits in modulating metabolic and inflammatory biomarkers. Nonetheless, RT remains an indispensable complementary strategy within comprehensive exercise interventions for cancer patients. In addition, although current research highlights the positive role of enhancing PA in cancer prevention and treatment (136, 137), our findings did not reveal significant advantages of PA-focused exercise prescriptions (e.g., ≥10,000 steps/day walking routines) in improving metabolic or inflammatory biomarker levels.

Inflammatory markers such as TNF-α, IL-8, and IL-10 showed greater improvements when weekly exercise duration exceeded 280 minutes and total exercise volume surpassed 2,000 MET-min/week. These findings suggest that improvements in inflammatory biomarkers may require not only sufficient intensity per session but also an accumulation of weekly exercise volume. A large-scale RCT conducted by Brown and colleagues among colorectal cancer survivors compared the effects of 300 minutes versus 150 minutes per week of moderate-intensity AE on a range of health outcomes (50–52). The study emphasized that the dose–response relationship between exercise volume and inflammation was not linear. The optimal range for improving inflammatory markers through moderate-intensity AE was identified as 150–220 minutes per week. Similarly, another RCT among breast cancer survivors found that reductions in IL-6 concentrations were associated with increased total hours of moderate- or high-intensity AE (104). Our findings are generally consistent with these results, as our calculation of exercise volume included not only AE but also RT. Given that IL-6 in our analysis appeared to respond more strongly to per-session intensity rather than total duration, these findings collectively suggest that inflammatory biomarkers do not respond uniformly to exercise interventions.

Our findings indicate that when exercise intensity, intervention duration, and weekly exercise volume meet the minimum thresholds recommended by current exercise guidelines, improvements in glucose, leptin, and CRP are more pronounced when combined with caloric restriction. This is supported by two RCTs conducted in breast cancer survivors, which demonstrated that combined caloric restriction and exercise interventions yielded greater improvements in glucose, leptin, and CRP levels than caloric restriction alone (99, 100, 138). One proposed mechanism by which caloric restriction exerts metabolic benefits in cancer patients is through the reduction of circulating glucose, which promotes a metabolic shift from glucose to fat as the primary fuel source (2). This mechanism overlaps with those discussed earlier in our manuscript. Additionally, multiple RCTs have suggested that changes in CRP and other inflammatory markers may be related to changes in body weight in breast cancer patients (49, 103, 139). However, these findings do not diminish the independent role of exercise, as most biomarkers in our study did not show a strong interaction between exercise and caloric restriction. Moreover, an RCT in prostate cancer patients demonstrated that reductions in insulin following preoperative HIIT were independent of weight loss (140). Collectively, these findings suggest that for metabolic and inflammatory markers sensitive to fat reduction, combined caloric restrictions may offer enhanced benefits.

In addition, biomarkers that showed limited change in our study—such as total cholesterol, HDL-C, and LDL-C—exhibited distinct response patterns to exercise interventions. Total cholesterol (in studies lasting >25 weeks) and LDL-C (in studies >20 weeks) appeared to respond more favorably to long-term AE. LDL-C also required higher weekly exercise durations for improvement. HDL-C showed better responses when exercise was combined with caloric restriction. These findings suggest that improvements in cholesterol-related markers may require longer durations of intervention. However, the median duration of RCTs included in this review was only 12 weeks, which may explain the relatively small changes observed. Although recent studies have emphasized the importance of lipid metabolism in tumor growth and metastasis, and highlighted the potential of exercise to modulate lipid regulation in cancer patients, our findings further support this by showing significant improvements in triglyceride levels following exercise interventions (141, 142). Nonetheless, existing evidence only confirms the overall efficacy of exercise on lipid metabolism in cancer patients, while few RCTs have reported specific effects of different exercise prescription parameters on individual lipid biomarkers.

### 4.3 recommendations for optimizing exercise prescription parameters to improve inflammatory and metabolic biomarkers in cancer patients

Current exercise guidelines from the American College of Sports Medicine (ACSM) and Exercise & Sports Science Australia (ESSA) recommend a combination of AE and RT for cancer patients. The ACSM advises 150–300 minutes of moderate-intensity AE or 75–150 minutes of vigorous AE per week, combined with at least two sessions of RT (41). The ESSA further emphasizes maintaining at least moderate intensity and avoiding more than two consecutive rest days (42). Our findings indicate that the exercise doses recommended in current guidelines align with the optimal improvement ranges for key metabolic biomarkers, including glucose, insulin, triglycerides, and total cholesterol. Furthermore, combining regular exercise with caloric restriction appears to be more effective in reducing glucose, CRP, and leptin levels. Therefore, we suggest integrating caloric restriction with existing exercise prescriptions to enhance metabolic outcomes in cancer patients. In the included RCTs, caloric restriction was typically achieved by limiting processed meats, high-sugar and high-fat foods, sugary beverages, and alcohol, with an overall energy reduction of approximately 500–1000 kcal/day relative to baseline intake (48, 99, 100, 138, 143).

Our findings highlight the particular importance of high-intensity AE, especially for improving IL-6, IGF-1, and adiponectin, which play central roles in metabolic and inflammatory regulation. Moreover, TNF-α and IL-8 appeared to respond more favorably to interventions with longer weekly exercise durations (>280 minutes/week). Based on the conversion formulas used in this study, we recommend as a minimum effective dose for inflammation improvement: three sessions per week of high-intensity AE or HIIT (≥85% HRR), each lasting 40 minutes, in combination with two sessions of RT. Given concerns about the safety and adherence of high-intensity training in cancer populations, the implementation of such exercise prescriptions should strictly follow the general principles outlined in current guidelines, such as progressive intensity increases and individualized adjustment of exercise parameters. Notably, a recent systematic review by Mahdaviani et al. identified key enablers for HIIT adherence among cancer patients, including supervised delivery, standalone HIIT programs (without concurrent training), and relatively short session durations (144). In addition, Dias-da-Silva and colleagues proposed practical recommendations regarding HIIT format and prescription precision (145). Together, these studies represent important advancements in high-intensity exercise prescription for cancer patients and may serve as valuable references for clinicians designing individualized training programs.

Tailoring exercise prescriptions to different phases of cancer treatment is also necessary. The safety and efficacy of exercise during treatment have been demonstrated in multiple studies. A recent RCT in breast cancer patients undergoing neoadjuvant chemotherapy showed that combining AE and RT significantly reduced treatment discontinuation and may contribute to tumor shrinkage and pathological complete response (146). In our subgroup analysis, the Glucose–Insulin Group and Inflammatory Group showed smaller overall effects during treatment compared to the post-treatment phase. Multiple RCTs suggested that the impact of exercise on inflammation depends on the treatment modality, as cancer therapy induces a systemic inflammatory environment (129, 147). Another RCT found that concurrent endocrine therapy following radiotherapy may blunt exercise-induced improvements in metabolic and inflammatory biomarkers (143). Increases in IL-6, IGF-1, and TNF-α triggered by chemotherapy may be too substantial to be offset by exercise alone (121). Furthermore, treatment-related adverse effects may impair physiological adaptation to exercise during the early stages of intervention, thereby reducing its effectiveness (148, 149). These findings suggest that the complex pathophysiological milieu during cancer treatment may blunt the effectiveness of exercise in modulating metabolic and inflammatory biomarkers.

Our subgroup meta-regression for breast cancer patients showed that the optimal intensity range during treatment was ≤6.5 METs for Glucose–Insulin and Lipid groups, but ≥9 METs for the Inflammatory Group. This suggests that moderate-intensity exercise may be sufficient to improve metabolic outcomes during treatment, whereas enhancing anti-inflammatory effects likely requires higher intensity. One study argued that conventional linear progression models, while safe and feasible, may not adequately accommodate the physiological fluctuations caused by cancer treatment (28). Training intensity and volume should be proactively reduced during symptom peaks and gradually restored during recovery phases (150). Additionally, the feasibility and adherence to high-intensity exercise protocols must be carefully considered. An RCT in colorectal cancer patients reported that although overall adherence to the exercise prescription was high, adherence to HIIT was only 28.9%, compared to 95.7% for moderate-intensity continuous training (MICT) (151). A similar pattern was observed in breast cancer patients, where adherence to HIIT was lower than to MICT (152). Based on the above findings, how to balance the effectiveness and compliance of exercise intervention during cancer treatment is still a direction worthy of further study.

### 4.4 Limitations and future research directions

This study is not without limitations. First, due to the limited number of available trials, we excluded several biomarkers originally planned for analysis, including VEGF, IGFBP-1, and IL-1β. Second, few RCTs reported the effects of different exercise prescription parameters on lipid metabolism biomarkers, thereby restricting our understanding of how exercise modulates lipid metabolic pathways. Third, although background characteristics such as BMI and age were found to have moderating effects on several outcomes, the modeling in this study was based on aggregate rather than individual patient data (IPD), which limits the ability to adjust for confounding factors at the patient level. Fourth, incomplete reporting of exercise prescription parameters in several RCTs required imputation, which may have introduced inconsistency. Fifth, part of our mechanistic interpretations was informed by animal model evidence, which should be viewed as exploratory and hypothesis-generating rather than definitive. Finally, differences in baseline metabolic profiles, treatment regimens, and exercise tolerance across cancer types may have contributed to variability in intervention effects. Given the moderate-to-low quality of evidence based on risk of bias and GRADE assessments, our findings should be interpreted with caution and applied in clinical practice with consideration of individual patient characteristics.

Future research should prioritize the following directions. First, further mechanistic studies using tumor-bearing animal models are needed to elucidate the pathways through which exercise influences metabolism and inflammation. Second, studies should more frequently monitor the temporal dynamics of metabolic and inflammatory biomarkers throughout exercise interventions. Third, the specific effects of different exercise prescription parameters on lipid metabolism warrant further investigation. Fourth, IPD meta-analyses should be conducted to explore how patient background characteristics moderate the effects of exercise. Fifth, future studies should systematically evaluate the effects of varying caloric intake levels, dietary macronutrient composition, and their interactions with exercise interventions. Sixth, future research should explore whether exercise-induced remodeling of the TME differs by cancer type. Lastly, future research should examine the relationships between metabolic and inflammatory biomarkers and broader health outcomes in cancer populations.

## 5 Conclusion

Regular exercise confers modest but favorable effects on metabolic and inflammatory biomarkers in cancer patients. Meta-regression highlighted the importance of high-intensity aerobic exercise (HRR > 85%) in modulating IL-6, adiponectin, and IGF-1, as well as longer weekly exercise duration (>280 min/week) in improving TNF-α and IL-8. Mechanistically, high-intensity aerobic exercise may serve as a primary trigger for activating pathways that mediate metabolic and inflammatory improvements.

## Data Availability

The original contributions presented in the study are included in the article/**Supplementary Material**. Further inquiries can be directed to the corresponding author.

## References

[1] Coller HA . Is cancer a metabolic disease? Am J Pathol. (2014) 184:4–17. doi: 10.1016/j.ajpath.2013.07.035, PMID: 24139946 PMC3873478

[2] Gyamfi J Kim J Choi J . Cancer as a metabolic disorder. Int J Mol Sci. (2022) 23:1155. doi: 10.3390/ijms23031155, PMID: 35163079 PMC8835572

[3] Martínez-Reyes I Chandel NS . Cancer metabolism: looking forward. Nat Rev Cancer. (2021) 21:669–80. doi: 10.1038/s41568-021-00378-6, PMID: 34272515

[4] Fu Y Zou T Shen X Nelson PJ Li J Wu C . Lipid metabolism in cancer progression and therapeutic strategies. MedComm (2020). (2021) 2:27–59. doi: 10.1002/mco2.27, PMID: 34766135 PMC8491217

[5] Xu X Peng Q Jiang X Tan S Yang Y Yang W . Metabolic reprogramming and epigenetic modifications in cancer: from the impacts and mechanisms to the treatment potential. Exp Mol Med. (2023) 55:1357–70. doi: 10.1038/s12276-023-01020-1, PMID: 37394582 PMC10394076

[6] Faubert B Solmonson A DeBerardinis RJ . Metabolic reprogramming and cancer progression. Science. (2020) 368:eaaw5473. doi: 10.1126/science.aaw5473, PMID: 32273439 PMC7227780

[7] Braun S Bitton-Worms K LeRoith D . The link between the metabolic syndrome and cancer. Int J Biol Sci. (2011) 7:1003–15. doi: 10.7150/ijbs.7.1003, PMID: 21912508 PMC3164150

[8] Liu W Chakraborty B Safi R Kazmin D Chang CY McDonnell DP . Dysregulated cholesterol homeostasis results in resistance to ferroptosis increasing tumorigenicity and metastasis in cancer. Nat Commun. (2021) 12:5103. doi: 10.1038/s41467-021-25354-4, PMID: 34429409 PMC8385107

[9] Dev R Bruera E Dalal S . Insulin resistance and body composition in cancer patients. Ann Oncol. (2018) 29:ii18–26. doi: 10.1093/annonc/mdx815, PMID: 29506229

[10] Deng L Liu T Liu CA Zhang Q Song MM Lin SQ . The association of metabolic syndrome score trajectory patterns with risk of all cancer types. Cancer. (2024) 130:2150–9. doi: 10.1002/cncr.35235, PMID: 38462898

[11] Chlebowski RT Aragaki AK Pan K Simon MS Neuhouser ML Haque R . Breast cancer incidence and mortality by metabolic syndrome and obesity: The Women’s Health Initiative. Cancer. (2024) 130:3147–56. doi: 10.1002/cncr.35318, PMID: 38736319

[12] Li Y Liu C Shi J Zheng X Chen Y Liu X . The association of metabolic disorders and prognosis in cancer patients. BMC Cancer. (2025) 25:278. doi: 10.1186/s12885-025-13707-x, PMID: 39962450 PMC11834268

[13] Hotamisligil GS . Inflammation and metabolic disorders. Nature. (2006) 444:860–7. doi: 10.1038/nature05485, PMID: 17167474

[14] Deng T Lyon CJ Bergin S Caligiuri MA Hsueh WA . Obesity, inflammation, and cancer. Annu Rev Pathol. (2016) 11:421–49. doi: 10.1146/annurev-pathol-012615-044359, PMID: 27193454

[15] Xia L Oyang L Lin J Tan S Han Y Wu N . The cancer metabolic reprogramming and immune response. Mol Cancer. (2021) 20:28. doi: 10.1186/s12943-021-01316-8, PMID: 33546704 PMC7863491

[16] Vitale E Rizzo A Santa K Jirillo E . Associations between “Cancer risk”, “Inflammation” and “Metabolic syndrome”: A scoping review. Biol (Basel). (2024) 13:352. doi: 10.3390/biology13050352, PMID: 38785834 PMC11117847

[17] Ruan GT Xie HL Gong YZ Ge YZ Zhang Q Wang ZW . Prognostic importance of systemic inflammation and insulin resistance in patients with cancer: a prospective multicenter study. BMC Cancer. (2022) 22:700. doi: 10.1186/s12885-022-09752-5, PMID: 35752767 PMC9233357

[18] Conteduca V Caffo O Galli L Maugeri A Scarpi E Maines F . Association among metabolic syndrome, inflammation, and survival in prostate cancer. Urol Oncol. (2018) 36:240.e1–.e11. doi: 10.1016/j.urolonc.2018.01.007, PMID: 29402534

[19] Padoan A Plebani M Basso D . Inflammation and pancreatic cancer: focus on metabolism, cytokines, and immunity. Int J Mol Sci. (2019) 20:676. doi: 10.3390/ijms20030676, PMID: 30764482 PMC6387440

[20] Millares L Barreiro E Cortes R Martinez-Romero A Balcells C Cascante M . Tumor-associated metabolic and inflammatory responses in early stage non-small cell lung cancer: Local patterns and prognostic significance. Lung Cancer. (2018) 122:124–30. doi: 10.1016/j.lungcan.2018.06.015, PMID: 30032820

[21] Xie Y Liu F Wu Y Zhu Y Jiang Y Wu Q . Inflammation in cancer: therapeutic opportunities from new insights. Mol Cancer. (2025) 24:51. doi: 10.1186/s12943-025-02243-8, PMID: 39994787 PMC11849313

[22] Locasale JW . Diet and exercise in cancer metabolism. Cancer Discov. (2022) 12:2249–57. doi: 10.1158/2159-8290.CD-22-0096, PMID: 36062923 PMC9547953

[23] Zhu C Ma H He A Li Y He C Xia Y . Exercise in cancer prevention and anticancer therapy: Efficacy, molecular mechanisms and clinical information. Cancer Lett. (2022) 544:215814. doi: 10.1016/j.canlet.2022.215814, PMID: 35803475

[24] Larson EA Dalamaga M Magkos F . The role of exercise in obesity-related cancers: Current evidence and biological mechanisms. Semin Cancer Biol. (2023) 91:16–26. doi: 10.1016/j.semcancer.2023.02.008, PMID: 36871634

[25] Sheinboim D Parikh S Manich P Markus I Dahan S Parikh R . An exercise-induced metabolic shield in distant organs blocks cancer progression and metastatic dissemination. Cancer Res. (2022) 82:4164–78. doi: 10.1158/0008-5472.CAN-22-0237, PMID: 36084256 PMC9762351

[26] Hu C Tang J Gao Y Cao R . Effects of physical exercise on body fat and laboratory biomarkers in cancer patients: a meta-analysis of 35 randomized controlled trials. Support Care Cancer. (2022) 30:1–12. doi: 10.1007/s00520-022-07013-6, PMID: 35501513

[27] Dinas PC On Behalf Of The Students Of Module Introduction To Systematic R Karaventza M Liakou C Georgakouli K Bogdanos D . Combined effects of physical activity and diet on cancer patients: A systematic review and meta-analysis. Nutrients. (2024) 16:1749. doi: 10.3390/nu16111749, PMID: 38892682 PMC11175154

[28] Sasso JP Eves ND Christensen JF Koelwyn GJ Scott J Jones LW . A framework for prescription in exercise-oncology research. J Cachexia Sarcopenia Muscle. (2015) 6:115–24. doi: 10.1002/jcsm.12042, PMID: 26136187 PMC4458077

[29] Jones LW Eves ND Scott JM . Bench-to-bedside approaches for personalized exercise therapy in cancer. Am Soc Clin Oncol Educ Book. (2017) 37:684–94. doi: 10.1200/EDBK_173836, PMID: 28561646

[30] Zhou Y Jia N Ding M Yuan K . Effects of exercise on inflammatory factors and IGF system in breast cancer survivors: a meta-analysis. BMC Womens Health. (2022) 22:507. doi: 10.1186/s12905-022-02058-5, PMID: 36482346 PMC9730577

[31] Wang J He Y Kim AR Lee KH Choi SW . Effects of different types of exercise on inflammatory markers in cancer patients: A systematic review and Bayesian network meta-analysis. J Sports Sci. (2025) 43:1121–38. doi: 10.1080/02640414.2025.2486886, PMID: 40197224

[32] Hojman P Gehl J Christensen JF Pedersen BK . Molecular mechanisms linking exercise to cancer prevention and treatment. Cell Metab. (2018) 27:10–21. doi: 10.1016/j.cmet.2017.09.015, PMID: 29056514

[33] Tanner-Smith EE Tipton E Polanin JR . Handling complex meta-analytic data structures using robust variance estimates: A tutorial in R. J Dev Life-Course Criminol. (2016) 2:85–112. doi: 10.1007/s40865-016-0026-5

[34] Tipton E . Small sample adjustments for robust variance estimation with meta-regression. Psychol Methods. (2015) 20:375–93. doi: 10.1037/met0000011, PMID: 24773356

[35] Hedges LV Tipton E Johnson MC . Robust variance estimation in meta-regression with dependent effect size estimates. Res Synth Methods. (2010) 1:39–65. doi: 10.1002/jrsm.5, PMID: 26056092

[36] Van Lissa CJ . Small sample meta-analyses: Exploring heterogeneity using MetaForest. In: Small sample size solutions. Abingdon, UK: Routledge (2020). p. 186–202.

[37] Castells X Saez M Barcheni M Cunill R Serrano D López B . Placebo response and its predictors in attention deficit hyperactivity disorder: A meta-analysis and comparison of meta-regression and metaForest. Int J Neuropsychopharmacol. (2022) 25:26–35. doi: 10.1093/ijnp/pyab054, PMID: 34355753 PMC8756096

[38] Parr NJ Loan CM Tanner-Smith EE . Using machine learning to identify and investigate moderators of alcohol use intervention effects in meta-analyses. Alcohol Alcohol. (2022) 57:26–34. doi: 10.1093/alcalc/agab036, PMID: 33969377 PMC8753777

[39] Page MJ McKenzie JE Bossuyt PM Boutron I Hoffmann TC Mulrow CD . The PRISMA 2020 statement: an updated guideline for reporting systematic reviews. Bmj. (2021) 372:n71. doi: 10.1136/bmj.n71, PMID: 33782057 PMC8005924

[40] Caspersen CJ Powell KE Christenson GM . Physical activity, exercise, and physical fitness: definitions and distinctions for health-related research. Public Health Rep. (1985) 100:126–31., PMID: 3920711 PMC1424733

[41] Campbell KL Winters-Stone KM Wiskemann J May AM Schwartz AL Courneya KS . Exercise guidelines for cancer survivors: consensus statement from international multidisciplinary roundtable. Med Sci Sports Exerc. (2019) 51:2375–90. doi: 10.1249/MSS.0000000000002116, PMID: 31626055 PMC8576825

[42] Hayes SC Newton RU Spence RR Galvão DA . The Exercise and Sports Science Australia position statement: Exercise medicine in cancer management. J Sci Med Sport. (2019) 22:1175–99. doi: 10.1016/j.jsams.2019.05.003, PMID: 31277921

[43] Cumpston M Li T Page MJ Chandler J Welch VA Higgins JP . Updated guidance for trusted systematic reviews: a new edition of the Cochrane Handbook for Systematic Reviews of Interventions. Cochrane Database Syst Rev. (2019) 10:Ed000142. doi: 10.1002/14651858.ED000142, PMID: 31643080 PMC10284251

[44] Sterne JAC Savović J Page MJ Elbers RG Blencowe NS Boutron I . RoB 2: a revised tool for assessing risk of bias in randomised trials. Bmj. (2019) 366:l4898. doi: 10.1136/bmj.l4898, PMID: 31462531

[45] Guyatt G Oxman AD Akl EA Kunz R Vist G Brozek J . GRADE guidelines: 1. Introduction-GRADE evidence profiles and summary of findings tables. J Clin Epidemiol. (2011) 64:383–94. doi: 10.1016/j.jclinepi.2010.04.026, PMID: 21195583

[46] Rosenthal R Cooper H Hedges L . Parametric measures of effect size. In: The handbook of research synthesis, (USA, New York: Russell Sage Foundation), vol. 621. (1994). p. 231–44.

[47] Plonsky L Ghanbar H . Multiple regression in L2 research: A methodological synthesis and guide to interpreting R2 values. Modern Lang J. (2018) 102:713–31. doi: 10.1111/modl.12509

[48] Wright JL Schenk JM Gulati R Beatty SJ VanDoren M Lin DW . The Prostate Cancer Active Lifestyle Study (PALS): A randomized controlled trial of diet and exercise in overweight and obese men on active surveillance. Cancer. (2024) 130:2108–19. doi: 10.1002/cncr.35241, PMID: 38353455 PMC11527460

[49] Isanejad A Nazari S Gharib B Motlagh AG . Comparison of the effects of high-intensity interval and moderate-intensity continuous training on inflammatory markers, cardiorespiratory fitness, and quality of life in breast cancer patients. J Sport Health Sci. (2023) 12:674–89. doi: 10.1016/j.jshs.2023.07.001, PMID: 37423313 PMC10658315

[50] Brown JC Compton SLE Meyerhardt JA Spielmann G Yang S . The dose-response effect of aerobic exercise on inflammation in colon cancer survivors. Front Oncol. (2023) 13. doi: 10.3389/fonc.2023.1257767, PMID: 38148846 PMC10750999

[51] Brown JC Troxel AB Ky B Damjanov N Zemel BS Rickels MR . Dose-response effects of aerobic exercise among colon cancer survivors: A randomized phase II trial. Clin Colorectal Cancer. (2018) 17:32–40. doi: 10.1016/j.clcc.2017.06.001, PMID: 28669606 PMC5733696

[52] Brown JC Rickels MR Troxel AB Zemel BS Damjanov N Ky B . Dose-response effects of exercise on insulin among colon cancer survivors. Endocr Relat Cancer. (2018) 25:11–9. doi: 10.1530/ERC-17-0377, PMID: 29018055 PMC5736434

[53] Papadopoulos E Gillen J Moore D Au D Kurgan N Klentrou P . High-intensity interval training or resistance training versus usual care in men with prostate cancer on active surveillance: a 3-arm feasibility randomized controlled trial. Appl Physiol Nutr Metab. (2021) 46:1535–44. doi: 10.1139/apnm-2021-0365, PMID: 34380000

[54] Schmitz KH Ahmed RL Hannan PJ Yee D . Safety and efficacy of weight training in recent breast cancer survivors to alter body composition, insulin, and insulin-like growth factor axis proteins. Cancer Epidemiol Biomarkers Prev. (2005) 14:1672–80. doi: 10.1158/1055-9965.EPI-04-0736, PMID: 16030100

[55] Uth J Fristrup B Sørensen V Helge EW Christensen MK Kjærgaard JB . Exercise intensity and cardiovascular health outcomes after 12 months of football fitness training in women treated for stage I-III breast cancer: Results from the football fitness After Breast Cancer (ABC) randomized controlled trial. Prog Cardiovasc Dis. (2020) 63:792–9. doi: 10.1016/j.pcad.2020.08.002, PMID: 32800792

[56] Wang Y Jin B Paxton RJ Yang W Wang X Jiao Y . The effects of exercise on insulin, glucose, IGF-axis and CRP in cancer survivors: Meta-analysis and meta-regression of randomised controlled trials. Eur J Cancer Care (Engl). (2020) 29:e13186. doi: 10.1111/ecc.13186, PMID: 31823458

[57] Bruinsma TJ Dyer AM Rogers CJ Schmitz KH Sturgeon KM . Effects of diet and exercise-induced weight loss on biomarkers of inflammation in breast cancer survivors: A systematic review and meta-analysis. Cancer Epidemiol Biomarkers Prev. (2021) 30:1048–62. doi: 10.1158/1055-9965.EPI-20-1029, PMID: 33737299 PMC8172485

[58] Tan L Mei J Tang R Huang D Qi K Ossowski Z . Can exercise as a complementary technique manage inflammatory markers in women with breast cancer who are overweight and obese? A systematic review and meta-analysis. Complement Ther Med. (2025) 88:103119. doi: 10.1016/j.ctim.2024.103119, PMID: 39710346

[59] Al-Mhanna SB Batrakoulis A Norhayati MN Mohamed M Drenowatz C Irekeola AA . Combined aerobic and resistance training improves body composition, alters cardiometabolic risk, and ameliorates cancer-related indicators in breast cancer patients and survivors with overweight/obesity: A systematic review and meta-analysis of randomized controlled trials. J Sports Sci Med. (2024) 23:366–95. doi: 10.52082/jssm, PMID: 38841642 PMC11149074

[60] Kong L Gao R . Aerobic exercise combined with resistance exercise training improves cardiopulmonary function and blood lipid of patients with breast cancer: A systematic review and meta-analysis. Med (Baltimore). (2022) 101:e32391. doi: 10.1097/MD.0000000000032391, PMID: 36595800 PMC9794326

[61] Bettariga F Taaffe DR Borsati A Avancini A Pilotto S Lazzarini SG . Effects of exercise on inflammation in female survivors of nonmetastatic breast cancer: a systematic review and meta-analysis. J Natl Cancer Inst. (2025) djaf062. doi: 10.1093/jnci/djaf062, PMID: 40112254 PMC12505137

[62] Abbasi F Pourjalali H do Nascimento IJB Zargarzadeh N Mousavi SM Eslami R . The effects of exercise training on inflammatory biomarkers in patients with breast cancer: A systematic review and meta-analysis. Cytokine. (2022) 149:155712. doi: 10.1016/j.cyto.2021.155712, PMID: 34644675

[63] Kang DW Lee J Suh SH Ligibel J Courneya KS Jeon JY . Effects of exercise on insulin, IGF axis, adipocytokines, and inflammatory markers in breast cancer survivors: A systematic review and meta-analysis. Cancer Epidemiol Biomarkers Prev. (2017) 26:355–65. doi: 10.1158/1055-9965.EPI-16-0602, PMID: 27742668

[64] Lee J Hwang Y . The effects of exercise interventions on fatigue, body composition, physical fitness, and biomarkers in breast cancer patients during and after treatment: a systematic review and meta-analysis of randomized controlled trials. J Cancer Surviv. (2025) 1–29. doi: 10.1007/s11764-025-01772-x, PMID: 40056311

[65] Jiao Q Xu B Meng C Xu F Li S Zhong J . Effectiveness of aerobic exercise intervention on cardiovascular disease risk in female breast cancer: a systematic review with meta-analyses. BMC Public Health. (2024) 24:3355. doi: 10.1186/s12889-024-20592-9, PMID: 39623369 PMC11610245

[66] O’Neill HM . AMPK and exercise: glucose uptake and insulin sensitivity. Diabetes Metab J. (2013) 37:1–21. doi: 10.4093/dmj.2013.37.1.1, PMID: 23441028 PMC3579147

[67] Spaulding HR Yan Z . AMPK and the adaptation to exercise. Annu Rev Physiol. (2022) 84:209–27. doi: 10.1146/annurev-physiol-060721-095517, PMID: 35143330 PMC8919726

[68] Steinberg GR Hardie DG . New insights into activation and function of the AMPK. Nat Rev Mol Cell Biol. (2023) 24:255–72. doi: 10.1038/s41580-022-00547-x, PMID: 36316383

[69] Douen AG Ramlal T Rastogi S Bilan PJ Cartee GD Vranic M . Exercise induces recruitment of the “insulin-responsive glucose transporter”. Evidence for distinct intracellular insulin- and exercise-recruitable transporter pools in skeletal muscle. J Biol Chem. (1990) 265:13427–30. doi: 10.1016/S0021-9258(18)77362-6, PMID: 2199436

[70] Nesher R Karl IE Kipnis DM . Dissociation of effects of insulin and contraction on glucose transport in rat epitrochlearis muscle. Am J Physiol. (1985) 249:C226–32. doi: 10.1152/ajpcell.1985.249.3.C226, PMID: 3898861

[71] Richter EA Garetto LP Goodman MN Ruderman NB . Enhanced muscle glucose metabolism after exercise: modulation by local factors. Am J Physiol. (1984) 246:E476–82. doi: 10.1152/ajpendo.1984.246.6.E476, PMID: 6430094

[72] Koh HJ Hirshman MF He H Li Y Manabe Y Balschi JA . Adrenaline is a critical mediator of acute exercise-induced AMP-activated protein kinase activation in adipocytes. Biochem J. (2007) 403:473–81. doi: 10.1042/BJ20061479, PMID: 17253964 PMC1876380

[73] Shen Y Zhou H Jin W Lee HJ . Acute exercise regulates adipogenic gene expression in white adipose tissue. Biol Sport. (2016) 33:381–91. doi: 10.5604/20831862.1224395, PMID: 28090143 PMC5143777

[74] Gao Y Zhang W Zeng LQ Bai H Li J Zhou J . Exercise and dietary intervention ameliorate high-fat diet-induced NAFLD and liver aging by inducing lipophagy. Redox Biol. (2020) 36:101635. doi: 10.1016/j.redox.2020.101635, PMID: 32863214 PMC7365984

[75] Linecker M Frick L Kron P Limani P Kambakamba P Tschuor C . Exercise improves outcomes of surgery on fatty liver in mice: A novel effect mediated by the AMPK pathway. Ann Surg. (2020) 271:347–55. doi: 10.1097/SLA.0000000000002904, PMID: 30138163

[76] Takekoshi K Fukuhara M Quin Z Nissato S Isobe K Kawakami Y . Long-term exercise stimulates adenosine monophosphate-activated protein kinase activity and subunit expression in rat visceral adipose tissue and liver. Metabolism. (2006) 55:1122–8. doi: 10.1016/j.metabol.2006.04.007, PMID: 16839850

[77] Lin J Zhang X Sun Y Xu H Li N Wang Y . Exercise ameliorates muscular excessive mitochondrial fission, insulin resistance and inflammation in diabetic rats via irisin/AMPK activation. Sci Rep. (2024) 14:10658. doi: 10.1038/s41598-024-61415-6, PMID: 38724553 PMC11082241

[78] Carapeto P Iwasaki K Hela F Kahng J Alves-Wagner AB Middelbeek RJW . Exercise activates AMPK in mouse and human pancreatic islets to decrease senescence. Nat Metab. (2024) 6:1976–90. doi: 10.1038/s42255-024-01130-8, PMID: 39317751 PMC12005094

[79] Fix DK Counts BR Smuder AJ Sarzynski MA Koh HJ Carson JA . Wheel running improves fasting-induced AMPK signaling in skeletal muscle from tumor-bearing mice. Physiol Rep. (2021) 9:e14924. doi: 10.14814/phy2.14924, PMID: 34270178 PMC8284248

[80] Piguet AC Saran U Simillion C Keller I Terracciano L Reeves HL . Regular exercise decreases liver tumors development in hepatocyte-specific PTEN-deficient mice independently of steatosis. J Hepatol. (2015) 62:1296–303. doi: 10.1016/j.jhep.2015.01.017, PMID: 25623824

[81] Wang Y Yuan J Liu H Chen J Zou J Zeng X . Elevated meteorin-like protein from high-intensity interval training improves heart function via AMPK/HDAC4 pathway. Genes Dis. (2024) 11:101100. doi: 10.1016/j.gendis.2023.101100, PMID: 39281832 PMC11400619

[82] Møller AB Lønbro S Farup J Voss TS Rittig N Wang J . Molecular and cellular adaptations to exercise training in skeletal muscle from cancer patients treated with chemotherapy. J Cancer Res Clin Oncol. (2019) 145:1449–60. doi: 10.1007/s00432-019-02911-5, PMID: 30968255 PMC11810380

[83] Kelly M Keller C Avilucea PR Keller P Luo Z Xiang X . AMPK activity is diminished in tissues of IL-6 knockout mice: the effect of exercise. Biochem Biophys Res Commun. (2004) 320:449–54. doi: 10.1016/j.bbrc.2004.05.188, PMID: 15219849

[84] Zhu YP Brown JR Sag D Zhang L Suttles J . Adenosine 5’-monophosphate-activated protein kinase regulates IL-10-mediated anti-inflammatory signaling pathways in macrophages. J Immunol. (2015) 194:584–94. doi: 10.4049/jimmunol.1401024, PMID: 25512602 PMC4343033

[85] Sag D Carling D Stout RD Suttles J . Adenosine 5’-monophosphate-activated protein kinase promotes macrophage polarization to an anti-inflammatory functional phenotype. J Immunol. (2008) 181:8633–41. doi: 10.4049/jimmunol.181.12.8633, PMID: 19050283 PMC2756051

[86] Mancini SJ White AD Bijland S Rutherford C Graham D Richter EA . Activation of AMP-activated protein kinase rapidly suppresses multiple pro-inflammatory pathways in adipocytes including IL-1 receptor-associated kinase-4 phosphorylation. Mol Cell Endocrinol. (2017) 440:44–56. doi: 10.1016/j.mce.2016.11.010, PMID: 27840174 PMC5228585

[87] Zou Y Chen Z Sun C Yang D Zhou Z Peng X . Exercise intervention mitigates pathological liver changes in NAFLD zebrafish by activating SIRT1/AMPK/NRF2 signaling. Int J Mol Sci. (2021) 22:10940. doi: 10.3390/ijms222010940, PMID: 34681600 PMC8536011

[88] Rao E Zhang Y Zhu G Hao J Persson XM Egilmez NK . Deficiency of AMPK in CD8+ T cells suppresses their anti-tumor function by inducing protein phosphatase-mediated cell death. Oncotarget. (2015) 6:7944–58. doi: 10.18632/oncotarget.3501, PMID: 25760243 PMC4480727

[89] Puppa MJ White JP Velázquez KT Baltgalvis KA Sato S Baynes JW . The effect of exercise on IL-6-induced cachexia in the Apc (Min/+) mouse. J Cachexia Sarcopenia Muscle. (2012) 3:117–37. doi: 10.1007/s13539-011-0047-1, PMID: 22476915 PMC3374019

[90] White JP Puppa MJ Gao S Sato S Welle SL Carson JA . Muscle mTORC1 suppression by IL-6 during cancer cachexia: a role for AMPK. Am J Physiol Endocrinol Metab. (2013) 304:E1042–52. doi: 10.1152/ajpendo.00410.2012, PMID: 23531613 PMC3651620

[91] Solsona-Vilarrasa E Vousden KH . Obesity, white adipose tissue and cancer. FEBS J. (2025) 292:2189–207. doi: 10.1111/febs.17312, PMID: 39496581 PMC12062788

[92] Villarroya F Cereijo R Gavaldà-Navarro A Villarroya J Giralt M . Inflammation of brown/beige adipose tissues in obesity and metabolic disease. J Intern Med. (2018) 284:492–504. doi: 10.1111/joim.12803, PMID: 29923291

[93] Saltiel AR Olefsky JM . Inflammatory mechanisms linking obesity and metabolic disease. J Clin Invest. (2017) 127:1–4. doi: 10.1172/JCI92035, PMID: 28045402 PMC5199709

[94] Yan K . Recent advances in the effect of adipose tissue inflammation on insulin resistance. Cell Signal. (2024) 120:111229. doi: 10.1016/j.cellsig.2024.111229, PMID: 38763181

[95] Mu WJ Zhu JY Chen M Guo L . Exercise-mediated browning of white adipose tissue: its significance, mechanism and effectiveness. Int J Mol Sci. (2021) 22:11512. doi: 10.3390/ijms222111512, PMID: 34768943 PMC8583930

[96] Maliszewska K Kretowski A . Brown adipose tissue and its role in insulin and glucose homeostasis. Int J Mol Sci. (2021) 22:1530. doi: 10.3390/ijms22041530, PMID: 33546400 PMC7913527

[97] Brooks GC Blaha MJ Blumenthal RS . Relation of C-reactive protein to abdominal adiposity. Am J Cardiol. (2010) 106:56–61. doi: 10.1016/j.amjcard.2010.02.017, PMID: 20609648

[98] Paepegaey AC Genser L Bouillot JL Oppert JM Clément K Poitou C . High levels of CRP in morbid obesity: the central role of adipose tissue and lessons for clinical practice before and after bariatric surgery. Surg Obes Relat Dis. (2015) 11:148–54. doi: 10.1016/j.soard.2014.06.010, PMID: 25393045

[99] Lin D Sturgeon KMM Gordon BRR Brown JCC Sears DDD Sarwer DBB . WISER survivor trial: combined effect of exercise and weight loss interventions on adiponectin and leptin levels in breast cancer survivors with overweight or obesity. Nutrients. (2023) 15:3453. doi: 10.3390/nu15153453, PMID: 37571390 PMC10421485

[100] Sturgeon KM Brown JC Sears DD Sarwer DB Schmitz KH . WISER survivor trial: combined effect of exercise and weight loss interventions on inflammation in breast cancer survivors. Med Sci Sports Exerc. (2023) 55:209–15. doi: 10.1249/MSS.0000000000003050, PMID: 36170550 PMC9840668

[101] Winters-Stone KM Wood LJ Stoyles S Dieckmann NF . The effects of resistance exercise on biomarkers of breast cancer prognosis: A pooled analysis of three randomized trials. Cancer Epidemiol Biomarkers Prev. (2018) 27:146–53. doi: 10.1158/1055-9965.EPI-17-0766, PMID: 29141853 PMC5811190

[102] Arikawa AY Kaufman BC Raatz SK Kurzer MS . Effects of a parallel-arm randomized controlled weight loss pilot study on biological and psychosocial parameters of overweight and obese breast cancer survivors. Pilot Feasibil Stud. (2018) 4:17. doi: 10.1186/s40814-017-0160-9, PMID: 28702218 PMC5504770

[103] Jones LW Fels DR West M Allen JD Broadwater G Barry WT . Modulation of circulating angiogenic factors and tumor biology by aerobic training in breast cancer patients receiving neoadjuvant chemotherapy. Cancer Prev Res (Philadelphia Pa). (2013) 6:925–37. doi: 10.1158/1940-6207.CAPR-12-0416, PMID: 23842792 PMC3800005

[104] Pakiz B Bardwell W Rock C Mills P . Effects of a weight loss intervention on body mass, fitness, and inflammatory biomarkers in overweight or obese breast cancer survivors. Int J Behav Med. (2011) 18:333–41. doi: 10.1007/s12529-010-9079-8, PMID: 21336679 PMC3212681

[105] Kasprzak A . Insulin-like growth factor 1 (IGF-1) signaling in glucose metabolism in colorectal cancer. Int J Mol Sci. (2021) 22:6434. doi: 10.3390/ijms22126434, PMID: 34208601 PMC8234711

[106] Feng L Li B Xi Y Cai M Tian Z . Aerobic exercise and resistance exercise alleviate skeletal muscle atrophy through IGF-1/IGF-1R-PI3K/Akt pathway in mice with myocardial infarction. Am J Physiol Cell Physiol. (2022) 322:C164–c76. doi: 10.1152/ajpcell.00344.2021, PMID: 34852207

[107] Li B Feng L Wu X Cai M Yu JJ Tian Z . Effects of different modes of exercise on skeletal muscle mass and function and IGF-1 signaling during early aging in mice. J Exp Biol. (2022) 225:jeb244650. doi: 10.1242/jeb.244650, PMID: 36205111

[108] Yeves AM Godoy Coto J Pereyra EV Medina AJ Arbelaez LFG Cavalli FA . Apelin/APJ signaling in IGF-1-induced acute mitochondrial and antioxidant effects in spontaneously hypertensive rat myocardium. J Physiol Biochem. (2024) 80:949–59. doi: 10.1007/s13105-024-01055-6, PMID: 39453580

[109] Mohebinejad M Kazeminasab F Ghanbari Rad M Bagheri R Razi M Willoughby D . The combined effect of high-intensity interval training and time-restricted feeding on the AKT-IGF-1-mTOR signaling pathway in the muscle tissue of type 2 diabetic rats. Nutrients. (2025) 17:1404. doi: 10.3390/nu17091404, PMID: 40362714 PMC12073226

[110] Yu M King B Ewert E Su X Mardiyati N Zhao Z . Exercise Activates p53 and Negatively Regulates IGF-1 Pathway in Epidermis within a Skin Cancer Model. PloS One. (2016) 11:e0160939. doi: 10.1371/journal.pone.0160939, PMID: 27509024 PMC4979999

[111] Khoramipour K Chamari K Hekmatikar AA Ziyaiyan A Taherkhani S Elguindy NM . Adiponectin: structure, physiological functions, role in diseases, and effects of nutrition. Nutrients. (2021) 13:1180. doi: 10.3390/nu13041180, PMID: 33918360 PMC8066826

[112] Pham DV Park PH . Adiponectin triggers breast cancer cell death via fatty acid metabolic reprogramming. J Exp Clin Cancer Res. (2022) 41:9. doi: 10.1186/s13046-021-02223-y, PMID: 34986886 PMC8729140

[113] Chakraborty D Jin W Wang J . The bifurcated role of adiponectin in colorectal cancer. Life Sci. (2021) 278:119524. doi: 10.1016/j.lfs.2021.119524, PMID: 33887344 PMC8205988

[114] Diniz TA Aquino Júnior JCJ Mosele FC Cabral-Santos C Lima Junior EA Teixeira AAS . Exercise-induced AMPK activation and IL-6 muscle production are disturbed in adiponectin knockout mice. Cytokine. (2019) 119:71–80. doi: 10.1016/j.cyto.2019.03.009, PMID: 30903866

[115] Iwabu M Yamauchi T Okada-Iwabu M Sato K Nakagawa T Funata M . Adiponectin and AdipoR1 regulate PGC-1alpha and mitochondria by Ca(2+) and AMPK/SIRT1. Nature. (2010) 464:1313–9. doi: 10.1038/nature08991, PMID: 20357764

[116] Morinaga M Sako N Isobe M Lee-Hotta S Sugiura H Kametaka S . Aerobic exercise ameliorates cancer cachexia-induced muscle wasting through adiponectin signaling. Int J Mol Sci. (2021) 22:3110. doi: 10.3390/ijms22063110, PMID: 33803685 PMC8002946

[117] de Alcantara Borba D da Silva Alves E Rosa JPP Facundo LA Costa CMA Silva AC . Can IGF-1 serum levels really be changed by acute physical exercise? A systematic review and meta-analysis. J Phys Act Health. (2020) 17:575–84. doi: 10.1123/jpah.2019-0453, PMID: 32259791

[118] Otu LI Otu A . Adiponectin and the control of metabolic dysfunction: is exercise the magic bullet? Front Physiol. (2021) 12:651732. doi: 10.3389/fphys.2021.651732, PMID: 33897460 PMC8058350

[119] Wadley AJ Chen YW Lip GY Fisher JP Aldred S . Low volume-high intensity interval exercise elicits antioxidant and anti-inflammatory effects in humans. J Sports Sci. (2016) 34:1–9. doi: 10.1080/02640414.2015.1035666, PMID: 25915178

[120] Hooshmand Moghadam B Golestani F Bagheri R Cheraghloo N Eskandari M Wong A . The effects of high-intensity interval training vs. Moderate-intensity continuous training on inflammatory markers, body composition, and physical fitness in overweight/obese survivors of breast cancer: A randomized controlled clinical trial. Cancers (Basel). (2021) 13. doi: 10.3390/cancers13174386, PMID: 34503198 PMC8430701

[121] Febvey-Combes O Jobard E Rossary A Pialoux V Foucaut A-M Morelle M . Effects of an exercise and nutritional intervention on circulating biomarkers and metabolomic profiling during adjuvant treatment for localized breast cancer: results from the PASAPAS feasibility randomized controlled trial. Integr Cancer Ther. (2021) 20:1–10. doi: 10.1177/1534735420977666, PMID: 33655799 PMC7934026

[122] Dethlefsen C Lillelund C Midtgaard J Andersen C Pedersen BK Christensen JF . Exercise regulates breast cancer cell viability: systemic training adaptations versus acute exercise responses. Breast Cancer Res Treat. (2016) 159:469–79. doi: 10.1007/s10549-016-3970-1, PMID: 27601139

[123] PapadopetrakiA GiannopoulosA GiaskevitsT MoustogiannisA PappaM ZagouriF . The exercise-conditioned human serum and skeletal muscle cells secretome induce apoptosis in breast cancer cells. J Sport Health Sci. (2025), 101051. doi: 10.1016/j.jshs.2025.101051, PMID: 40334838

[124] James DE Kraegen EW Chisholm DJ . Muscle glucose metabolism in exercising rats: comparison with insulin stimulation. Am J Physiol. (1985) 248:E575–80. doi: 10.1152/ajpendo.1985.248.5.E575, PMID: 3887943

[125] Pataky MW Yu CS Nie Y Arias EB Singh M Mendias CL . Skeletal muscle fiber type-selective effects of acute exercise on insulin-stimulated glucose uptake in insulin-resistant, high-fat-fed rats. Am J Physiol Endocrinol Metab. (2019) 316:E695–e706. doi: 10.1152/ajpendo.00482.2018, PMID: 30753114 PMC6580167

[126] Kido K Sase K Yokokawa T Fujita S . Enhanced skeletal muscle insulin sensitivity after acute resistance-type exercise is upregulated by rapamycin-sensitive mTOR complex 1 inhibition. Sci Rep. (2020) 10:8509. doi: 10.1038/s41598-020-65397-z, PMID: 32444657 PMC7244536

[127] Camera DM Edge J Short MJ Hawley JA Coffey VG . Early time course of Akt phosphorylation after endurance and resistance exercise. Med Sci Sports Exerc. (2010) 42:1843–52. doi: 10.1249/MSS.0b013e3181d964e4, PMID: 20195183

[128] D’Souza NC Kesibi D Yeung C Shakeri D D’Souza AI Macpherson AK . The impact of sex, body mass index, age, exercise type and exercise duration on interstitial glucose levels during exercise. Sensors (Basel). (2023) 23:9059. doi: 10.3390/s23229059, PMID: 38005447 PMC10674905

[129] Hiensch AE Mijwel S Bargiela D Wengström Y May AM Rundqvist H . Inflammation mediates exercise effects on fatigue in patients with breast cancer. Med Sci Sports Exercise. (2021) 53:496–504. doi: 10.1249/MSS.0000000000002490, PMID: 32910094 PMC7886356

[130] Christensen JF Tolver A Andersen JL Rørth M Daugaard G Hojman P . Resistance training does not protect against increases in plasma cytokine levels among germ cell cancer patients during and after chemotherapy. J Clin Endocrinol Metab. (2014) 99:2967–76. doi: 10.1210/jc.2013-4495, PMID: 25050898

[131] Christensen JF Jones LW Tolver A Jørgensen LW Andersen JL Adamsen L . Safety and efficacy of resistance training in germ cell cancer patients undergoing chemotherapy: a randomized controlled trial. Br J Cancer. (2014) 111:8–16. doi: 10.1038/bjc.2014.273, PMID: 24867693 PMC4090736

[132] Hagstrom AD Marshall PWM Lonsdale C Papalia S Cheema BS Toben C . The effect of resistance training on markers of immune function and inflammation in previously sedentary women recovering from breast cancer: a randomized controlled trial. Breast Cancer Res Treat. (2016) 155:471–82. doi: 10.1007/s10549-016-3688-0, PMID: 26820653

[133] Youd JM Rattigan S Clark MG . Acute impairment of insulin-mediated capillary recruitment and glucose uptake in rat skeletal muscle *in vivo* by TNF-alpha. Diabetes. (2000) 49:1904–9. doi: 10.2337/diabetes.49.11.1904, PMID: 11078458

[134] Halse R Pearson SL McCormack JG Yeaman SJ Taylor R . Effects of tumor necrosis factor-alpha on insulin action in cultured human muscle cells. Diabetes. (2001) 50:1102–9. doi: 10.2337/diabetes.50.5.1102, PMID: 11334414

[135] Schmidt ME Meynköhn A Habermann N Wiskemann J Oelmann J Hof H . Resistance exercise and inflammation in breast cancer patients undergoing adjuvant radiation therapy: mediation analysis from a randomized, controlled intervention trial. Int J Radiat Oncol Biol Phys. (2016) 94:329–37. doi: 10.1016/j.ijrobp.2015.10.058, PMID: 26853341

[136] Albini A La Vecchia C Magnoni F Garrone O Morelli D Janssens JP . Physical activity and exercise health benefits: cancer prevention, interception, and survival. Eur J Cancer Prev. (2025) 34:24–39. doi: 10.1097/CEJ.0000000000000898, PMID: 38920329

[137] Misiąg W Piszczyk A Szymańska-Chabowska A Chabowski M . Physical activity and cancer care-A review. Cancers (Basel). (2022) 14. doi: 10.3390/cancers14174154, PMID: 36077690 PMC9454950

[138] Gnagnarella P Dragà D Raja S Baggi F Simoncini MC Sabbatini A . Physical activity and/or dietary intervention in overweight or obese breast cancer survivors: results of the InForma randomized trial. J Cancer Survivorship. (2024) 18:1732–46. doi: 10.1007/s11764-023-01415-z, PMID: 37418169

[139] Dieli-Conwright CM Harrigan M Cartmel B Chagpar A Bai Y Li FY . Impact of a randomized weight loss trial on breast tissue markers in breast cancer survivors. NPJ Breast Cancer. (2022) 8:29. doi: 10.1038/s41523-022-00396-z, PMID: 35256599 PMC8901848

[140] Kang D-W Field CJ Patel D Fairey AS Boule NG Dieli-Conwright CM . Effects of high-intensity interval training on cardiometabolic biomarkers in patients with prostate cancer undergoing active surveillance: a randomized controlled trial. Prostate Cancer Prostatic Dis. (2024) 28:469–74. doi: 10.1038/s41391-024-00867-3, PMID: 39009705

[141] Liu H Yang T Choi S . Modulation of lipid metabolism by exercise: exploring its potential as a therapeutic target in cancer endocrinology. Front Endocrinol (Lausanne). (2025) 16:1580559. doi: 10.3389/fendo.2025.1580559, PMID: 40487761 PMC12141028

[142] Peng Q Zhan C Shen Y Xu Y Ren B Feng Z . Blood lipid metabolic biomarkers are emerging as significant prognostic indicators for survival in cancer patients. BMC Cancer. (2024) 24:1549. doi: 10.1186/s12885-024-13265-8, PMID: 39695484 PMC11657272

[143] SenthilKumar G Schottstaedt AM Peterson LL Pedersen LN Chitambar CR Vistocky A . Stay on track: a pilot randomized control trial on the feasibility of a diet and exercise intervention in patients with breast cancer receiving radiotherapy. Cancer Res Commun. (2024) 4:1211–26. doi: 10.1158/2767-9764.CRC-23-0148, PMID: 38530195 PMC11075661

[144] Mahdaviani B Selk-Ghaffari M Sarzaeim M Thornton JS . Barriers and enablers of adherence to high-intensity interval training among patients with cancer: a systematic review and meta-analysis. Br J Sports Med. (2024) 58:1285–94. doi: 10.1136/bjsports-2024-108163, PMID: 39332844

[145] Dias-da-Silva G Jr. Panissa VLG Derchain SFM Ferreira MLV Telles GD Buzaglo GBB . High-intensity interval training for cancer patients: A review of key considerations for exercise prescription. Sports Med. (2025) 55:619–39. doi: 10.1007/s40279-024-02145-7, PMID: 39602033

[146] SchmidtME GoldschmidtS KreutzC MüllerJ SchneeweissA MayAM . Effects of aerobic or resistance exercise during neoadjuvant chemotherapy on tumor response and therapy completion in women with breast cancer: The randomized controlled BENEFIT trial. J Sport Health Sci. (2025) 101064. doi: 10.1016/j.jshs.2025.101064, PMID: 40447136

[147] Parent-Roberge H Fontvieille A Marechal R Wagner R Fulop T Pavic M . Effects of combined exercise training on the inflammatory profile of older cancer patients treated with systemic therapy. Brain Behav Immun - Health. (2020) 2:100016. doi: 10.1016/j.bbih.2019.100016, PMID: 38377414 PMC8474500

[148] Hamaker ME Prins MC Schiphorst AH van Tuyl SA Pronk A van den Bos F . Long-term changes in physical capacity after colorectal cancer treatment. J Geriatr Oncol. (2015) 6:153–64. doi: 10.1016/j.jgo.2014.10.001, PMID: 25454769

[149] Eng C . Toxic effects and their management: daily clinical challenges in the treatment of colorectal cancer. Nat Rev Clin Oncol. (2009) 6:207–18. doi: 10.1038/nrclinonc.2009.16, PMID: 19333227

[150] Kirkham AA Bland KA Zucker DS Bovard J Shenkier T McKenzie DC . Chemotherapy-periodized” Exercise to accommodate for cyclical variation in fatigue. Med Sci Sports Exerc. (2020) 52:278–86. doi: 10.1249/MSS.0000000000002151, PMID: 31490858

[151] Moraitis AM Rose NB Johnson AF Dunston ER Garrido-Laguna I Hobson P . Feasibility and acceptability of an mHealth, home-based exercise intervention in colorectal cancer survivors: A pilot randomized controlled trial. PloS One. (2023) 18:e0287152. doi: 10.1371/journal.pone.0287152, PMID: 37347792 PMC10286977

[152] Witlox L Velthuis MJ Boer JH Steins Bisschop CN Wall EV Meulen W . Attendance and compliance with an exercise program during localized breast cancer treatment in a randomized controlled trial: The PACT study. PloS One. (2019) 14:e0215517. doi: 10.1371/journal.pone.0215517, PMID: 31067223 PMC6505930

