# Supplementary material for: Effects of different exercise prescription parameters on metabolic and inflammatory biomarkers in cancer patients: a systematic review, meta-analysis, and meta-regression
Source: Front Immunol. 2025 Aug 14;16:1663560. doi: 10.3389/fimmu.2025.1663560 (PMC12390815; doi:10.3389/fimmu.2025.1663560)
Supplement: Supplementary file 1 [file Table1.docx]

**Supplementary information 1. PRISMA 2020 checklist.**

| **Section and Topic** | **Item #** | **Checklist item** | **Location where item is reported** |
| --- | --- | --- | --- |
| **TITLE** | | |  |
| Title | 1 | Identify the report as a systematic review. | 1 |
| **ABSTRACT** | | |  |
| Abstract | 2 | See the PRISMA 2020 for Abstracts checklist. | 2 |
| **INTRODUCTION** | | |  |
| Rationale | 3 | Describe the rationale for the review in the context of existing knowledge. | 6 |
| Objectives | 4 | Provide an explicit statement of the objective(s) or question(s) the review addresses. | 6 |
| **METHODS** | | |  |
| Eligibility criteria | 5 | Specify the inclusion and exclusion criteria for the review and how studies were grouped for the syntheses. | 7 |
| Information sources | 6 | Specify all databases, registers, websites, organisations, reference lists and other sources searched or consulted to identify studies. Specify the date when each source was last searched or consulted. | 7 |
| Search strategy | 7 | Present the full search strategies for all databases, registers and websites, including any filters and limits used. | 7 |
| Selection process | 8 | Specify the methods used to decide whether a study met the inclusion criteria of the review, including how many reviewers screened each record and each report retrieved, whether they worked independently, and if applicable, details of automation tools used in the process. | 7 |
| Data collection process | 9 | Specify the methods used to collect data from reports, including how many reviewers collected data from each report, whether they worked independently, any processes for obtaining or confirming data from study investigators, and if applicable, details of automation tools used in the process. | 7 |
| Data items | 10a | List and define all outcomes for which data were sought. Specify whether all results that were compatible with each outcome domain in each study were sought (e.g. for all measures, time points, analyses), and if not, the methods used to decide which results to collect. | 8 |
|  | 10b | List and define all other variables for which data were sought (e.g. participant and intervention characteristics, funding sources). Describe any assumptions made about any missing or unclear information. | 8 |
| Study risk of bias assessment | 11 | Specify the methods used to assess risk of bias in the included studies, including details of the tool(s) used, how many reviewers assessed each study and whether they worked independently, and if applicable, details of automation tools used in the process. | 8 |
| Effect measures | 12 | Specify for each outcome the effect measure(s) (e.g. risk ratio, mean difference) used in the synthesis or presentation of results. | 8 |
| Synthesis methods | 13a | Describe the processes used to decide which studies were eligible for each synthesis (e.g. tabulating the study intervention characteristics and comparing against the planned groups for each synthesis (item #5)). | 8 |
|  | 13b | Describe any methods required to prepare the data for presentation or synthesis, such as handling of missing summary statistics, or data conversions. | 8 |
|  | 13c | Describe any methods used to tabulate or visually display results of individual studies and syntheses. | 8 |
|  | 13d | Describe any methods used to synthesize results and provide a rationale for the choice(s). If meta-analysis was performed, describe the model(s), method(s) to identify the presence and extent of statistical heterogeneity, and software package(s) used. | 8 |
|  | 13e | Describe any methods used to explore possible causes of heterogeneity among study results (e.g. subgroup analysis, meta-regression). | 8 |
|  | 13f | Describe any sensitivity analyses conducted to assess robustness of the synthesized results. | 8 |
| Reporting bias assessment | 14 | Describe any methods used to assess risk of bias due to missing results in a synthesis (arising from reporting biases). | 8 |
| Certainty assessment | 15 | Describe any methods used to assess certainty (or confidence) in the body of evidence for an outcome. | 8 |
| **RESULTS** | | |  |
| Study selection | 16a | Describe the results of the search and selection process, from the number of records identified in the search to the number of studies included in the review, ideally using a flow diagram. | 10 |
|  | 16b | Cite studies that might appear to meet the inclusion criteria, but which were excluded, and explain why they were excluded. | 10 |
| Study characteristics | 17 | Cite each included study and present its characteristics. | 10 |
| Risk of bias in studies | 18 | Present assessments of risk of bias for each included study. | 11 |
| Results of individual studies | 19 | For all outcomes, present, for each study: (a) summary statistics for each group (where appropriate) and (b) an effect estimate and its precision (e.g. confidence/credible interval), ideally using structured tables or plots. | 11 |
| Results of syntheses | 20a | For each synthesis, briefly summarise the characteristics and risk of bias among contributing studies. | 11 |
|  | 20b | Present results of all statistical syntheses conducted. If meta-analysis was done, present for each the summary estimate and its precision (e.g. confidence/credible interval) and measures of statistical heterogeneity. If comparing groups, describe the direction of the effect. | 11 |
|  | 20c | Present results of all investigations of possible causes of heterogeneity among study results. | 11 |
|  | 20d | Present results of all sensitivity analyses conducted to assess the robustness of the synthesized results. | 11 |
| Reporting biases | 21 | Present assessments of risk of bias due to missing results (arising from reporting biases) for each synthesis assessed. | 11 |
| Certainty of evidence | 22 | Present assessments of certainty (or confidence) in the body of evidence for each outcome assessed. | 11 |
| **DISCUSSION** | | |  |
| Discussion | 23a | Provide a general interpretation of the results in the context of other evidence. | 15 |
|  | 23b | Discuss any limitations of the evidence included in the review. | 24 |
|  | 23c | Discuss any limitations of the review processes used. | 24 |
|  | 23d | Discuss implications of the results for practice, policy, and future research. | 25 |
| **OTHER INFORMATION** | | |  |
| Registration and protocol | 24a | Provide registration information for the review, including register name and registration number, or state that the review was not registered. | 7 |
|  | 24b | Indicate where the review protocol can be accessed, or state that a protocol was not prepared. | 7 |
|  | 24c | Describe and explain any amendments to information provided at registration or in the protocol. | 7 |
| Support | 25 | Describe sources of financial or non-financial support for the review, and the role of the funders or sponsors in the review. | 26 |
| Competing interests | 26 | Declare any competing interests of review authors. | 26 |
| Availability of data, code and other materials | 27 | Report which of the following are publicly available and where they can be found: template data collection forms; data extracted from included studies; data used for all analyses; analytic code; any other materials used in the review. | 26 |

*From:*  Page MJ, McKenzie JE, Bossuyt PM, Boutron I, Hoffmann TC, Mulrow CD, et al. The PRISMA 2020 statement: an updated guideline for reporting systematic reviews. BMJ 2021;372:n71. doi: 10.1136/bmj.n71. This work is licensed under CC BY 4.0. To view a copy of this license, visit

**Supplementary information 2. Search Strategy.**

| Database | Search Strategy |
| --- | --- |
| Pubmed | #1. "Exercise"[MeSH Terms] OR "exercise therapy"[MeSH Terms] OR "physical exercise"[Title/Abstract] OR "physical exercises"[Title/Abstract] OR "physical activity"[Title/Abstract] OR "activities physical"[Title/Abstract] OR "activity physical"[Title/Abstract] OR "physical activities"[Title/Abstract] OR "aerobic exercise"[Title/Abstract] OR "aerobic exercises"[Title/Abstract] OR "exercises aerobic"[Title/Abstract] OR "isometric exercise"[Title/Abstract] OR "isometric exercises"[Title/Abstract] OR "acute exercise"[Title/Abstract] OR "exercise training"[Title/Abstract] OR "exercise therapy"[MeSH Terms] OR "rehabilitation exercise"[Title/Abstract] OR "exercise rehabilitation"[Title/Abstract] OR "exercises rehabilitation"[Title/Abstract] OR "resistance training"[MeSH Terms] OR "strength training"[Title/Abstract] OR (("Weight-Lifting"[MeSH Terms] OR ("weight"[All Fields] AND "lifting"[All Fields]) OR "Weight-Lifting"[All Fields]) AND "strengthening program"[Title/Abstract]) OR "weight lifting exercise program"[Title/Abstract] OR "endurance training"[MeSH Terms] OR "high intensity interval training"[MeSH Terms] OR "circuit based exercise"[MeSH Terms] OR "Pilates"[Title/Abstract] OR "Walking"[Title/Abstract]  #2. "Cancer"[Title/Abstract] OR "tumor"[Title/Abstract] OR "neoplasm"[Title/Abstract] OR "oncology"[Title/Abstract] OR "chemotherapy"[Title/Abstract] OR "radiotherapy"[Title/Abstract] OR "cancer survivors"[MeSH Terms] OR "cancer survivor"[Title/Abstract] OR "survivors cancer"[Title/Abstract] OR "long term cancer survivor"[Title/Abstract] OR "long term cancer survivors"[Title/Abstract] OR "cancer survivorship"[Title/Abstract] OR "neoplasms"[MeSH Terms]  #3. "Biomarkers"[MeSH Terms] OR "Biomarkers"[Title/Abstract] OR "blood biomarkers"[Title/Abstract] OR "inflammatory markers"[Title/Abstract] OR "Cytokines"[Title/Abstract] OR "c reactive protein"[Title/Abstract] OR "CRP"[Title/Abstract] OR "Interleukin"[Title/Abstract] OR "TNF-alpha"[Title/Abstract] OR "Adipokines"[Title/Abstract] OR "Leptin"[Title/Abstract] OR "Adiponectin"[Title/Abstract] OR "insulin resistance"[Title/Abstract] OR "lipid profile"[Title/Abstract] OR "Cholesterol"[Title/Abstract] OR "Triglycerides"[Title/Abstract] OR "Glucose"[Title/Abstract] OR "HbA1c"[Title/Abstract]  #4. "randomized controlled trial"[Publication Type] OR "randomized"[Title/Abstract] OR "placebo"[Title/Abstract]  #5. #1 AND #2 AND #3 AND #4 |
| Embase | #1. ('exercise'/exp OR 'physical exercise':ti,ab OR 'physical exercises':ti,ab OR 'physical activity':ti,ab OR 'activities, physical':ti,ab OR 'activity, physical':ti,ab OR 'physical activities':ti,ab OR 'aerobic exercise':ti,ab OR 'isometric exercise':ti,ab OR 'acute exercise':ti,ab OR 'exercise training':ti,ab OR 'rehabilitation exercise':ti,ab OR 'exercise therapy'/exp OR 'resistance training'/exp OR 'strength training':ti,ab OR 'weight lifting strengthening program':ti,ab OR 'endurance training'/exp OR 'high intensity interval training'/exp OR 'circuit-based exercise'/exp OR 'taichi':ti,ab OR 'yoga':ti,ab OR 'pilates':ti,ab OR 'qi gong':ti,ab OR 'baduanjin':ti,ab OR 'walking':ti,ab)  #2. ('cancer'/exp OR 'tumor'/exp OR 'neoplasm'/exp OR 'oncology':ti,ab OR 'chemotherapy':ti,ab OR 'radiotherapy':ti,ab OR 'cancer survivor'/exp OR 'long-term cancer survivor':ti,ab OR 'cancer survivorship':ti,ab)  #3. ('biomarker'/exp OR 'biological marker':ti,ab OR 'blood biomarker':ti,ab OR 'inflammatory marker':ti,ab OR 'cytokine'/exp OR 'c-reactive protein'/exp OR 'tumor necrosis factor alpha':ti,ab OR 'interleukin-6':ti,ab OR 'interleukin-10':ti,ab OR 'adipokine':ti,ab OR 'leptin':ti,ab OR 'adiponectin':ti,ab OR 'insulin resistance':ti,ab OR 'lipid profile':ti,ab OR 'cholesterol':ti,ab OR 'triglycerides':ti,ab OR 'glucose':ti,ab OR 'hba1c':ti,ab OR 'oxidative stress markers':ti,ab OR 'hormonal biomarkers':ti,ab OR 'genetic marker'/exp OR 'immune markers':ti,ab)  #4. ('randomized controlled trial'/exp OR 'randomized controlled trials':ti,ab OR 'randomized trial':ti,ab OR 'controlled clinical trial'/exp OR 'controlled trial':ti,ab OR 'random allocation':ti,ab OR 'double-blind method'/exp OR 'single-blind method'/exp OR 'placebo'/exp OR 'placebo-controlled':ti,ab OR 'randomized':ti,ab OR 'clinical trial':ti,ab OR 'trial protocol':ti,ab OR 'double blind':ti,ab OR 'single blind':ti,ab OR 'crossover trial'/exp OR 'parallel group':ti,ab OR 'random assignment':ti,ab OR 'multicenter study'/exp OR 'multicenter trial':ti,ab OR 'randomization'/exp OR 'randomized study':ti,ab OR 'randomised':ti,ab OR 'randomised controlled trial':ti,ab OR 'sham control':ti,ab OR 'placebo effect':ti,ab OR 'placebo response':ti,ab OR 'control group'/exp OR 'trial design'/exp OR 'intervention study'/exp)  #5. #1 AND #2 AND #3 AND #4 |
| Web of Science | #1. ('randomized controlled trial'/exp OR 'randomized controlled trials':ti,ab OR 'randomized trial':ti,ab OR 'controlled clinical trial'/exp OR 'controlled trial':ti,ab OR 'random allocation':ti,ab OR 'double-blind method'/exp OR 'single-blind method'/exp OR 'placebo'/exp OR 'placebo-controlled':ti,ab OR 'randomized':ti,ab OR 'clinical trial':ti,ab OR 'trial protocol':ti,ab OR 'double blind':ti,ab OR 'single blind':ti,ab OR 'crossover trial'/exp OR 'parallel group':ti,ab OR 'random assignment':ti,ab OR 'multicenter study'/exp OR 'multicenter trial':ti,ab OR 'randomization'/exp OR 'randomized study':ti,ab OR 'randomised':ti,ab OR 'randomised controlled trial':ti,ab OR 'sham control':ti,ab OR 'placebo effect':ti,ab OR 'placebo response':ti,ab OR 'control group'/exp OR 'trial design'/exp OR 'intervention study'/exp)  #2. TS=("cancer" OR "tumor" OR "neoplasm" OR "oncology" OR "chemotherapy" OR "radiotherapy" OR "cancer survivor" OR "long-term cancer survivor" OR "cancer survivorship")  #3. TS=("biomarker" OR "biological marker" OR "blood biomarker" OR "inflammatory marker" OR "cytokine" OR "c-reactive protein" OR "tumor necrosis factor alpha" OR "interleukin-6" OR "interleukin-10" OR "adipokine" OR "leptin" OR "adiponectin" OR "insulin resistance" OR "lipid profile" OR "cholesterol" OR "triglycerides" OR "glucose" OR "hba1c" OR "oxidative stress markers" OR "hormonal biomarkers" OR "genetic marker" OR "immune markers")  #4. TS=("randomized controlled trial" OR "randomized controlled trials" OR "randomized trial" OR "controlled clinical trial" OR "controlled trial" OR "random allocation" OR "double-blind method" OR "single-blind method" OR "placebo" OR "placebo-controlled" OR "randomized" OR "clinical trial" OR "trial protocol" OR "double blind" OR "single blind" OR "crossover trial" OR "parallel group" OR "random assignment" OR "multicenter study" OR "multicenter trial" OR "randomization" OR "randomized study" OR "randomised" OR "randomised controlled trial" OR "sham control" OR "placebo effect" OR "placebo response" OR "control group" OR "trial design" OR "intervention study")  #5. #1 AND #2 AND #3 AND #4 |
| Cochrane library | #1. ("exercise" OR "physical exercise" OR "physical exercises" OR "physical activity" OR "activities, physical" OR "activity, physical" OR "physical activities" OR "aerobic exercise" OR "isometric exercise" OR "acute exercise" OR "exercise training" OR "rehabilitation exercise" OR "exercise therapy" OR "resistance training" OR "strength training" OR "weight lifting strengthening program" OR "endurance training" OR "high intensity interval training" OR "circuit-based exercise" OR "taichi" OR "yoga" OR "pilates" OR "qi gong" OR "baduanjin" OR "walking")  #2. ("colorectal cancer" OR "colon cancer" OR "rectal cancer" OR "colorectal neoplasm" OR "colorectal carcinoma" OR "colon neoplasm" OR "rectal neoplasm" OR "colon carcinoma" OR "rectal carcinoma" OR "colorectal adenocarcinoma" OR "colorectal tumor" OR "colorectal malignancy" OR "colorectal oncology" OR "colorectal cancer survivor" OR "long-term colorectal cancer survivor" OR "colorectal cancer survivorship" OR "chemotherapy" OR "radiotherapy")  #3. (("fatigue" OR "tiredness" OR "physical fatigue" OR "mental fatigue" OR "chronic fatigue syndrome" OR "fatigue severity scale" OR "fatigue resistance")OR ("healthy" OR "health status" OR "physical health" OR "well-being" OR "mental health" OR "general health" OR "health-related quality of life" OR "self-rated health" OR "health survey" OR "sf-36" OR "sf-12" OR "sf-8" OR "hrqol")OR ("physical function" OR "physical fitness" OR "physical performance" OR "physical ability" OR "functional ability" OR "functional capacity" OR "functional performance" OR "daily living activities" OR "adl" OR "physical endurance" OR "functional decline" OR "disability" OR "physical independence")OR ("athletic ability" OR "physical aptitude" OR "sports performance" OR "endurance" OR "exercise capacity" OR "exercise performance" OR "cardiorespiratory fitness" OR "aerobic capacity" OR "muscle performance" OR "strength performance") OR ("biomarker" OR "biological marker" OR "inflammatory marker" OR "cytokine" OR "c-reactive protein" OR "tumor necrosis factor alpha" OR "interleukin-6" OR "interleukin-10" OR "biomarker panel" OR "metabolic markers" OR "oxidative stress markers" OR "hormonal biomarkers" OR "genetic marker" OR "immune markers") OR ("muscle strength" OR "muscle power" OR "muscle endurance" OR "grip strength" OR "isometric strength" OR "dynamic strength" OR "leg strength" OR "upper body strength" OR "lower body strength" OR "muscle function"))  #4. ("randomized controlled trial" OR "randomized controlled trials" OR "randomized trial" OR "controlled clinical trial" OR "controlled trial" OR "random allocation" OR "double-blind method" OR "single-blind method" OR "placebo" OR "placebo-controlled" OR "randomized" OR "clinical trial" OR "trial protocol" OR "double blind" OR "single blind" OR "crossover trial" OR "parallel group" OR "random assignment" OR "multicenter study" OR "multicenter trial" OR "randomization" OR "randomized study" OR "randomised" OR "sham control" OR "placebo effect" OR "placebo response" OR "control group" OR "trial design" OR "intervention study")  #5. #1 AND #2 AND #3 AND #4 |
| EBSCOhost | #1. (MH "Exercise" OR TI "physical exercise" OR AB "physical exercise" OR TI "physical exercises" OR AB "physical exercises" OR TI "physical activity" OR AB "physical activity" OR TI "activities, physical" OR AB "activities, physical" OR TI "aerobic exercise" OR AB "aerobic exercise" OR TI "isometric exercise" OR AB "isometric exercise" OR TI "acute exercise" OR AB "acute exercise" OR TI "exercise training" OR AB "exercise training" OR TI "rehabilitation exercise" OR AB "rehabilitation exercise" OR (MH "Exercise Therapy") OR (MH "Resistance Training") OR TI "strength training" OR AB "strength training" OR TI "weight lifting strengthening program" OR AB "weight lifting strengthening program" OR (MH "Endurance Training") OR (MH "High Intensity Interval Training") OR (MH "Circuit Training") OR TI "taichi" OR AB "taichi" OR TI "yoga" OR AB "yoga" OR TI "pilates" OR AB "pilates" OR TI "qi gong" OR AB "qi gong" OR TI "baduanjin" OR AB "baduanjin" OR TI "walking" OR AB "walking")  #2. (MH "Neoplasms" OR TI "cancer" OR AB "cancer" OR TI "tumor" OR AB "tumor" OR TI "neoplasm" OR AB "neoplasm" OR TI "oncology" OR AB "oncology" OR TI "chemotherapy" OR AB "chemotherapy" OR TI "radiotherapy" OR AB "radiotherapy" OR (MH "Cancer Survivors") OR TI "long-term cancer survivor" OR AB "long-term cancer survivor" OR TI "cancer survivorship" OR AB "cancer survivorship")  #3. (MH "Biomarkers" OR TI "biomarker" OR AB "biomarker" OR TI "biological marker" OR AB "biological marker" OR TI "blood biomarker" OR AB "blood biomarker" OR TI "inflammatory marker" OR AB "inflammatory marker" OR (MH "Cytokines") OR TI "cytokine" OR AB "cytokine" OR (MH "C-Reactive Protein") OR TI "c-reactive protein" OR AB "c-reactive protein" OR TI "tumor necrosis factor alpha" OR AB "tumor necrosis factor alpha" OR TI "interleukin-6" OR AB "interleukin-6" OR TI "interleukin-10" OR AB "interleukin-10" OR TI "adipokine" OR AB "adipokine" OR TI "leptin" OR AB "leptin" OR TI "adiponectin" OR AB "adiponectin" OR TI "insulin resistance" OR AB "insulin resistance" OR TI "lipid profile" OR AB "lipid profile" OR TI "cholesterol" OR AB "cholesterol" OR TI "triglycerides" OR AB "triglycerides" OR TI "glucose" OR AB "glucose" OR TI "hba1c" OR AB "hba1c" OR TI "oxidative stress markers" OR AB "oxidative stress markers" OR TI "hormonal biomarkers" OR AB "hormonal biomarkers" OR (MH "Genetic Markers") OR TI "immune markers" OR AB "immune markers")  #4. (MH "Randomized Controlled Trials" OR TI "randomized controlled trials" OR AB "randomized controlled trials" OR TI "randomized trial" OR AB "randomized trial" OR (MH "Controlled Clinical Trials") OR TI "controlled trial" OR AB "controlled trial" OR TI "random allocation" OR AB "random allocation" OR (MH "Double-Blind Method") OR (MH "Single-Blind Method") OR (MH "Placebos") OR TI "placebo" OR AB "placebo" OR TI "randomized" OR AB "randomized" OR TI "clinical trial" OR AB "clinical trial" OR TI "trial protocol" OR AB "trial protocol" OR TI "double blind" OR AB "double blind" OR TI "single blind" OR AB "single blind" OR (MH "Crossover Design") OR TI "parallel group" OR AB "parallel group" OR TI "random assignment" OR AB "random assignment" OR (MH "Multicenter Studies") OR TI "multicenter trial" OR AB "multicenter trial" OR TI "randomization" OR AB "randomization" OR TI "randomised" OR AB "randomised" OR TI "sham control" OR AB "sham control" OR TI "placebo effect" OR AB "placebo effect" OR TI "control group" OR AB "control group" OR (MH "Trial Design") OR (MH "Intervention Studies"))  #5. #1 AND #2 AND #3 AND #4 |

**Supplementary information 3. Information on RCTs Included in the Meta-Analysis.**

Table S1. Basic information of included studies

| No. | Study | Study registration number | Cancer type | Timing | Type | Duration (week) | Intervening measure and intensity | Single Exercise Intensity (MET) | Weekly exercise duration (min/week) | Weekly exercise volume (MET-min/week) | Supervision | Other Intervention |
| --- | --- | --- | --- | --- | --- | --- | --- | --- | --- | --- | --- | --- |
| 1 | Wright2024 [1] | NCT02454517 | PCa | Survivor | AE | 24 | 70-85% HRR. | 8 | 150 | 1200 | No | Calorie restriction |
| 2 | SenthilKumar2024 [2] |  | BC | During | AE+RT | 12 | AE: 30 min. RT: 30min. | 4 | 210 | 840 | No | Calorie restriction |
| 3 | Kang2024 [3] | NCT03203460 | PCa | Before | AE | 12 | AE, 42 min/time. 5 min warm-up, 60% VO2peak; 2 min high-intensity (HI) exercise, 85-95% VO2peak; 2 min moderate-intensity (MI) exercise, 40% VO2peak; 5-8 bouts; 5 min cool-down. | 9 | 126 | 1134 | Yes | No |
| 4 | Gnagnarella2023 [4] | NCT02622711 and ISRCTN53325751 | BC | Survivor | PA | 24 | 60 min moderate-intensity physical activity (MPA) or ≥ 30 min vigorous-intensity physical activity (VPA). | 4.5 | 210 | 945 | No | Calorie restriction |
| 5 | Sturgeon2023,Lin2023 [5, 6] | NCT01515124 | BC | Survivor | AE+RT | 52 | RT: 90 min. | 4 | 360 | 1440 | Half | Calorie restriction |
| 6 | Martins2023 [7] | NCT03644329 | BC | Survivor | RT | 12 | 4 RT, 80% 1RM, 8-12 reps/set, 3 sets. | 4 | 144 | 576 | No | No |
| 7 | Isanejad2023 [8] | IRCT20200208046418N1 | BC | Survivor | AE | 12 | HIIT, 32 min. 5 min warm-up,50%-60% VO2peak (65%-75% HRR); 4×4 min 90% VO2peak (95% HRR); 3×3 min 60% VO2peak (75% HRR); 3 min cool-down 50%-60% VO2peak (65%-75% HRR). | 10 | 96 | 960 | No | No |
|  |  |  |  |  | AE | 12 | AE, 41 min. warm-up 5 min;50%-60% VO2peak (65%-75% HRR); 33 min AE 60% VO2peak (75% HRR) ; 3 min cool-down 50%-60% VO2peak (65%-75% HRR). | 7 | 123 | 861 | No | No |
| 8 | Falz2023, Darmochwal2023 [9, 10] | DRKS00020499 | Mixed | Survivor | AE+RT | 24 | RT, 30 min: 5 min warm-up; 5 RT, 4 sets; 5 min cool-down.  AE: 75% HRR. | 6 | 186 | 1116 | No | No |
| 9 | Demark-Wahnefried2023 [11] | NCT04132219 | Mixed | Survivor | AE+RT | 24 |  | 4 | 150 | 600 | Yes | Calorie restriction |
| 10 | Cartmel2023 [12] | NCT02107066 | OC | Survivor | AE | 24 | 150 min/week MI AE | 7 | 150 | 1050 | No | No |
| 11 | Brown2023, Brown2018_E, Brown2018_C [13-15] | NCT02250053 | CRC | Survivor | AE | 24 | 50−70% HRR | 4.5 | 150 | 675 | No | No |
|  |  |  |  |  | AE | 24 | 50−70% HRR | 4.5 | 300 | 1350 | No | No |
| 12 | Bojesen2023 [16] | NCT04167436 | CRC | Before | AE+RT | 4 | HIIT, 21 min. 5 min warm-up; 4×3 min HIIT, 95% VO2max; 3×3 min recovery, 30% VO2max. RT, 8-12 reps/set, 3 sets. | 10 | 210 | 2100 | Yes | No |
| 13 | Moon2022 [17] | NCT03397030 | PCa | Before | AE+RT | 24 | AE: 30 min, 40-60% HRR. RT: 8 RT, 15 reps/1set, 3sets. | 3.5 | 270 | 945 | No | No |
| 14 | Lee2022 [18] | PRE20220127-002 | BC | Survivor | RT | 12 | 8 RT, 50 min/time, 80% 1RM, 8 reps/set, 4 sets. | 4 | 150 | 600 | Yes | No |
| 15 | Hwang2022 [19] | KCT0004796 | CRC | Survivor | RT | 6 | RT, 30 min/time. 6 types of RT, 20 min, 10 reps/set, 3 sets; 6 types of functional training, 10 min, 10 reps/set, 3 sets. | 4 | 210 | 840 | Half | No |
| 16 | Gorzelitz2022 [20] | NCT03722030 | EC | Survivor | RT | 10 |  | 4 | 120 | 480 | No | No |
| 17 | Dieli-Conwright2022 [21] | NCT02110641 | BC | Survivor | AE | 24 | 150 min/week MI AE, 10000 steps/day. | 6 | 150 | 900 | No | Calorie restriction |
| 18 | Papadopoulos2021 [22] | NCT04266262 | PCa | Before | AE | 8 | HIIT, 25 min. 3 min warm-up; 10×1 min HIIT, ≥85% HRR; 2 min cool-down. | 10 | 50 | 500 | Yes | No |
|  |  |  |  |  | RT | 8 | 6 RT,65-85% 1RM, 15 reps/1set, 2-3 sets. | 4 | 80 | 380 | Yes | No |
| 19 | Monazzami2021 [23] | IRCT20181024041445N1 | BC | Survivor | AE+RT | 8 | AE+RT: 90 min/time. RT: 40 min, borg 12-14. AE: 40 min, 70% HRR. | 5 | 270 | 1350 | No | No |
| 20 | Moghadam2021 [24] | IRCT20190731044398N2 | BC | Survivor | AE | 12 | HIIT, 40 min. 5 min warm-up, 50% VO2peak; 8×0.5 min HIIT, ＞90% HRmax, 2 min recovery; 5 min cool-down, 50% VO2peak. | 10 | 120 | 1200 | Yes | No |
|  |  |  |  |  | AE | 12 | AE, 40 min. 5 min warm-up, 50% VO2peak; 8×0.5 min MIIT, 55-65% VO2peak, 2 min recovery; 5 min cool-down, 50% VO2peak. | 7 | 120 | 840 | Yes | No |
| 21 | Hiensch2021 [25] | NCT02522260 | BC | During | AE+RT | 16 | RT: 80% 1RM, 8-12 reps/set,2-3 sets. HIIT: 3×3 min, 6-18 RPE,1 min recovery. | 10 | 120 | 1200 | Yes | No |
|  |  |  |  |  | AE | 16 | AE: 20 min, 13-15 RPE. HITT: 3×3min, 16-18 RPE, 1 min recovery. | 10 | 64 | 640 | Yes | No |
| 22 | Febvey-Combes2021 [26] | NCT01331772 | BC | During | AE | 24 | MI exercise, 45-60 min/times. | 7 | 180 | 1260 | Yes | No |
| 23 | Bade2021 [27] | NCT03352245 | NSCLC | During | PA | 12 | 10000 steps/day | 3 | 210 | 630 | No | No |
| 24 | Uth2020 [28] |  | BC | Survivor | AE | 52 | 60 min/time, 70-90% HRR | 8.5 | 120 | 1020 | Yes | No |
| 25 | Parent-Roberge2020 [29] |  | Mixed | During | AE+RT | 12 | AE: 40 min,70-75% HRR. RT: 10-15 reps/set,2-3 sets. | 6 | 264 | 1584 | Half | No |
| 26 | Ndjavera2020 [30] | NCT03776045 | PCa | During | AE+RT | 12 | AE+RT: 60 min/time. AE: 5 min warm-up, 6×5 min MICT, 55-85% HRR; 6 RT, 20 min, RPE 11-15, 10 reps/set, 2-4 sets. | 7.5 | 210 | 1575 | Half | No |
| 27 | Meyerhardt2020, Brown2020 [31, 32] | NCT01340300 | Mixed | Survivor | AE | 12 | 5 min warm-up; 30-60 min moderate-intensity (MI) exercise, 65-80% HRR; 5 min cool-down; 10 min static stretching. | 7 | 220 | 1540 | Half | No |
| 28 | Kim2020 [33] |  | BC | Survivor | RT | 12 |  | 4 | 48 | 192 | Half | No |
| 29 | Hojan2020 [34] |  | BC | During | AE+RT | 9 | AE+RT, 90 min/time. 2 min warm-up; 45 min AE, 80% HRR; 45 min RT, 8-10 reps/set,2-3 sets; 3 min cool-down. | 8 | 450 | 3600 | Yes | No |
| 30 | Demark-Wahnefried2020 [35] |  | BC | Before | AE | 4 | AE: 30 min | 4 | 210 | 840 | Yes | Calorie restriction |
| 31 | Chang2020 [36] | NCT02895178 | BC | Survivor | AE+RT | 12 | AE+RT: 60 min/time. 10 min warm-up; AE, RPE 13-15; 8 RT, RPE 11-15, 12-16 reps/set, 3 sets; 10 min cool-down. | 6 | 180 | 1080 | Yes | No |
| 32 | Shim2019 [37] | KUGH16034-001 | CRC | During | AE+RT | 24 | AE+RT, 50 min/time.  10 min warm-up; 12 AE and RT, 1 min/sets, 2 sets; 10 min cool-down. | 4.5 | 150 | 675 | No | No |
| 33 | Ligibel2019 [38] | NCT01516190 | BC | Before | AE+RT | 4 | AE+RT, 90 min/time.  5 min warm-up; 45 min AE;  6 RT, 20 min; 10 min cool-down. | 4.5 | 270 | 1215 | Half | No |
| 34 | Kleckner2019 [39] | NCT00924651 | Mixed | During | AE+RT | 6 | AE+RT, 60 min/time.  AE, 60-85% HRR;  10 RT, 3-5 RPE, 15 reps/set, 4 sets. | 7.5 | 420 | 3150 | Yes | No |
| 35 | Christensen2019 [40] | NCT02403024 | CRC | Survivor | AE | 12 | 3 min fast walking and 3 min slow walking cycles | 4 | 150 | 600 | No | No |
| 36 | Alizadeh2019 [41] | IRCT201202289171N1 | BC | During | HIIT | 12 | HIIT, 38 min. 5 min warm-up; 4×4 min HIIT, 95% HRR; 3×4 min recovery, 50-70% HRR; 5 min cool-down. | 10 | 114 | 1140 | Yes | No |
| 37 | Winters-Stone2018 [42] | NCT00659906 NCT00591747 NCT00665080 | BC | Survivor | RT | 52 | RT, 45 min. 80% 1RM, 8-12 reps/set, 2-3 sets. | 4 | 135 | 540 | Half | No |
| 38 | Sturgeon2018 [43] |  | BC | Survivor | AE+RT | 12 |  | 4.5 | 160 | 720 | No | No |
| 39 | Paulo2018 [44] | NCT02804308 | BC | During | AE+RT | 36 | AE+RT, 100 min/time.  5 min warm-up; 30 min AE, 75-80% HRR;  6 RT, 75% 1RM; 10 min cool-down. | 8 | 300 | 2400 | Yes | No |
| 40 | Bruno2018 [45] |  | BC | Survivor | AE | 12 | AE, 60 min/time, 2 times/week. AE, home, 30 min/time, 5 times/week. | 4.5 | 270 | 1215 | Half | No |
| 41 | Wall2017 [46] | ACTRN12609000200280 | PCa | During | AE+RT | 24 | AE+RT, 60 min/time.  5 min warm-up; 30 min AE, 70-90% HRR;  6 RT, 6-12 RM, 6 reps/set, 4 sets; 10 min cool-down. | 9 | 210 | 1890 | Half | No |
| 42 | Lee2017 [47] | ISRCTN47234641 | CRC | Survivor | AE+RT | 12 | AE: ⩾10 000 steps/day, ＜65% HRmax. | 4 | 306 | 1224 | Half | No |
| 43 | Kim2017 [48] | NCT02895178 | BC | Survivor | AE+RT | 12 | AE+RT: 60 min/time. 10 min warm-up; AE, 20 min, RPE 13-15; 8 RT, 20 min, RPE 11-15, 12-16 reps/set, 3 sets; 10 min cool-down. | 6 | 180 | 1080 | Yes | No |
| 44 | Hojan2017 [49] | ISRCTN80765858 | PCa | During | AE+RT | 52 | AE+RT, 90 min/time. 5 min warm-up; 40-50% HRR; 45 min AE, 70-80% HRR; RT, 45 min, 70-75% 1RM, 8 reps/set, 2 sets; 5 min cool-down. | 8 | 450 | 3600 | Half | No |
| 45 | Arikawa2017[50] | NCT02940470 | BC | Survivor | AE+RT | 12 | 60-70% HRR. | 5.5 | 225 | 1237.5 | No | Calorie restriction |
| 46 | Schmidt2016 [51] | NCT01468766 | BC | During | RT | 6 | RT, 60 min/time. 8 RT, 60-80% 1RM, 8-12 reps/set, 3 sets. | 4 | 120 | 480 | Yes | No |
| 47 | Lahart2016 [52] | NCT02408107 | BC | Survivor | PA | 24 | 30 min MVP | 4.5 | 210 | 945 | Half | No |
| 48 | Hvid2016 [53] |  | PCa | Survivor | AE | 104 | 45 min/time, 65% VO2max | 7 | 135 | 945 | No | No |
|  |  |  |  |  | AE | 24 | 45 min/time, 65% VO2max | 7 | 135 | 945 | No | No |
| 49 | Hojan2016 [54] |  | PCa | During | AE+RT | 8 | AE+RT, 55 min/time. 5 min warm-up; 30 min AE, 65-70% HRR; 5 RT, 15 min, 70-75% 1RM, 8 reps/set, 2 sets; 5 min cool-down. | 7.5 | 275 | 2062.5 | Yes | No |
| 50 | Harrigan2016 [55] |  | BC | Survivor | PA | 24 | 10000 steps/day | 3 | 210 | 630 | No | Calorie restriction |
| 51 | Hagstrom2016 [56] | ANZCTR#: 12612000346875 | BC | During | RT | 16 | 60 min/time, 80% 1RM, 8-10 reps/set,3 sets. | 4 | 180 | 720 | Yes | No |
| 52 | Dethlefsen2016 [57] | NCT00717717 | BC | During | AE+RT | 24 | AE+RT, 90 min/time.  5 min warm-up; 12×0.5 min HITT, 80-90% HRR, 0.5 min recovery;  RT, 70-90% 1RM, 8-10 reps/set, 3 sets; 5 min cool-down. | 10 | 270 | 2700 | Half | No |
| 53 | Swisher2015 [58] | NCT01498536 | BC | Survivor | AE | 12 | 30 min/time, 60-75% HRR. | 6.5 | 150 | 975 | Half | Calorie restriction |
| 54 | Glass2015 [59] |  | Mixed | During | AE | 12 | 45 min/time, 55-100% VO2peak. | 8 | 135 | 1080 | Yes | No |
| 55 | Cormie2015 [60] | ACTRN12610000691044 | PCa | During | AE+RT | 12 | AE+RT, 60 min/time.  warm-up; 30 min AE, 70-85% HRR;  8 RT, 60-85% 1RM, 6-12 reps/set, 4 sets; cool-down. | 8 | 270 | 2160 | Yes | No |
| 56 | Saxton2014, Scott2013 [61, 62] | ISRCTN08045231 | BC | During | AE+RT | 24 | AE+RT, 45 min/time.  30 min AE, 60-85% HRR.  RT, 10-15 min. | 8 | 135 | 1080 | Yes | Calorie restriction |
| 57 | Rogers2014 [63] |  | BC | During | AE+RT | 12 | AE: 40 min/time, 48-52% HRR. RT: 15 reps/set,2 sets. | 3.5 | 256 | 896 | Yes | No |
| 58 | Giallauria2014 [64] |  | BC | Survivor | AE | 52 | 40 min/time. 5 min warm-up; 30 min AE, 75% HRR;  5 min cool-down. | 6.5 | 120 | 780 | Yes | No |
| 59 | Christensen2014_B,Christensen2014_J [65, 66] | ISRCTN32132990 | GCC | During | RT | 9 | 10-12 RM, 10 reps/set,4 sets. | 4 | 144 | 576 | Yes | No |
| 60 | Rogers2013 [67] | NCT00640666 | BC | Survivor | AE+RT | 12 | RT: 20 reps/set,1 set. | 4 | 246 | 984 | Half | No |
| 61 | Jones2013 [68] |  | BC | During | AE | 24 | 30 min/time, 60-80% HRR. | 8 | 150 | 1200 | Half | No |
| 62 | LeeJones2013[69] | NCT00405678 | BC | During | AE | 12 | 40 min/time, 55-100% VO2max | 8 | 120 | 960 | Yes | No |
| 63 | Guinan2013 [70] | NCT01030887 | BC | Survivor | AE | 8 | 42 min/time, 65-75% HRR. | 8 | 210 | 1680 | Half | No |
| 64 | Ergun2013 [71] |  | BC | Survivor | AE+RT | 12 | AE+RT, 45 min/time.  AE, 30 min.  RT, 15 min. | 4.5 | 135 | 607.5 | Half | No |
|  |  |  |  |  | AE | 12 | 30 min/time | 4.5 | 90 | 405 | Half | No |
| 65 | Nuri2013 [72] |  | BC | During | AE+RT | 15 | AE+RT, 120 min/time.  45 min AE, 65% HRR;  9 RT, 60 min, 14 reps/set, 3 sets. | 5.5 | 240 | 1320 | Yes | No |
| 66 | Pakiz2011 [73] |  | BC | Survivor | AE | 16 | 60 min/time | 4.5 | 420 | 1890 | No | Calorie restriction |
| 67 | Gómez2011 [74] |  | BC | Survivor | AE+RT | 8 | AE+RT, 90 min/time.  5 min warm-up; 30 min AE, 80% HRR;  RT, 12-15 RM, 8-10 reps/set, 2 sets; 5 min cool-down. | 9 | 270 | 2430 | Yes | No |
| 68 | Sprod2010 [75] |  | Mixed | During | AE+RT | 4 | RT: 3-5 RPE, 15 reps/set, 4 sets. AE: 10000 steps/day. | 3.5 | 546 | 1911 | No | No |
| 69 | Galvão2010 [76] | ACTRN12607000263493 | PCa | During | AE+RT | 12 | warm-up; 20 min AE, 65-80% HRR;  6 RM, 4 sets; cool-down. | 6 | 136 | 816 | Yes | No |
| 70 | Ligibel2009 [77] |  | BC | Survivor | AE+RT | 16 | 8 RT, 50 min/time. | 4.5 | 190 | 855 | Half | No |
| 71 | Irwin2009 [78] |  | BC | Survivor | AE | 24 | 30 min/time, 60-80% HRR. | 7.5 | 150 | 1125 | Half | No |
| 72 | Schmitz2005 [79] |  | BC | Survivor | RT | 24 | 9 RT, 60 min/time, 10-12 RM, 8-10 reps/set, 3 sets. | 4 | 120 | 480 | Half | No |
| 73 | Hutnick2005 [80] |  | BC | Survivor | AE+RT | 12/24 | AE+RT, 90 min/time.  20 min AE, 60-75% HRR; RT, 8-12 reps/set, 3 sets. | 6 | 270 | 1620 | Half | No |
| 74 | Fairey2005, 2003 [81-83] |  | BC | Survivor | AE | 15 | 35 min/time, 70-75% VO2max | 8 | 105 | 840 | Yes | No |

Table S2. Outcome of included studies

| No. | Study | Glucose–Insulin Group | Lipid Group | Inflammatory Group | Outcome reported but not included |
| --- | --- | --- | --- | --- | --- |
| 1 | Wright2024 | Glucose, Insulin, IGF-1, IGFBP-3, Adiponectin, HOMA |  |  | C-peptide, IGF-1:IGFBP-3 ratio |
| 2 | SenthilKumar2024 | Glucose, Insulin, Adiponectin, Leptin, HbA1c |  | CRP, IL-6, TNF-α, IL-1ra, IFN-γ, IL-1β, IL-8, IL-10, IL-12 | IL-2, GMCSF, IL-1α, Leptin: Adiponectin ratio |
| 3 | Kang2024 | Glucose, Insulin, IGF-1, IGFBP-1, IGFBP-3, Adiponectin, Leptin, HbA1c | Triglycerides, Total cholesterol, HDL-C, LDL-C |  | Non-HDL-C |
| 4 | Gnagnarella2023 | Glucose, Insulin, HOMA | Triglycerides, Total cholesterol, HDL-C, LDL-C | Hs-CRP | Beta estradiol, LDL/HDL ratio, TOT/HDL ratio |
| 5 | Sturgeon2023, Lin2023 | Adiponectin, Leptin |  | CRP | ICAM-1, VCAM-1, A:L ratio |
| 6 | Martins2023 |  |  | IL-6, TNF-α, IFN-γ, IL-1β, IL-10 | TGF-b1 |
| 7 | Isanejad2023 | Glucose, Insulin, Adiponectin, Leptin, HOMA | Total cholesterol, HDL-C, LDL-C | IL-6, TNF-α, IL-10 | SOCS3, Estradiol, TNF-α/IL-10 |
| 8 | Falz2023,Darmochwal2023 | Insulin, Adiponectin, Leptin, HbA1c | Triglycerides | CRP |  |
| 9 | Demark-Wahnefried2023 | Glucose, Adiponectin, Leptin | Total cholesterol, HDL-C | CRP |  |
| 10 | Cartmel2023 | Insulin, IGF-1, Adiponectin, Leptin |  | CRP, IL-6, TNF-α, VEGF |  |
| 11 | Brown2023, Brown2018_E, Brown2018_C | Glucose, Insulin, IGF-1, IGFBP-3, HOMA |  | Hs-CRP, IL-6 | sTNF-αR2, sICAM-1, sVCAM-1, Fructosamine, C-peptide |
| 12 | Bojesen2023 |  |  | CRP |  |
| 13 | Moon2022 |  |  | IL-6, TNF-α, IFN-γ, IL-12, VEGF | Eotaxin, IL-5, IL-1α |
| 14 | Lee2022 |  |  | Hs-CRP | NKCA |
| 15 | Hwang2022 | Glucose, Insulin, Triglycerides | Total cholesterol, HDL-C | Hs-CRP |  |
| 16 | Gorzelitz2022 | HbA1c |  | CRP |  |
| 17 | Dieli-Conwright2022 | Insulin, IGF-1, Adiponectin, Leptin |  | CRP, IL-6, TNF-α |  |
| 18 | Papadopoulos2021 | IGF-1, IGFBP-3 |  | IL-6, TNF-α, IFN-γ, IL-10 |  |
| 19 | Monazzami2021 |  |  | TNF-α, IL-12 | TNF-α/IL-12 |
| 20 | Moghadam2021 | Adiponectin, Leptin |  | IL-6, TNF-α, IL-8, IL-10 |  |
| 21 | Hiensch2021 |  |  | IL-6, TNF-α, IFN-γ, IL-1β, IL-8, IL-10, IL-12, VEGF | LAP TGF-beta-1, IL-18, IL-4, IL-7, CSF-1, CD27, CD40_L, CD40, CD70, FasL, TNFRSF-4, TNFRSF9, TNFRSF-12, TNFRSF-14 |
| 22 | Febvey-Combes2021 | Insulin, IGF-1, Adiponectin, Leptin |  | IL-6, TNF-α |  |
| 23 | Bade2021 | Insulin, Leptin |  | CRP | Soluble PD-1, Soluble PD-L1 |
| 24 | Uth2020 |  | Triglycerides, Total cholesterol, HDL-C, LDL-C | CRP |  |
| 25 | Parent-Roberge2020 | Glucose, Insulin, Adiponectin, Leptin | Triglycerides, Total cholesterol, HDL-C, LDL-C | CRP, IL-6, IL-1ra, IL-1β, IL-10 | LAR: Leptin to adiponectin ratio, IL-6/IL-10, IL-6/IL-1ra, IL-1β/IL-1ra, IL-15, Albuminemia, Prealbuminemia, KYN/TRP ratio. |
| 26 | Ndjavera2020 | Glucose, Insulin | Triglycerides, Total cholesterol, HDL-C, LDL-C |  |  |
| 27 | Meyerhardt2020, Brown2020 | Glucose, Insulin, IGF-1, IGFBP-3, Leptin, HOMA |  | Hs-CRP, IL-6 | sTNF-αR2 |
| 28 | Kim2020 |  |  | IL-6, TNF-α |  |
| 29 | Hojan2020 |  |  | Hs-CRP, IL-6 | MYO—myoglobin, CK—creatine kinase, CK-MB—creatine kinase myocardial band, AST—aspartate aminotransferase, ALT—alanine aminotransferase |
| 30 | Demark-Wahnefried2020 | Insulin, Leptin |  | IL-6, TNF-α, VEGF | Estradiol, Estrone, Testosterone, SHBG, Free fatty acids, Fibroblast growth factor-β, Ki-67, PCNA, I-R cytoplasm, I-R membrane, I-R nuclear, TNFα cytoplasm, TNFα membrane, TNFα nuclear |
| 31 | Chang2020 | Glucose, Insulin, HOMA | Triglycerides, Total cholesterol, HDL-C, LDL-C |  |  |
| 32 | Shim2019 | Adiponectin, Leptin |  |  |  |
| 33 | Ligibel2019 | Insulin, IGF-1, Adiponectin, Leptin |  | CRP, IL-6 |  |
| 34 | Kleckner2019 |  |  | IL-6, IFN-γ, IL-1β, IL-8, IL-10 | sTNFR1a |
| 35 | Christensen2019 | Leptin | Triglycerides, Total cholesterol, HDL-C, LDL-C | IL-6, TNF-α |  |
| 36 | Alizadeh2019 |  |  | IL-6, TNF-α, IFN-γ, IL-1β, IL-8, IL-10 | TNF-α/IL-10, IL-6/IL-10, IL4 |
| 37 | Winters-Stone2018 | Insulin, IGF-1, IGFBP-1, IGFBP-3, Adiponectin, Leptin |  | CRP, IL-6, TNF-α, IL-1β | SAA |
| 38 | Sturgeon2018 | Insulin |  | IL-6, TNF-α, IL-1β, IL-8 |  |
| 39 | Paulo2018 | Glucose | Triglycerides, Total cholesterol, HDL-C, LDL-C | CRP | Non-HDL-C |
| 40 | Bruno2018 | Glucose, Insulin, IGF-1, HOMA | Triglycerides, Total cholesterol, HDL-C, LDL-C |  |  |
| 41 | Wall2017 | Insulin, HbA1c | Triglycerides, Total cholesterol, HDL-C, LDL-C | CRP | Testosterone, PSA |
| 42 | Lee2017 | Glucose, Insulin, Adiponectin, HOMA | Triglycerides, Total cholesterol, HDL-C | Hs-CRP, TNF-α |  |
| 43 | Kim2017 | Insulin, Adiponectin, Leptin |  | Hs-CRP | Leptin: Adiponectin ratio |
| 44 | Hojan2017 |  | Triglycerides, Total cholesterol, HDL-C, LDL-C | IL-6, TNF-α, IL-1β | ALT, AST |
| 45 | Arikawa2017 | Glucose, Insulin, IGF-1, IGFBP-3, Adiponectin, Leptin, HOMA |  | CRP, IL-6 | QUICKI quantitative insulin sensitivity check index, F2-isoprostanes, 6-Sulfatoxymelatonin |
| 46 | Schmidt2016 |  |  | IL-6, IL-1ra | IL-6/IL-1RA |
| 47 | Lahart2016 | Insulin, HOMA | Triglycerides, Total cholesterol, HDL-C, LDL-C |  | TC/HDL-C ratio |
| 48 | Hvid2016_24m | Glucose, Insulin, IGF-1, IGFBP-1, Adiponectin, Leptin, HOMA | Triglycerides, Total cholesterol, HDL-C, LDL-C | IL-6, TNF-α | Testosterone, Matsuda index |
| 48 | Hvid2016_6m | Glucose, Insulin, HOMA | Triglycerides, Total cholesterol, HDL-C, LDL-C |  |  |
| 49 | Hojan2016 |  |  | IL-6, TNF-α, IL-1β | Hemoglobin, Red blood cells, White blood cells, Lymphocytes, Platelets, PSA |
| 50 | Harrigan2016 | Glucose, Insulin, Adiponectin, Leptin |  | CRP, IL-6, TNF-α |  |
| 51 | Hagstrom2016 |  |  | CRP, IL-6, TNF-α, IL-10 |  |
| 52 | Dethlefsen2016 | Glucose, Insulin, Leptin |  | IL-6, TNF-α, IL-8, IL-10 | LDL/HDL ratio |
| 53 | Swisher2015 | Insulin, Adiponectin, Leptin |  | CRP, IL-6, TNF-α |  |
| 54 | Glass2015 |  |  | TNF-α, VEGF | IL-4, Hepatocyte growth factor (HGF), MIP-1 β/CCL4, Cytotoxic T lymphocytes, B lymphocytes, Natural killer (NK) cells, Monocytes |
| 55 | Cormie2015 | Glucose, Insulin | Triglycerides, Total cholesterol, HDL-C, LDL-C | CRP | Total cholesterol: HDL-cholesterol ratio, Glycated haemoglobin, Vitamin D, Alkaline phosphatase, P1NP, N-telopeptide, N-telopeptide/creatinine ratio, Testosterone, PSA |
| 56 | Saxton2014;Scott2013 | IGF-1, IGFBP-1, IGFBP-3, Leptin, HOMA | Total cholesterol, HDL-C | Hs-CRP, IL-6, TNF-α | Estradiol, Estrone Testosterone, SHBG, Leukocyte counts |
| 57 | Rogers2014 |  |  | IL-6, TNF-α, IL-8, IL-10 | IL-6: IL-10, IL-8: IL-10, TNF-alpha: IL-10 |
| 58 | Giallauria2014 |  | Triglycerides, Total cholesterol, HDL-C, LDL-C | Hs-CRP, IL-6 |  |
| 59 | Christensen2014_B, Christensen2014_J | Glucose, Insulin | Triglycerides, Total cholesterol, HDL-C, LDL-C | IL-6, TNF-α, IFN-γ, IL-8, IL-10 |  |
| 60 | Rogers2013 | Adiponectin, Leptin |  | IL-6, TNF-α, IL-8, IL-10 | IL-6: IL-10 ratio, IL-8: IL-10 , TNF-α: IL-10 ratio |
| 61 | Jones2013 |  |  | CRP, IL-6, TNF-α |  |
| 62 | LeeJones2013 |  |  | IL-1β, VEGF |  |
| 63 | Guinan2013 | Glucose, Insulin, HOMA, HbA1c | Triglycerides, Total cholesterol, HDL-C, LDL-C |  | TC: HDL-C ratio |
| 64 | Ergun2013 |  |  | IL-6, TNF-α, IL-8, VEGF |  |
| 65 | Nuri2013 | Glucose, Insulin, HOMA | Triglycerides, HDL-C |  |  |
| 66 | Pakiz2011 |  |  | IL-6, TNF-α, IL-8, VEGF |  |
| 67 | Gómez2011 |  |  | IL-6, TNF-α, IL-1ra, IFN-γ, IL-1β, IL-8, IL-10, VEGF | IL-2, IL4, IL-1α, VCAM-1, IL10 / TNF α ratio, beta-NGF, CTACK, eotaxin, FGF basic, G-CSF, gmCSFα, HGF, ICAM1, IFNα2, IFNγ, IL1α, IL1ß, IL1ra, IL2, IL2ra, IL3, IL4, IL6, IL7, IL8, IL9, IL10, IL12, IL13, IL15, IL16, IL17, IL18, IP10, LIF, MCS-F, MIP1α, MIP1β, MIF, MCP1, MCP3, MIG, PDGF bb, SCF, SCGFβ, SDF1α, TRAIL, TNFα, TNFβ, and VEGF |
| 68 | Sprod2010 |  |  | IL-6, TNF-α | sTNF-R |
| 69 | Galvão2010 | Glucose, Insulin | Triglycerides, Total cholesterol, HDL-C, LDL-C | CRP | Testosterone, PSA |
| 70 | Ligibel2009 | Insulin, Adiponectin, Leptin |  |  | HMWA |
| 71 | Irwin2009 | Insulin, IGF-1, IGFBP-3 |  |  |  |
| 72 | Schmitz2005 | Glucose, Insulin, IGF-1, IGFBP-1, IGFBP-3, HOMA |  |  | IGFBP-2 |
| 73 | Hutnick2005 |  |  | IL-6, IFN-γ | IFN-γ/IL-6 |
| 74 | Fairey2005,2003 | Glucose, Insulin, IGF-1, IGFBP-1, IGFBP-3, HOMA | Triglycerides, Total cholesterol, HDL-C, LDL-C | CRP | IGF-I: IGFBP-3 molar ratio, Total cholesterol: HDL-C ratio, IGF-II |

References

1. Wright JL, Schenk JM, Gulati R, Beatty SJ, VanDoren M, Lin DW, et al. The Prostate Cancer Active Lifestyle Study (PALS): A randomized controlled trial of diet and exercise in overweight and obese men on active surveillance. Cancer Name. 2024; <https://doi.org/10.1002/cncr.35241>

2. SenthilKumar G, Schottstaedt AM, Peterson LL, Pedersen LN, Chitambar CR, Vistocky A, et al. Stay on Track: a Pilot Randomized Control Trial on the Feasibility of a Diet and Exercise Intervention in Patients with Breast Cancer Receiving Radiotherapy. Cancer research communications Name. 2024; <https://doi.org/10.1158/2767-9764.CRC-23-0148>

3. Kang D-W, Field CJ, Patel D, Fairey AS, Boule NG, Dieli-Conwright CM, et al. Effects of high-intensity interval training on cardiometabolic biomarkers in patients with prostate cancer undergoing active surveillance: a randomized controlled trial. Prostate Cancer and Prostatic Diseases Name. 2024; <https://doi.org/10.1038/s41391-024-00867-3>

4. Gnagnarella P, Dragà D, Raja S, Baggi F, Simoncini MC, Sabbatini A, et al. Physical activity and/or dietary intervention in overweight or obese breast cancer survivors: results of the InForma randomized trial. Journal of cancer survivorship : research and practice Name. 2024; <https://doi.org/10.1007/s11764-023-01415-z>

5. Sturgeon KM, Brown JC, Sears DD, Sarwer DB, Schmitz KH. WISER Survivor Trial: Combined Effect of Exercise and Weight Loss Interventions on Inflammation in Breast Cancer Survivors. Med Sci Sports Exerc Name. 2023; <https://doi.org/10.1249/mss.0000000000003050>

6. Lin D, Sturgeon KMM, Gordon BRR, Brown JCC, Sears DDD, Sarwer DBB, et al. WISER Survivor Trial: Combined Effect of Exercise and Weight Loss Interventions on Adiponectin and Leptin Levels in Breast Cancer Survivors with Overweight or Obesity. Nutrients Name. 2023; <https://doi.org/10.3390/nu15153453>

7. Martins FM, Santagnello SB, de Oliveira Junior GN, de Sousa JFR, Michelin MA, Nomelini RS, et al. Lower-Body Resistance Training Reduces Interleukin-1β and Transforming Growth Factor-β1 Levels and Fatigue and Increases Physical Performance in Breast Cancer Survivors. Journal of strength and conditioning research Name. 2023; <https://doi.org/10.1519/JSC.0000000000004270>

8. Isanejad A, Nazari S, Gharib B, Motlagh AG. Comparison of the effects of high-intensity interval and moderate-intensity continuous training on inflammatory markers, cardiorespiratory fitness, and quality of life in breast cancer patients. J Sport Health Sci Name. 2023; <https://doi.org/10.1016/j.jshs.2023.07.001>

9. Falz R, Bischoff C, Thieme R, Tegtbur U, Hillemanns P, Stolzenburg JU, et al. Effect of home-based online training and activity feedback on oxygen uptake in patients after surgical cancer therapy: a randomized controlled trial. BMC Med Name. 2023; <https://doi.org/10.1186/s12916-023-03010-6>

10. Darmochwal S, Bischoff C, Thieme R, Gockel I, Tegtbur U, Hillemanns P, et al. Impact of home-based training and nutritional behavior on body composition and metabolic markers in cancer patients: data from the CRBP-TS study. Frontiers in Nutrition Name. 2023; <https://doi.org/10.3389/fnut.2023.1152218>

11. Demark-Wahnefried W, Oster RA, Crane TE, Rogers LQ, Cole WW, Kaur H, et al. Results of DUET: A Web-Based Weight Loss Randomized Controlled Feasibility Trial among Cancer Survivors and Their Chosen Partners. Cancers Name. 2023; <https://doi.org/10.3390/cancers15051577>

12. Cartmel B, Li FY, Zhou Y, Gottlieb L, Lu L, Mszar R, et al. Randomized trial of exercise on cancer-related blood biomarkers and survival in women with ovarian cancer. Cancer Medicine Name. 2023; <https://doi.org/10.1002/cam4.6187>

13. Brown JC, Compton SLE, Meyerhardt JA, Spielmann G, Yang S. The dose-response effect of aerobic exercise on inflammation in colon cancer survivors. Frontiers in Oncology Name. 2023; <https://doi.org/10.3389/fonc.2023.1257767>

14. Brown JC, Rickels MR, Troxel AB, Zemel BS, Damjanov N, Ky B, et al. Dose-response effects of exercise on insulin among colon cancer survivors. Endocr Relat Cancer Name. 2018; <https://doi.org/10.1530/erc-17-0377>

15. Brown JC, Troxel AB, Ky B, Damjanov N, Zemel BS, Rickels MR, et al. Dose-response Effects of Aerobic Exercise Among Colon Cancer Survivors: A Randomized Phase II Trial. Clin Colorectal Cancer Name. 2018; <https://doi.org/10.1016/j.clcc.2017.06.001>

16. Bojesen RD, Dalton SO, Skou ST, Jørgensen LB, Walker LR, Eriksen JR, et al. Preoperative multimodal prehabilitation before elective colorectal cancer surgery in patients with WHO performance status I or II: randomized clinical trial. BJS Open Name. 2023; <https://doi.org/10.1093/bjsopen/zrad134>

17. Moon C, Gallegos AM, Sheikh B, Kumar P, Liss M, Patel DI. Pilot Study on the Impact of a Home-Based Exercise Program on Inflammatory Cytokines and Quality of Life in Men with Prostate Cancer Under Active Surveillance. Cancer Control Name. 2022; <https://doi.org/10.1177/10732748221130964>

18. Lee KJ, An KO. Impact of High-Intensity Circuit Resistance Exercise on Physical Fitness, Inflammation, and Immune Cells in Female Breast Cancer Survivors: A Randomized Control Trial. Int J Environ Res Public Health Name. 2022; <https://doi.org/10.3390/ijerph19095463>

19. Hwang S-H, Kang D-W, Lee M-K, Byeon JY, Park H, Park D-H, et al. Changes in DNA methylation after 6-week exercise training in colorectal cancer survivors: A preliminary study. Asia-Pacific Journal of Clinical Oncology Name. 2022; <https://doi.org/10.1111/ajco.13482>

20. Gorzelitz JS, Stoller S, Costanzo E, Gangnon R, Koltyn K, Dietz AT, et al. Improvements in strength and agility measures of functional fitness following a telehealth-delivered home-based exercise intervention in endometrial cancer survivors. Supportive Care in Cancer Name. 2022; <https://doi.org/10.1007/s00520-021-06415-2>

21. Dieli-Conwright CM, Harrigan M, Cartmel B, Chagpar A, Bai Y, Li FY, et al. Impact of a randomized weight loss trial on breast tissue markers in breast cancer survivors. NPJ Breast Cancer Name. 2022; <https://doi.org/10.1038/s41523-022-00396-z>

22. Papadopoulos E, Gillen J, Moore D, Au D, Kurgan N, Klentrou P, et al. High-intensity interval training or resistance training versus usual care in men with prostate cancer on active surveillance: a 3-arm feasibility randomized controlled trial. Appl Physiol Nutr Metab Name. 2021; <https://doi.org/10.1139/apnm-2021-0365>

23. Monazzami A, Momenpur R, Alipour E, Yari K, Payandeh M. Effects of Eight-Week Combined Resistance and Endurance Training on Salivary Interleukin-12, Tumor Necrosis Factor, Cortisol, and Testosterone Levels in Patients with Breast Cancer. International Journal of Cancer Management Name. 2021; <https://doi.org/https://doi.org/10.5812/ijcm.109039>

24. Hooshmand Moghadam B, Golestani F, Bagheri R, Cheraghloo N, Eskandari M, Wong A, et al. The Effects of High-Intensity Interval Training vs. Moderate-Intensity Continuous Training on Inflammatory Markers, Body Composition, and Physical Fitness in Overweight/Obese Survivors of Breast Cancer: A Randomized Controlled Clinical Trial. Cancers (Basel) Name. 2021; <https://doi.org/10.3390/cancers13174386>

25. Hiensch AE, Mijwel S, Bargiela D, Wengström Y, May AM, Rundqvist H. Inflammation Mediates Exercise Effects on Fatigue in Patients with Breast Cancer. Medicine and science in sports and exercise Name. 2021; <https://doi.org/10.1249/MSS.0000000000002490>

26. Febvey-Combes O, Jobard E, Rossary A, Pialoux V, Foucaut A-M, Morelle M, et al. Effects of an Exercise and Nutritional Intervention on Circulating Biomarkers and Metabolomic Profiling During Adjuvant Treatment for Localized Breast Cancer: Results From the PASAPAS Feasibility Randomized Controlled Trial. Integrative Cancer Therapies Name. 2021; <https://doi.org/10.1177/1534735420977666>

27. Bade BC, Gan G, Li F, Lu L, Tanoue L, Silvestri GA, et al. “Randomized trial of physical activity on quality of life and lung cancer biomarkers in patients with advanced stage lung cancer: a pilot study”. BMC Cancer Name. 2021; <https://doi.org/10.1186/s12885-021-08084-0>

28. Uth J, Fristrup B, Sørensen V, Helge EW, Christensen MK, Kjærgaard JB, et al. Exercise intensity and cardiovascular health outcomes after 12 months of football fitness training in women treated for stage I-III breast cancer: Results from the football fitness After Breast Cancer (ABC) randomized controlled trial. Progress in Cardiovascular Diseases Name. 2020; <https://doi.org/10.1016/j.pcad.2020.08.002>

29. Parent-Roberge H, Fontvieille A, Marechal R, Wagner R, Fulop T, Pavic M, et al. Effects of combined exercise training on the inflammatory profile of older cancer patients treated with systemic therapy. Brain, behavior, and immunity - health Name. 2020; <https://doi.org/10.1016/j.bbih.2019.100016>

30. Ndjavera W, Orange ST, O'Doherty AF, Leicht AS, Rochester M, Mills R, et al. Exercise-induced attenuation of treatment side-effects in patients with newly diagnosed prostate cancer beginning androgen-deprivation therapy: a randomised controlled trial. BJU Int Name. 2020; <https://doi.org/10.1111/bju.14922>

31. Meyerhardt JA, Irwin ML, Jones LW, Zhang S, Campbell N, Brown JC, et al. Randomized phase II trial of exercise, metformin, or both on metabolic biomarkers in colorectal and breast cancer survivors. JNCI Cancer Spectrum Name. 2020; <https://doi.org/https://doi.org/10.1093/jncics/pkz096>

32. Brown JC, Zhang S, Ligibel JA, Irwin ML, Jones LW, Campbell N, et al. Effect of Exercise or Metformin on Biomarkers of Inflammation in Breast and Colorectal Cancer: a Randomized Trial. Cancer prevention research (Philadelphia, Pa) Name. 2020; <https://doi.org/10.1158/1940-6207.CAPR-20-0188>

33. Kim SH, Song YK, Han J, Ko YH, Lee H, Kang MJ, et al. Pro-inflammatory Cytokine Levels and Cancer-related Fatigue in Breast Cancer Survivors: Effects of an Exercise Adherence Program. J Breast Cancer Name. 2020; <https://doi.org/10.4048/jbc.2020.23.e22>

34. Hojan K, Procyk D, Horyńska-Kęstowicz D, Leporowska E, Litwiniuk M. The preventive role of regular physical training in ventricular remodeling, serum cardiac markers, and exercise performance changes in breast cancer in women undergoing trastuzumab therapy—an reh-her study. Journal of Clinical Medicine Name. 2020; <https://doi.org/10.3390/jcm9051379>

35. Demark-Wahnefried W, Rogers LQ, Gibson JT, Harada S, Frugé AD, Oster RA, et al. Randomized trial of weight loss in primary breast cancer: Impact on body composition, circulating biomarkers and tumor characteristics. International Journal of Cancer Name. 2020; <https://doi.org/10.1002/ijc.32637>

36. Chang JS, Kim TH, Kong ID. Exercise intervention lowers aberrant serum WISP-1 levels with insulin resistance in breast cancer survivors: a randomized controlled trial. Sci Rep Name. 2020; <https://doi.org/10.1038/s41598-020-67794-w>

37. Shim YJ, Kim HJ, Oh SC, Lee SI, Choi SW. Exercise during adjuvant treatment for colorectal cancer: treatment completion, treatment-related toxicities, body composition, and serum level of adipokines. Cancer Manag Res Name. 2019; <https://doi.org/10.2147/cmar.S208754>

38. Ligibel JA, Dillon D, Giobbie-Hurder A, McTiernan A, Frank E, Cornwell M, et al. Impact of a Pre-Operative Exercise Intervention on Breast Cancer Proliferation and Gene Expression: Results from the Pre-Operative Health and Body (PreHAB) Study. Clin Cancer Res Name. 2019; <https://doi.org/10.1158/1078-0432.Ccr-18-3143>

39. Kleckner IR, Kamen C, Cole C, Fung C, Heckler CE, Guido JJ, et al. Effects of exercise on inflammation in patients receiving chemotherapy: a nationwide NCORP randomized clinical trial. Supportive care in cancer Name. 2019; <https://doi.org/10.1007/s00520-019-04772-7>

40. Christensen JF, Sundberg A, Osterkamp J, Thorsen-Streit S, Nielsen AB, Olsen CK, et al. Interval Walking Improves Glycemic Control and Body Composition After Cancer Treatment: A Randomized Controlled Trial. J Clin Endocrinol Metab Name. 2019; <https://doi.org/10.1210/jc.2019-00590>

41. Alizadeh AM, Isanejad A, Sadighi S, Mardani M, Kalaghchi B, Hassan ZM. High-intensity interval training can modulate the systemic inflammation and HSP70 in the breast cancer: a randomized control trial. J Cancer Res Clin Oncol Name. 2019; <https://doi.org/10.1007/s00432-019-02996-y>

42. Winters-Stone KM, Wood LJ, Stoyles S, Dieckmann NF. The effects of resistance exercise on biomarkers of breast cancer prognosis: A pooled analysis of three randomized trials. Cancer Epidemiology Biomarkers and Prevention Name. 2018; <https://doi.org/10.1158/1055-9965.EPI-17-0766>

43. Kathleen M Sturgeon 1 WF, Mariane Heroux 2. Change in Inflammatory Biomarkers and Adipose Tissue in BRCA1/2+ Breast Cancer Survivors Following a Yearlong Lifestyle Modification Program. Cancer Prevention Research Name. 2018; <https://doi.org/10.1158/1940-6207.CAPR-18-0098>

44. de Paulo TRS, Winters-Stone KM, Viezel J, Rossi FE, Simões RR, Tosello G, et al. Effects of resistance plus aerobic training on body composition and metabolic markers in older breast cancer survivors undergoing aromatase inhibitor therapy. Exp Gerontol Name. 2018; <https://doi.org/10.1016/j.exger.2018.07.022>

45. Bruno E, Roveda E, Vitale J, Montaruli A, Berrino F, Villarini A, et al. Effect of aerobic exercise intervention on markers of insulin resistance in breast cancer women. Eur J Cancer Care (Engl) Name. 2018; <https://doi.org/10.1111/ecc.12617>

46. Wall BA, GalvãO DA, Fatehee N, Taaffe DR, Spry N, Joseph D, et al. Exercise Improves V˙O2max and Body Composition in Androgen Deprivation Therapy-treated Prostate Cancer Patients. Med Sci Sports Exerc Name. 2017; <https://doi.org/10.1249/mss.0000000000001277>

47. Lee MK, Kim JY, Kim DI, Kang DW, Park JH, Ahn KY, et al. Effect of home-based exercise intervention on fasting insulin and Adipocytokines in colorectal cancer survivors: a randomized controlled trial. Metabolism: Clinical and Experimental Name. 2017; <https://doi.org/10.1016/j.metabol.2017.07.005>

48. Kim TH, Chang JS, Park KS, Park J, Kim N, Lee JI, et al. Effects of exercise training on circulating levels of Dickkpof-1 and secreted frizzledrelated protein-1 in breast cancer survivors: A pilot single-blind randomized controlled trial. PLoS ONE Name. 2017; <https://doi.org/10.1371/journal.pone.0171771>

49. Hojan K, Kwiatkowska-Borowczyk E, Leporowska E, Milecki P. Inflammation, cardiometabolic markers, and functional changes in men with prostate cancer: A randomized controlled trial of a 12-month exercise program. Polish Archives of Internal Medicine Name. 2017; <https://doi.org/10.20452/pamw.3888>

50. Arikawa AY, Kaufman BC, Raatz SK, Kurzer MS. Effects of a parallel-arm randomized controlled weight loss pilot study on biological and psychosocial parameters of overweight and obese breast cancer survivors. Pilot Feasibility Stud Name. 2018; <https://doi.org/10.1186/s40814-017-0160-9>

51. Schmidt ME, Meynköhn A, Habermann N, Wiskemann J, Oelmann J, Hof H, et al. Resistance Exercise and Inflammation in Breast Cancer Patients Undergoing Adjuvant Radiation Therapy: Mediation Analysis From a Randomized, Controlled Intervention Trial. Int J Radiat Oncol Biol Phys Name. 2016; <https://doi.org/10.1016/j.ijrobp.2015.10.058>

52. Lahart IM, Metsios GS, Nevill AM, Kitas GD, Carmichael AR. Randomised controlled trial of a home-based physical activity intervention in breast cancer survivors. BMC Cancer Name. 2016; <https://doi.org/10.1186/s12885-016-2258-5>

53. Hvid T, Lindegaard B, Winding K, Iversen P, Brasso K, Solomon TPJ, et al. Effect of a 2-year home-based endurance training intervention on physiological function and PSA doubling time in prostate cancer patients. Cancer Causes and Control Name. 2016; <https://doi.org/10.1007/s10552-015-0694-1>

54. Hojan K, Kwiatkowska-Borowczyk E, Leporowska E, Górecki M, Ozga-Majchrzak O, Milecki T, et al. Physical exercise for functional capacity, blood immune function, fatigue, and quality of life in high-risk prostate cancer patients during radiotherapy: a prospective, randomized clinical study. Eur J Phys Rehabil Med Name. 2016;

55. Harrigan M, Cartmel B, Loftfield E, Sanft T, Chagpar AB, Zhou Y, et al. Randomized trial comparing telephone versus in-person weight loss counseling on body composition and circulating biomarkers in women treated for breast cancer: The lifestyle, exercise, and nutrition (LEAN) study. Journal of Clinical Oncology Name. 2016; <https://doi.org/10.1200/JCO.2015.61.6375>

56. Hagstrom AD, Marshall PWM, Lonsdale C, Papalia S, Cheema BS, Toben C, et al. The effect of resistance training on markers of immune function and inflammation in previously sedentary women recovering from breast cancer: a randomized controlled trial. Breast Cancer Research and Treatment Name. 2016; <https://doi.org/10.1007/s10549-016-3688-0>

57. Dethlefsen C, Lillelund C, Midtgaard J, Andersen C, Pedersen BK, Christensen JF, et al. Exercise regulates breast cancer cell viability: systemic training adaptations versus acute exercise responses. Breast Cancer Res Treat Name. 2016; <https://doi.org/10.1007/s10549-016-3970-1>

58. Swisher AK, Abraham J, Bonner D, Gilleland D, Hobbs G, Kurian S, et al. Exercise and dietary advice intervention for survivors of triple-negative breast cancer: effects on body fat, physical function, quality of life, and adipokine profile. Support Care Cancer Name. 2015; <https://doi.org/10.1007/s00520-015-2667-z>

59. Glass OK, Inman BA, Broadwater G, Courneya KS, Mackey JR, Goruk S, et al. Effect of aerobic training on the host systemic milieu in patients with solid tumours: an exploratory correlative study. Br J Cancer Name. 2015; <https://doi.org/10.1038/bjc.2014.662>

60. Cormie P, Galvão DA, Spry N, Joseph D, Chee R, Taaffe DR, et al. Can supervised exercise prevent treatment toxicity in patients with prostate cancer initiating androgen-deprivation therapy: a randomised controlled trial. BJU Int Name. 2015; <https://doi.org/10.1111/bju.12646>

61. Saxton JM, Scott EJ, Daley AJ, Woodroofe M, Mutrie N, Crank H, et al. Effects of an exercise and hypocaloric healthy eating intervention on indices of psychological health status, hypothalamic-pituitary-adrenal axis regulation and immune function after early-stage breast cancer: a randomised controlled trial. Breast Cancer Res Name. 2014; <https://doi.org/10.1186/bcr3643>

62. Scott E, Daley AJ, Doll H, Woodroofe N, Coleman RE, Mutrie N, et al. Effects of an exercise and hypocaloric healthy eating program on biomarkers associated with long-term prognosis after early-stage breast cancer: a randomized controlled trial. Cancer Causes Control Name. 2013; <https://doi.org/10.1007/s10552-012-0104-x>

63. Rogers LQ, Vicari S, Trammell R, Hopkins-Price P, Fogleman A, Spenner A, et al. Biobehavioral factors mediate exercise effects on fatigue in breast cancer survivors. Med Sci Sports Exerc Name. 2014; <https://doi.org/10.1249/mss.0000000000000210>

64. Giallauria F, Gentile M, Chiodini P, Berrino F, Mattiello A, Maresca L, et al. Exercise training reduces high mobility group box-1 protein levels in women with breast cancer: findings from the DIANA-5 study. Monaldi archives for chest disease = archivio monaldi per LE malattie del torace Name. 2014; <https://doi.org/10.4081/monaldi.2014.45>

65. Christensen JF, Tolver A, Andersen JL, Rørth M, Daugaard G, Hojman P. Resistance training does not protect against increases in plasma cytokine levels among germ cell cancer patients during and after chemotherapy. J Clin Endocrinol Metab Name. 2014; <https://doi.org/10.1210/jc.2013-4495>

66. Christensen JF, Jones LW, Tolver A, Jørgensen LW, Andersen JL, Adamsen L, et al. Safety and efficacy of resistance training in germ cell cancer patients undergoing chemotherapy: a randomized controlled trial. Br J Cancer Name. 2014; <https://doi.org/10.1038/bjc.2014.273>

67. Rogers LQ, Fogleman A, Trammell R, Hopkins-Price P, Vicari S, Rao K, et al. Effects of a physical activity behavior change intervention on inflammation and related health outcomes in breast cancer survivors: pilot randomized trial. Integr Cancer Ther Name. 2013; <https://doi.org/10.1177/1534735412449687>

68. Jones SB, Thomas GA, Hesselsweet SD, Alvarez-Reeves M, Yu H, Irwin ML. Effect of exercise on markers of inflammation in breast cancer survivors: the Yale exercise and survivorship study. Cancer Prev Res (Phila) Name. 2013; <https://doi.org/10.1158/1940-6207.Capr-12-0278>

69. Jones LW, Fels DR, West M, Allen JD, Broadwater G, Barry WT, et al. Modulation of circulating angiogenic factors and tumor biology by aerobic training in breast cancer patients receiving neoadjuvant chemotherapy. Cancer prevention research (Philadelphia, Pa) Name. 2013; <https://doi.org/10.1158/1940-6207.CAPR-12-0416>

70. Guinan E, Hussey J, Broderick JM, Lithander FE, O'Donnell D, Kennedy MJ, et al. The effect of aerobic exercise on metabolic and inflammatory markers in breast cancer survivors--a pilot study. Support Care Cancer Name. 2013; <https://doi.org/10.1007/s00520-013-1743-5>

71. Ergun M, Eyigor S, Karaca B, Kisim A, Uslu R. Effects of exercise on angiogenesis and apoptosis-related molecules, quality of life, fatigue and depression in breast cancer patients. Eur J Cancer Care (Engl) Name. 2013; <https://doi.org/10.1111/ecc.12068>

72. Nuri R, Kordi MR, Moghaddasi M, Rahnama N, Damirchi A, Rahmani-Nia F, et al. Effect of combination exercise training on metabolic syndrome parameters in postmenopausal women with breast cancer. J Cancer Res Ther Name. 2012; <https://doi.org/10.4103/0973-1482.98977>

73. Pakiz B, Flatt S, Bardwell W, Rock C, Mills P. Effects of a Weight Loss Intervention on Body Mass, Fitness, and Inflammatory Biomarkers in Overweight or Obese Breast Cancer Survivors. International Journal of Behavioral Medicine Name. 2011; <https://doi.org/10.1007/s12529-010-9079-8>

74. Gómez AM, Martínez C, Fiuza-Luces C, Herrero F, Pérez M, Madero L, et al. Exercise training and cytokines in breast cancer survivors. Int J Sports Med Name. 2011; <https://doi.org/10.1055/s-0031-1271697>

75. Sprod LK, Palesh OG, Janelsins MC, Peppone LJ, Heckler CE, Jacob Adams M, et al. Exercise, sleep quality, and mediators of sleep in breast and prostate cancer patients receiving radiation therapy. Community Oncology Name. 2010; <https://doi.org/10.1016/S1548-5315(11)70427-2>

76. Galvão DA, Taaffe DR, Spry N, Joseph D, Newton RU. Combined resistance and aerobic exercise program reverses muscle loss in men undergoing androgen suppression therapy for prostate cancer without bone metastases: a randomized controlled trial. J Clin Oncol Name. 2010; <https://doi.org/10.1200/jco.2009.23.2488>

77. Ligibel JA, Giobbie-Hurder A, Olenczuk D, Campbell N, Salinardi T, Winer EP, et al. Impact of a mixed strength and endurance exercise intervention on levels of adiponectin, high molecular weight adiponectin and leptin in breast cancer survivors. Cancer Causes Control Name. 2009; <https://doi.org/10.1007/s10552-009-9358-3>

78. Irwin ML, Varma K, Alvarez-Reeves M, Cadmus L, Wiley A, Chung GG, et al. Randomized controlled trial of aerobic exercise on insulin and insulin-like growth factors in breast cancer survivors: the Yale Exercise and Survivorship study. Cancer Epidemiol Biomarkers Prev Name. 2009; <https://doi.org/10.1158/1055-9965.Epi-08-0531>

79. Schmitz KH, Ahmed RL, Hannan PJ, Yee D. Safety and efficacy of weight training in recent breast cancer survivors to alter body composition, insulin, and insulin-like growth factor axis proteins. Cancer Epidemiol Biomarkers Prev Name. 2005; <https://doi.org/10.1158/1055-9965.Epi-04-0736>

80. Hutnick NA, Williams NI, Kraemer WJ, Orsega-Smith E, Dixon RH, Bleznak AD, et al. Exercise and lymphocyte activation following chemotherapy for breast cancer. Med Sci Sports Exerc Name. 2005; <https://doi.org/10.1249/01.mss.0000175857.84936.1a>

81. Fairey AS, Courneya KS, Field CJ, Bell GJ, Jones LW, Mackey JR. Randomized controlled trial of exercise and blood immune function in postmenopausal breast cancer survivors. J Appl Physiol (1985) Name. 2005; <https://doi.org/10.1152/japplphysiol.00566.2004>

82. Fairey AS, Courneya KS, Field CJ, Bell GJ, Jones LW, Martin BS, et al. Effect of exercise training on C-reactive protein in postmenopausal breast cancer survivors: a randomized controlled trial. Brain Behav Immun Name. 2005; <https://doi.org/10.1016/j.bbi.2005.04.001>

83. Fairey AS, Courneya KS, Field CJ, Bell GJ, Jones LW, Mackey JR. Effects of exercise training on fasting insulin, insulin resistance, insulin-like growth factors, and insulin-like growth factor binding proteins in postmenopausal breast cancer survivors: a randomized controlled trial. Cancer Epidemiology Biomarkers & Prevention Name. 2003;

**Supplementary information 4. Information on Effect Sizes.**

| ID | Effect Size Name | | Unit | Number of studies | Number of effect sizes |
| --- | --- | --- | --- | --- | --- |
| A | Glucose–Insulin Group | | | | |
| A1 | Glucose |  | mg/dL, mmol/L, mM, mmol/liter | 27 | 35 |
| A2 | Insulin |  | pmol/L, pg/mL, pM, mU/L, pmol/liter, mg/dL, μU/ml, μU/mL, microunits/mL | 40 | 44 |
| A3 | IGF-1 |  | ng/mL, μg/L, | 17 | 19 |
| A4* | Adiponectin |  | μg/mL, ng/mL, pg/mL, ng/L, mg/L | 23 | 26 |
| A5 | Leptin |  | ng/L, pg/mL, μg/mL, ng/mL, μg/L, pg/L | 27 | 30 |
| A6 | HOMA index |  | % | 17 | 23 |
| B | Lipid Group | | | | |
| B1 | Triglycerides |  | mmol/L, mg/dL, mM | 24 | 29 |
| B2 | Total cholesterol |  | mmol/L, mg/dL, mM | 25 | 31 |
| B3* | HDL-C |  | mmol/L, mg/dL, mM | 24 | 32 |
| B4 | LDL-C |  | mmol/L, mg/dL, mM | 23 | 27 |
| C | Inflammatory Group | | | | |
| C1 | CRP | Hs-CRP | mg/L, mg/dL | 33 | 39 |
|  |  | CRP | mg/L, mg/dL, ng/mL, mmol/L |  |  |
| C2 | IL-6 |  | ft/mL, pg/mL, pg/L, fg/mL | 42 | 52 |
| C3 | TNF-α |  | pg/mL, mg/L, ng/L, ft/mL, pg/L, fg/mL | 35 | 40 |
| C4* | IFN-γ |  | pg/mL, mg/L | 10 | 15 |
| C5 | IL-8 |  | pg/mL, mg/L | 13 | 15 |
| C6 | IL-10 |  | fg/mL, pg/mL, mg/L | 15 | 19 |
| D | Excluded markers | | | | |
| D1 | IGFBP-1 |  | ng/mL | 6 | 6 |
| D2 | IGFBP-3 |  | ng/mL, nmol/L, μg/mL | 11 | 13 |
| D3 | HbA1c |  | % | 6 | 6 |
| D4 | IL-1ra |  | pg/mL, mg/L | 4 | 4 |
| D5 | IL-1β |  | ft/mL, pg/mL, mg/L | 12 | 13 |
| D6 | IL-12 |  | pg/mL, mg/L | 4 | 6 |
| D7 | VEGF | VEGF, VEGF-A, VEGF-C, VEGFR-2 | pg/ml | 9 | 15 |

Note: IGF-1 = Insulin-like growth factor 1; IGFBP-1 = Insulin-like growth factor binding protein 1; IGFBP-3 = Insulin-like growth factor binding protein 3; HOMA = Homeostasis model assessmen; HbA1c = Hemoglobin A1c; HDL-C = High-density lipoprotein cholesterol; LDL-C = Low-density lipoprotein cholesterol; CRP = C-reactive protein; hs-CRP = High-sensitivity C-reactive protein; IL-6 = Interleukin-6; TNF-α = Tumor necrosis factor alpha; IL-1ra = Interleukin-1 receptor antagonist; IFN-γ = Interferon gamma; IL-1β = Interleukin-1 beta; IL-8 = Interleukin-8; IL-10 = Interleukin-10; IL-12 = Interleukin-12; VEGF = Vascular endothelial growth factor. Effect sizes marked with an asterisk (*) have been converted; more negative values indicate better outcomes.

**Supplementary information 5.Overall effect size and sensitivity analyses**

Table S3. RVE model parameters without moderator variables

|  | ES | SE | t | dfs | prob | CI.L | CI.U | sig | I² |
| --- | --- | --- | --- | --- | --- | --- | --- | --- | --- |
| Glucose | -0.07 | 0.07 | -1.08 | 23.83 | 0.29 | -0.21 | 0.06 |  | 0.35 |
| Insulin | -0.24 | 0.08 | -3.26 | 35.11 | 0.00 | -0.40 | -0.09 | *** | 0.49 |
| IGF-1 | -0.04 | 0.12 | -0.33 | 14.89 | 0.75 | -0.29 | 0.21 |  | 0.51 |
| Adiponectin | 0.01 | 0.05 | 0.16 | 13.26 | 0.88 | -0.11 | 0.13 |  | 0.00 |
| Leptin | -0.16 | 0.06 | -2.55 | 21.39 | 0.02 | -0.29 | -0.03 | ** | 0.27 |
| HOMA index | -0.19 | 0.09 | -2.09 | 12.85 | 0.06 | -0.39 | 0.01 | * | 0.29 |
| Triglycerides | -0.16 | 0.06 | -2.58 | 18.23 | 0.02 | -0.29 | -0.03 | ** | 0.20 |
| Total cholesterol | -0.11 | 0.05 | -2.12 | 16.88 | 0.05 | -0.21 | 0.00 | ** | 0.00 |
| HDL-C | -0.03 | 0.05 | -0.70 | 17.43 | 0.49 | -0.14 | 0.07 |  | 0.00 |
| LDL-C | -0.05 | 0.05 | -0.91 | 14.62 | 0.38 | -0.16 | 0.06 |  | 0.00 |
| CRP | -0.19 | 0.09 | -2.12 | 29.41 | 0.04 | -0.37 | -0.01 | ** | 0.63 |
| IL-6 | -0.17 | 0.10 | -1.78 | 39.76 | 0.08 | -0.37 | 0.02 | * | 0.69 |
| TNF-α | -0.22 | 0.13 | -1.70 | 33.41 | 0.10 | -0.49 | 0.04 | * | 0.74 |
| IFN-γ | -0.08 | 0.26 | 0.32 | 8.93 | 0.76 | -0.50 | 0.37 |  | 0.85 |
| IL-8 | -0.16 | 0.15 | -1.02 | 10.48 | 0.33 | -0.49 | 0.18 |  | 0.63 |
| IL-10 | 0.03 | 0.11 | 0.25 | 12.12 | 0.81 | -0.21 | 0.27 |  | 0.36 |

Table S4. Sensitivity analysis results

| NO. | 1 | 2 | 3 | 4 | 5 | 6 | 7 | 8 | 9 |
| --- | --- | --- | --- | --- | --- | --- | --- | --- | --- |
| rho | 0.1 | 0.2 | 0.3 | 0.4 | 0.5 | 0.6 | 0.7 | 0.8 | 0.9 |
| Glucose | -0.07 | -0.07 | -0.07 | -0.07 | -0.07 | -0.07 | -0.07 | -0.07 | -0.07 |
| Insulin | -0.24 | -0.24 | -0.24 | -0.24 | -0.24 | -0.24 | -0.24 | -0.24 | -0.24 |
| IGF-1 | -0.04 | -0.04 | -0.04 | -0.04 | -0.04 | -0.04 | -0.04 | -0.04 | -0.04 |
| Adiponectin | 0.01 | 0.01 | 0.01 | 0.01 | 0.01 | 0.01 | 0.01 | 0.01 | 0.01 |
| Leptin | -0.16 | -0.16 | -0.16 | -0.16 | -0.16 | -0.16 | -0.16 | -0.16 | -0.16 |
| HOMA index | -0.19 | -0.19 | -0.19 | -0.19 | -0.19 | -0.19 | -0.19 | -0.19 | -0.19 |
| Triglycerides | -0.16 | -0.16 | -0.16 | -0.16 | -0.16 | -0.16 | -0.16 | -0.16 | -0.16 |
| Total cholesterol | -0.11 | -0.11 | -0.11 | -0.11 | -0.11 | -0.11 | -0.11 | -0.11 | -0.11 |
| HDL-C | -0.03 | -0.03 | -0.03 | -0.03 | -0.03 | -0.03 | -0.03 | -0.03 | -0.03 |
| LDL-C | -0.05 | -0.05 | -0.05 | -0.05 | -0.05 | -0.05 | -0.05 | -0.05 | -0.05 |
| CRP | -0.19 | -0.19 | -0.19 | -0.19 | -0.19 | -0.19 | -0.19 | -0.19 | -0.19 |
| IL-6 | -0.17 | -0.17 | -0.17 | -0.17 | -0.17 | -0.17 | -0.17 | -0.17 | -0.17 |
| TNF-α | -0.22 | -0.22 | -0.22 | -0.22 | -0.22 | -0.22 | -0.22 | -0.22 | -0.22 |
| IFN-γ | -0.08 | -0.08 | -0.08 | -0.08 | -0.08 | -0.08 | -0.08 | -0.08 | -0.08 |
| IL-8 | -0.16 | -0.16 | -0.16 | -0.16 | -0.16 | -0.16 | -0.16 | -0.16 | -0.16 |
| IL-10 | 0.03 | 0.03 | 0.03 | 0.03 | 0.03 | 0.03 | 0.03 | 0.03 | 0.03 |


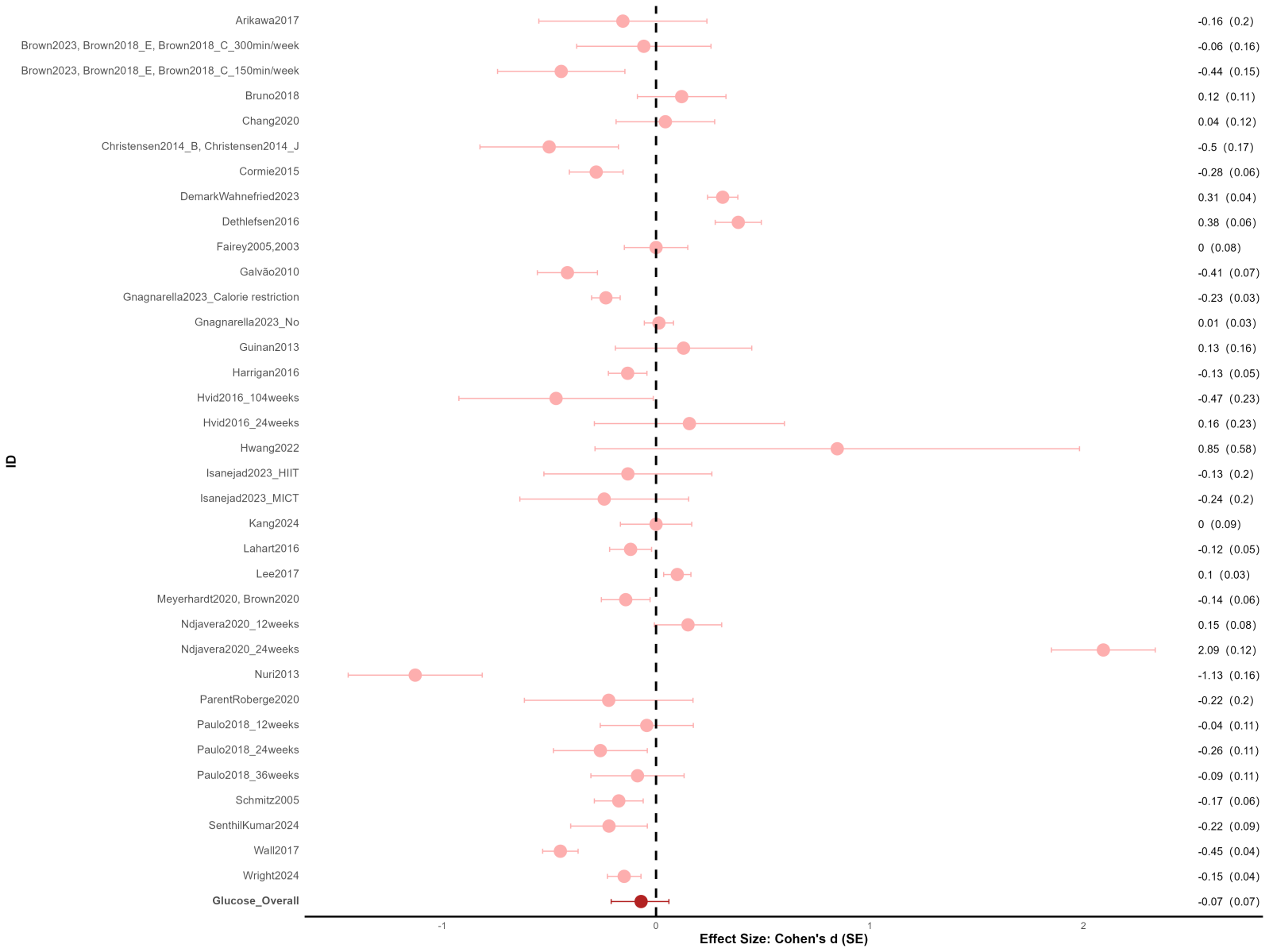


Fig. S1. Forest plot of Glucose


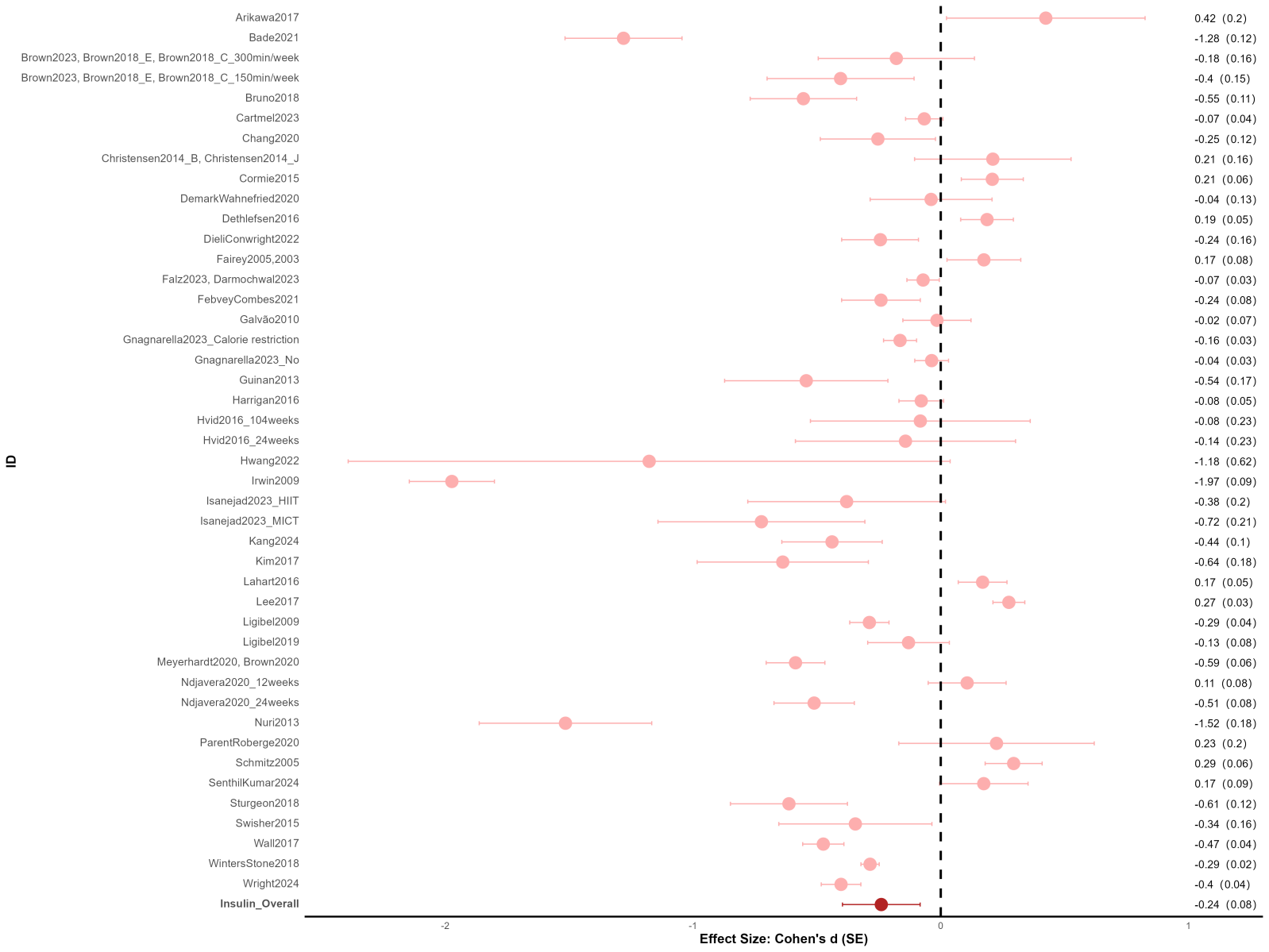


Fig. S2. Forest plot of Insulin


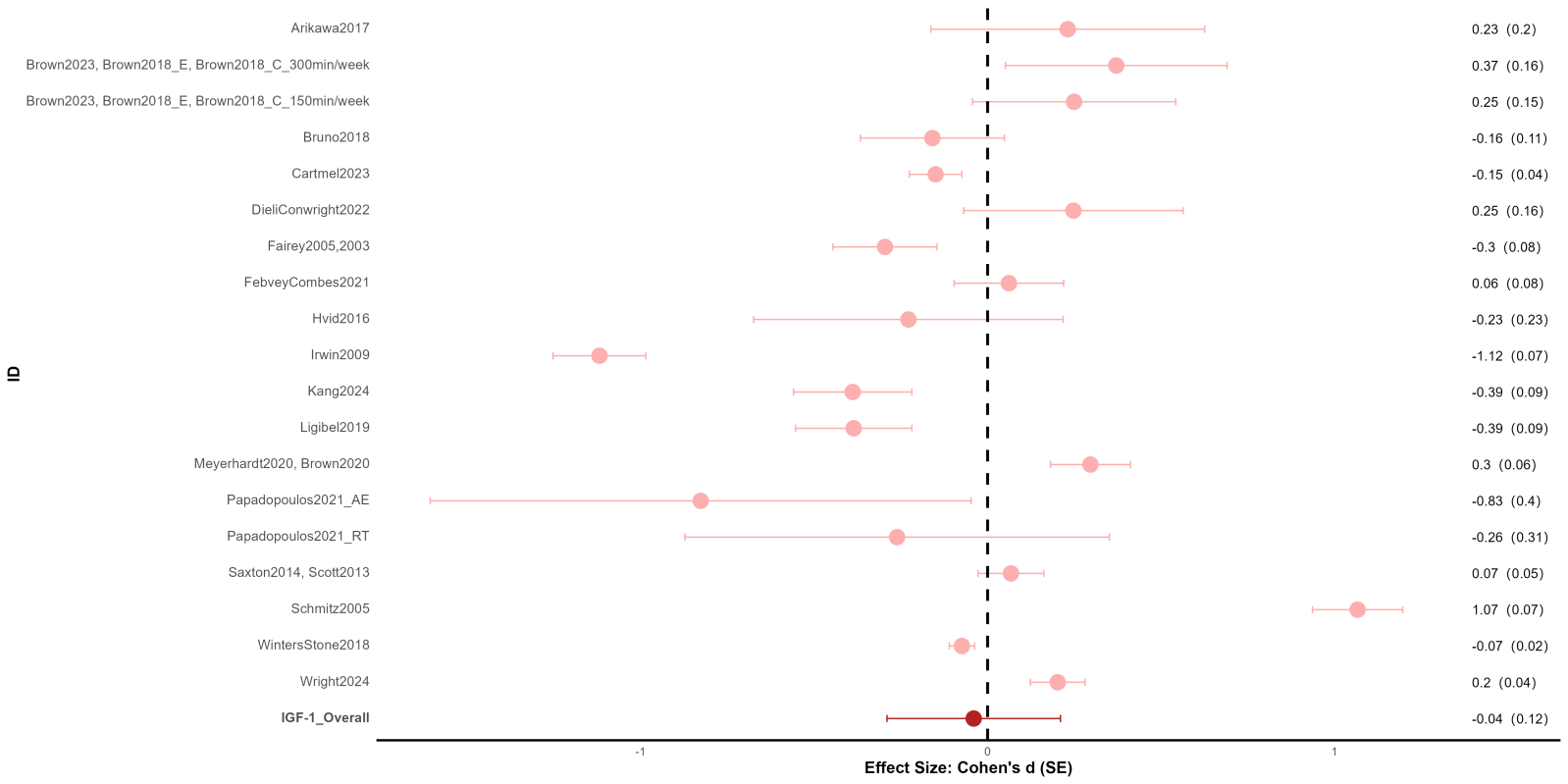


Fig. S3. Forest plot of IGF-1


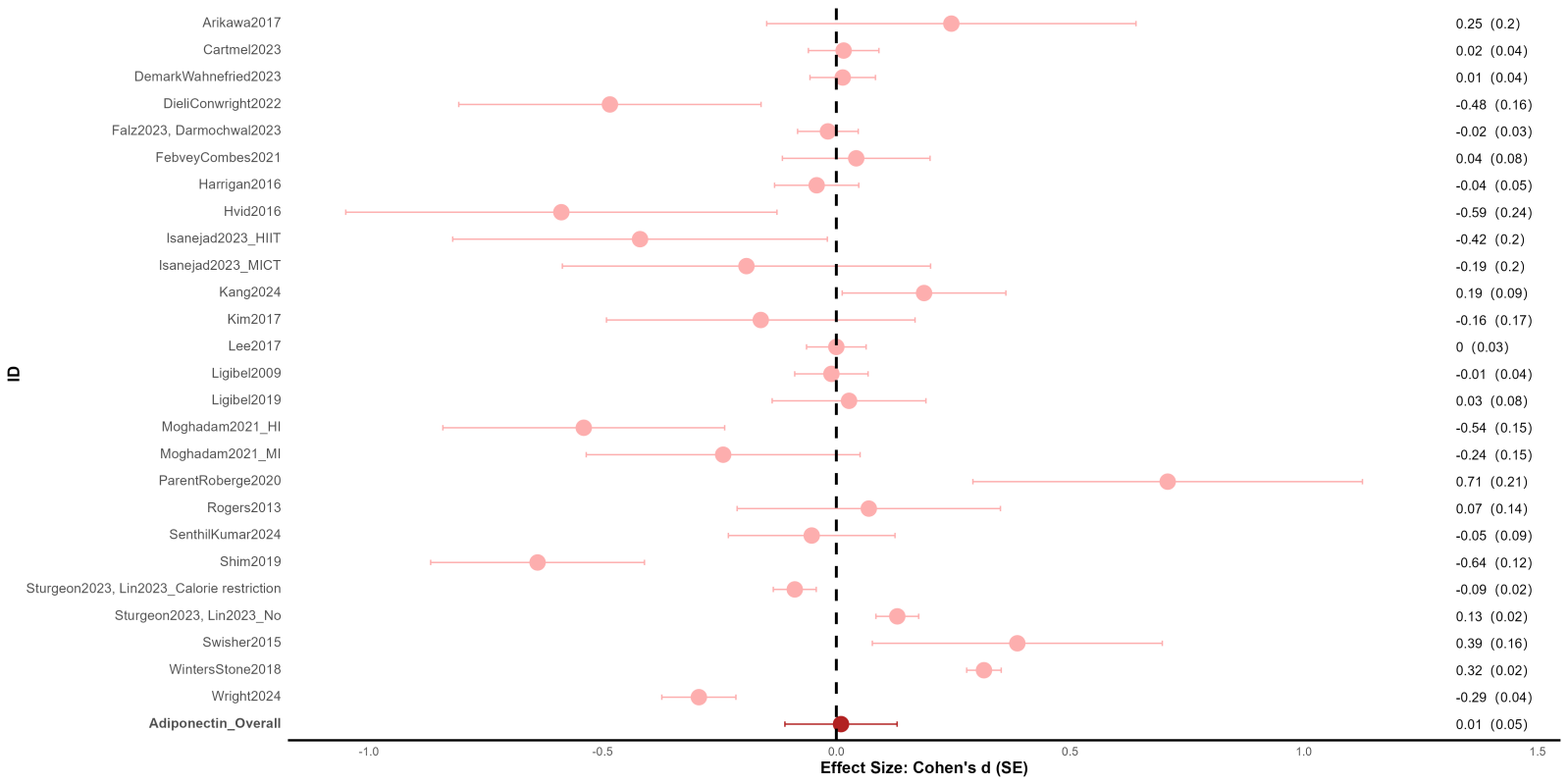


Fig. S4. Forest plot of Adiponectin


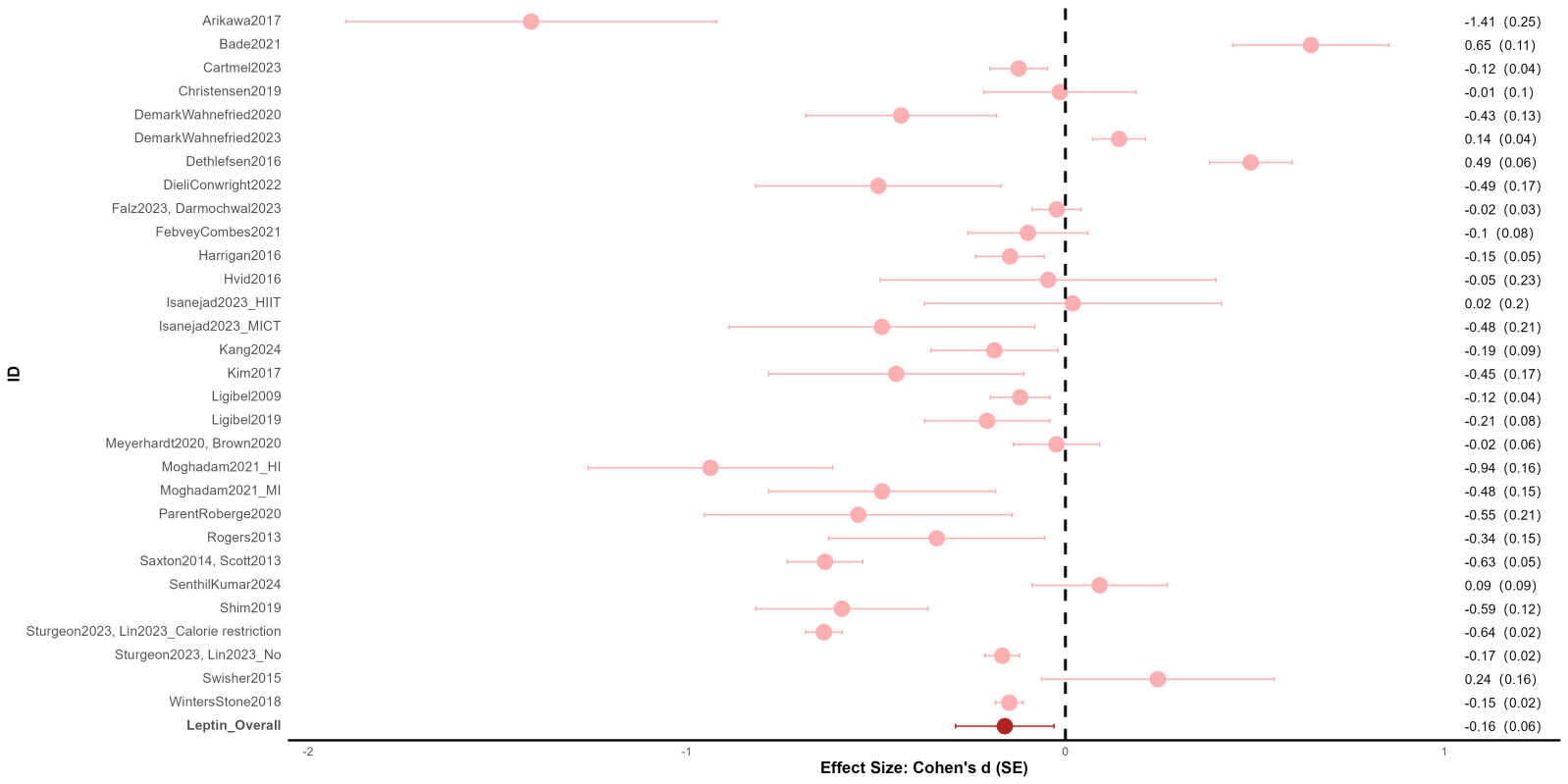


Fig. S5. Forest plot of Leptin


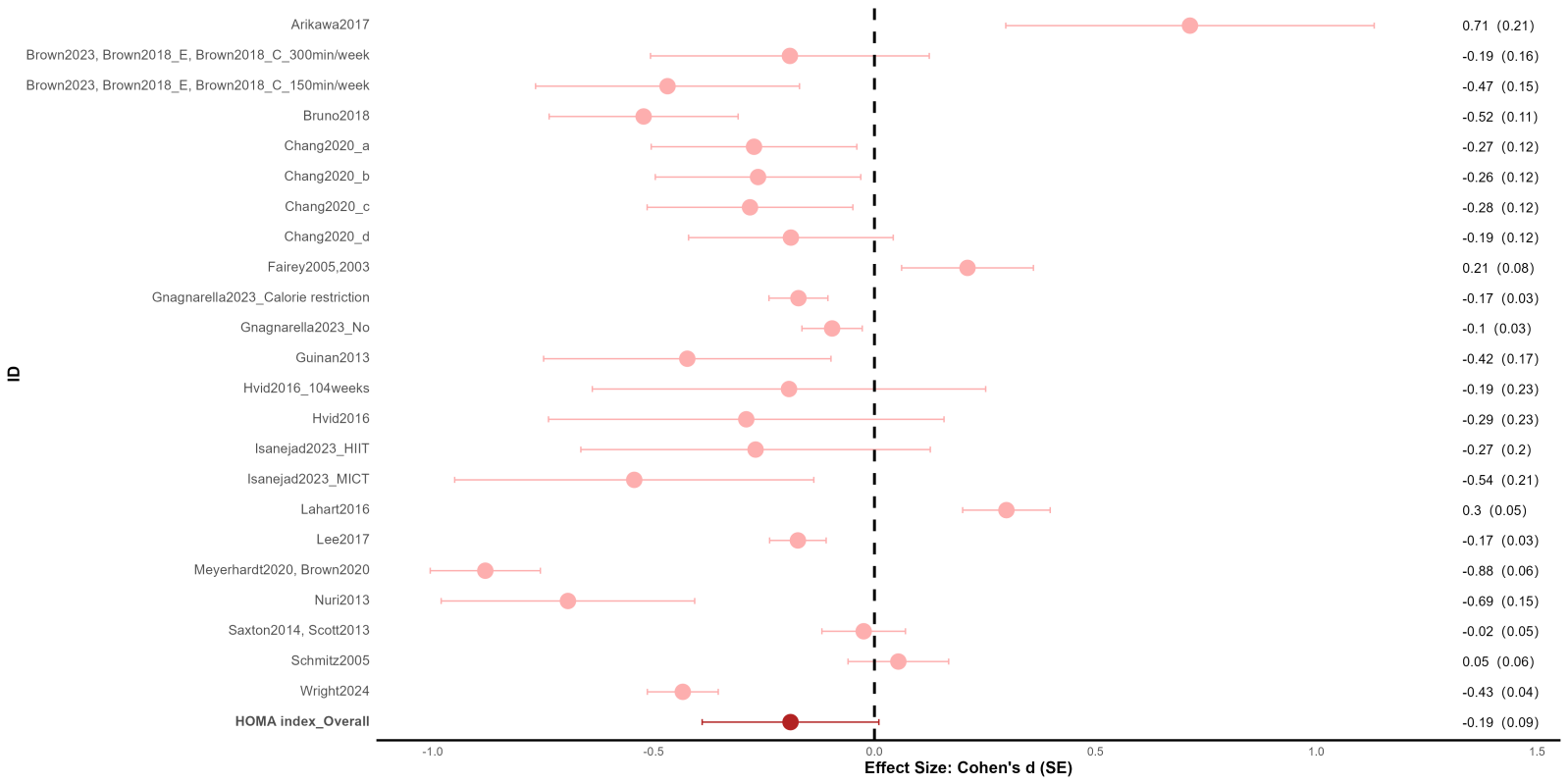


Fig. S6. Forest plot of HOMA index


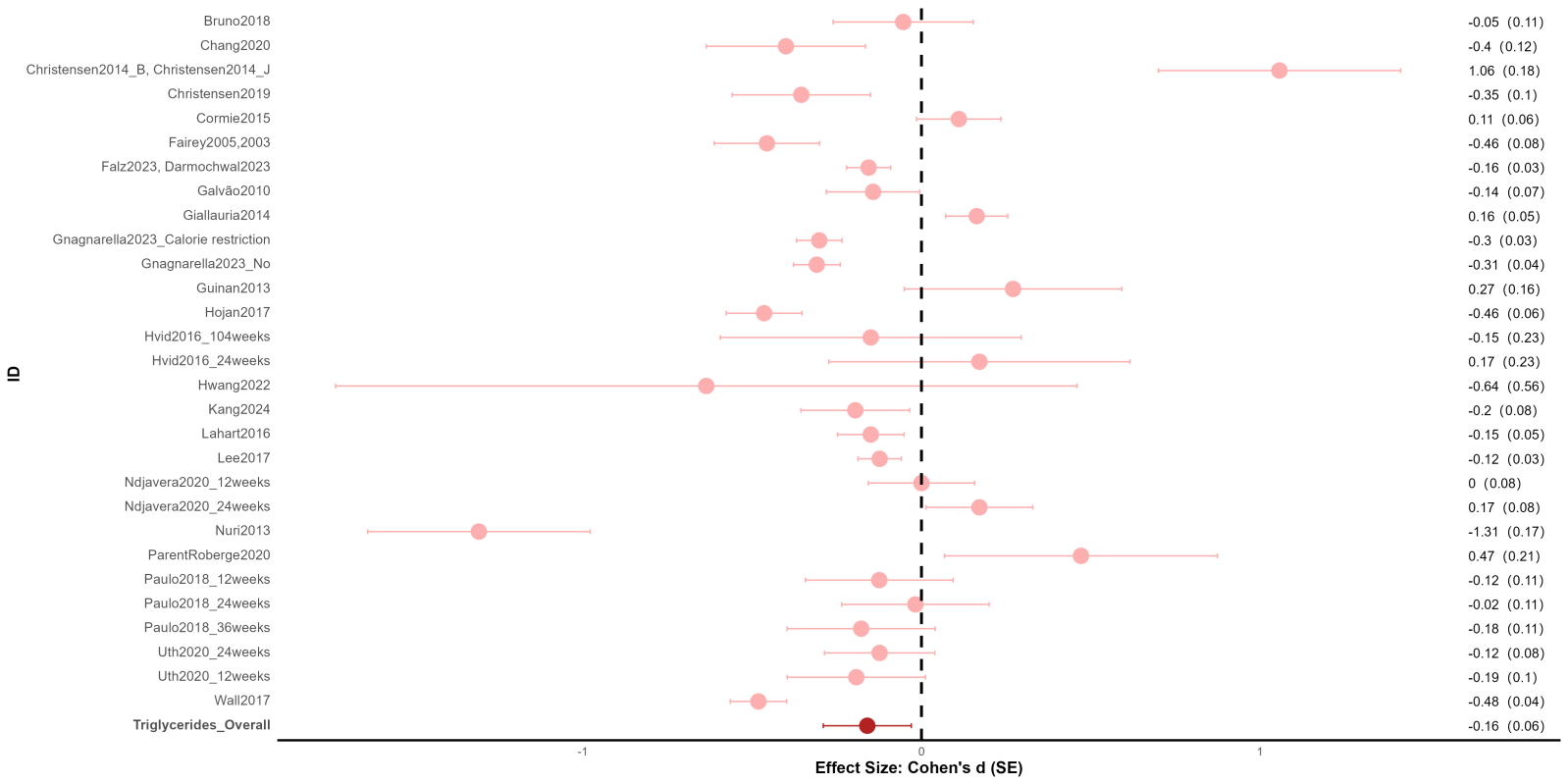


Fig. S7. Forest plot of Triglycerides


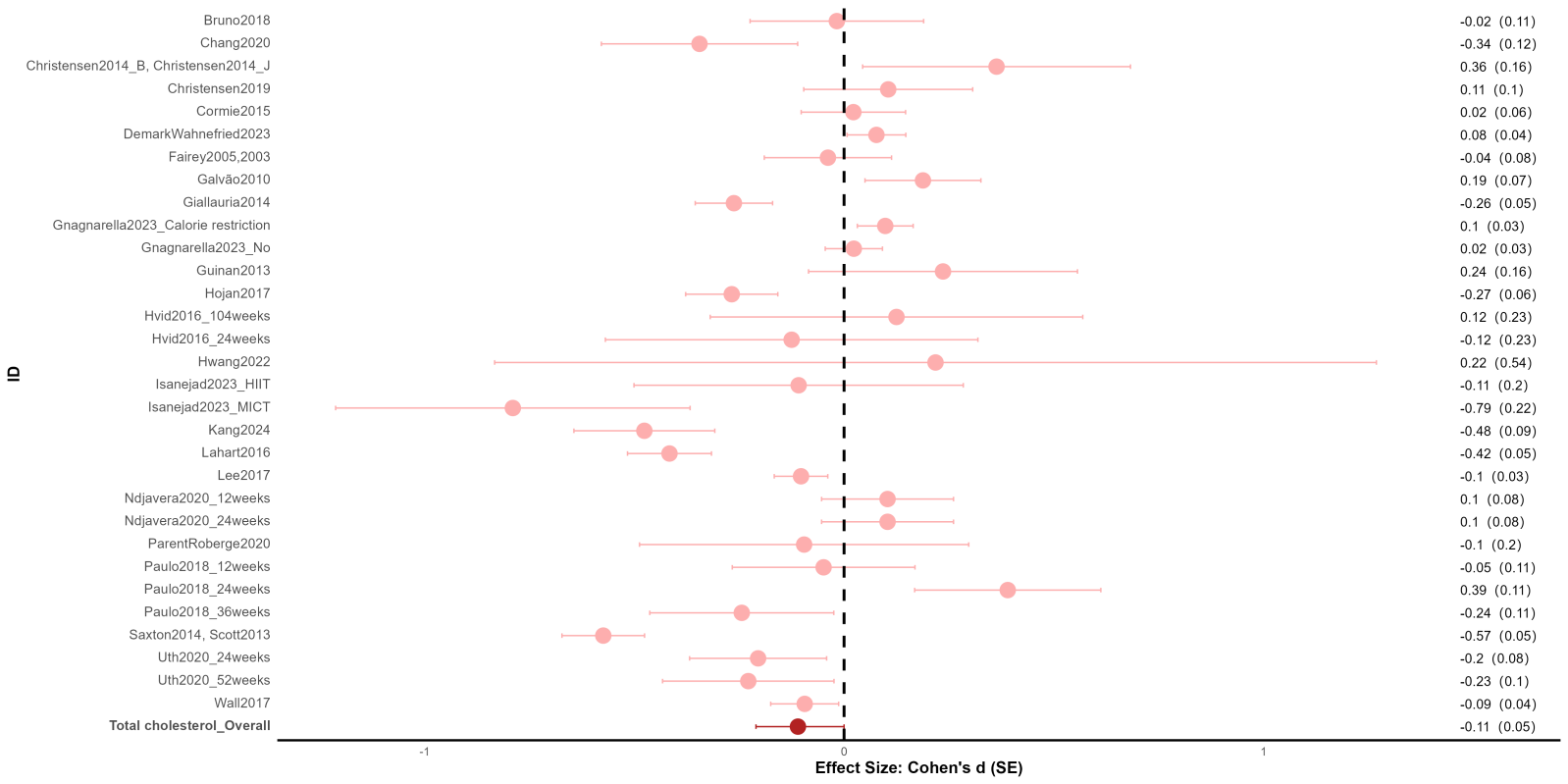


Fig. S8. Forest plot of Total cholesterol


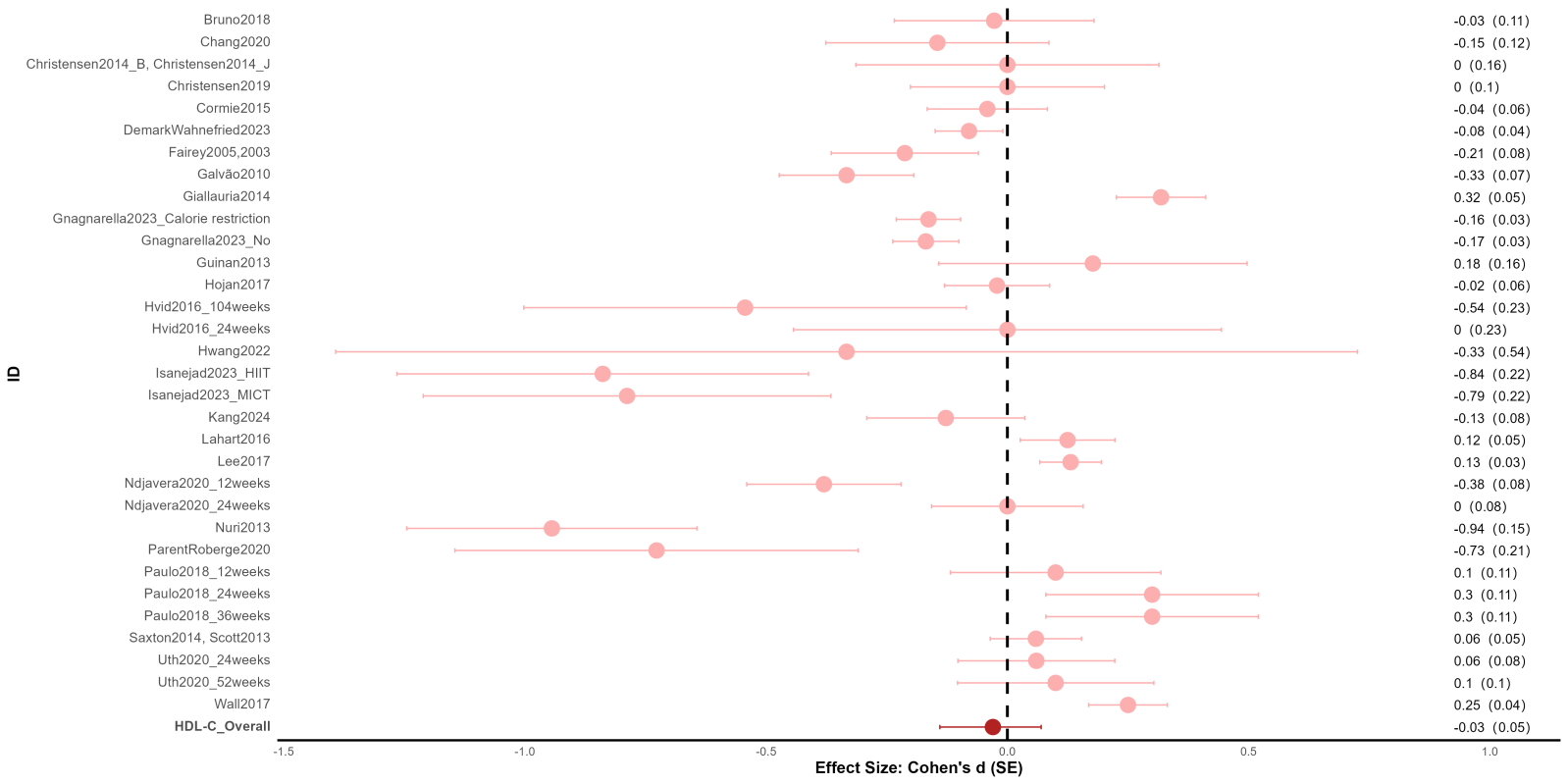


Fig. S9. Forest plot of HDL-C


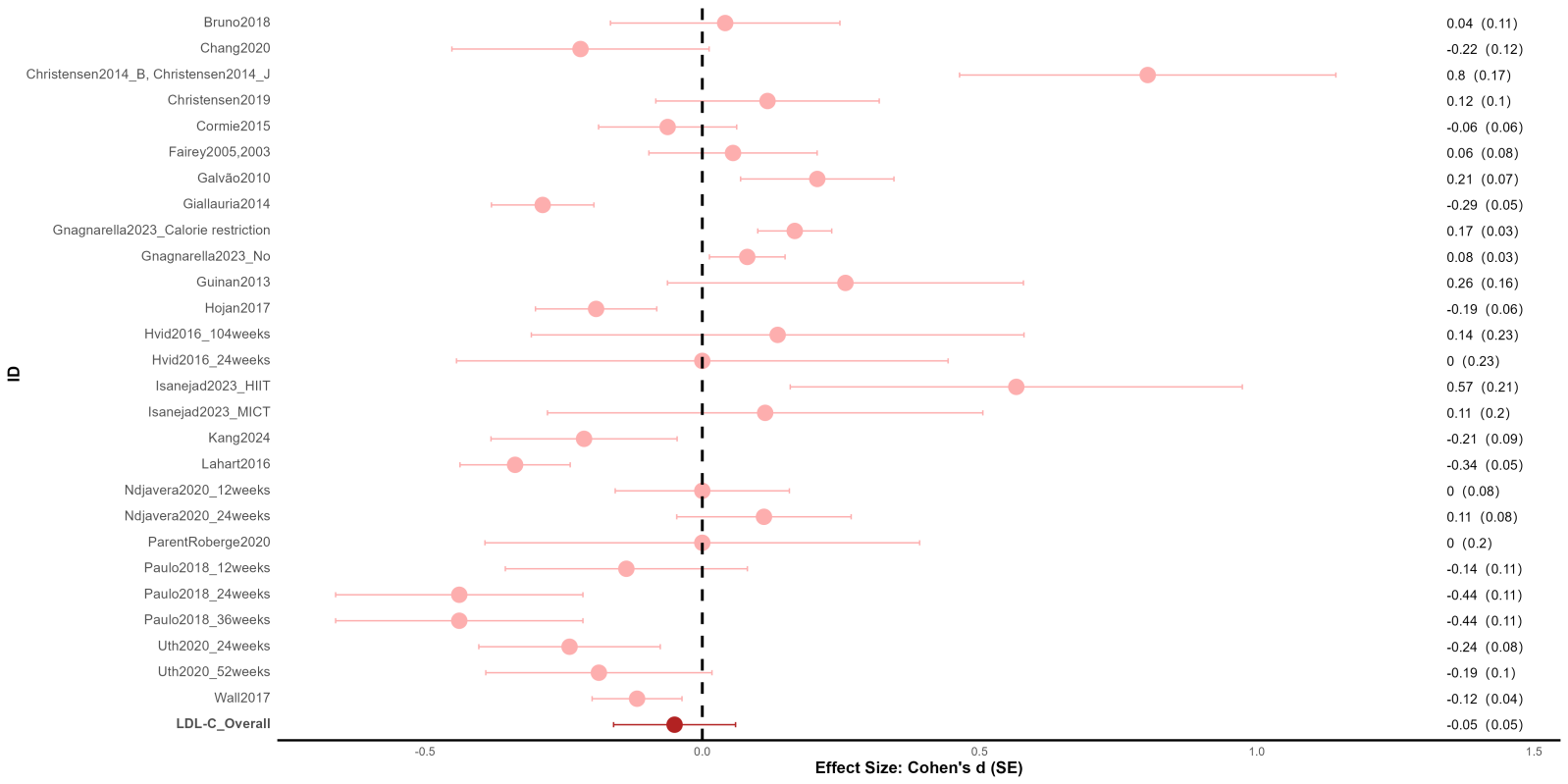


Fig. S10. Forest plot of LDL-C


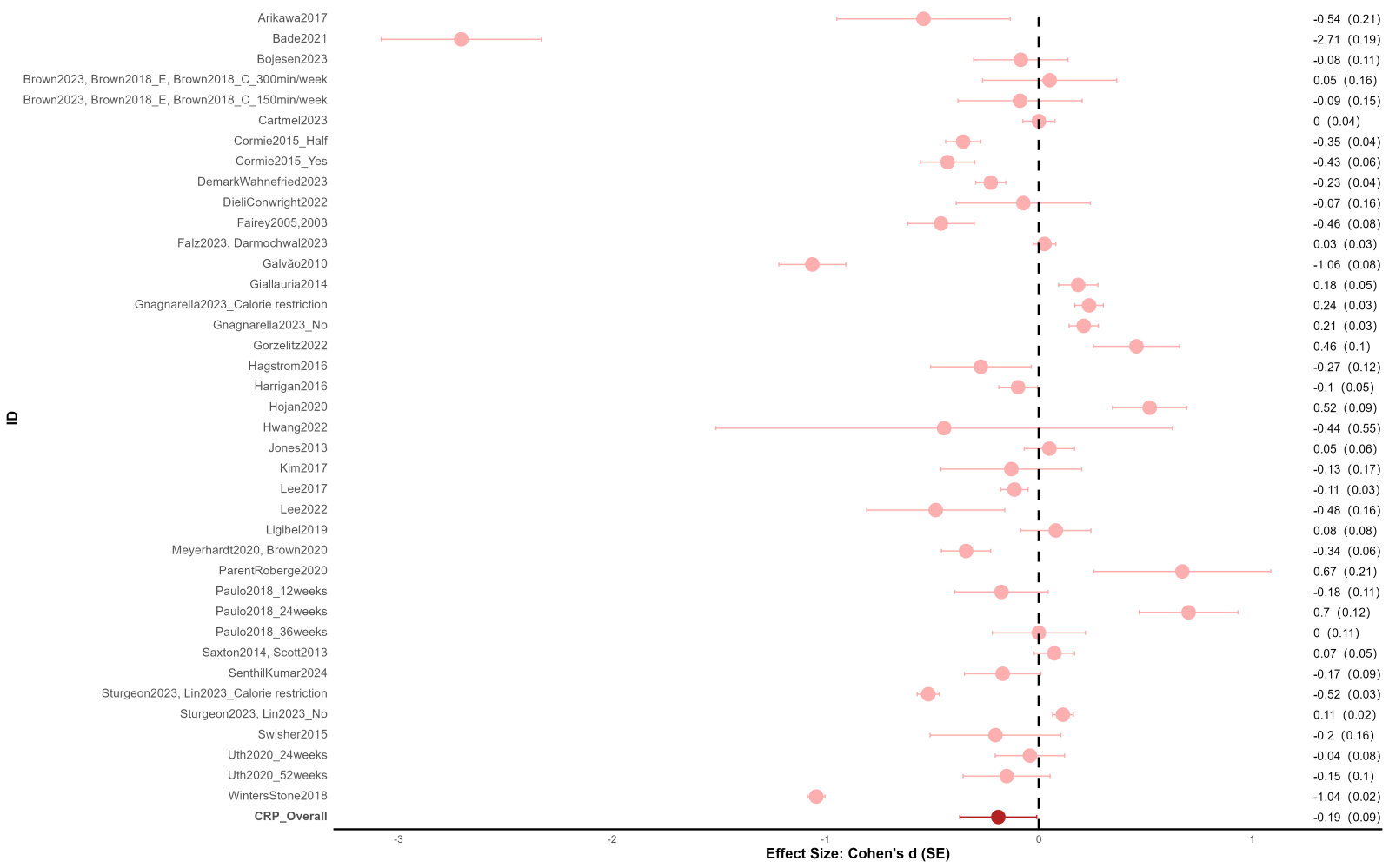


Fig. S11. Forest plot of CRP


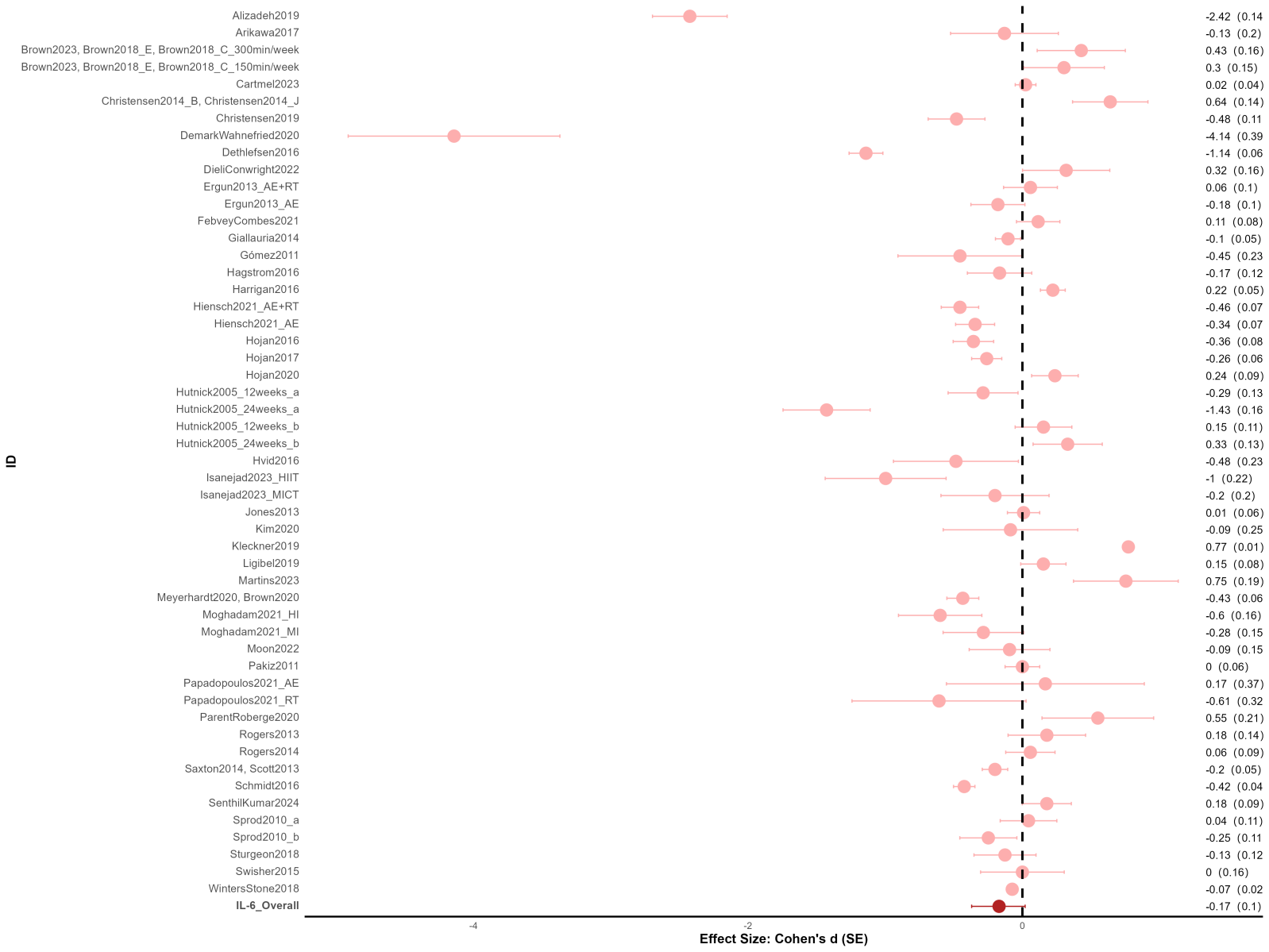


Fig. S12. Forest plot of IL-6


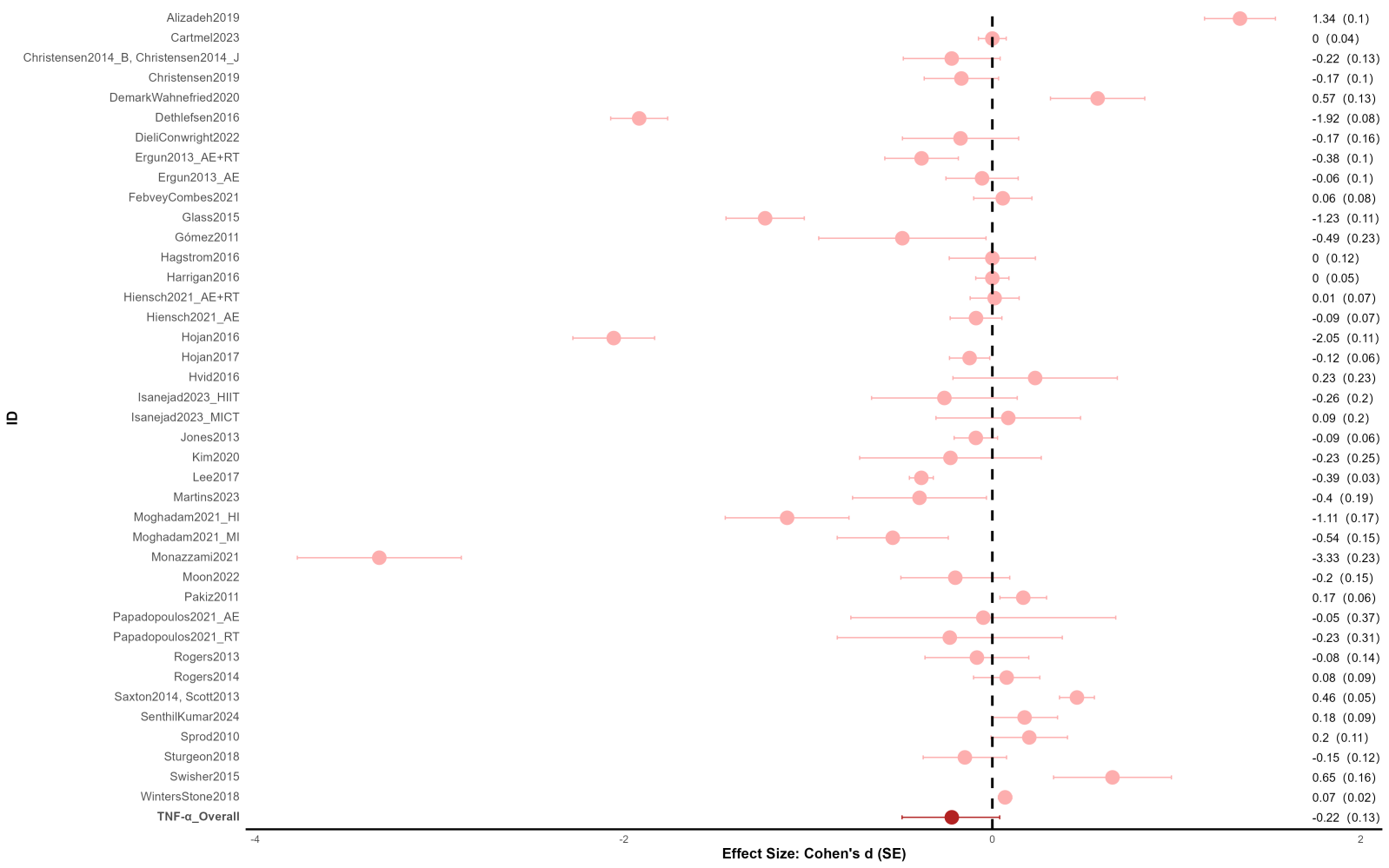


Fig. S13. Forest plot of TNF-α


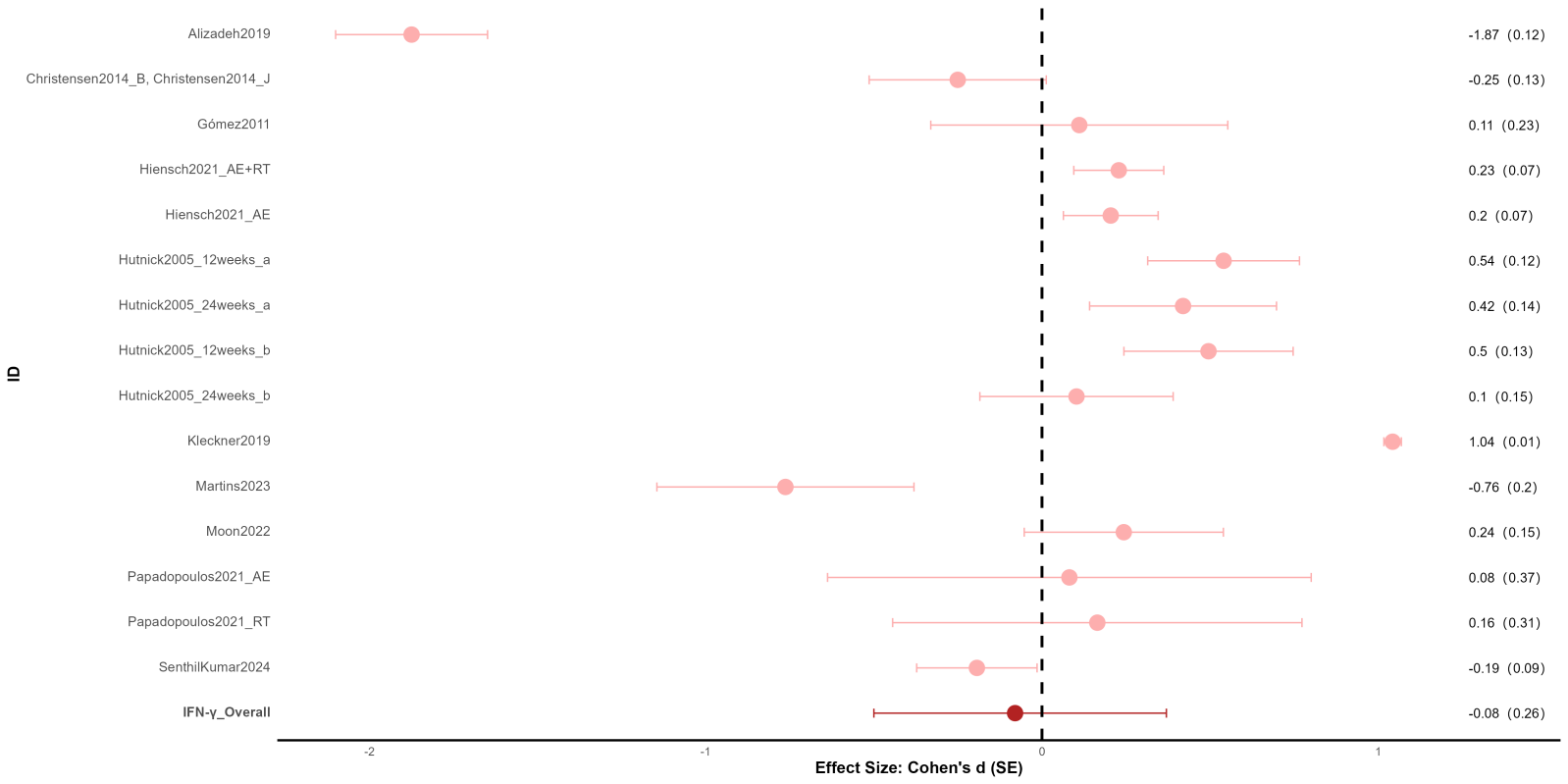


Fig. S14. Forest plot of IFN-γ


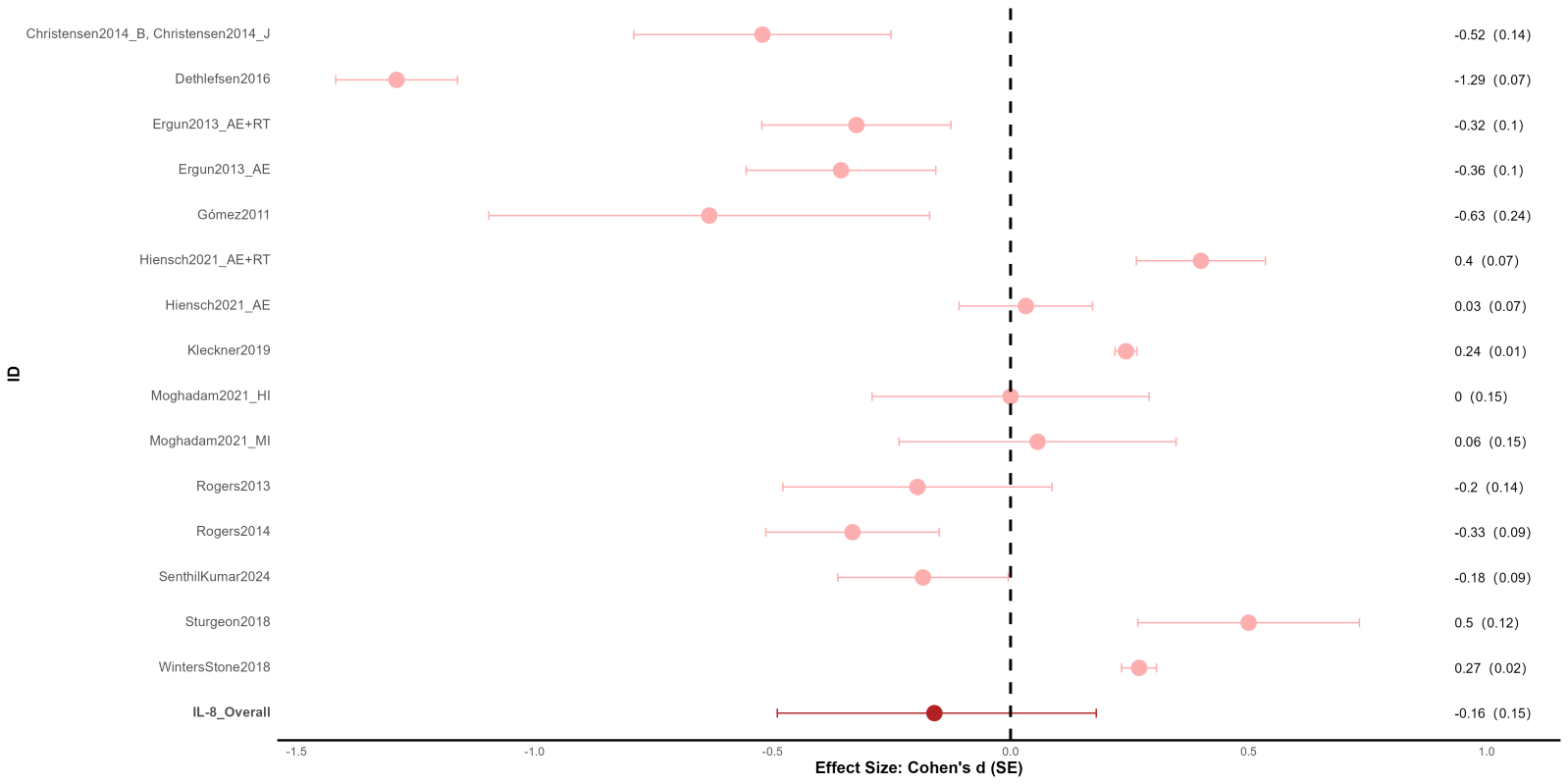


Fig. S15. Forest plot of IL-8


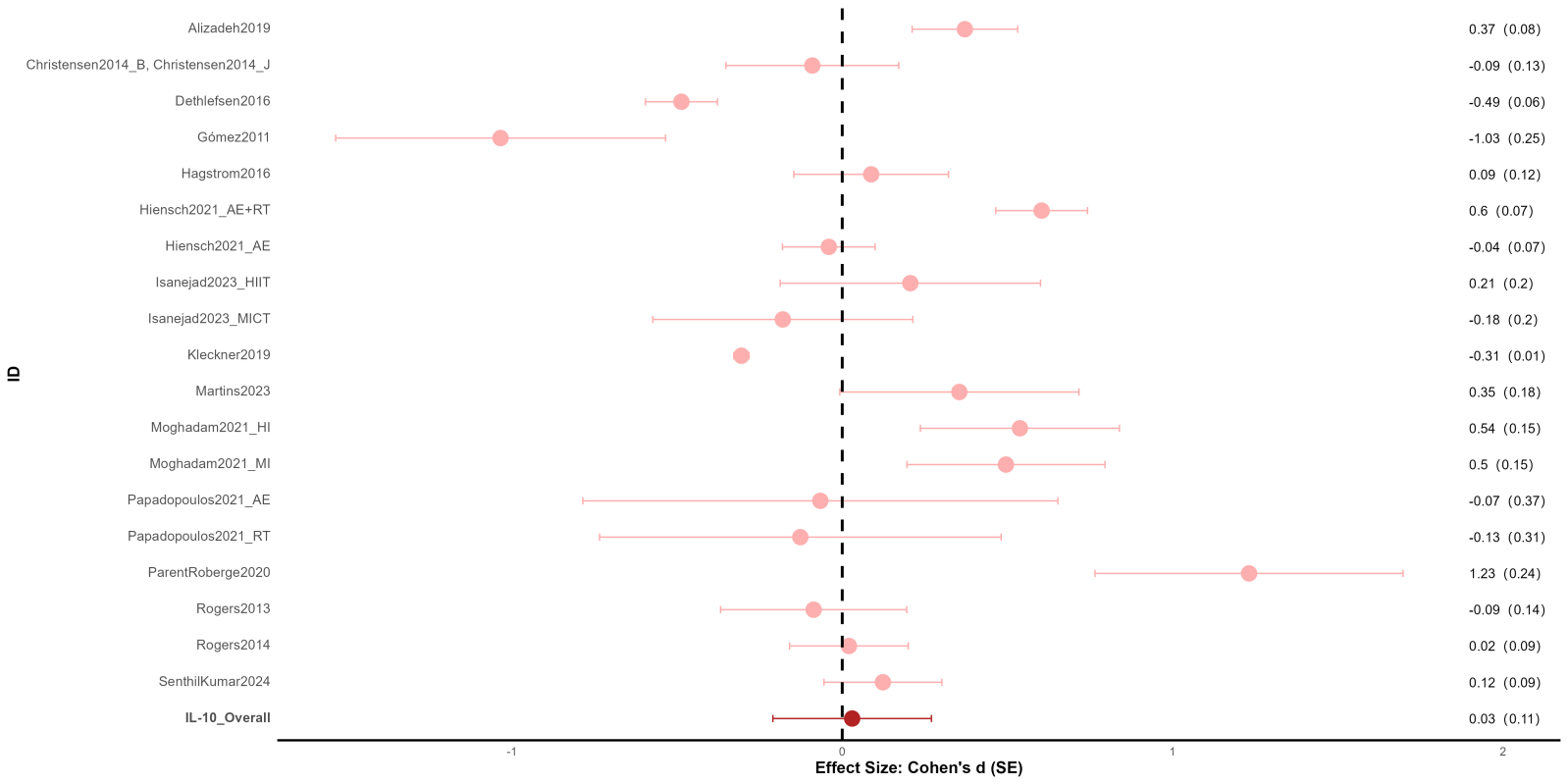


Fig. S16. Forest plot of IL-10

**Supplementary information 6. Risk of Bias and Publication Bias.**


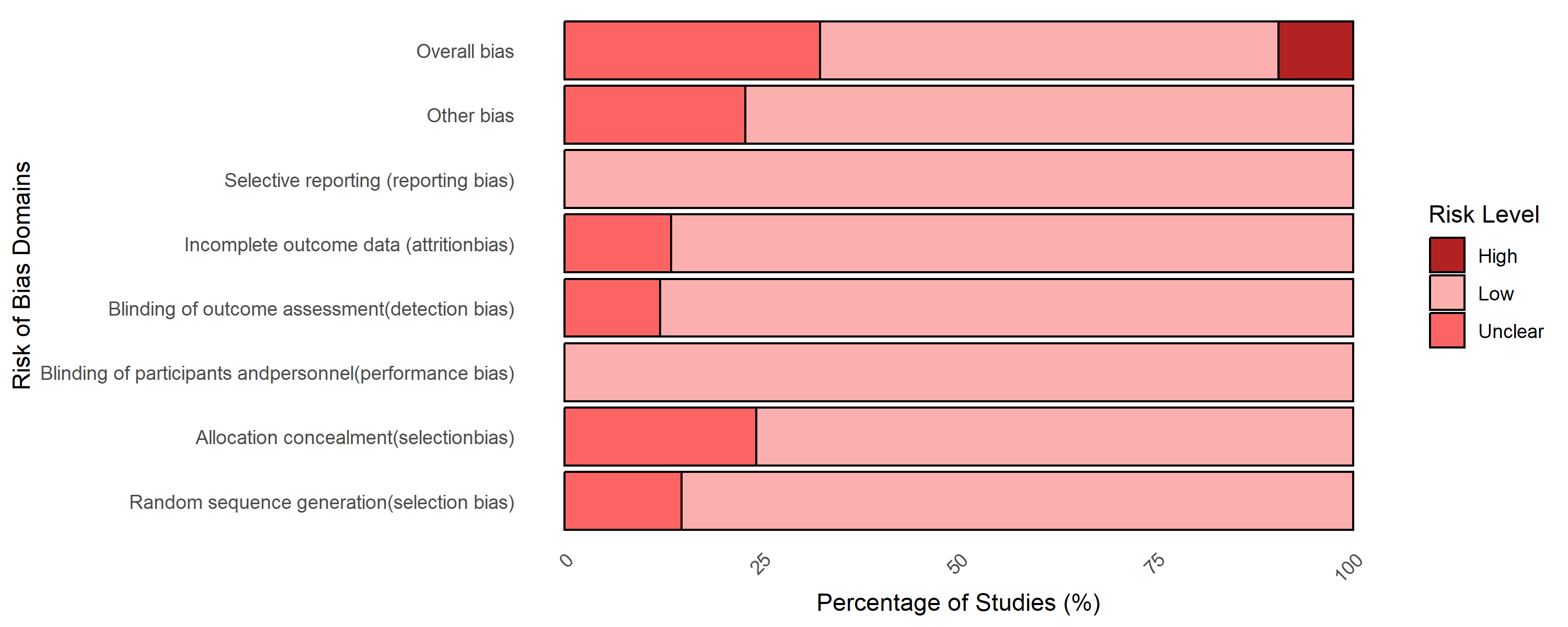


Fig. S17. Summary of risk of bias


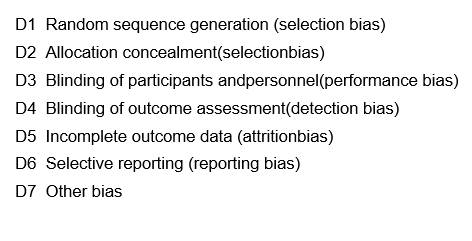

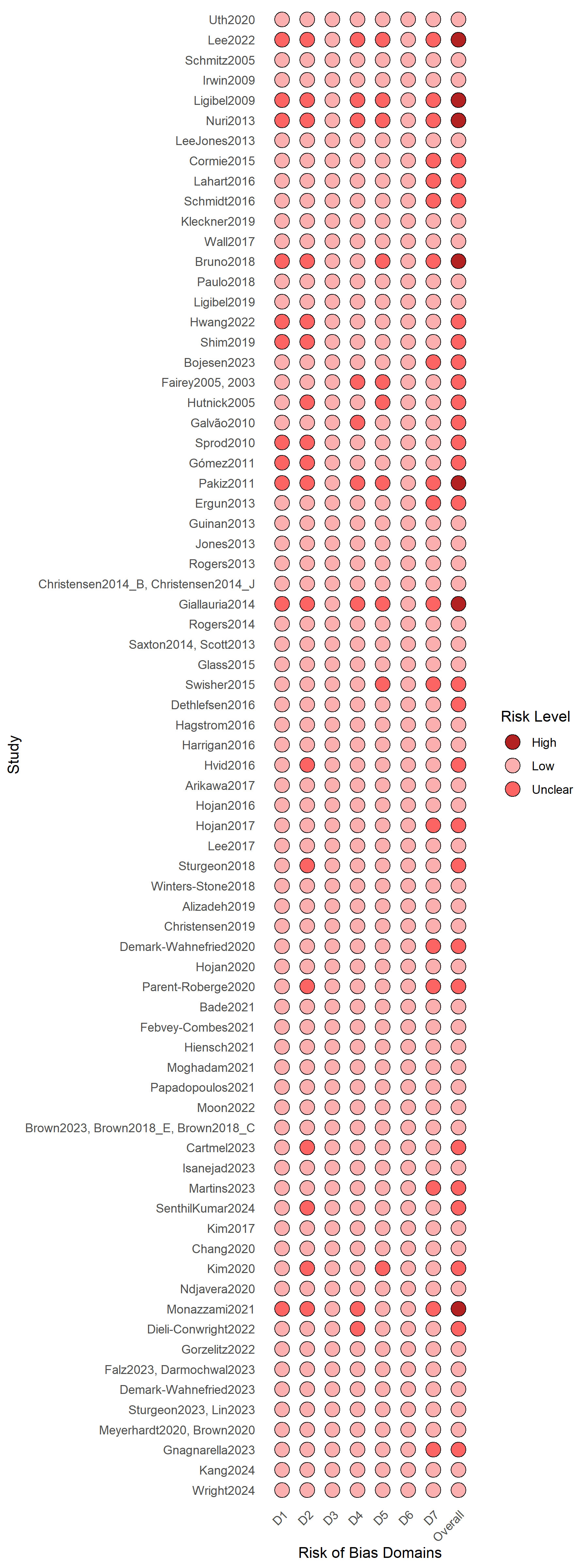


Fig. S18. Details of risk of bias


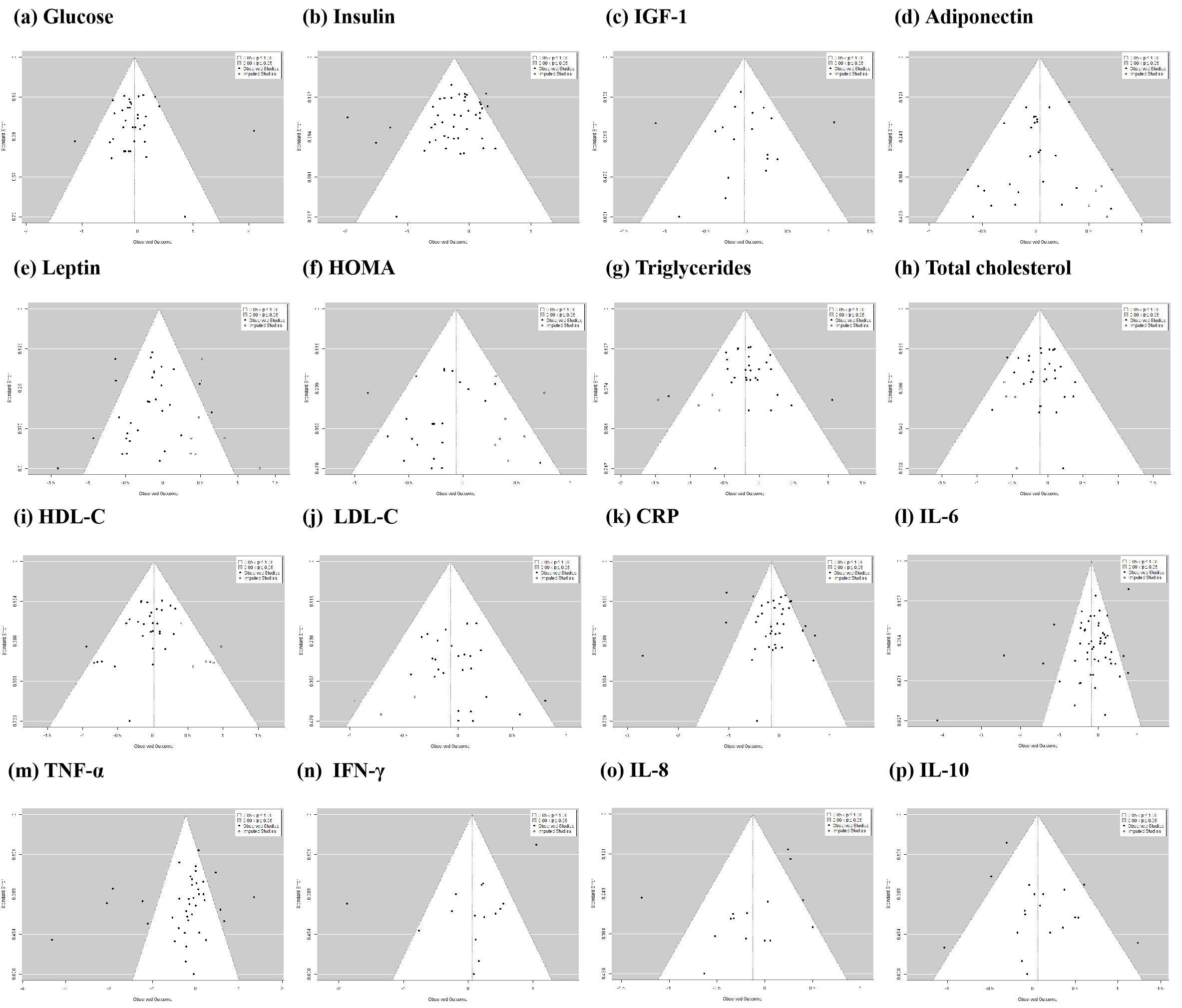


Fig. S19. Funnel plot

**Supplementary information 7. Extraction Strategy for Moderator Variables.**

Table S5. Moderating variable information summary

| NO. | Name | Extraction Strategy |
| --- | --- | --- |
| (a) Exercise Prescription Moderator Variables | | |
| 1 | Type (AE/RT/PA/AE+RT) | AE is aerobic exercise. RT is resistance exercise. PA is a recommendation for increasing physical activity. |
| 2 | EI Duration | Duration is the duration of the exercise intervention. Only pre- and post-intervention values are included. |
| 3 | Single Exercise Intensity | HRmax/HRR (Heart Rate Reserve): 50%–70% = 3–6 MET, 70%–85% = 6–8 MET, >85% = 8-10 MET.  If the intensity of RT is not clearly marked, it is calculated according to 4.5 MET.  Borg scale: 12–20 = 4–10 MET. |
| 4 | Single Exercise Time | Set a time for an exercise session. |
| 5 | Weekly Exercise Frequency | The frequency of exercise per week. If AE and RT are performed separately, they should be calculated separately. |
| 6 | Weekly Exercise Duration | Total time of exercise intervention per week. |
| 7 | Weekly Exercise Volume | Weekly Exercise Volume = Single Exercise Intensity × Weekly Exercise Duration |
| 8 | Supervision (Yes/Half/No) | Yes indicates that the exercise intervention was fully supervised, including in-person or virtual sessions throughout the intervention period. Half indicates partial supervision, such as supervision provided only during the initial weeks, or a combination of supervised and home-based sessions. No indicates no supervision, including interventions with only initial demonstrations, home-based programs without regular contact, or those delivered solely through telephone guidance. |
| 9 | Other Intervention (Calorie restriction/No) | No indicates the absence of structured co-interventions. General recommendations such as simple psychological guidance, basic nutritional supplementation, weight loss advice, or the lack of a clearly defined caloric restriction strategy were all classified as No. |
| (b) Background Moderator Variables | | |
| 1 | Timing (Before/During/Survivor) | Before indicates that the intervention was implemented during the prehabilitation phase or the active surveillance period prior to primary cancer treatment. During indicates that the intervention was delivered during the course of cancer treatment (e.g., surgery, chemotherapy, radiotherapy, or hormone therapy). Survivor indicates that the intervention was administered after the completion of all cancer treatments. |
| 2 | EI Age | Age is the mean age of the intervention group. |
| 3 | CON Age | Age is the mean age of the control group. |
| 4 | EI BMI | BMI is the mean body mass index of the intervention group. |
| 5 | CON BMI | BMI is the mean body mass index of the control group. |
| 6 | Cancer Type | BC = Breast cancer;  CRC = Colorectal cancer;  EC = Endometrial cancer;  GCC = Gastroesophageal and colorectal cancer;  Mixed = Mixed cancer types;  NSCLC = Non-small cell lung cancer;  OC = Ovarian cancer;  PCa = Prostate cancer. |
| 7 | Cancer Stage (0-II) | Cancer Stage (0-II) is the proportion of 0-II cancer patients in the study determined by clinical examinations (such as imaging examinations, physical examinations, etc.) and pathological examinations (such as histology, cytology, etc.). |
| 8 | Publication Year |  |
| 9 | Continents |  |
| 10 | Detection Method | Detection Method refers to the assay technique used for cytokine measurement, such as ELISA, MSD, and others. |
|  | Race* |  |
|  | Surgery Type* |  |
|  | Chemotherapy Regimen* |  |
|  | Comorbidity (Diabetes)* |  |
|  | Comorbidity (Cardiovascular disease)* |  |
|  | Comorbidity (Pulmonary disease)* |  |
|  | Smoking Status* |  |
|  | Drinking Status* |  |
|  | Other symptoms (Sarcopenia)* |  |
|  | Other symptoms (Weakness)* |  |
|  | Weight* |  |
|  | Male/Female* |  |

Note: The asterisk (*) denotes the excluded moderator variables.

Table S6. Frequency statistics of the moderating variables

| No. | Variables Name | Median/N | Interquartile range (Q1–Q3) |
| --- | --- | --- | --- |
| (a) Exercise Prescription Moderator Variables | | | |
| 1 | Type (AE/RT/PA/AE+RT) |  |  |
|  | AE+RT | 211 |  |
|  | AE | 189 |  |
|  | RT | 48 |  |
|  | PA | 33 |  |
| 2 | EI Duration | 12 | 12-24 |
| 3 | Single Exercise Intensity | 6 | 4.5-8 |
| 4 | Single Exercise Time | 42 | 34-60 |
| 5 | Weekly Exercise Frequency | 3 | 3-5 |
| 6 | Weekly Exercise Duration | 180 | 135-246 |
| 7 | Weekly Exercise Volume | 1020 | 840-1350 |
| 8 | Supervision (Yes/Half/No) |  |  |
|  | Yes | 184 |  |
|  | Half | 148 |  |
|  | No | 149 |  |
| 9 | Other Intervention (Calorie restriction/No) |  |  |
|  | Calorie restriction | 75 |  |
|  | No | 406 |  |
| (b) Background Moderator Variables | | | |
| 1 | Timing (Before/During/After) |  |  |
|  | Before | 37 |  |
|  | During | 158 |  |
|  | After | 286 |  |
| 2 | EI Age | 56.3 | 52.4-62 |
| 3 | CON Age | 57 | 52.8-62.1 |
| 4 | EI BMI | 28.2 | 25.98-30.23 |
| 5 | CON BMI | 28.2 | 26.4-29.8 |
| 6 | Cancer Type |  |  |
|  | BC | 293 |  |
|  | PCa | 87 |  |
|  | Mixed | 37 |  |
|  | CRC | 37 |  |
|  | GCC | 11 |  |
|  | OC | 7 |  |
|  | Other | 9 |  |
| 7 | Cancer Stage (0-II) | 0.73 | 0.63-0.9 |
| 8 | Publication Year | 2018 | 2016-2021 |
| 9 | Continents |  |  |
|  | USA | 159 |  |
|  | Denmark | 60 |  |
|  | Canada | 39 |  |
|  | Iran | 44 |  |
|  | Korea | 34 |  |
|  | Italy | 30 |  |
|  | Other | 115 |  |
| 10 | Detection Method |  |  |
|  | ELISA | 158 |  |
|  | NO | 123 |  |
|  | Multiplex | 40 |  |
|  | ACA | 32 |  |
|  | Calculation | 25 |  |
|  | MSD | 25 |  |
|  | Other | 77 |  |

Note: AE is aerobic exercise. RT is resistance exercise. PA is a recommendation for increasing physical activity.

**Supplementary information 8. Results of the RVE Univariate Regression Model.**

| Model | labels | b.r | SE | t | dfs | prob | CI.L | CI.U | sig | I^2^ (%) | R^2^ |
| --- | --- | --- | --- | --- | --- | --- | --- | --- | --- | --- | --- |
| (a) Exercise Prescription Moderator Variables | | | | | | | | | | | |
| EI.Type | | | | | | | | | | | |
| Adiponectin | X.Intercept. (AE) | -0.11 | 0.09 | -1.24 | 5.14 | 0.27 | -0.34 | 0.12 |  | 0 | 0 |
|  | RT | 0.43 | 0.09 | 4.79 | 5.14 | 0.00 | 0.20 | 0.65 | *** |  |  |
| HOMA | X.Intercept. (AE) | -0.40 | 0.14 | -2.80 | 5.77 | 0.03 | -0.75 | -0.05 | ** | 0.23 | 0.29 |
|  | RT | 0.45 | 0.14 | 3.18 | 5.77 | 0.02 | 0.10 | 0.80 | ** |  |  |
| Total cholesterol | X.Intercept. (AE) | -0.15 | 0.07 | -2.08 | 6.37 | 0.08 | -0.33 | 0.02 | * | 0 | 0 |
|  | RT | 0.48 | 0.10 | 5.04 | 1.18 | 0.10 | -0.37 | 1.33 | * |  |  |
| LDL-C | X.Intercept. (AE) | -0.06 | 0.08 | -0.69 | 6.38 | 0.51 | -0.25 | 0.14 |  | 0 | 0 |
|  | RT | 0.86 | 0.08 | 10.71 | 6.38 | 0.00 | 0.67 | 1.05 | *** |  |  |
| IL-6 | X.Intercept. (AE) | -0.41 | 0.21 | -1.94 | 15.69 | 0.07 | -0.86 | 0.04 | * | 0.70 | -0.06 |
|  | PA | 0.63 | 0.21 | 3.00 | 15.69 | 0.01 | 0.18 | 1.08 | *** |  |  |
| Single.Exercise.Intensity | | | | | | | | | | | |
| Adiponectin | X.Intercept. | 0.31 | 0.16 | 1.89 | 9.38 | 0.09 | -0.06 | 0.67 | * | 0 | 0 |
|  | Single.Exercise.Intensity | -0.06 | 0.03 | -2.16 | 7.71 | 0.06 | -0.12 | 0.00 | * |  |  |
| Weekly.Exercise.Duration | | | | | | | | | | | |
| IL-10 | X.Intercept. | 0.42 | 0.10 | 4.09 | 5.81 | 0.01 | 0.16 | 0.67 | *** | 0.26 | 0.48 |
|  | Weekly.Exercise.Duration | 0.00 | 0.00 | -4.17 | 2.73 | 0.03 | 0.00 | 0.00 | ** |  |  |
| Weekly.Exercise.Volume | | | | | | | | | | | |
| HOMA | X.Intercept. | 0.60 | 0.32 | 1.89 | 3.51 | 0.14 | -0.33 | 1.52 |  | 0.09 | 0.75 |
|  | Weekly.Exercise.Volume | -0.00 | 0.00 | -2.48 | 3.87 | 0.07 | 0.00 | 0.00 | * |  |  |
| CRP | X.Intercept. | -0.52 | 0.20 | -2.63 | 8.21 | 0.03 | -0.98 | -0.07 | ** | 0.59 | 0.14 |
|  | Weekly.Exercise.Volume | 0.00 | 0.00 | 2.62 | 3.33 | 0.07 | 0.00 | 0.00 | * |  |  |
| IL-10 | X.Intercept. | 0.36 | 0.10 | 3.48 | 10.24 | 0.01 | 0.13 | 0.58 | *** | 0.17 | 0.64 |
|  | Weekly.Exercise.Volume | -0.00 | 0.00 | -4.03 | 2.81 | 0.03 | 0.00 | 0.00 | ** |  |  |
| EI.Duration | | | | | | | | | | | |
| LDL-C | X.Intercept. | 0.10 | 0.08 | 1.14 | 14.62 | 0.27 | -0.08 | 0.27 |  | 0 | 0 |
|  | EI.Duration | -0.01 | 0.00 | -2.27 | 4.93 | 0.07 | -0.01 | 0.00 | * |  |  |
| Supervision | | | | | | | | | | | |
| Adiponectin | X.Intercept. | 0.13 | 0.08 | 1.65 | 4.14 | 0.17 | -0.09 | 0.35 |  | 0 | 0 |
|  | No | -0.27 | 0.10 | -2.60 | 9.97 | 0.03 | -0.51 | -0.04 | ** |  |  |
| HDL-C | X.Intercept. | 0.05 | 0.06 | 0.81 | 5.39 | 0.45 | -0.11 | 0.21 |  | 0 | 0 |
|  | No | -0.26 | 0.11 | -2.39 | 3.19 | 0.09 | -0.59 | 0.07 | * |  |  |
| LDL-C | X.Intercept. | -0.11 | 0.07 | -1.60 | 4.65 | 0.18 | -0.29 | 0.07 |  | 0 | 0 |
|  | No | 0.25 | 0.07 | 3.39 | 3.69 | 0.03 | 0.04 | 0.46 | ** |  |  |
| Other.Intervention | | | | | | | | | | | |
| TNF-α | X.Intercept. | 0.26 | 0.11 | 2.40 | 5.93 | 0.05 | -0.01 | 0.52 | * | 0.73 | 0.07 |
|  | No | -0.61 | 0.19 | -3.22 | 9.32 | 0.01 | -1.03 | -0.18 | *** |  |  |
| (b) Background Moderator Variables | | | | | | | | | | | |
| Timing | | | | | | | | | | | |
| IGF-1 | X.Intercept. (Before) | -0.42 | 0.03 | -12.19 | 1.76 | 0.01 | -0.58 | -0.25 | ** | 0.53 | -0.07 |
|  | During | 0.48 | 0.03 | 14.02 | 2.48 | 0.00 | 0.36 | 0.60 | *** |  |  |
|  | Survivor | 0.44 | 0.16 | 2.68 | 2.53 | 0.09 | -0.14 | 1.01 | * |  |  |
| EI.BMI | | | | | | | | | | | |
| TNF-α | X.Intercept. | -2.89 | 1.00 | -2.88 | 12.23 | 0.01 | -5.07 | -0.71 | ** | 0.71 | 0.13 |
|  | EI.BMI | 0.10 | 0.03 | 2.82 | 11.72 | 0.02 | 0.02 | 0.17 | ** |  |  |
| CON.BMI | | | | | | | | | | | |
| TNF-α | X.Intercept. | -2.35 | 0.87 | -2.68 | 13.25 | 0.02 | -4.23 | -0.46 | ** | 0.72 | 0.09 |
|  | CON.BMI | 0.07 | 0.03 | 2.59 | 12.67 | 0.02 | 0.01 | 0.14 | ** |  |  |
| Cancer.Type | | | | | | | | | | | |
| Glucose | X.Intercept. (BC) | -0.10 | 0.07 | -1.27 | 11.99 | 0.23 | -0.26 | 0.07 |  | 0.40 | -0.24 |
|  | GCC | -0.40 | 0.07 | -5.41 | 11.99 | 0.00 | -0.57 | -0.24 | *** |  |  |
| Insulin | X.Intercept. (BC) | -0.27 | 0.11 | -2.43 | 20.68 | 0.02 | -0.49 | -0.04 | ** | 0.5 | -0.06 |
|  | GCC | 0.48 | 0.11 | 4.36 | 20.68 | 0.00 | 0.25 | 0.70 | *** |  |  |
|  | NSCLC | -1.01 | 0.11 | -9.29 | 20.68 | 0.00 | -1.24 | -0.79 | *** |  |  |
|  | OC | 0.20 | 0.11 | 1.82 | 20.68 | 0.08 | -0.03 | 0.43 | * |  |  |
| IGF-1 | X.Intercept. (BC) | -0.05 | 0.18 | -0.26 | 8.74 | 0.80 | -0.46 | 0.37 |  | 0.6 | -0.47 |
|  | CRC | 0.36 | 0.18 | 1.96 | 8.74 | 0.08 | -0.06 | 0.77 | * |  |  |
|  | Mixed | 0.34 | 0.18 | 1.88 | 8.74 | 0.09 | -0.07 | 0.76 | * |  |  |
| Leptin | X.Intercept. (BC) | -0.23 | 0.08 | -2.79 | 12.59 | 0.02 | -0.41 | -0.05 | ** | 0.25 | 0.11 |
|  | NSCLC | 0.88 | 0.08 | 10.60 | 12.59 | 0.00 | 0.70 | 1.06 | *** |  |  |
| HOMA | X.Intercept. (BC) | -0.06 | 0.09 | -0.67 | 7.66 | 0.52 | -0.27 | 0.15 |  | 0.08 | 0.78 |
|  | Mixed | -0.82 | 0.09 | -9.19 | 7.66 | 0.00 | -1.03 | -0.61 | *** |  |  |
| Triglycerides | X.Intercept. (BC) | -0.21 | 0.10 | -2.13 | 7.44 | 0.07 | -0.44 | 0.02 | * | 0.12 | 0.44 |
|  | GCC | 1.27 | 0.10 | 12.98 | 7.44 | 0.00 | 1.04 | 1.49 | *** |  |  |
| Total cholesterol | X.Intercept. (BC) | -0.20 | 0.09 | -2.21 | 7.62 | 0.06 | -0.40 | 0.01 | * | 0 | 0 |
|  | GCC | 0.56 | 0.09 | 6.30 | 7.62 | 0.00 | 0.35 | 0.77 | *** |  |  |
| LDL-C | X.Intercept. (BC) | -0.09 | 0.08 | -1.08 | 6.67 | 0.32 | -0.29 | 0.11 |  | 0 | 0 |
|  | CRC | 0.21 | 0.08 | 2.48 | 6.67 | 0.04 | 0.01 | 0.41 | ** |  |  |
|  | GCC | 0.89 | 0.08 | 10.63 | 6.67 | 0.00 | 0.69 | 1.10 | *** |  |  |
| CRP | X.Intercept. (BC) | -0.13 | 0.09 | -1.36 | 16.92 | 0.19 | -0.33 | 0.07 |  | 0.53 | 0.33 |
|  | EC | 0.59 | 0.09 | 6.21 | 16.92 | 0.00 | 0.39 | 0.78 | *** |  |  |
|  | NSCLC | -2.58 | 0.09 | -27.36 | 16.92 | 0.00 | -2.78 | -2.38 | *** |  |  |
| IL-6 | X.Intercept. (BC) | -0.25 | 0.13 | -1.99 | 27.13 | 0.06 | -0.51 | 0.01 | * | 0.66 | 0.09 |
|  | GCC | 0.89 | 0.13 | 7.08 | 27.13 | 0.00 | 0.63 | 1.15 | *** |  |  |
|  | OC | 0.27 | 0.13 | 2.17 | 27.13 | 0.04 | 0.01 | 0.53 | ** |  |  |
| IFN-γ | X.Intercept. (BC) | 0.35 | 0.35 | 0.99 | 4.97 | 0.37 | -0.56 | 1.26 |  | 0.78 | 0.33 |
|  | Mixed | -1.39 | 0.35 | -3.94 | 4.97 | 0.01 | -2.31 | -0.48 | ** |  |  |
| Nation | | | | | | | | | | | |
| Glucose | X.Intercept. (Australia) | -0.38 | 0.05 | -7.43 | 1.98 | 0.02 | -0.61 | -0.16 | ** | 0.35 | -0.01 |
|  | Brazil | 0.25 | 0.05 | 4.91 | 1.98 | 0.04 | 0.03 | 0.48 | ** |  |  |
|  | Canada | 0.34 | 0.07 | 4.50 | 3.67 | 0.01 | 0.12 | 0.55 | ** |  |  |
|  | Ireland | 0.51 | 0.05 | 9.92 | 1.98 | 0.01 | 0.29 | 0.74 | ** |  |  |
|  | Italy | 0.36 | 0.12 | 2.93 | 2.25 | 0.09 | -0.12 | 0.83 | * |  |  |
|  | Korea | 0.53 | 0.10 | 5.12 | 2.96 | 0.01 | 0.20 | 0.87 | ** |  |  |
|  | USA | 0.30 | 0.09 | 3.16 | 3.74 | 0.04 | 0.03 | 0.56 | ** |  |  |
| Insulin | X.Intercept. (Australia) | -0.10 | 0.15 | -0.70 | 2.99 | 0.54 | -0.57 | 0.36 |  | 0.53 | -0.16 |
|  | Ireland | -0.44 | 0.15 | -3.02 | 2.99 | 0.06 | -0.91 | 0.02 | * |  |  |
| IGF-1 | X.Intercept. (Canada) | -0.38 | 0.06 | -6.55 | 1.79 | 0.03 | -0.66 | -0.10 | ** | 0.6 | -0.42 |
|  | France | 0.44 | 0.06 | 7.61 | 1.79 | 0.02 | 0.16 | 0.72 | ** |  |  |
|  | Italy | 0.22 | 0.06 | 3.82 | 1.79 | 0.07 | -0.06 | 0.50 | * |  |  |
|  | UK | 0.45 | 0.06 | 7.71 | 1.79 | 0.02 | 0.17 | 0.73 | ** |  |  |
| Triglycerides | X.Intercept. (Australia) | -0.20 | 0.19 | -1.06 | 1.97 | 0.40 | -1.01 | 0.62 |  | 0.25 | -0.33 |
|  | Iran | -1.11 | 0.19 | -5.98 | 1.97 | 0.03 | -1.92 | -0.30 | ** |  |  |
| Total cholesterol | X.Intercept. (Australia) | 0.01 | 0.08 | 0.16 | 1.89 | 0.89 | -0.37 | 0.40 |  | 0.25 | -0.33 |
|  | Iran | -0.46 | 0.08 | -5.52 | 1.89 | 0.04 | -0.84 | -0.08 | ** |  |  |
|  | Poland | -0.28 | 0.08 | -3.36 | 1.89 | 0.08 | -0.66 | 0.10 | * |  |  |
| HDL-C | X.Intercept. (Australia) | 0.01 | 0.18 | 0.07 | 1.89 | 0.95 | -0.80 | 0.82 |  | 0 | 0 |
|  | Iran | -0.90 | 0.19 | -4.77 | 1.42 | 0.08 | -2.13 | 0.33 | * |  |  |
| LDL-C | X.Intercept. (Australia) | -0.02 | 0.09 | -0.18 | 1.89 | 0.88 | -0.44 | 0.41 |  | 0 | 0 |
|  | Brazil | -0.32 | 0.09 | -3.46 | 1.89 | 0.08 | -0.74 | 0.10 | * |  |  |
|  | Iran | 0.36 | 0.09 | 3.84 | 1.89 | 0.07 | -0.07 | 0.78 | * |  |  |
| CRP | X.Intercept. (Australia) | -0.58 | 0.24 | -2.40 | 1.98 | 0.14 | -1.63 | 0.47 |  | 0.65 | -0.13 |
|  | Brazil | 0.76 | 0.24 | 3.12 | 1.98 | 0.09 | -0.30 | 1.81 | * |  |  |
|  | Italy | 0.79 | 0.24 | 3.23 | 2.43 | 0.06 | -0.10 | 1.67 | * |  |  |
|  | Poland | 1.10 | 0.24 | 4.54 | 1.98 | 0.05 | 0.05 | 2.15 | ** |  |  |
| Detection.Method | | | | | | | | | | | |
| CRP | X.Intercept. (ACA) | -0.07 | 0.05 | -1.26 | 3.90 | 0.28 | -0.22 | 0.08 |  | 0.66 | -0.16 |
|  | MSD | -0.14 | 0.05 | -2.51 | 3.90 | 0.07 | -0.29 | 0.02 | * |  |  |
| IL-8 | X.Intercept. (ACA) | -0.22 | 0.08 | -2.72 | 2.96 | 0.07 | -0.49 | 0.04 | * | 0.53 | 0.12 |
|  | Multiplex | 0.41 | 0.16 | 2.60 | 5.48 | 0.04 | 0.02 | 0.81 | ** |  |  |
|  | PELISA | 0.44 | 0.08 | 5.35 | 2.96 | 0.01 | 0.18 | 0.70 | ** |  |  |

**Supplementary information 9. Results of the MetaForest Regression Model.**

Table S7. Parameters of the MetaForest regression model with exercise prescription moderator variables

| ID | R²_OOB_ | τ² | Feature Weights | Mtry | min.node.size | R²_CV_ | R²_CV_ SD | RMSE | RMSE SD | MAE | MAE SD |
| --- | --- | --- | --- | --- | --- | --- | --- | --- | --- | --- | --- |
| Glucose | -0.15 | 0.14 | fixed | 2.00 | 4.00 | 0.07 | 0.08 | 0.49 | 0.17 | 0.34 | 0.09 |
| Insulin | -0.26 | 0.18 | fixed | 2.00 | 4.00 | 0.04 | 0.06 | 0.51 | 0.12 | 0.39 | 0.08 |
| IGF-1 | -0.35 | 0.22 | unif | 2.00 | 2.00 | 0.14 | 0.21 | 0.53 | 0.14 | 0.42 | 0.11 |
| Adiponectin | -0.05 | 0.02 | fixed | 2.00 | 4.00 | 0.09 | 0.13 | 0.31 | 0.06 | 0.24 | 0.05 |
| Leptin | -0.22 | 0.09 | fixed | 2.00 | 4.00 | 0.05 | 0.07 | 0.44 | 0.08 | 0.36 | 0.06 |
| HOMA index | -0.08 | 0.03 | fixed | 2.00 | 4.00 | 0.12 | 0.17 | 0.36 | 0.09 | 0.27 | 0.07 |
| Triglycerides | -0.07 | 0.01 | fixed | 2.00 | 4.00 | 0.08 | 0.11 | 0.41 | 0.12 | 0.29 | 0.08 |
| Total cholesterol | -0.13 | 0.00 | fixed | 2.00 | 4.00 | 0.06 | 0.08 | 0.28 | 0.05 | 0.22 | 0.04 |
| HDL-C | 0.05 | 0.00 | random | 2.00 | 2.00 | 0.13 | 0.13 | 0.32 | 0.06 | 0.25 | 0.05 |
| LDL-C | 0.10 | 0.00 | unif | 2.00 | 2.00 | 0.19 | 0.16 | 0.26 | 0.08 | 0.20 | 0.05 |
| CRP | -0.18 | 0.24 | fixed | 2.00 | 4.00 | 0.05 | 0.06 | 0.54 | 0.18 | 0.37 | 0.09 |
| IL-6 | -0.08 | 0.30 | fixed | 2.00 | 4.00 | 0.07 | 0.10 | 0.76 | 0.29 | 0.47 | 0.11 |
| TNF-α | -0.17 | 0.57 | fixed | 2.00 | 4.00 | 0.10 | 0.11 | 0.82 | 0.22 | 0.55 | 0.14 |
| IFN-γ | -0.17 | 0.46 | fixed | 2.00 | 4.00 | 0.20 | 0.20 | 0.72 | 0.27 | 0.53 | 0.18 |
| IL-8 | -0.37 | 0.28 | unif | 2.00 | 2.00 | 0.30 | 0.31 | 0.46 | 0.14 | 0.38 | 0.12 |
| IL-10 | 0.00 | 0.05 | fixed | 2.00 | 4.00 | 0.35 | 0.28 | 0.46 | 0.17 | 0.35 | 0.12 |

Note: R^2^_OOB_: Out-of-Bag R-Squared. τ²: Between-Studies Variance. R^2^_CV_: Cross-Validation R-Squared. R^2^_CV_ SD: Cross-Validation R-Squared Standard Deviation. Mtry: Number of Variables Randomly Sampled as Candidates at Each Split. min.node.size: Minimum Node Size. RMSE: Root Mean Square Error. MASE: Mean Absolute Scaled Error.


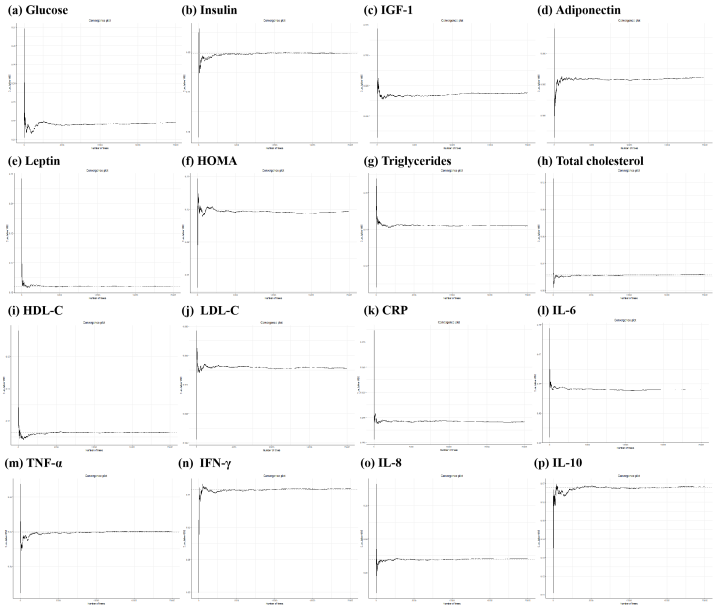


Fig. S20. Metaforest convergence plot with exercise prescription moderator variables. Each line represents one bootstrap iteration of the MetaForest model, plotting out-of-bag (OOB) prediction error across trees. A stable convergence is indicated when OOB error stabilizes with increasing tree number, suggesting reliable model performance.


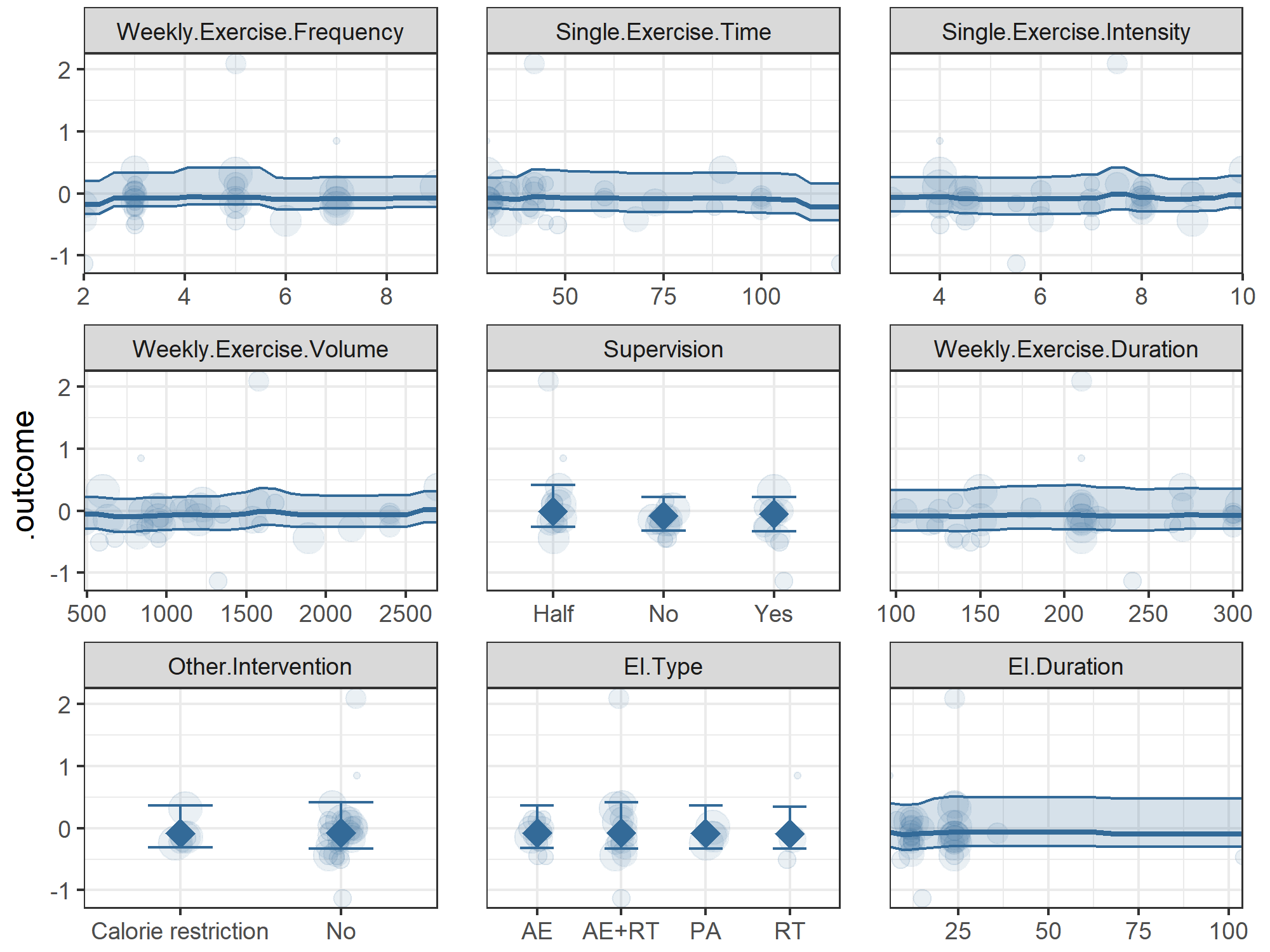


Fig. S21. Partial dependence plot (exercise prescription moderator variables of Glucose). Each panel shows the estimated partial dependence of the outcome on a specific moderator variable in the MetaForest model. For categorical moderators (e.g., Supervision, Other.Intervention, EI.Type), the plot displays the estimated mean effect and variability for each group. For continuous variables, each panel displays a smoothed curve showing how the predicted outcome changes across the range of the moderator, after averaging out the effects of other variables in the MetaForest model. Nonlinear patterns (e.g., U-shaped or threshold effects) may indicate optimal ranges of intervention.


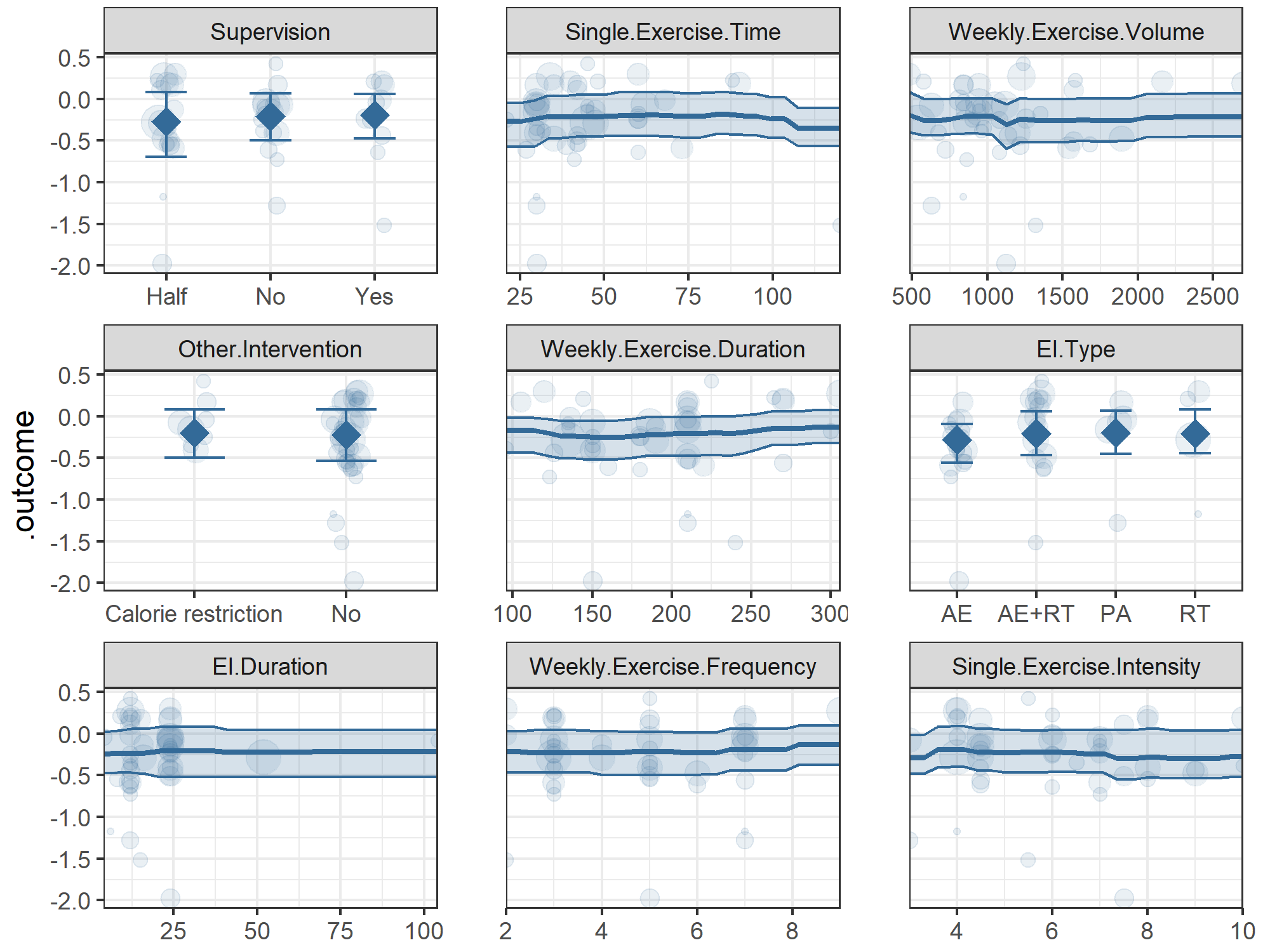


Fig. S22. Partial dependence plot (exercise prescription moderator variables of Insulin)


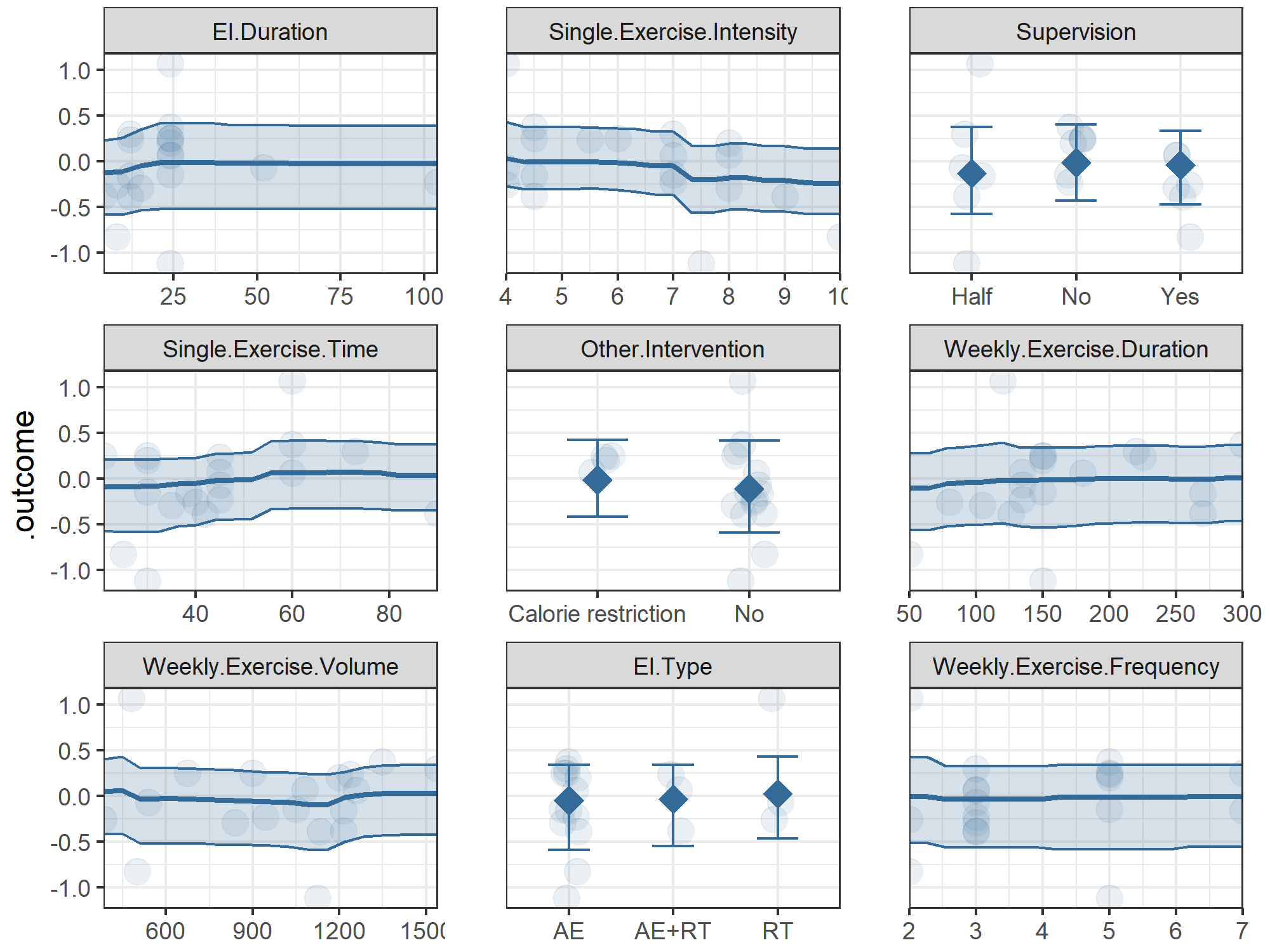


Fig. S23. Partial dependence plot (exercise prescription moderator variables of IGF-1)


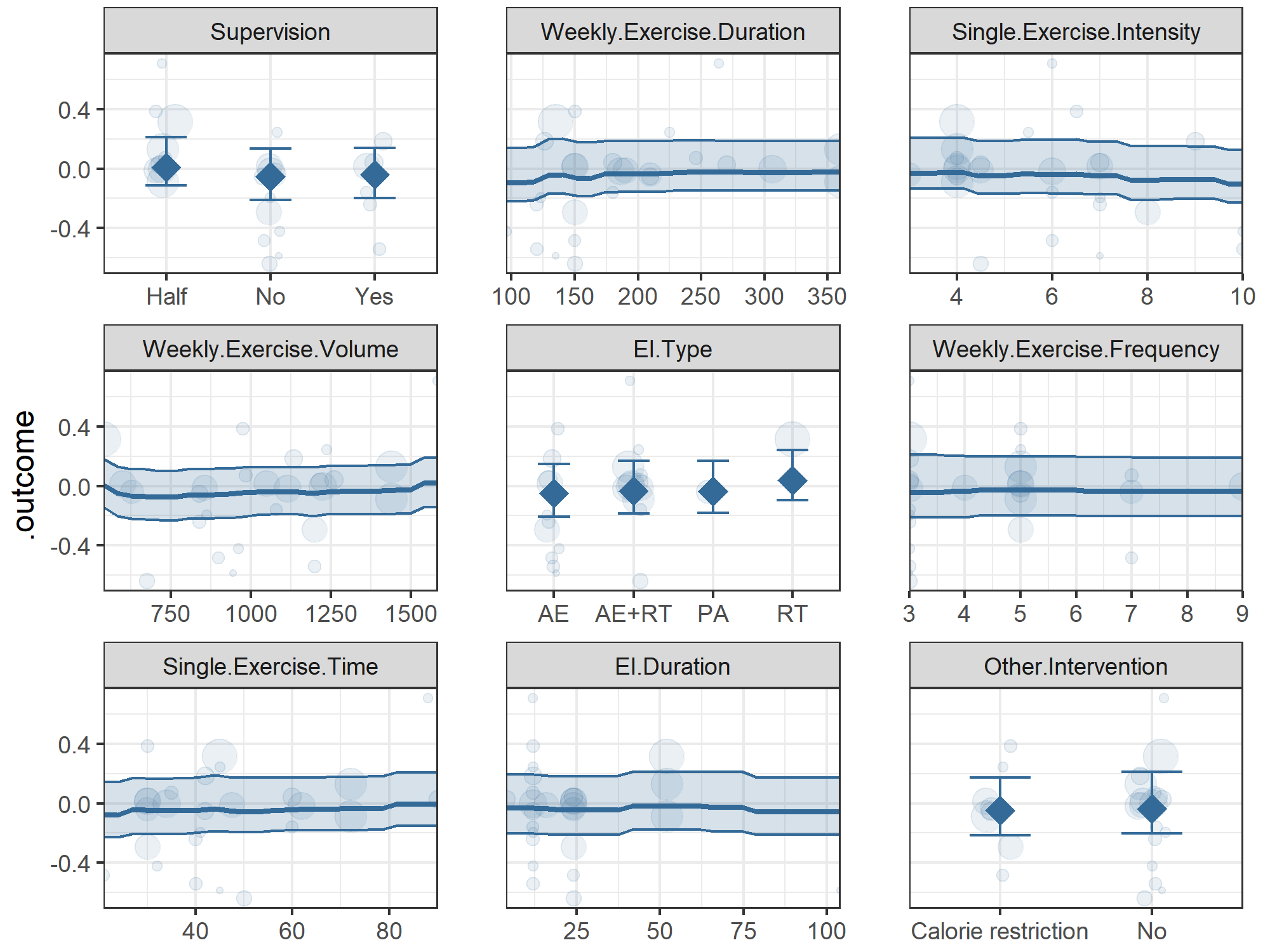


Fig. S24. Partial dependence plot (exercise prescription moderator variables of Adiponectin)


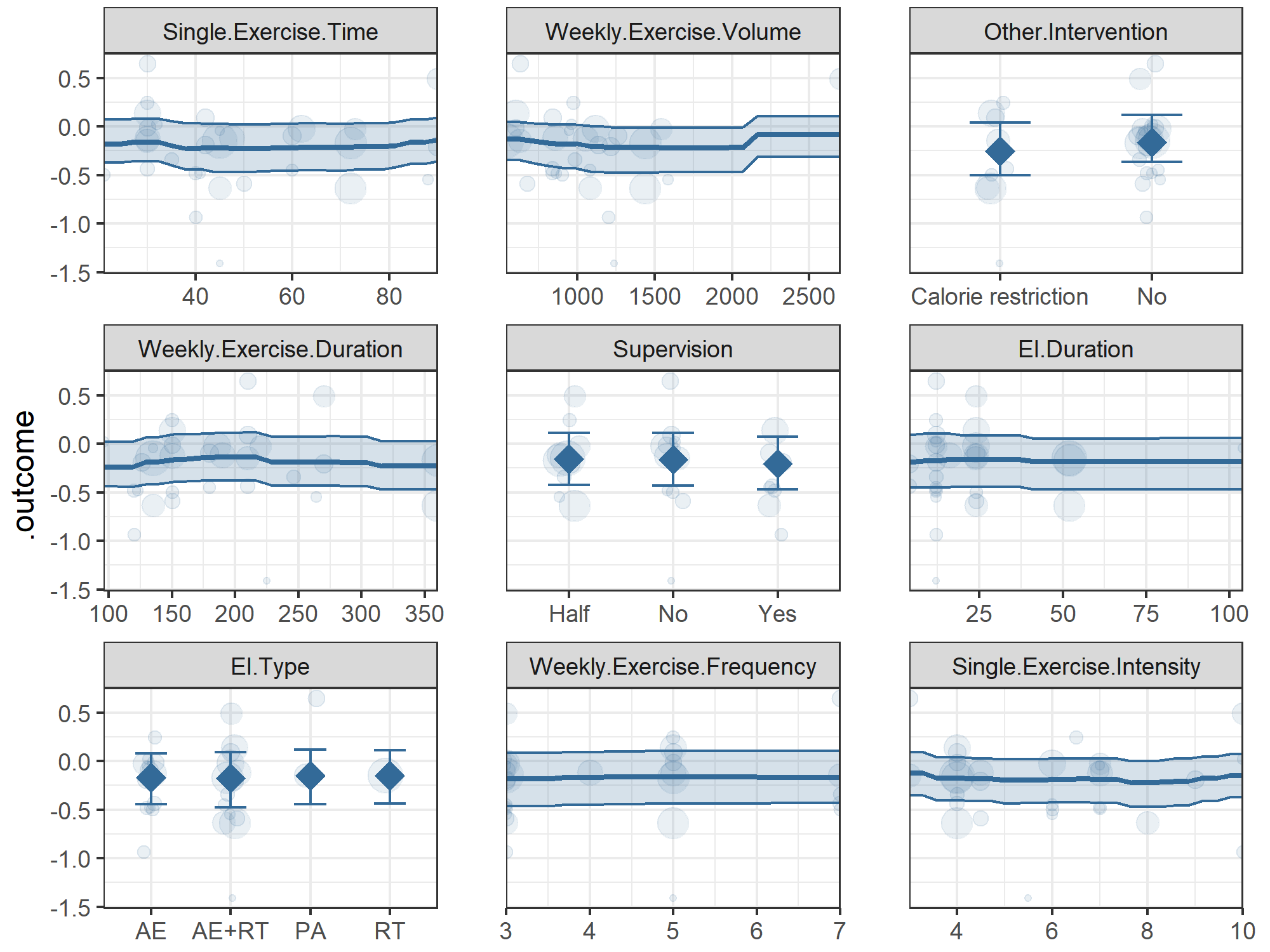


Fig. S25. Partial dependence plot (exercise prescription moderator variables of Leptin)


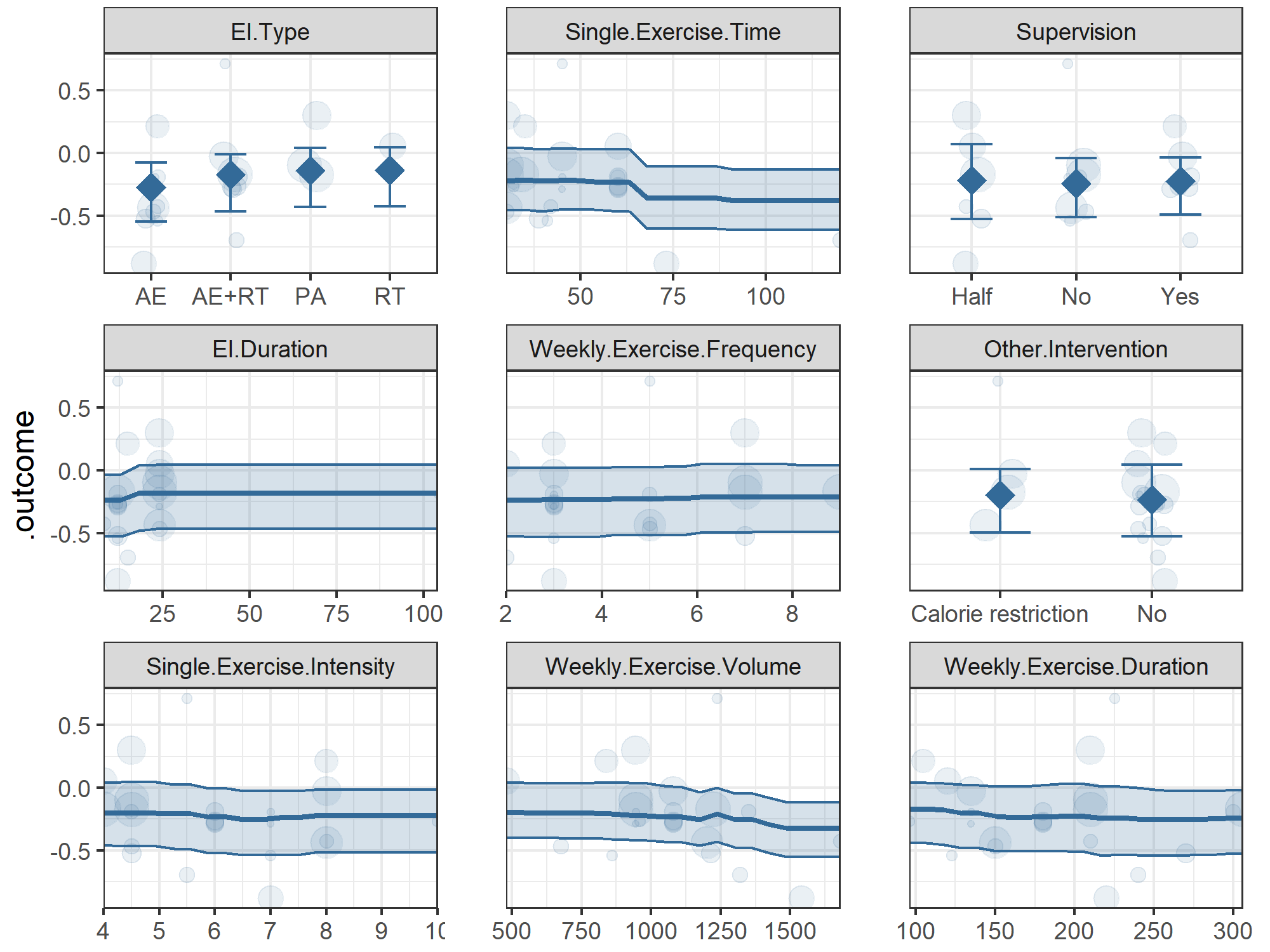


Fig. S26. Partial dependence plot (exercise prescription moderator variables of HOMA index)


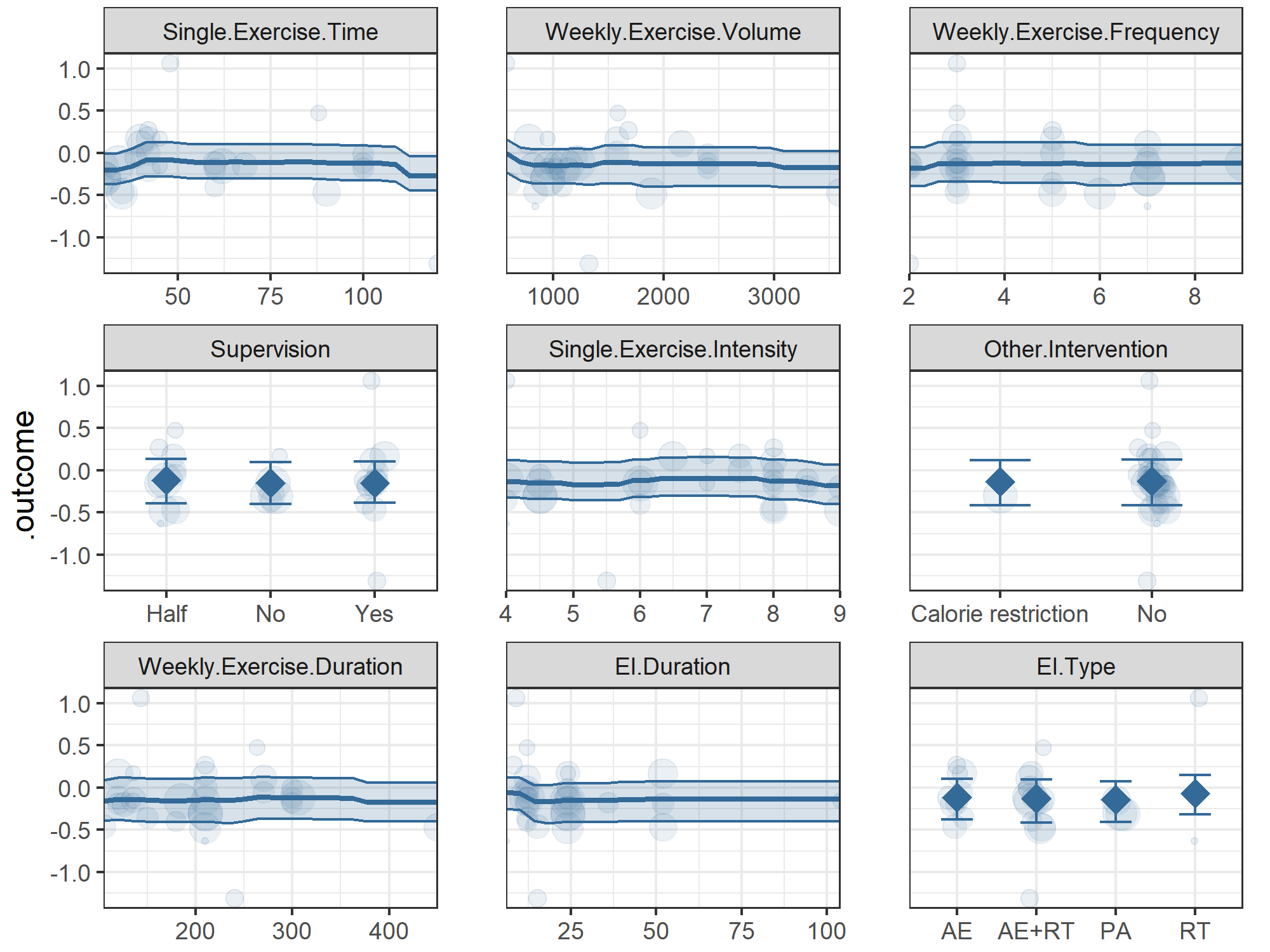


Fig. S27. Partial dependence plot (exercise prescription moderator variables of Triglycerides)


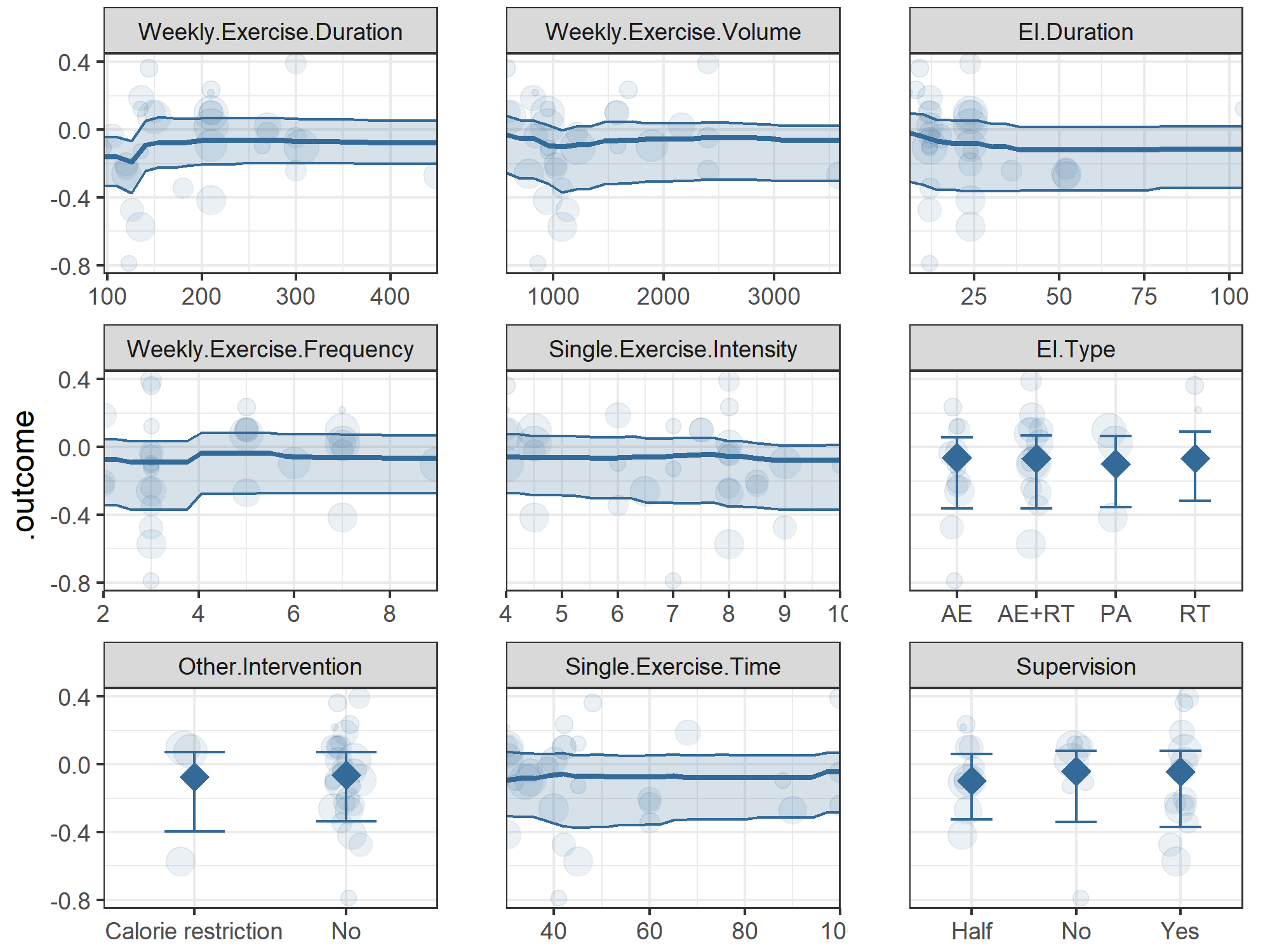


Fig. S28. Partial dependence plot (exercise prescription moderator variables of Total cholesterol)


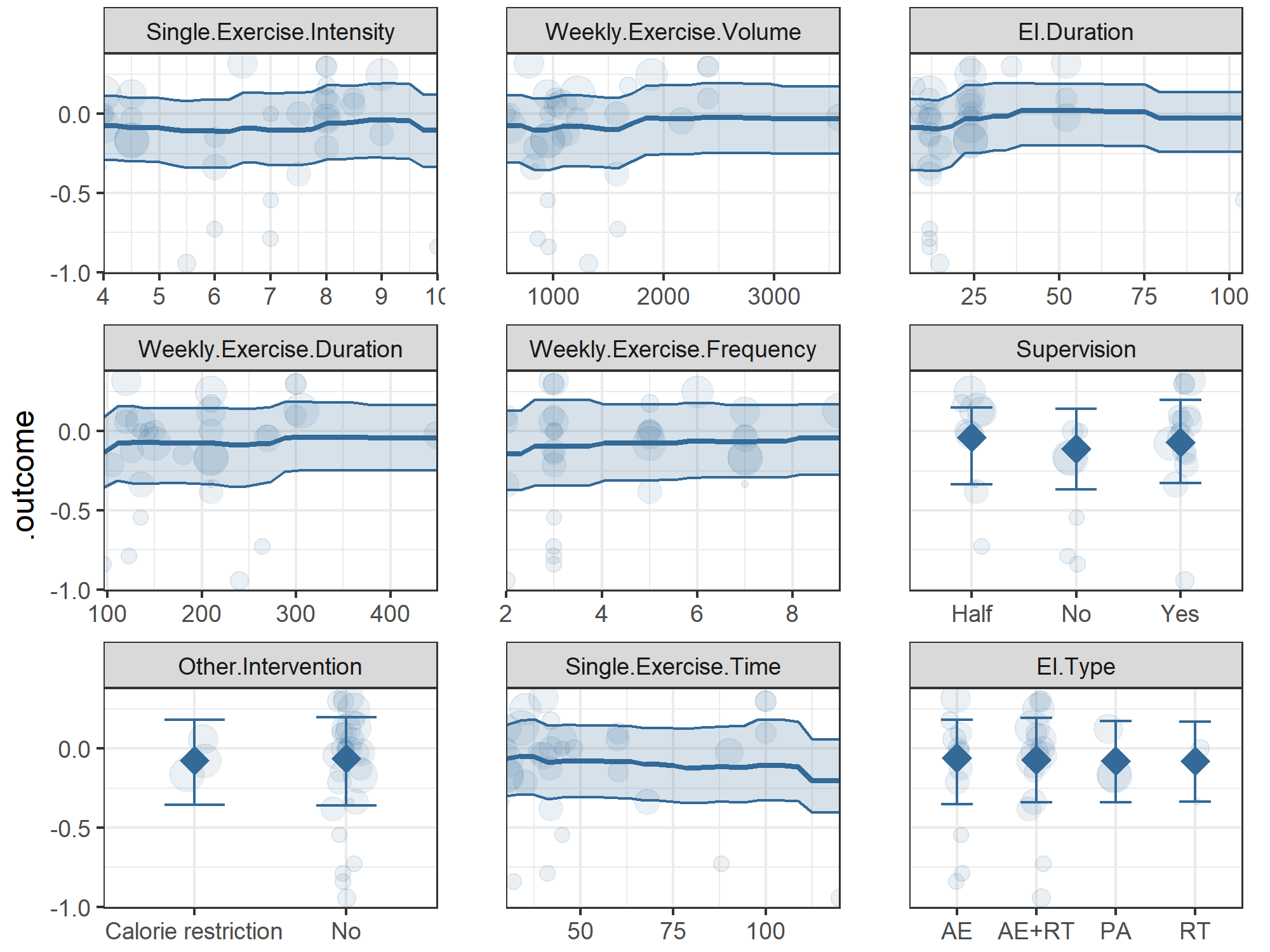


Fig. S29. Partial dependence plot (exercise prescription moderator variables of HDL-C)


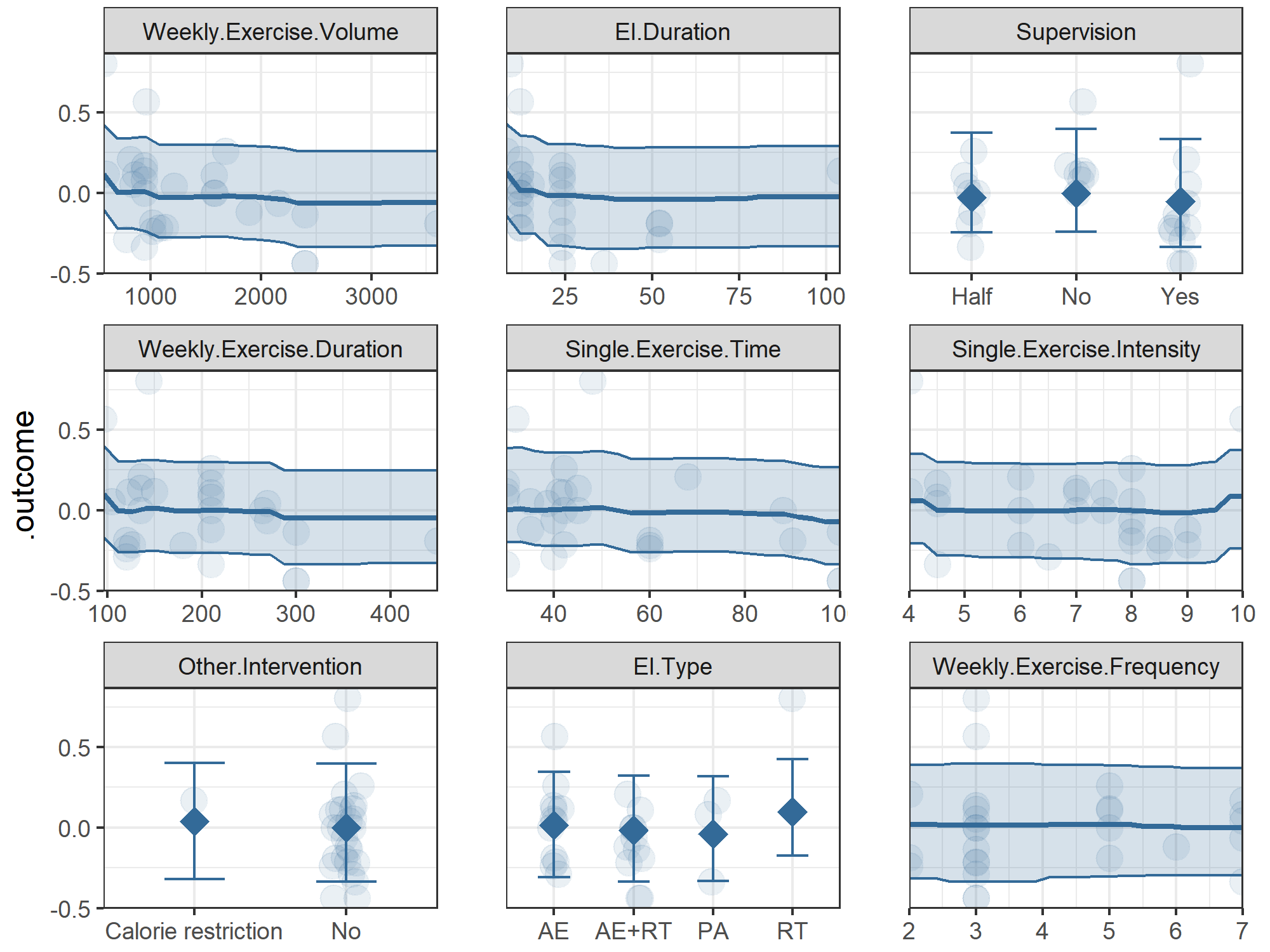


Fig. S30. Partial dependence plot (exercise prescription moderator variables of LDL-C)


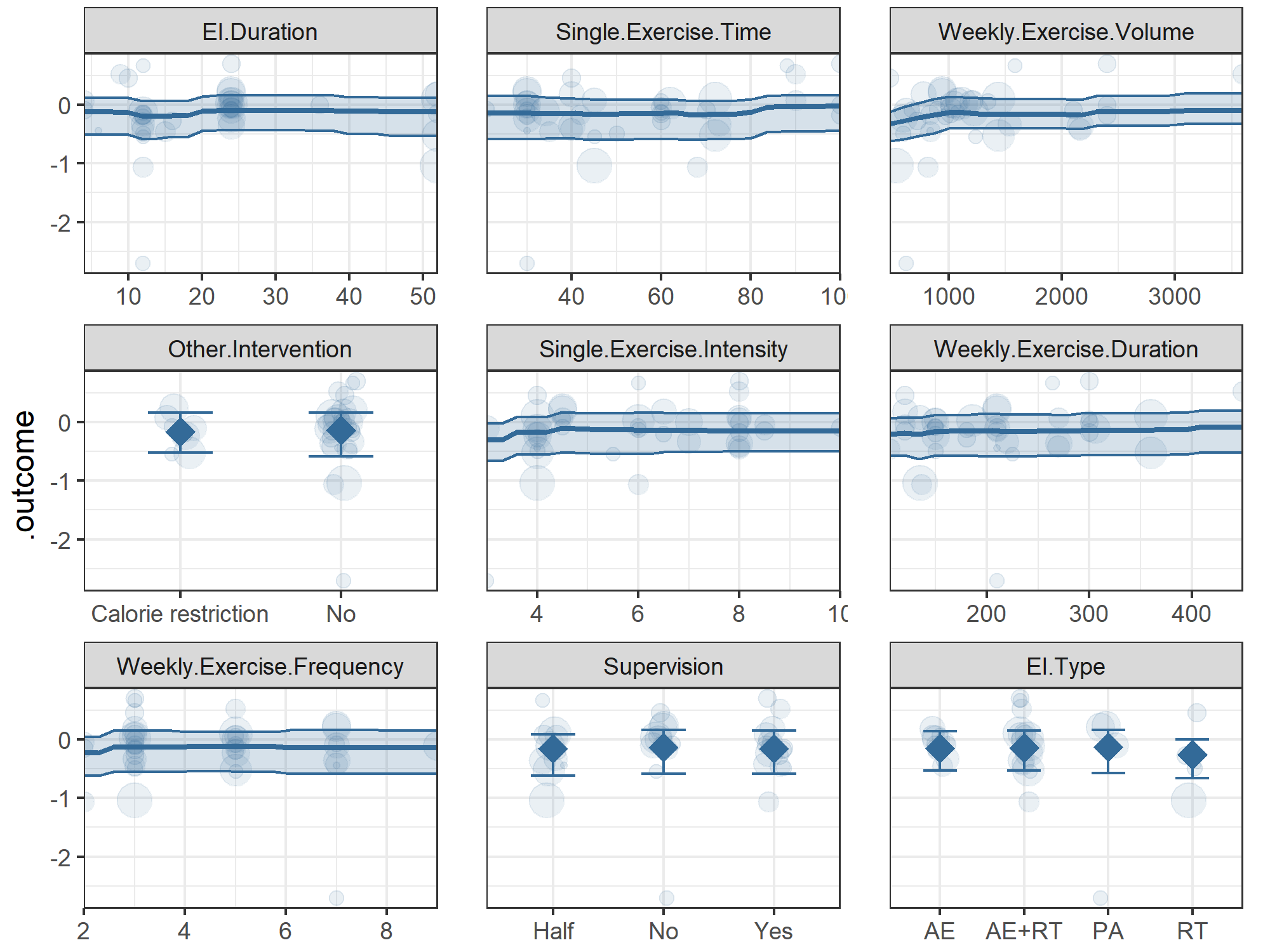


Fig. S31. Partial dependence plot (exercise prescription moderator variables of CRP)


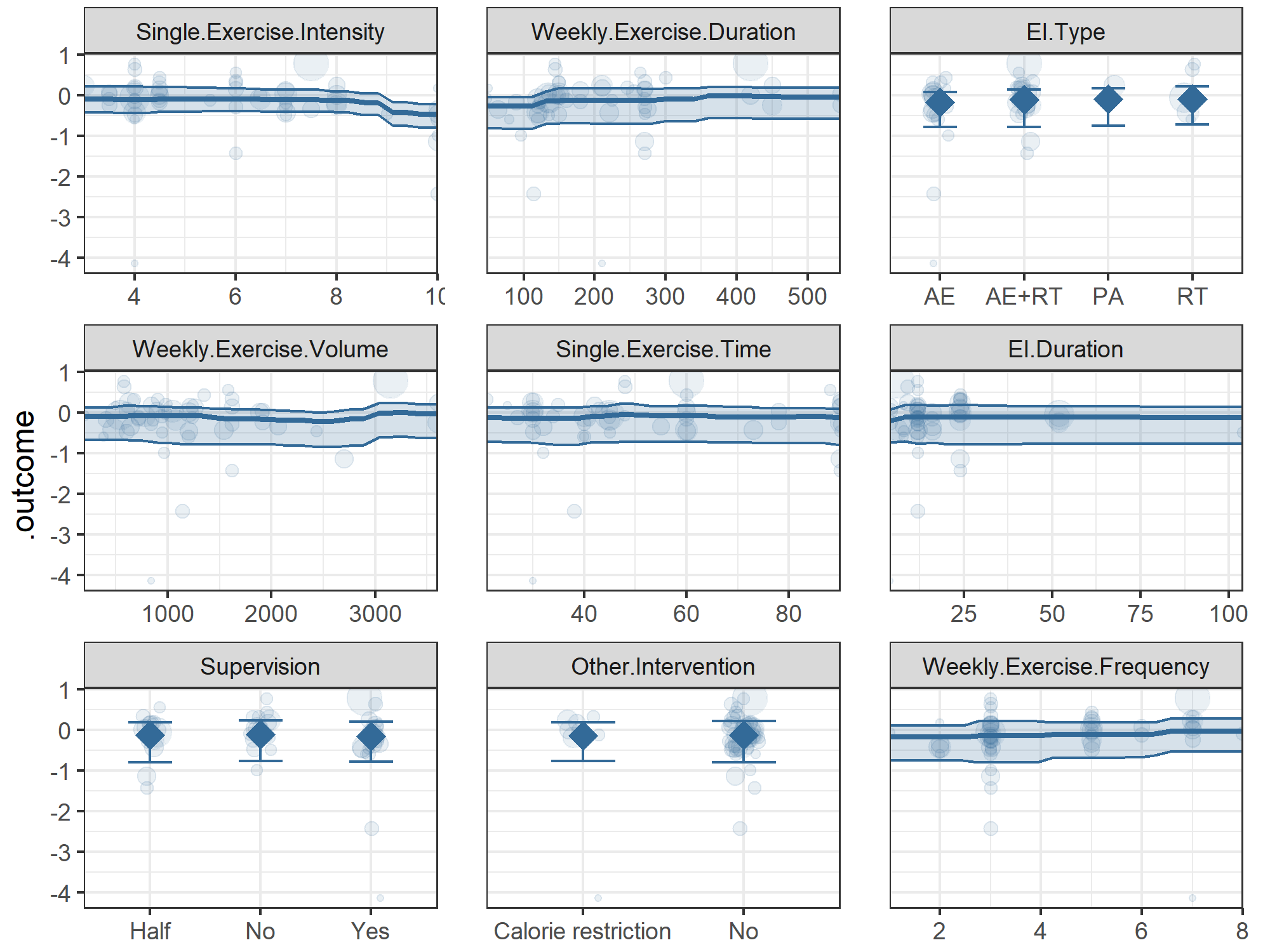


Fig. S32. Partial dependence plot (exercise prescription moderator variables of IL-6)


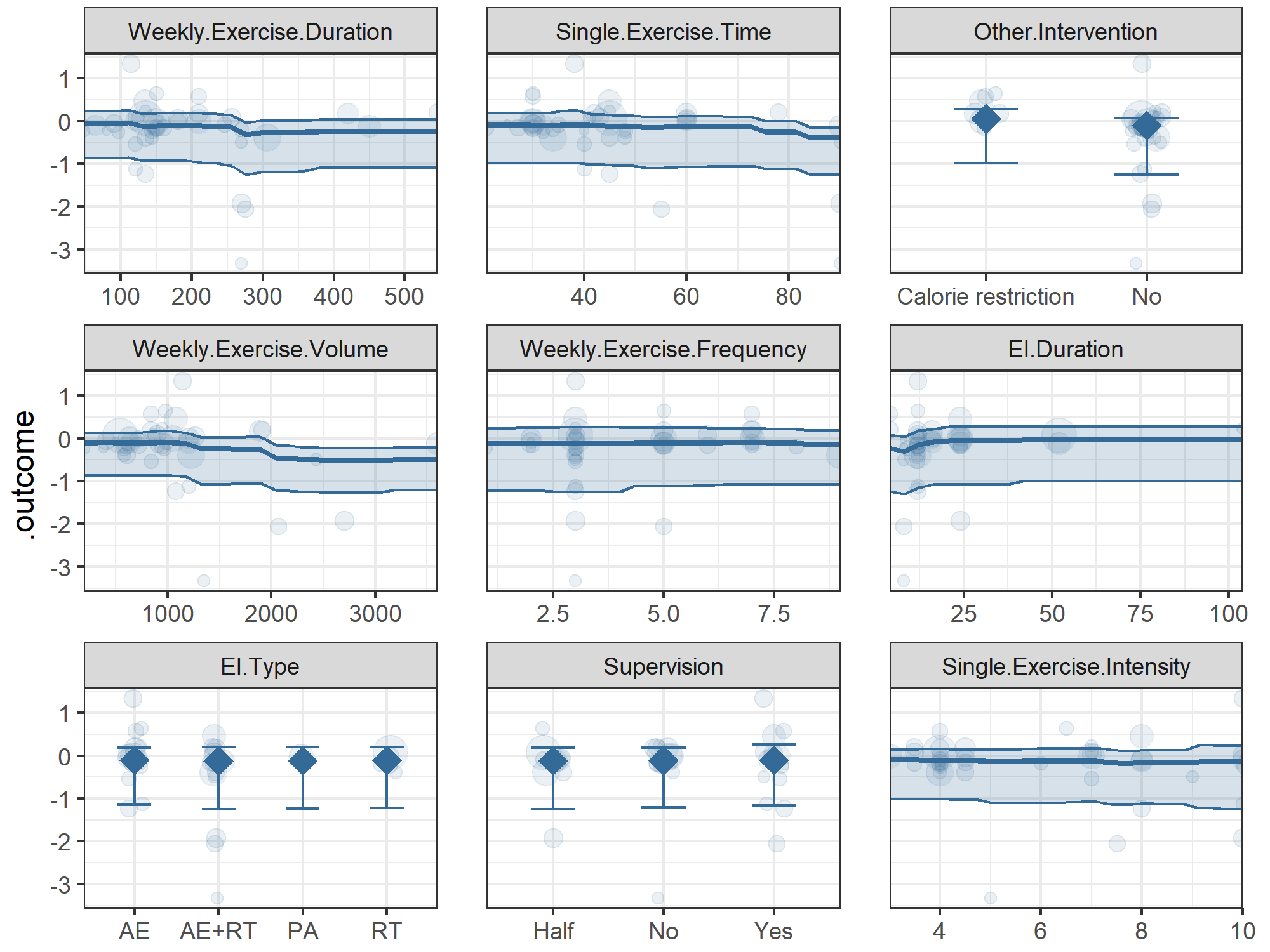


Fig. S33. Partial dependence plot (exercise prescription moderator variables of TNF-α)


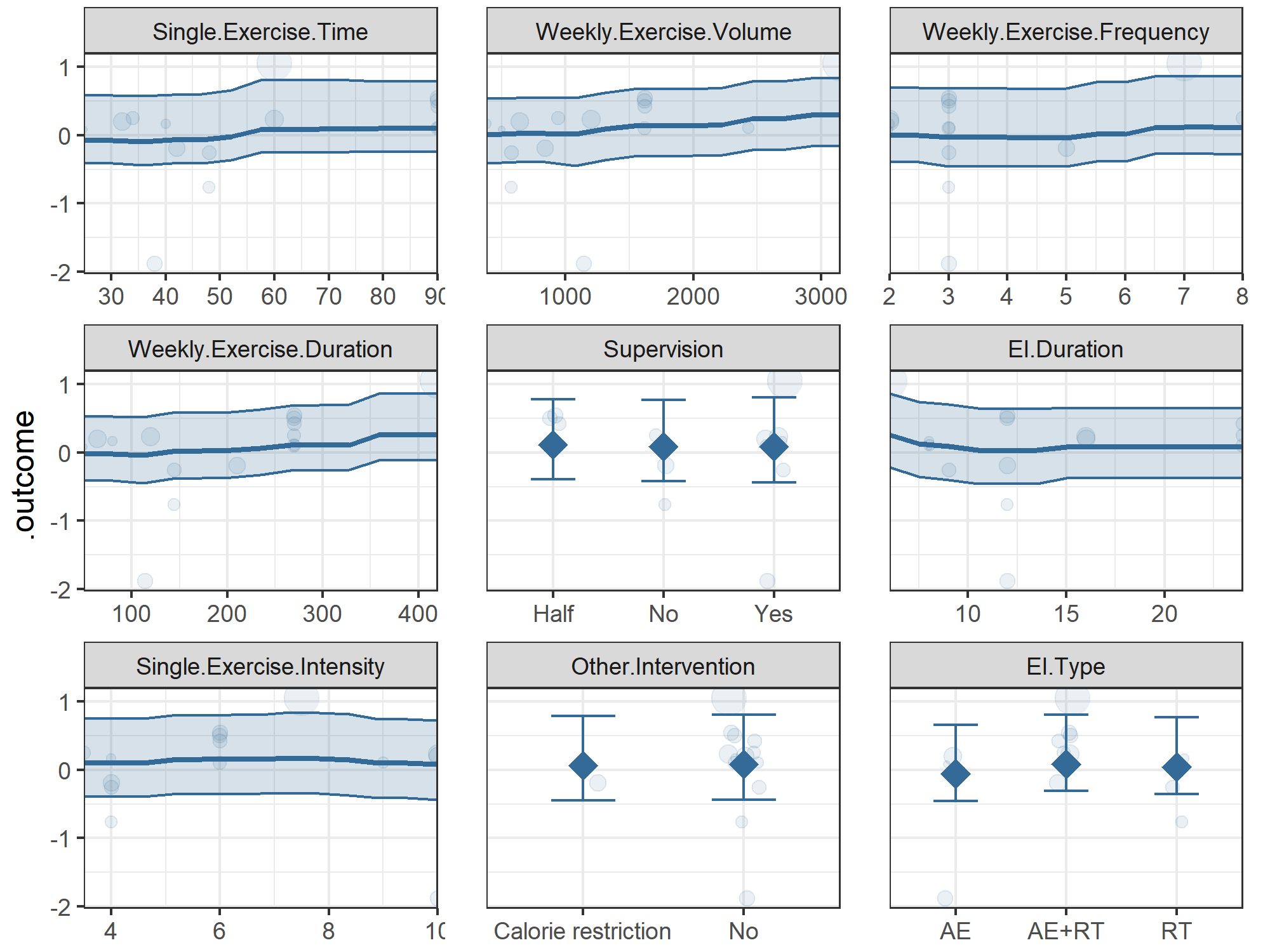


Fig. S34. Partial dependence plot (exercise prescription moderator variables of IFN-γ)


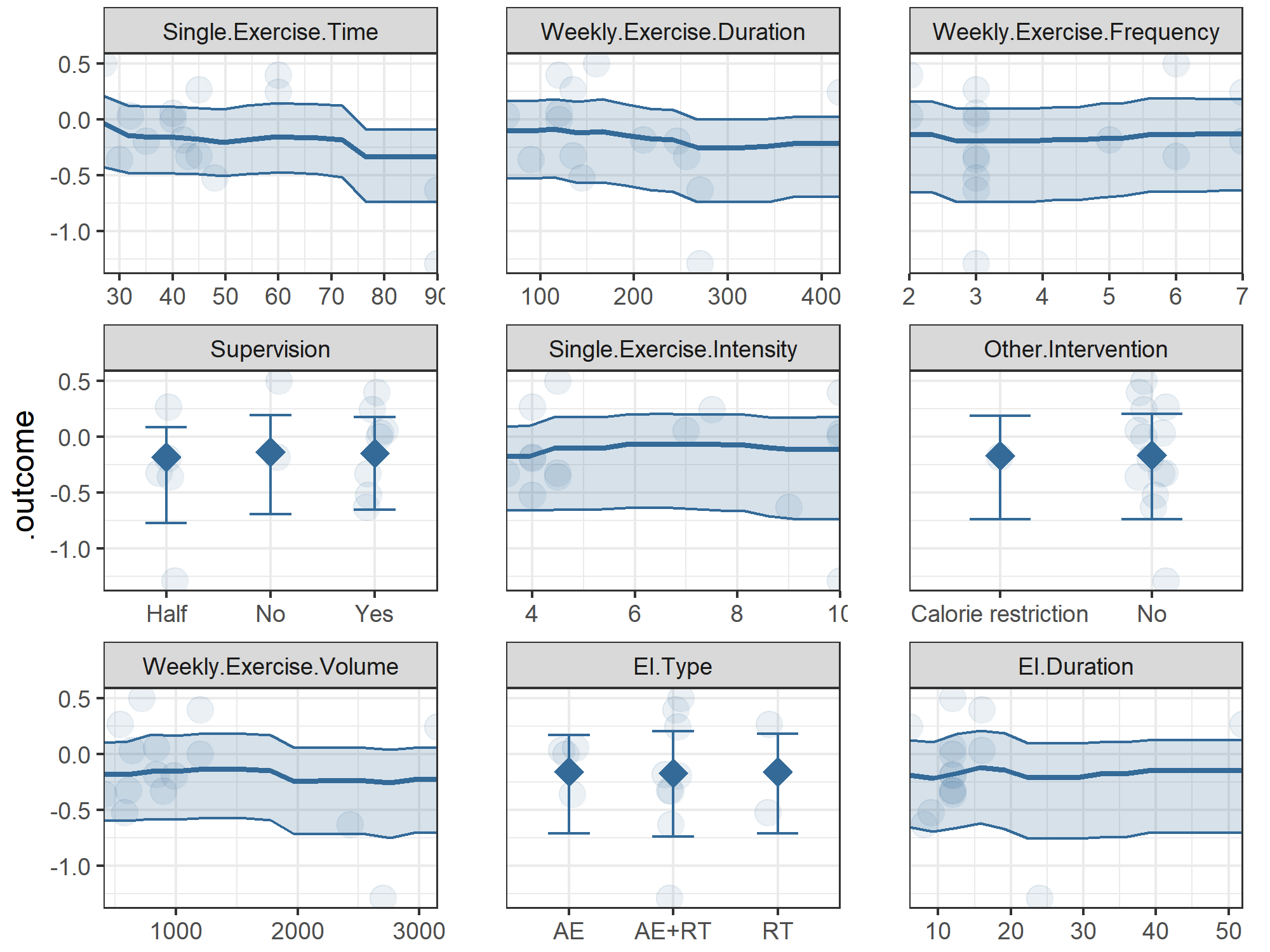


Fig. S35. Partial dependence plot (exercise prescription moderator variables of IL-8)


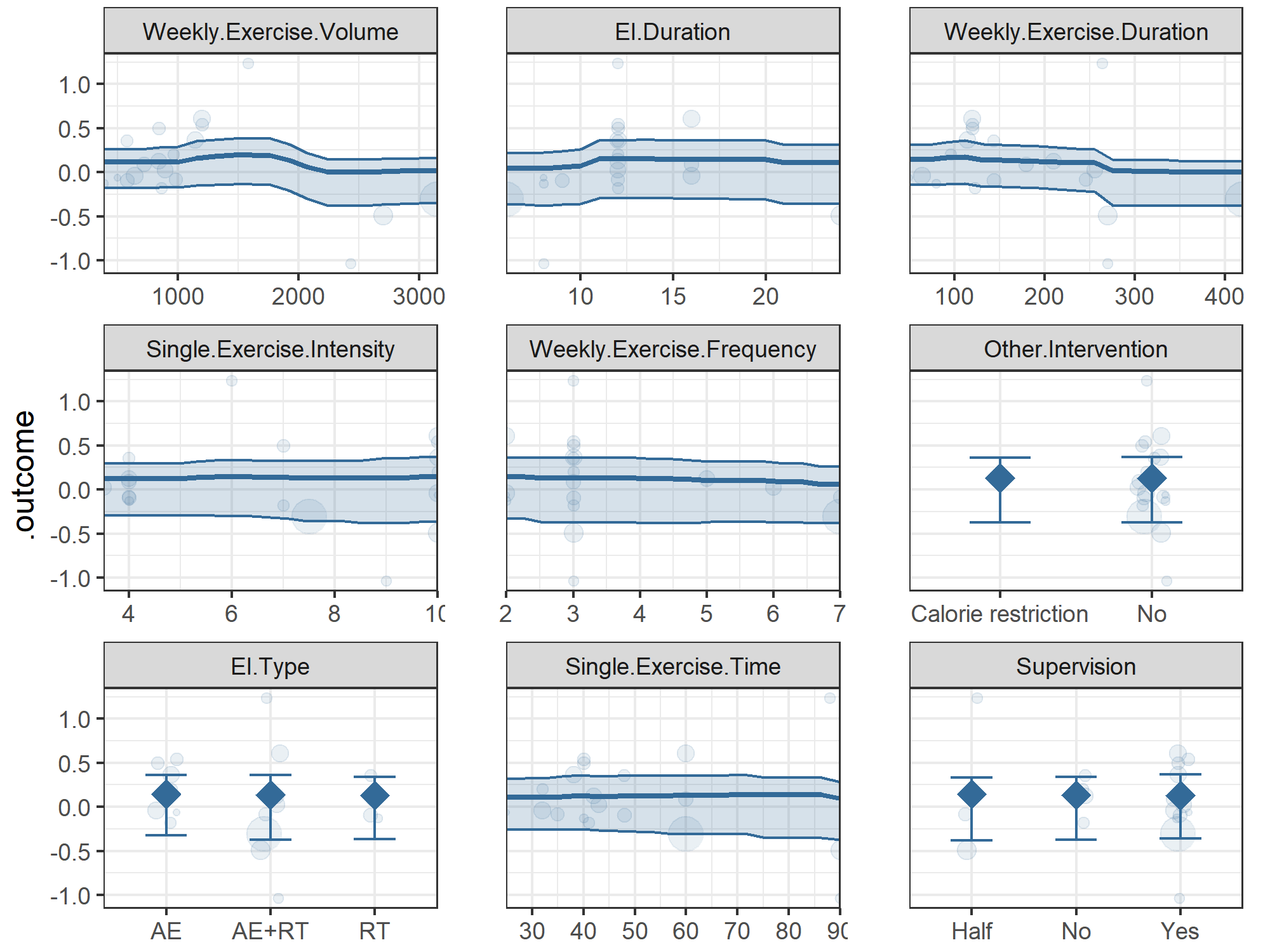


Fig. S36. Partial dependence plot (exercise prescription moderator variables of IL-10)

Table S8. Parameters of the MetaForest regression model with background moderating variables

| ID | R²_OOB_ | τ² | Feature Weights | Mtry | min.node.size | R²_CV_ | R²_CV_ SD | RMSE | RMSE SD | MAE | MAE SD |
| --- | --- | --- | --- | --- | --- | --- | --- | --- | --- | --- | --- |
| Glucose | -0.10 | 0.12 | fixed | 2.00 | 4.00 | 0.09 | 0.10 | 0.50 | 0.17 | 0.32 | 0.09 |
| Insulin | -0.26 | 0.19 | fixed | 2.00 | 4.00 | 0.08 | 0.08 | 0.53 | 0.11 | 0.39 | 0.07 |
| IGF-1 | -0.45 | 0.29 | unif | 2.00 | 4.00 | 0.15 | 0.20 | 0.54 | 0.15 | 0.41 | 0.10 |
| Adiponectin | -0.29 | 0.03 | fixed | 2.00 | 4.00 | 0.10 | 0.12 | 0.32 | 0.07 | 0.25 | 0.06 |
| Leptin | 0.03 | 0.06 | random | 2.00 | 4.00 | 0.09 | 0.12 | 0.41 | 0.09 | 0.32 | 0.07 |
| HOMA index | 0.05 | 0.02 | unif | 2.00 | 2.00 | 0.20 | 0.21 | 0.33 | 0.09 | 0.25 | 0.07 |
| Triglycerides | -0.08 | 0.01 | fixed | 2.00 | 4.00 | 0.07 | 0.09 | 0.40 | 0.13 | 0.28 | 0.08 |
| Total cholesterol | -0.16 | 0.00 | fixed | 2.00 | 4.00 | 0.05 | 0.09 | 0.28 | 0.05 | 0.22 | 0.04 |
| HDL-C | 0.00 | 0.00 | fixed | 2.00 | 3.00 | 0.14 | 0.15 | 0.32 | 0.07 | 0.25 | 0.05 |
| LDL-C | 0.16 | 0.00 | unif | 2.00 | 2.00 | 0.26 | 0.20 | 0.25 | 0.08 | 0.20 | 0.05 |
| CRP | -0.06 | 0.16 | fixed | 2.00 | 4.00 | 0.08 | 0.12 | 0.52 | 0.17 | 0.36 | 0.08 |
| IL-6 | -0.16 | 0.33 | fixed | 2.00 | 4.00 | 0.05 | 0.06 | 0.78 | 0.30 | 0.48 | 0.12 |
| TNF-α | -0.18 | 0.55 | fixed | 2.00 | 4.00 | 0.05 | 0.07 | 0.84 | 0.23 | 0.55 | 0.14 |
| IFN-γ | -0.31 | 0.56 | unif | 2.00 | 2.00 | 0.18 | 0.22 | 0.71 | 0.32 | 0.49 | 0.20 |
| IL-8 | 0.28 | 0.09 | fixed | 2.00 | 4.00 | 0.39 | 0.26 | 0.37 | 0.13 | 0.30 | 0.11 |
| IL-10 | -0.16 | 0.13 | fixed | 2.00 | 4.00 | 0.17 | 0.21 | 0.49 | 0.14 | 0.38 | 0.11 |


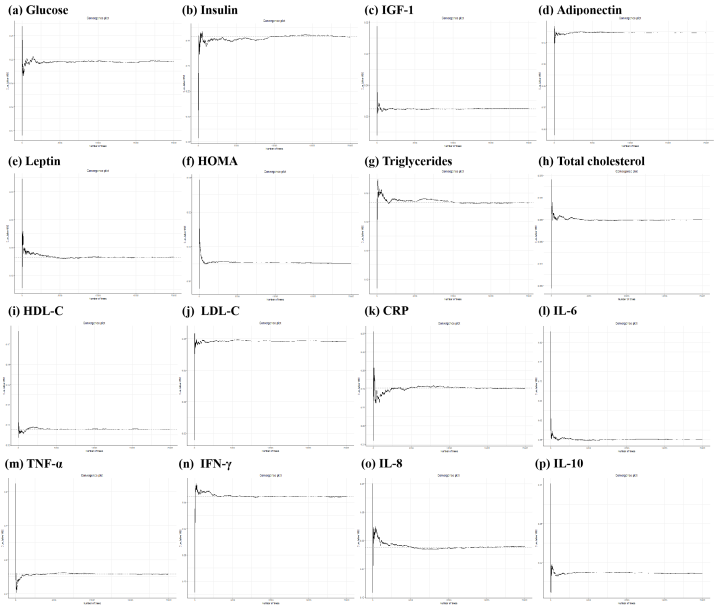


Fig. S37. Metaforest convergence plot with background moderating variables


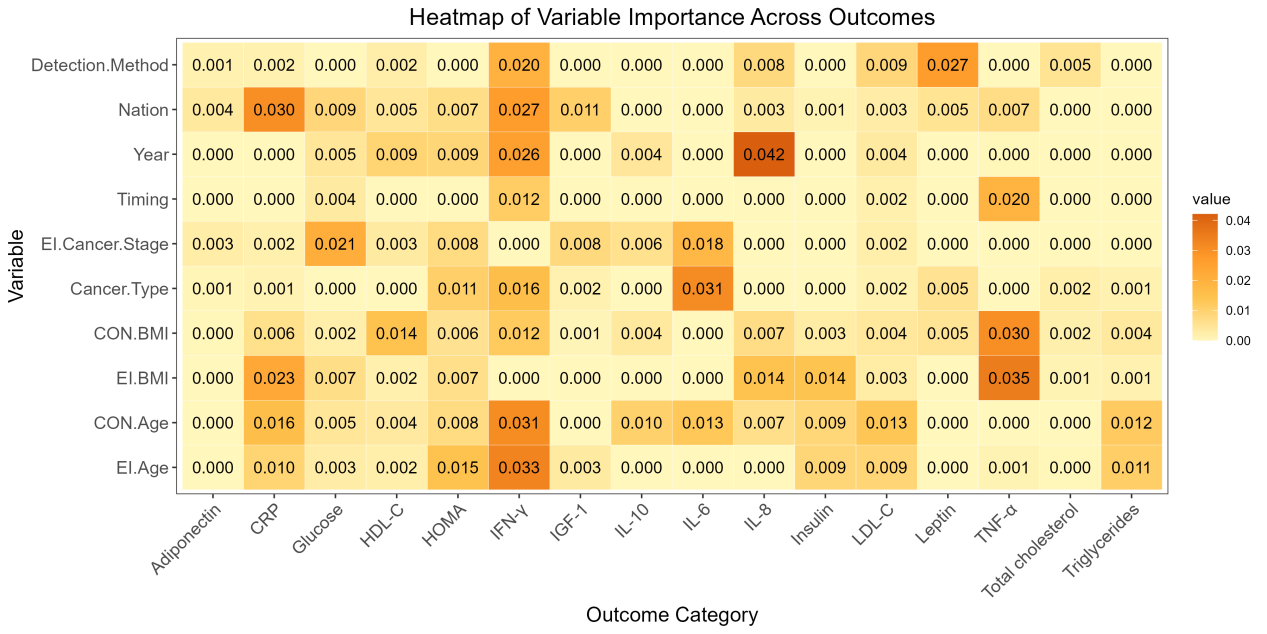


Fig. S38. Heat map of the importance of background moderator variables


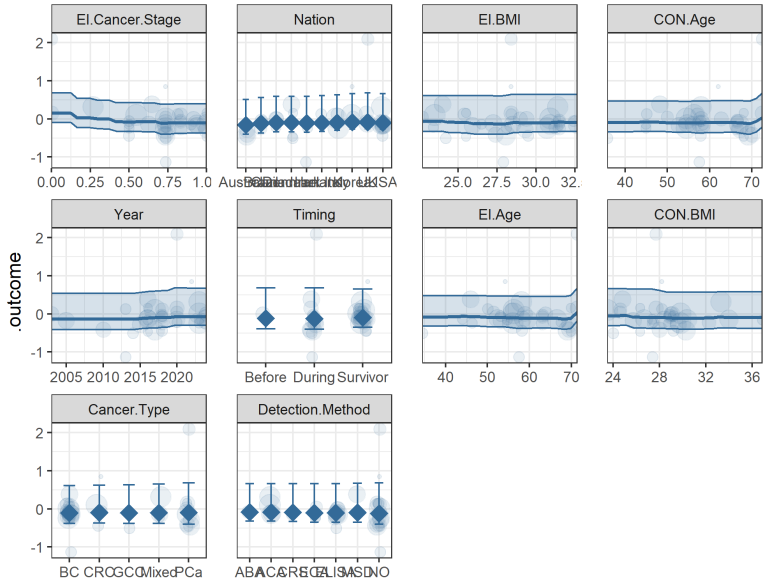


Fig. S39. Partial dependence plot (background moderator variables of Glucose)


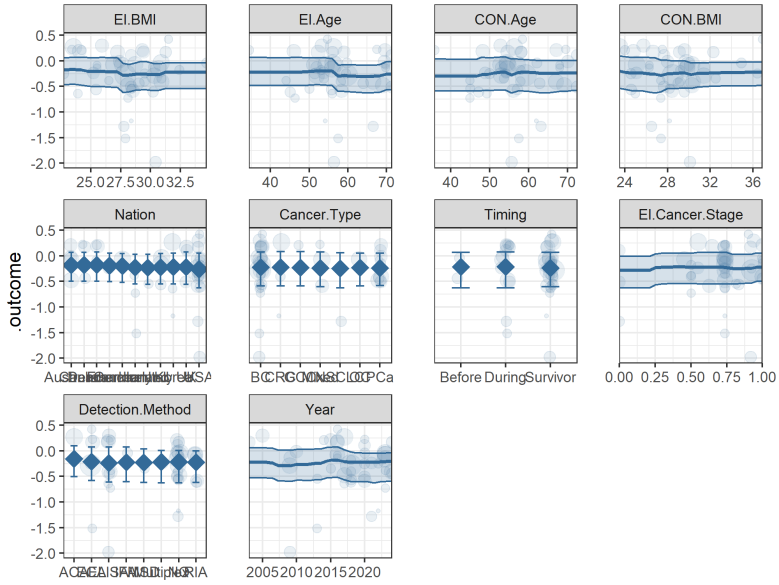


Fig. S40. Partial dependence plot (background moderator variables of Insulin)


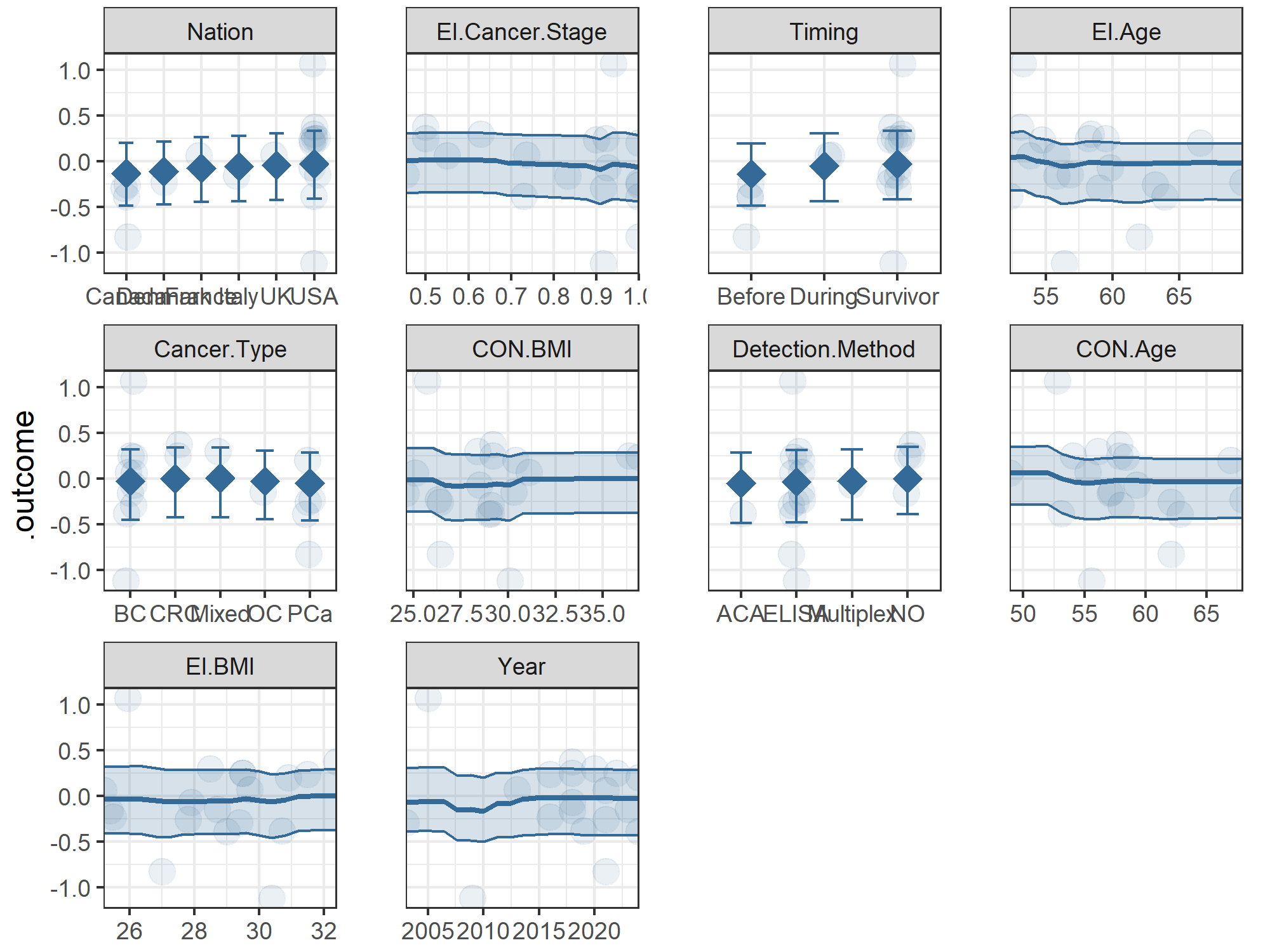


Fig. S41. Partial dependence plot (background moderator variables of IGF-1)


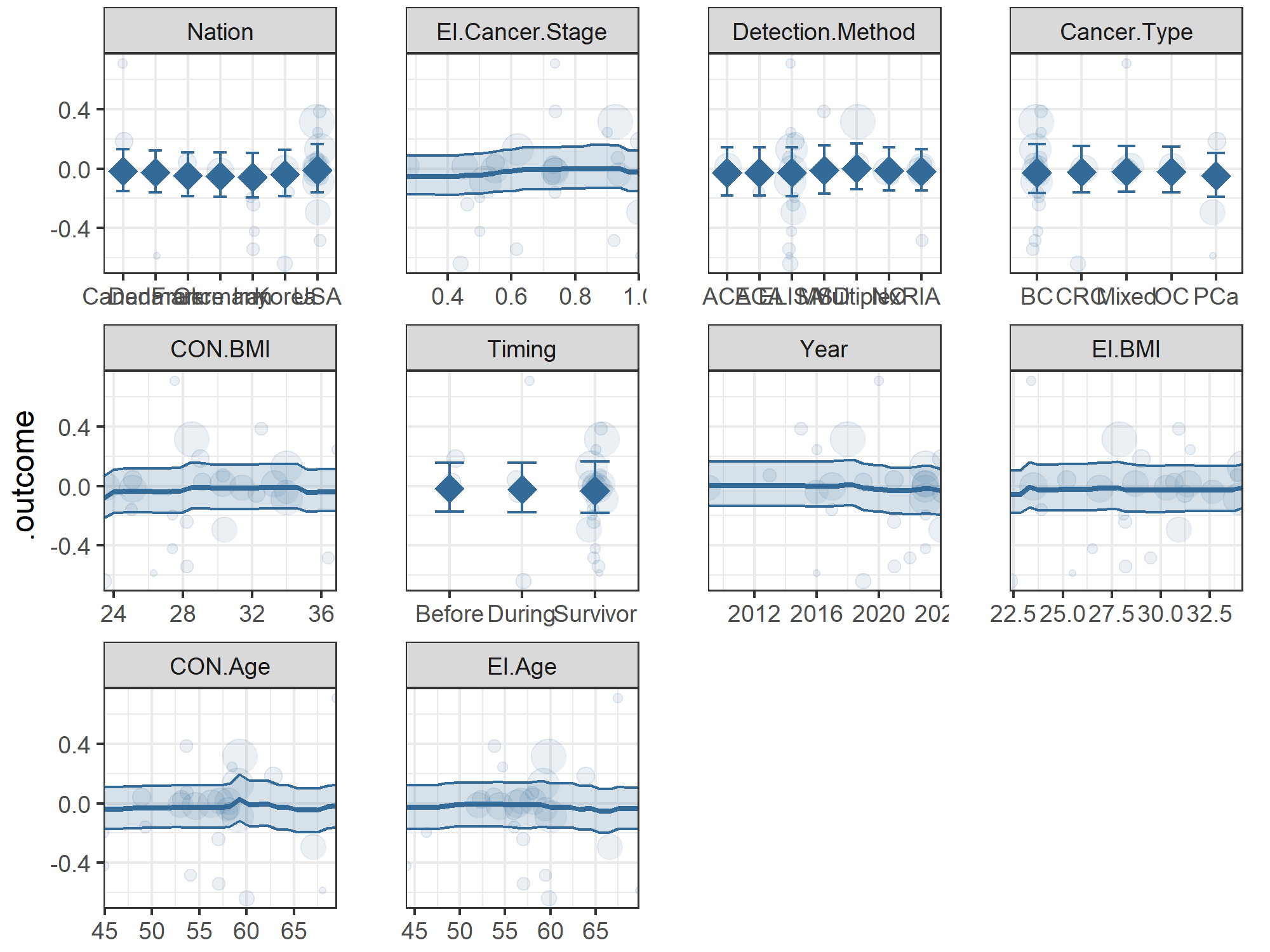


Fig. S42. Partial dependence plot (background moderator variables of Adiponectin)


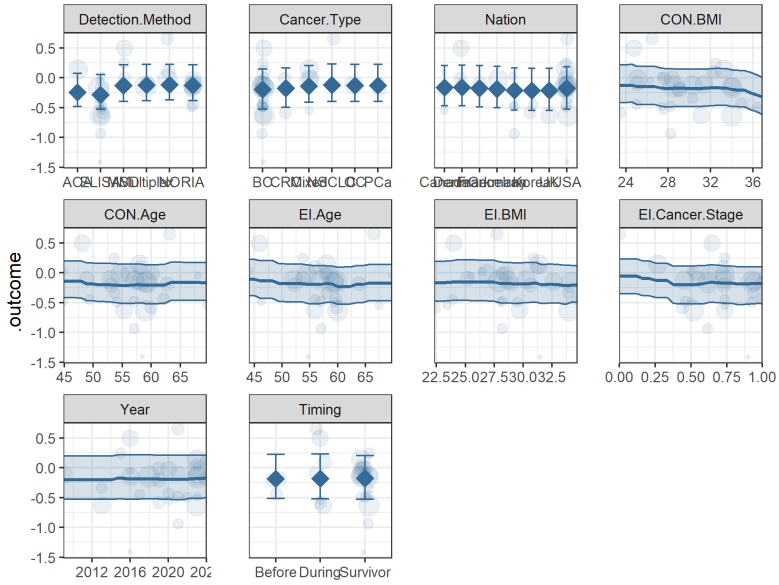


Fig. S43. Partial dependence plot (background moderator variables of Leptin)


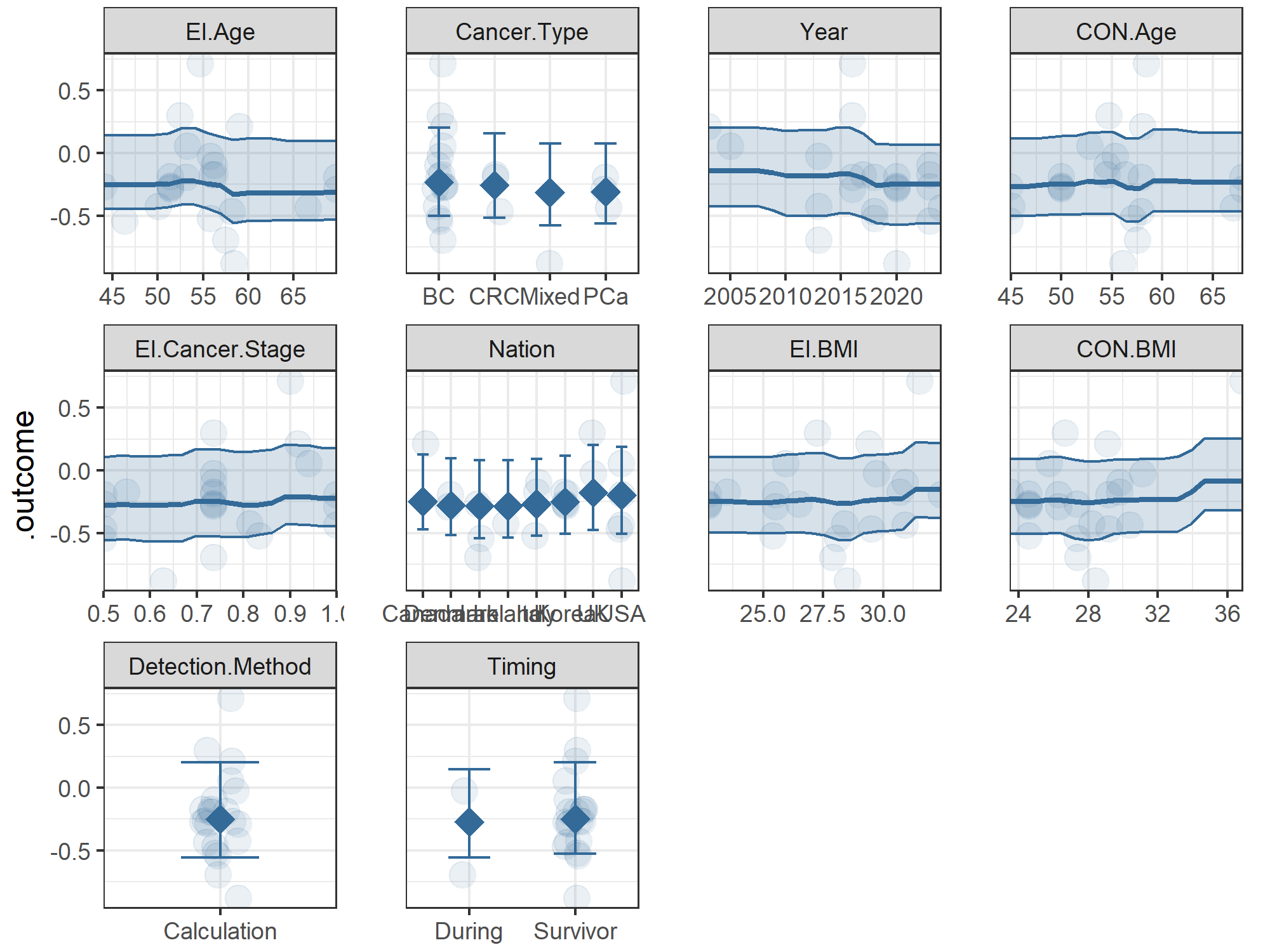


Fig. S44. Partial dependence plot (background moderator variables of HOMA index)


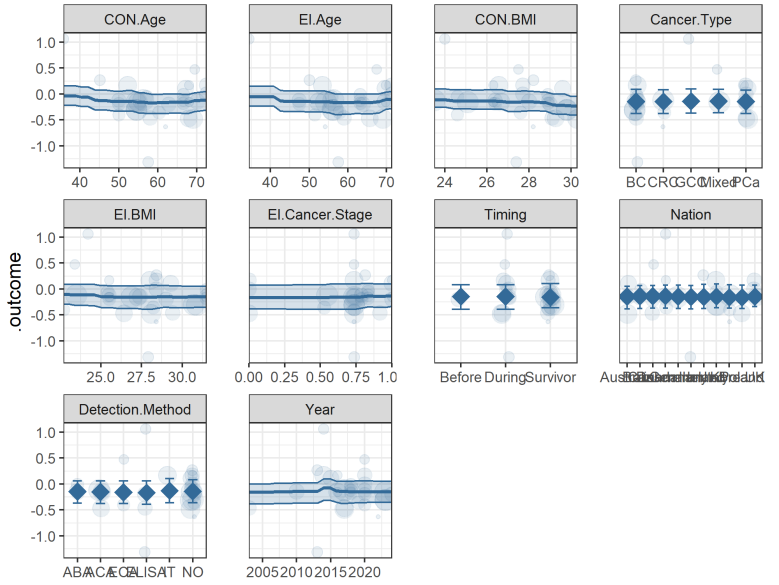


Fig. S45. Partial dependence plot (background moderator variables of Triglycerides)


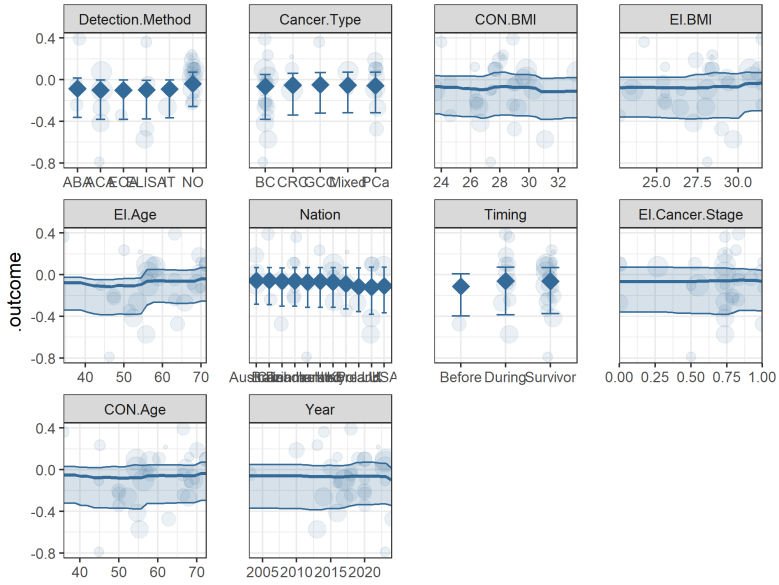


Fig. S46. Partial dependence plot (background moderator variables of Total cholesterol)


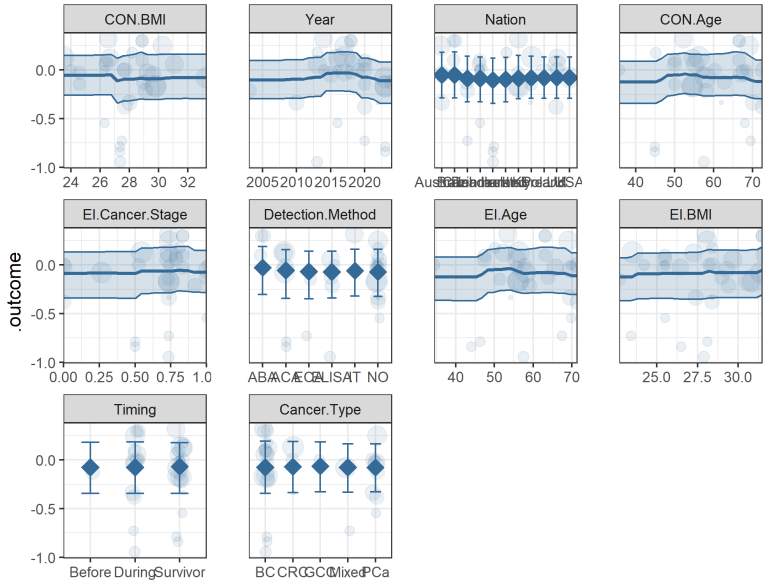


Fig. S47. Partial dependence plot (background moderator variables of HDL-C)


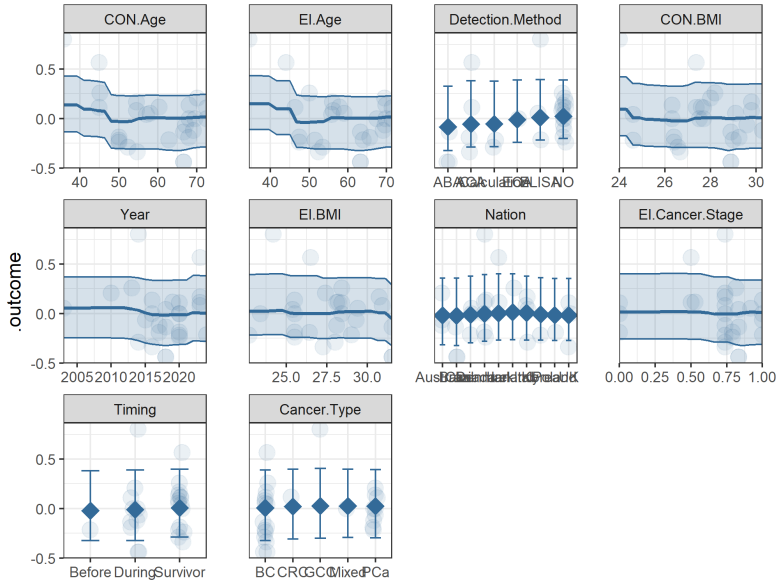


Fig. S48. Partial dependence plot (background moderator variables of LDL-C)


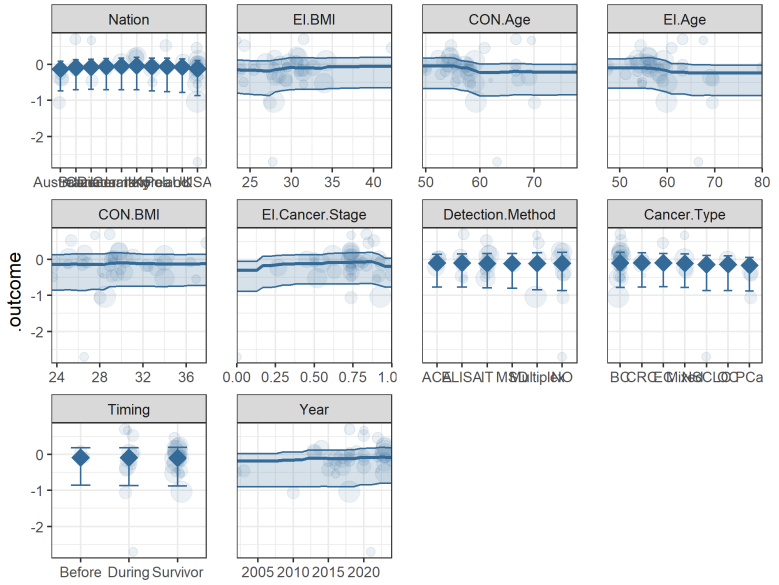


Fig. S49. Partial dependence plot (background moderator variables of CRP)


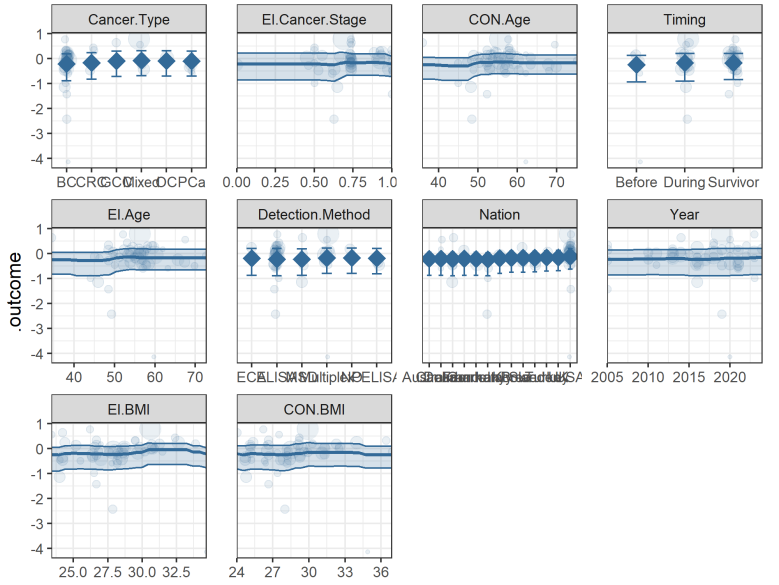


Fig. S50. Partial dependence plot (background moderator variables of IL-6)


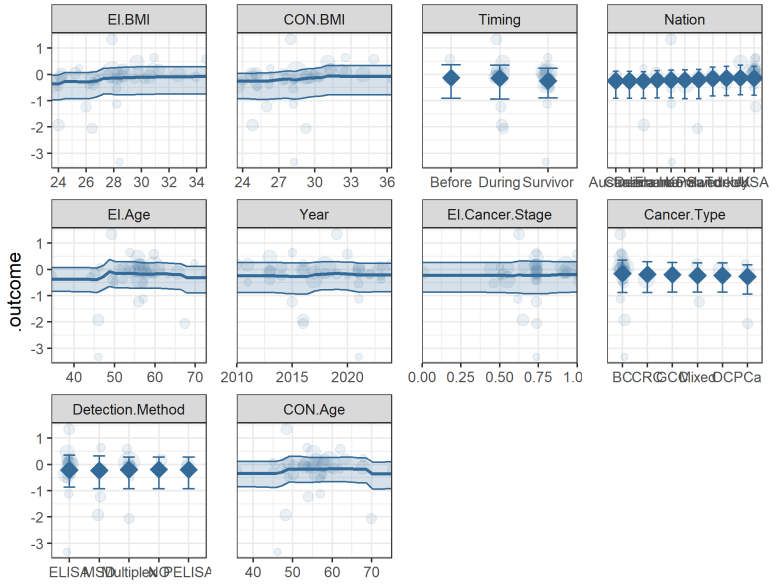


Fig. S51. Partial dependence plot (background moderator variables of TNF-α)


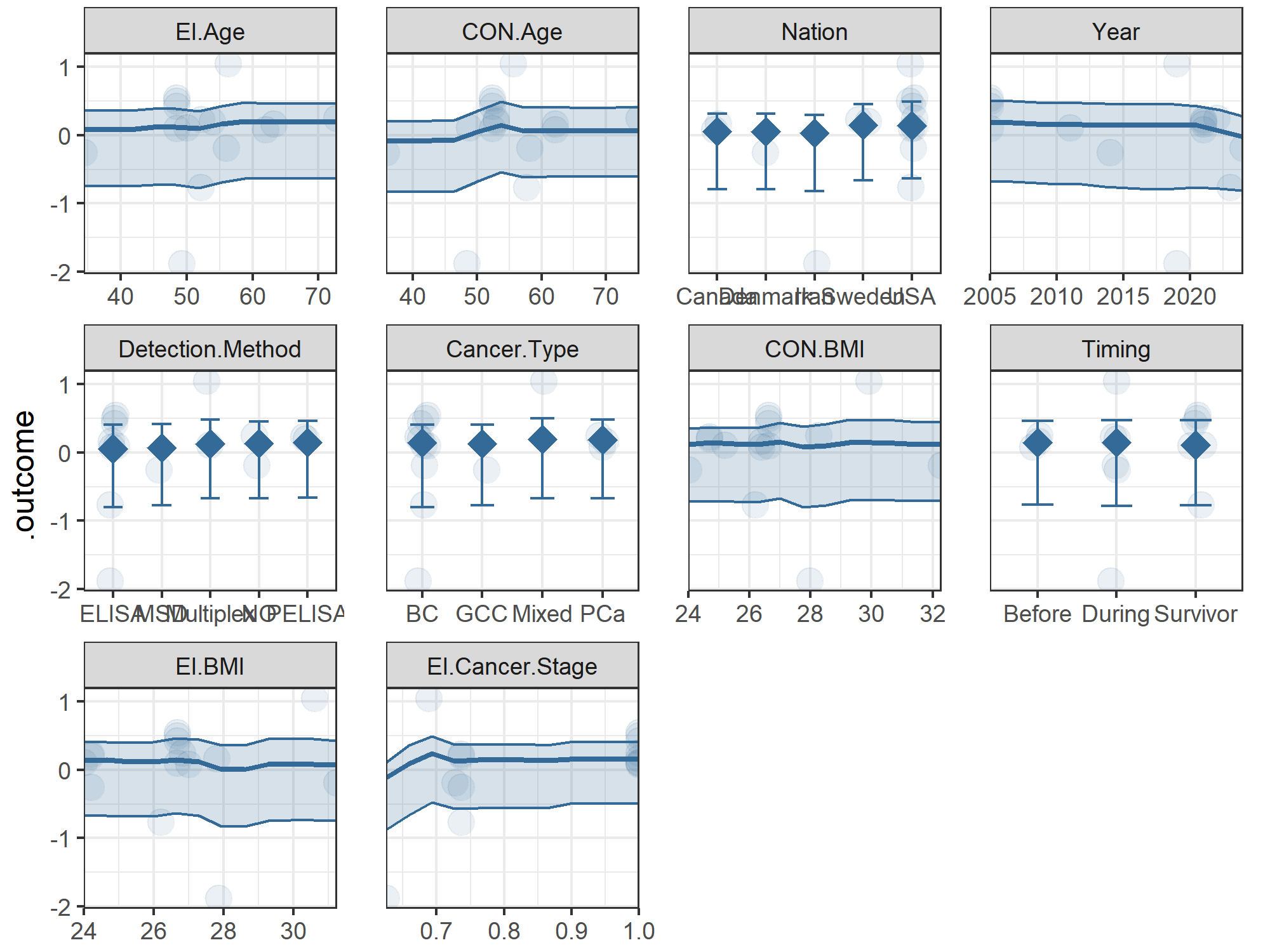


Fig. S52. Partial dependence plot (background moderator variables of IFN-γ)


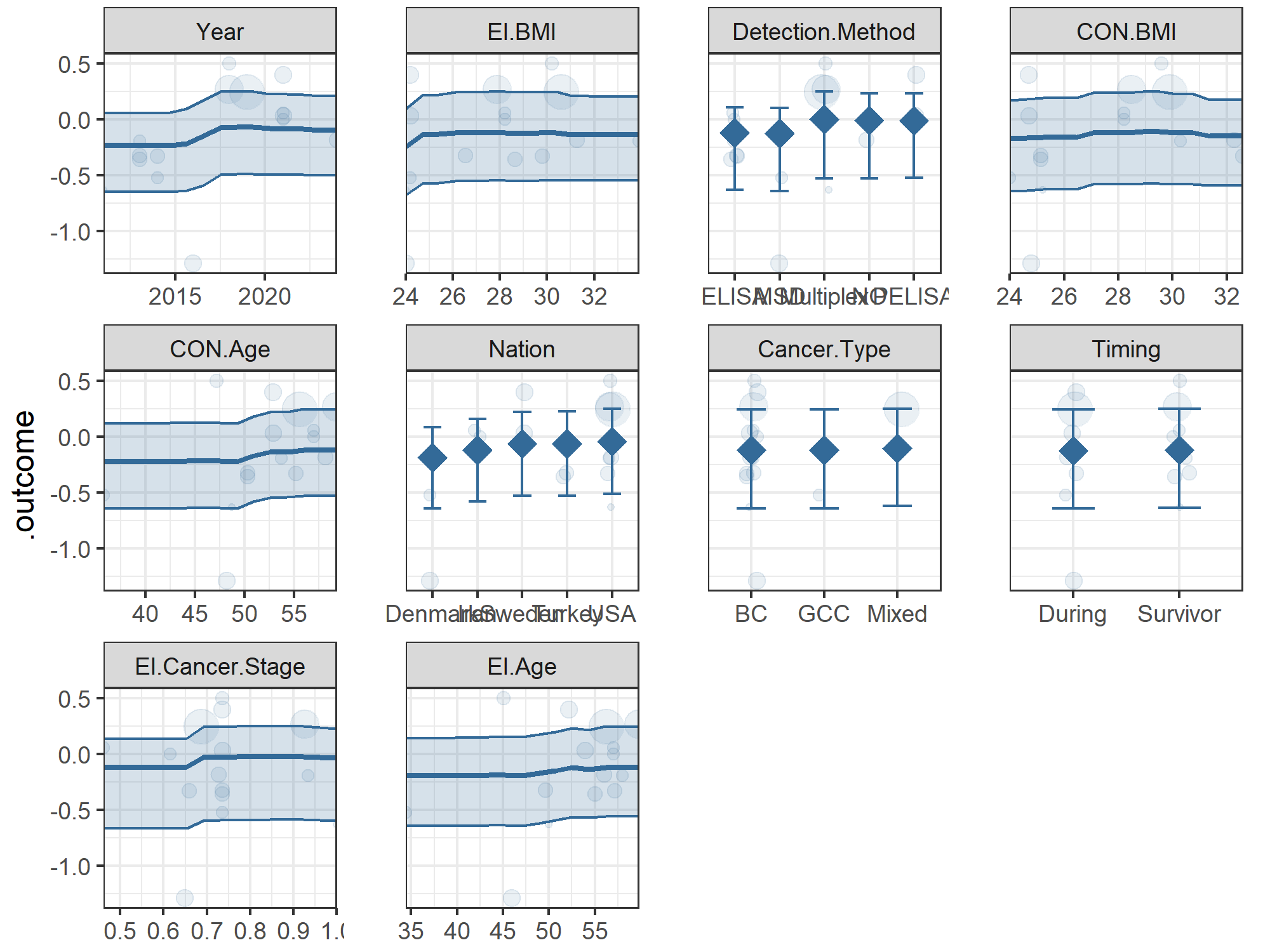


Fig. S53. Partial dependence plot (background moderator variables of IL-8)


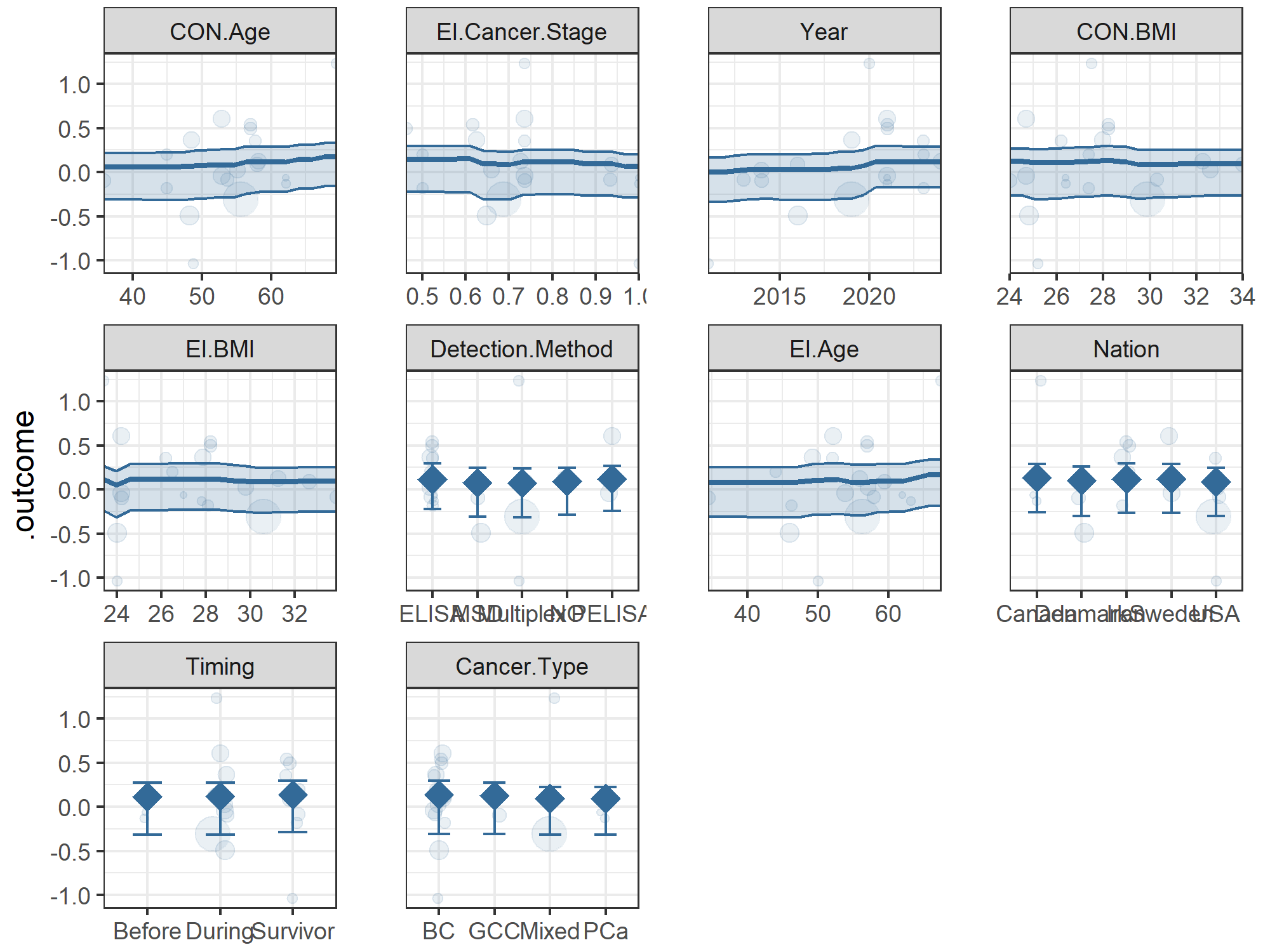


Fig. S54. Partial dependence plot (background moderator variables of IL-10)

**Supplementary information 10. Results of the MetaForest regression models for subgroups by cancer type.**


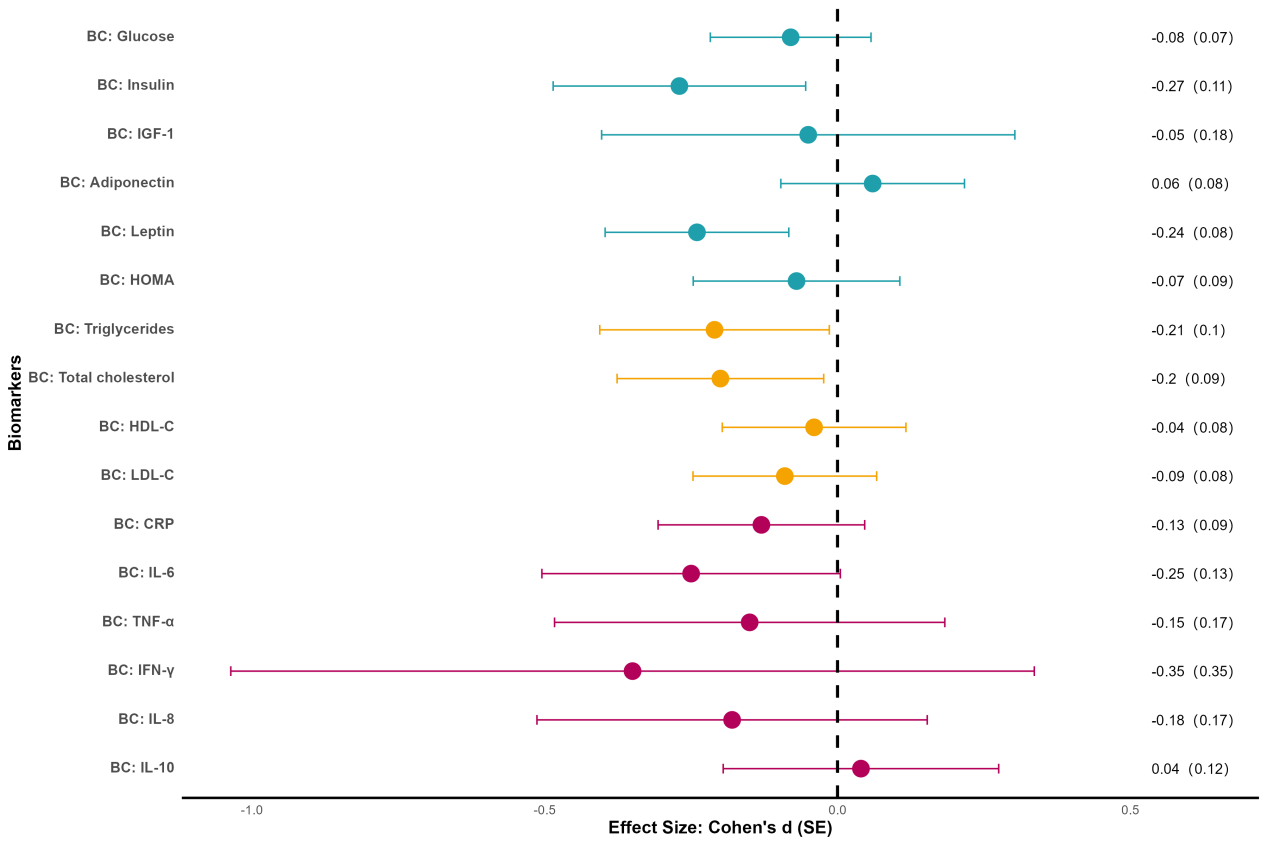


Fig. S55. Forest plot of subgroups by cancer type

Table S9. RVE model parameters without moderators for subgroups by cancer type

|  | ES | SE | t | dfs | prob | CI.L | CI.U | sig | I² |
| --- | --- | --- | --- | --- | --- | --- | --- | --- | --- |
| BC: Glucose | -0.08 | 0.07 | -1.22 | 9.76 | 0.25 | -0.23 | 0.07 |  | 0.00 |
| BC: Insulin | -0.27 | 0.11 | -2.46 | 20.99 | 0.02 | -0.50 | -0.04 | ** | 0.59 |
| BC: IGF-1 | -0.05 | 0.18 | -0.26 | 8.79 | 0.80 | -0.46 | 0.37 |  | 0.71 |
| BC: Adiponectin | 0.06 | 0.08 | 0.76 | 6.61 | 0.47 | -0.13 | 0.24 |  | 0.00 |
| BC: Leptin | -0.24 | 0.08 | -2.80 | 13.33 | 0.01 | -0.42 | -0.05 | ** | 0.32 |
| BC: HOMA index | -0.07 | 0.09 | -0.72 | 8.17 | 0.49 | -0.28 | 0.15 |  | 0.19 |
| BC: Triglycerides | -0.21 | 0.10 | -2.12 | 7.92 | 0.07 | -0.45 | 0.02 | * | 0.28 |
| BC: Total cholesterol | -0.20 | 0.09 | -2.21 | 7.62 | 0.06 | -0.40 | 0.01 | * | 0.00 |
| BC: HDL-C | -0.04 | 0.08 | -0.45 | 9.05 | 0.66 | -0.22 | 0.15 |  | 0.15 |
| BC: LDL-C | -0.09 | 0.08 | -1.08 | 6.67 | 0.32 | -0.29 | 0.11 |  | 0.00 |
| BC: CRP | -0.13 | 0.09 | -1.39 | 17.21 | 0.18 | -0.32 | 0.07 |  | 0.63 |
| BC: IL-6 | -0.25 | 0.13 | -1.98 | 27.17 | 0.06 | -0.51 | 0.01 | * | 0.68 |
| BC: TNF-α | -0.15 | 0.17 | -0.91 | 22.70 | 0.37 | -0.50 | 0.19 |  | 0.77 |
| BC: IFN-γ | -0.35 | 0.35 | -0.99 | 4.97 | 0.36 | -1.26 | 0.55 |  | 0.82 |
| BC: IL-8 | -0.18 | 0.17 | -1.06 | 8.77 | 0.31 | -0.57 | 0.20 |  | 0.69 |
| BC: IL-10 | 0.04 | 0.12 | 0.30 | 9.16 | 0.77 | -0.23 | 0.30 |  | 0.31 |

Table S10. Parameters of MetaForest regression models with exercise prescription moderator variables in breast cancer subgroups

| ID | R²_OOB_ | τ² | Feature Weights | Mtry | min.node.size | R²_CV_ | R²_CV_ SD | RMSE | RMSE SD | MAE | MAE SD |
| --- | --- | --- | --- | --- | --- | --- | --- | --- | --- | --- | --- |
| Glucose | -0.14 | 0.00 | fixed | 2.00 | 4.00 | 0.12 | 0.16 | 0.30 | 0.14 | 0.22 | 0.08 |
| Insulin | -0.32 | 0.28 | fixed | 2.00 | 4.00 | 0.08 | 0.11 | 0.62 | 0.16 | 0.45 | 0.11 |
| IGF-1 | -0.33 | 0.38 | fixed | 2.00 | 4.00 | 0.44 | 0.39 | 0.62 | 0.18 | 0.52 | 0.15 |
| Adiponectin | -0.06 | 0.02 | fixed | 2.00 | 2.00 | 0.24 | 0.26 | 0.26 | 0.06 | 0.21 | 0.06 |
| Leptin | -0.22 | 0.10 | fixed | 2.00 | 4.00 | 0.11 | 0.13 | 0.44 | 0.13 | 0.35 | 0.10 |
| HOMA index | -0.35 | 0.03 | fixed | 2.00 | 4.00 | 0.21 | 0.23 | 0.40 | 0.11 | 0.30 | 0.09 |
| Triglycerides | -0.10 | 0.01 | fixed | 2.00 | 2.00 | 0.22 | 0.23 | 0.37 | 0.15 | 0.27 | 0.11 |
| Total cholesterol | -0.27 | 0.03 | fixed | 2.00 | 4.00 | 0.20 | 0.21 | 0.34 | 0.08 | 0.27 | 0.07 |
| HDL-C | 0.00 | 0.01 | fixed | 2.00 | 2.00 | 0.20 | 0.23 | 0.39 | 0.13 | 0.30 | 0.10 |
| LDL-C | 0.18 | 0.00 | unif | 2.00 | 2.00 | 0.44 | 0.27 | 0.24 | 0.07 | 0.20 | 0.06 |
| CRP | -0.01 | 0.11 | unif | 2.00 | 4.00 | 0.15 | 0.15 | 0.37 | 0.07 | 0.28 | 0.06 |
| IL-6 | -0.10 | 0.44 | fixed | 2.00 | 4.00 | 0.12 | 0.15 | 0.84 | 0.36 | 0.51 | 0.15 |
| TNF-α | 0.02 | 0.50 | fixed | 2.00 | 4.00 | 0.22 | 0.21 | 0.83 | 0.29 | 0.55 | 0.16 |
| IL-8 | -0.03 | 0.20 | unif | 2.00 | 2.00 | 0.39 | 0.29 | 0.44 | 0.15 | 0.35 | 0.13 |
| IL-10 | 0.29 | 0.00 | fixed | 2.00 | 4.00 | 0.40 | 0.28 | 0.35 | 0.15 | 0.28 | 0.11 |

Note: R^2^_OOB_: Out-of-Bag R-Squared. τ²: Between-Studies Variance. R^2^_CV_: Cross-Validation R-Squared. R^2^_CV_ SD: Cross-Validation R-Squared Standard Deviation. Mtry: Number of Variables Randomly Sampled as Candidates at Each Split. min.node.size: Minimum Node Size. RMSE: Root Mean Square Error. MASE: Mean Absolute Scaled Error.


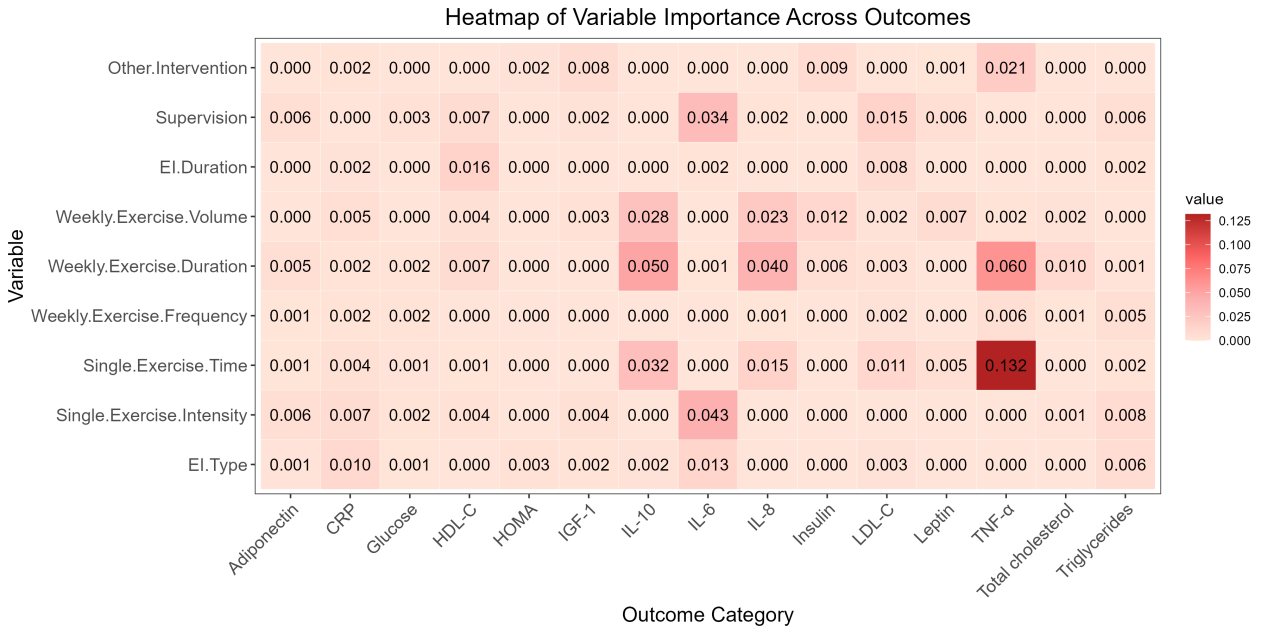


Fig. S56. Heat map of the importance of exercise prescription moderator variables in BC subgroups


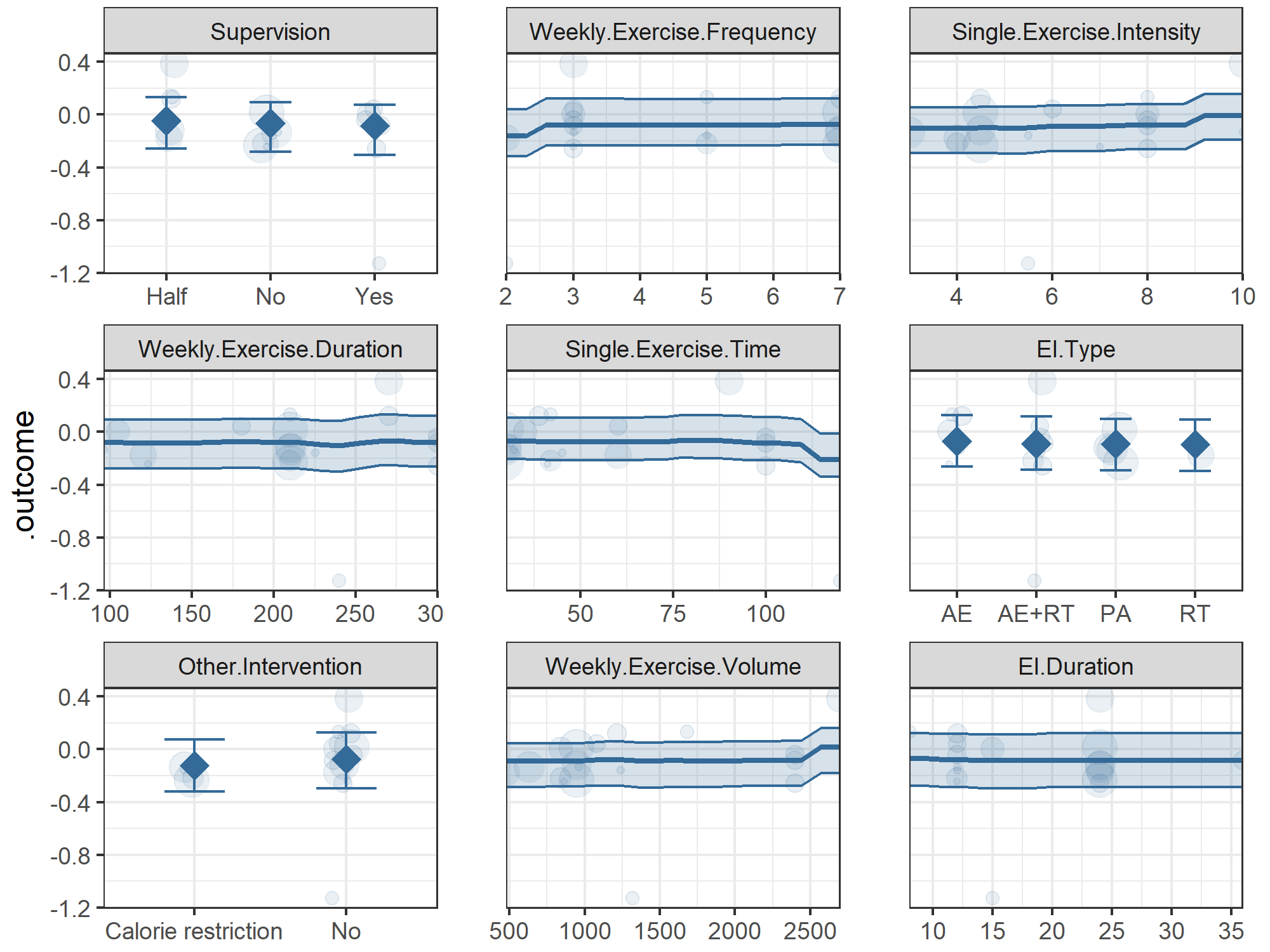


Fig. S57. Partial dependence plot (exercise prescription moderator variables of Glucose in BC subgroups)


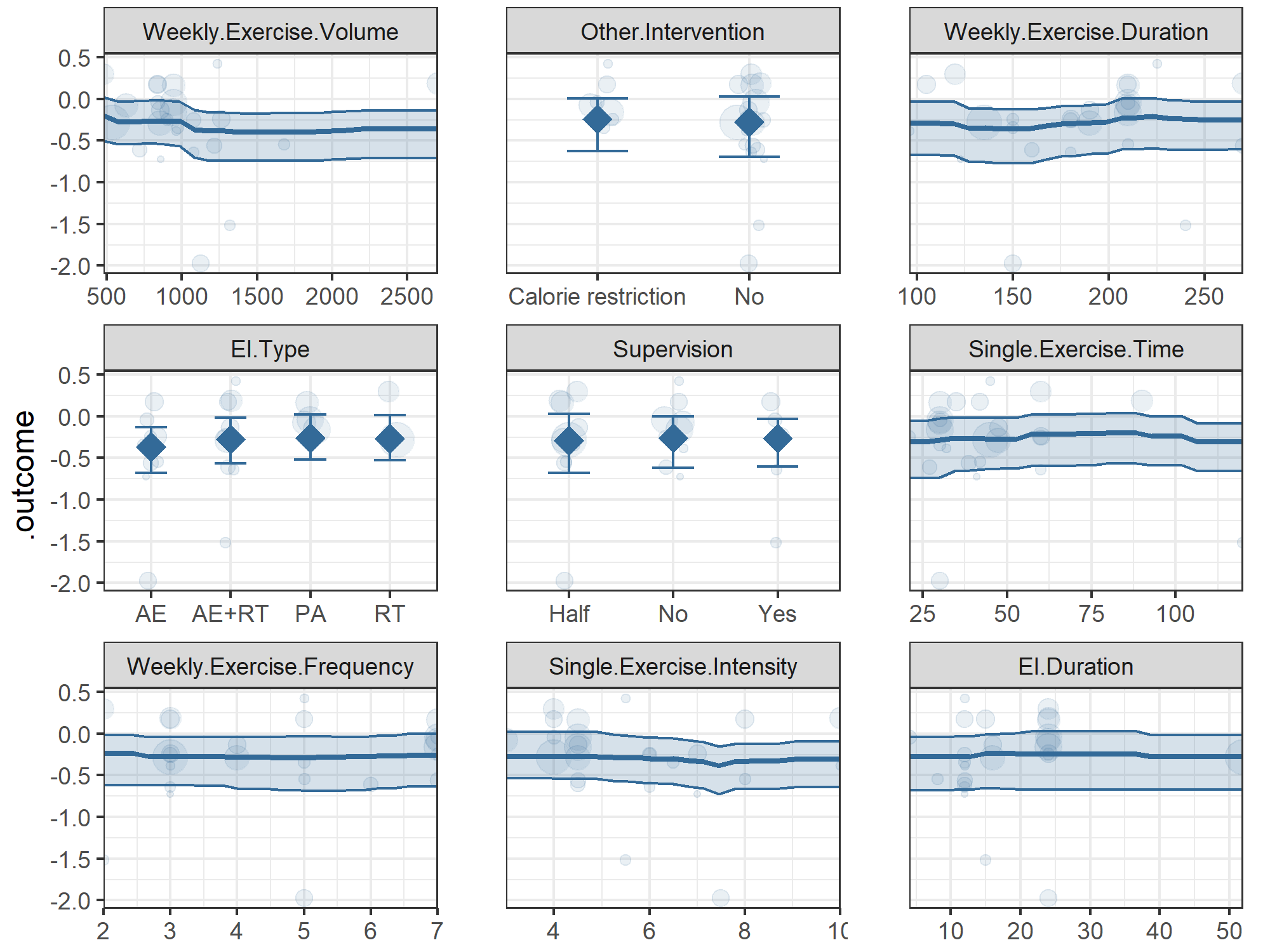


Fig. S58. Partial dependence plot (exercise prescription moderator variables of Insulin in BC subgroups)


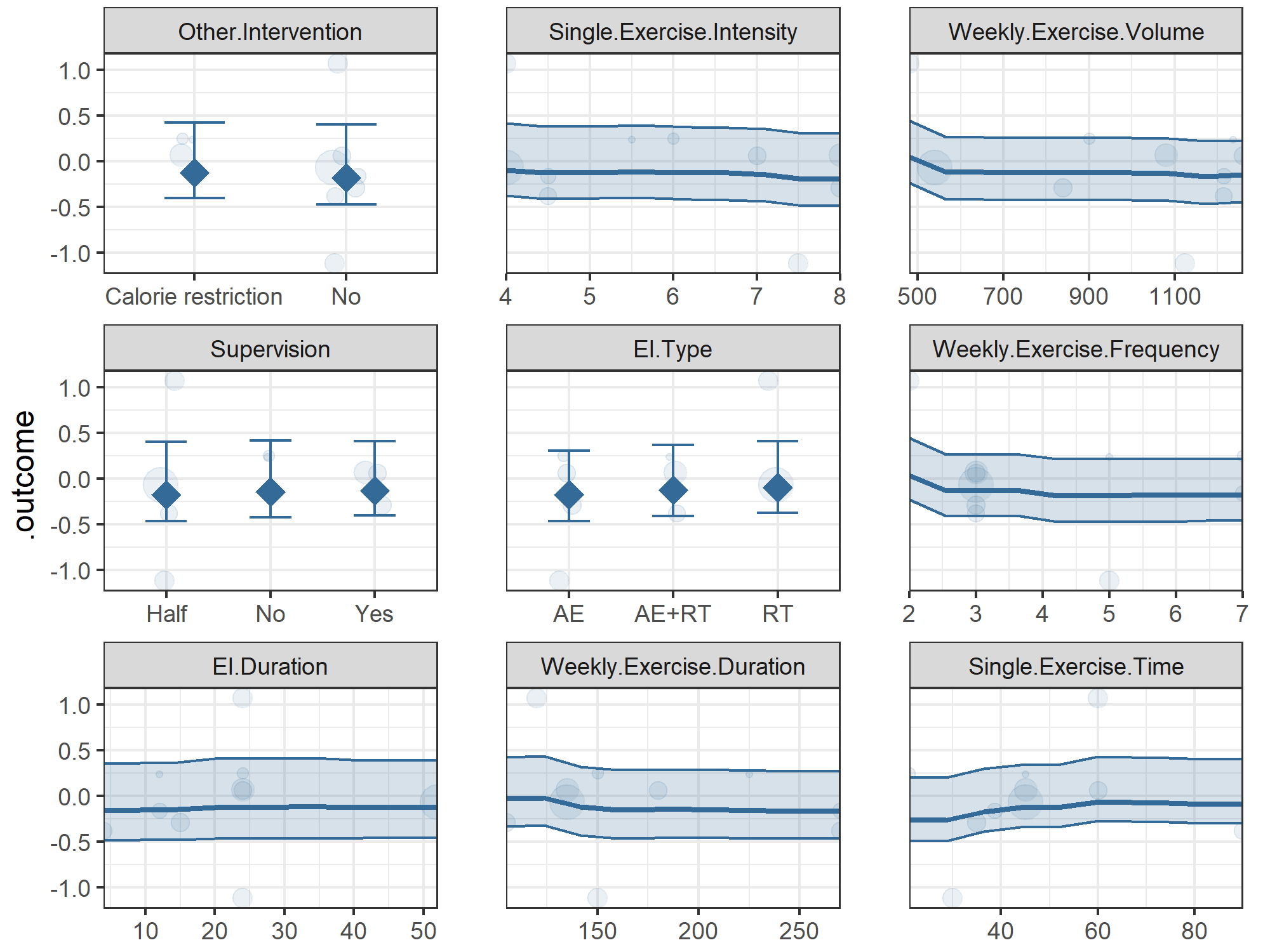


Fig. S59. Partial dependence plot (exercise prescription moderator variables of IGF-1 in BC subgroups)


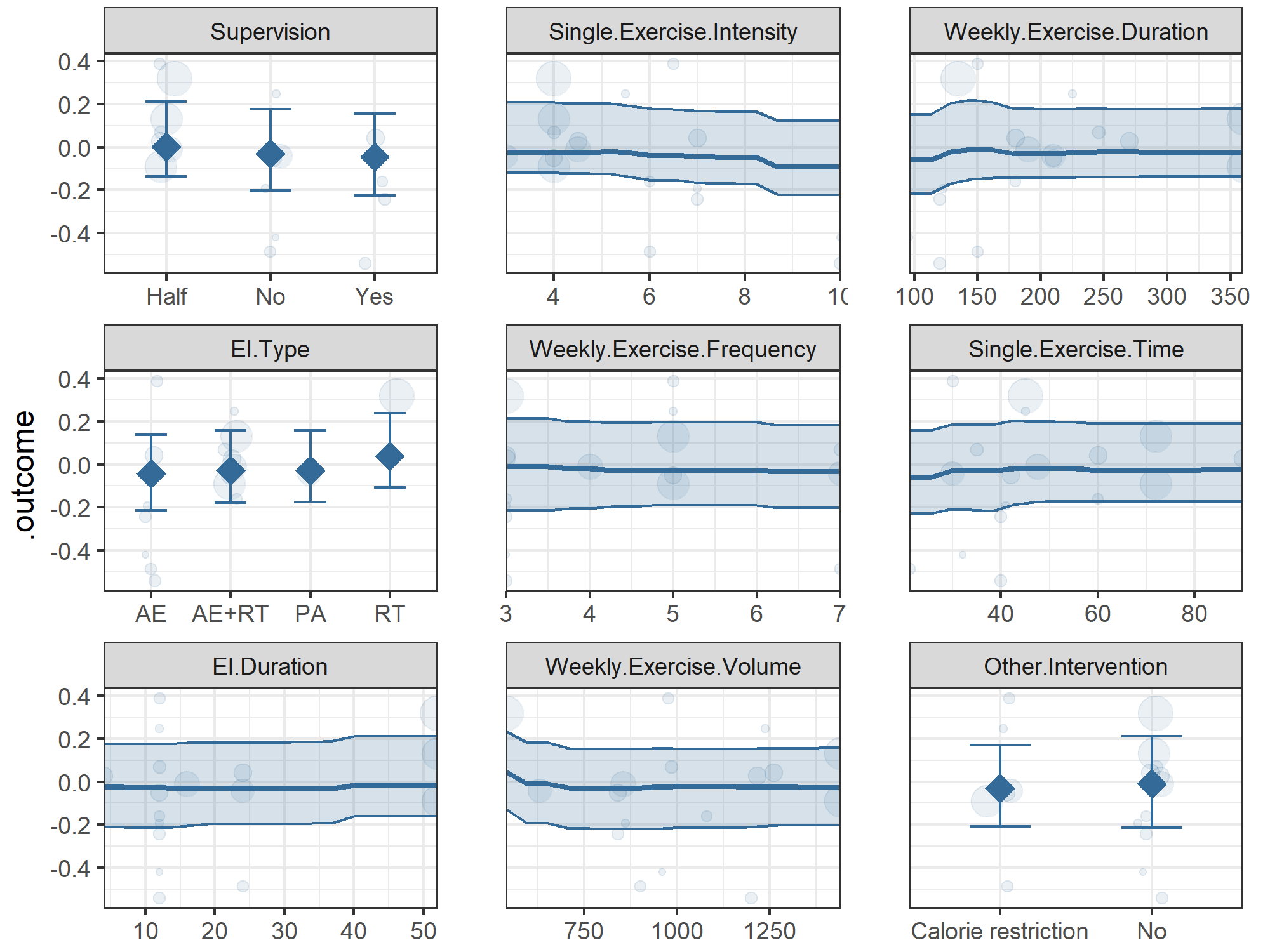


Fig. S60. Partial dependence plot (exercise prescription moderator variables of Adiponectin in BC subgroups)


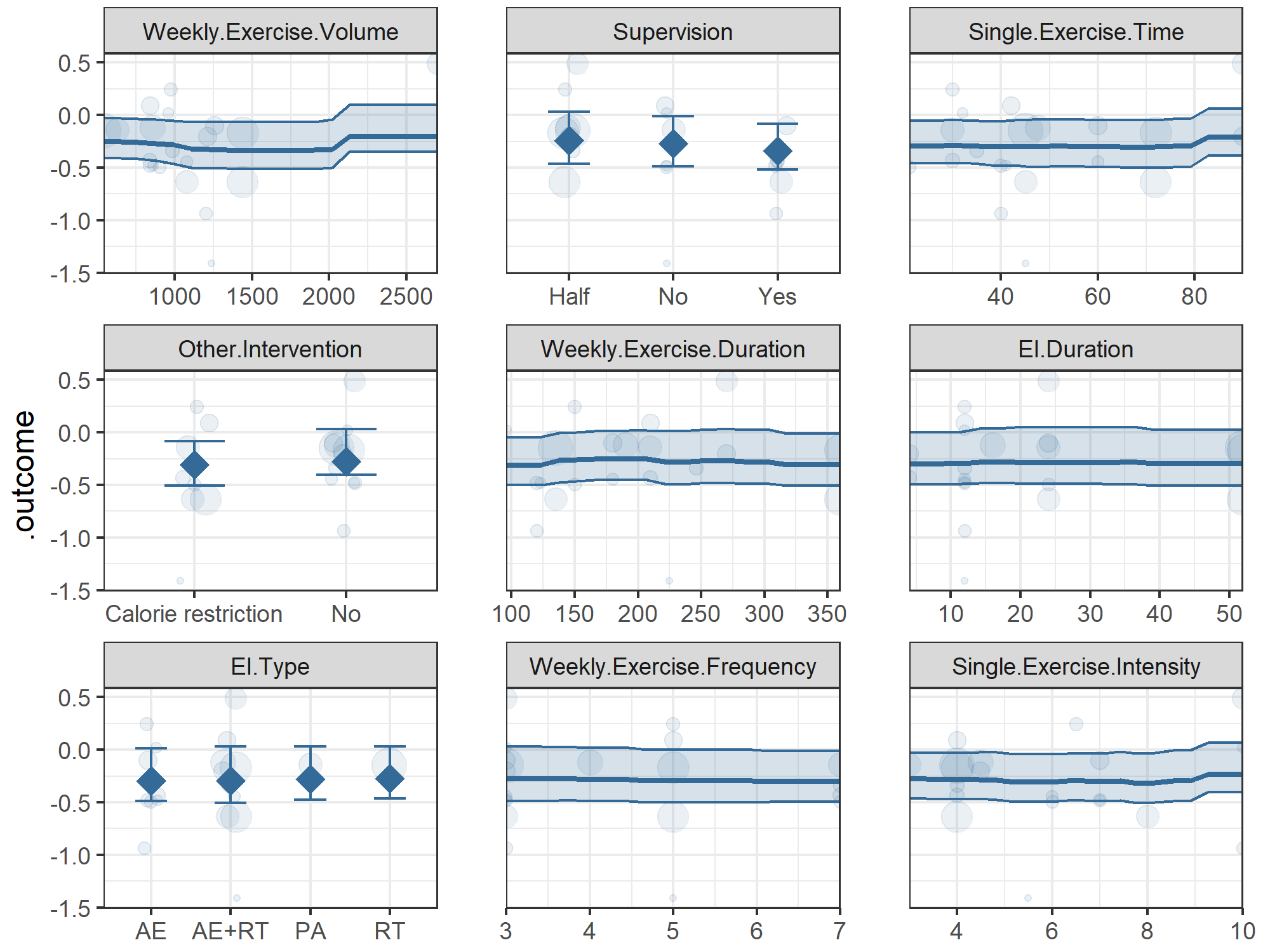


Fig. S61. Partial dependence plot (exercise prescription moderator variables of Leptin in BC subgroups)


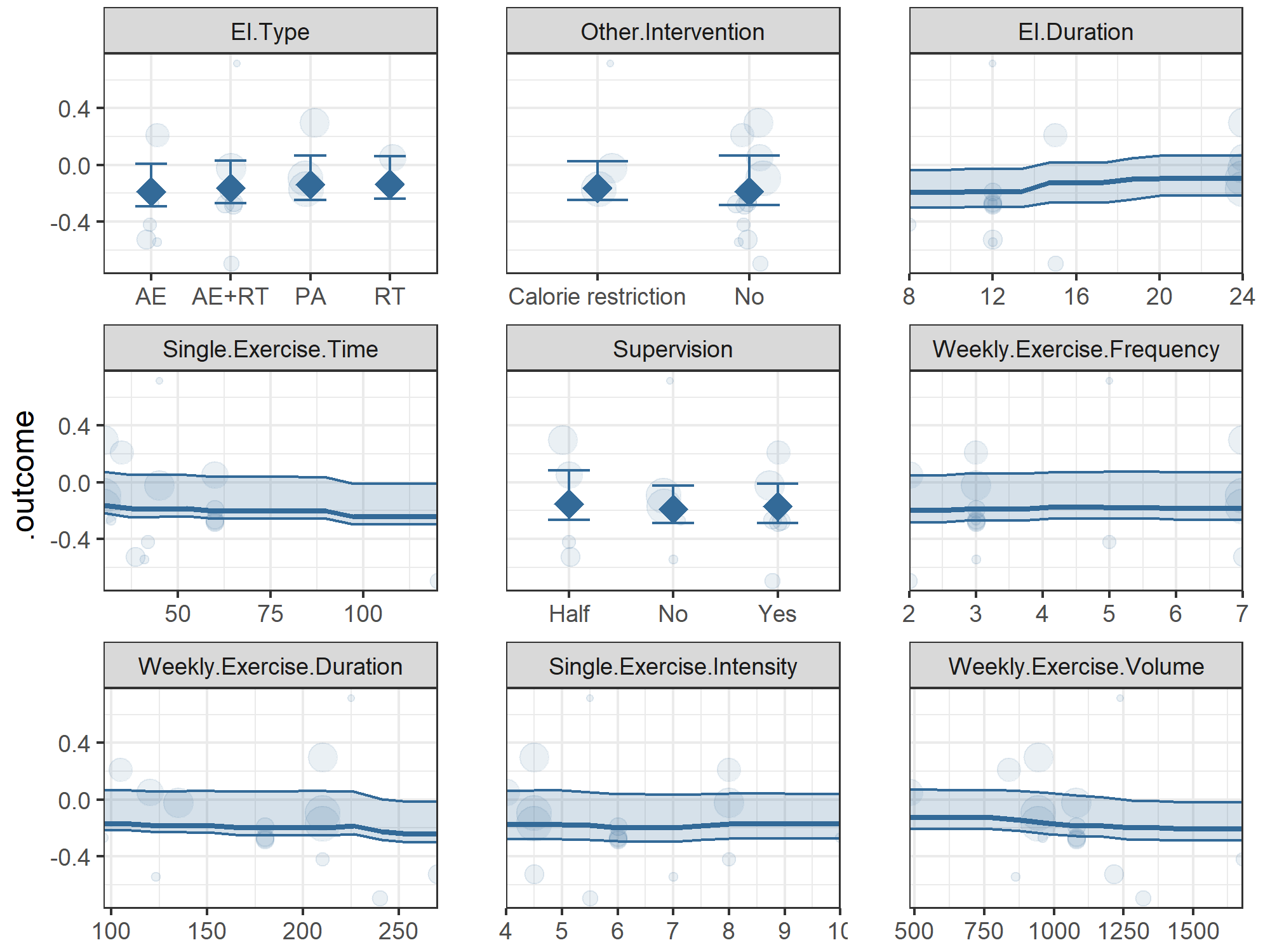


Fig. S62. Partial dependence plot (exercise prescription moderator variables of HOMA index in BC subgroups)


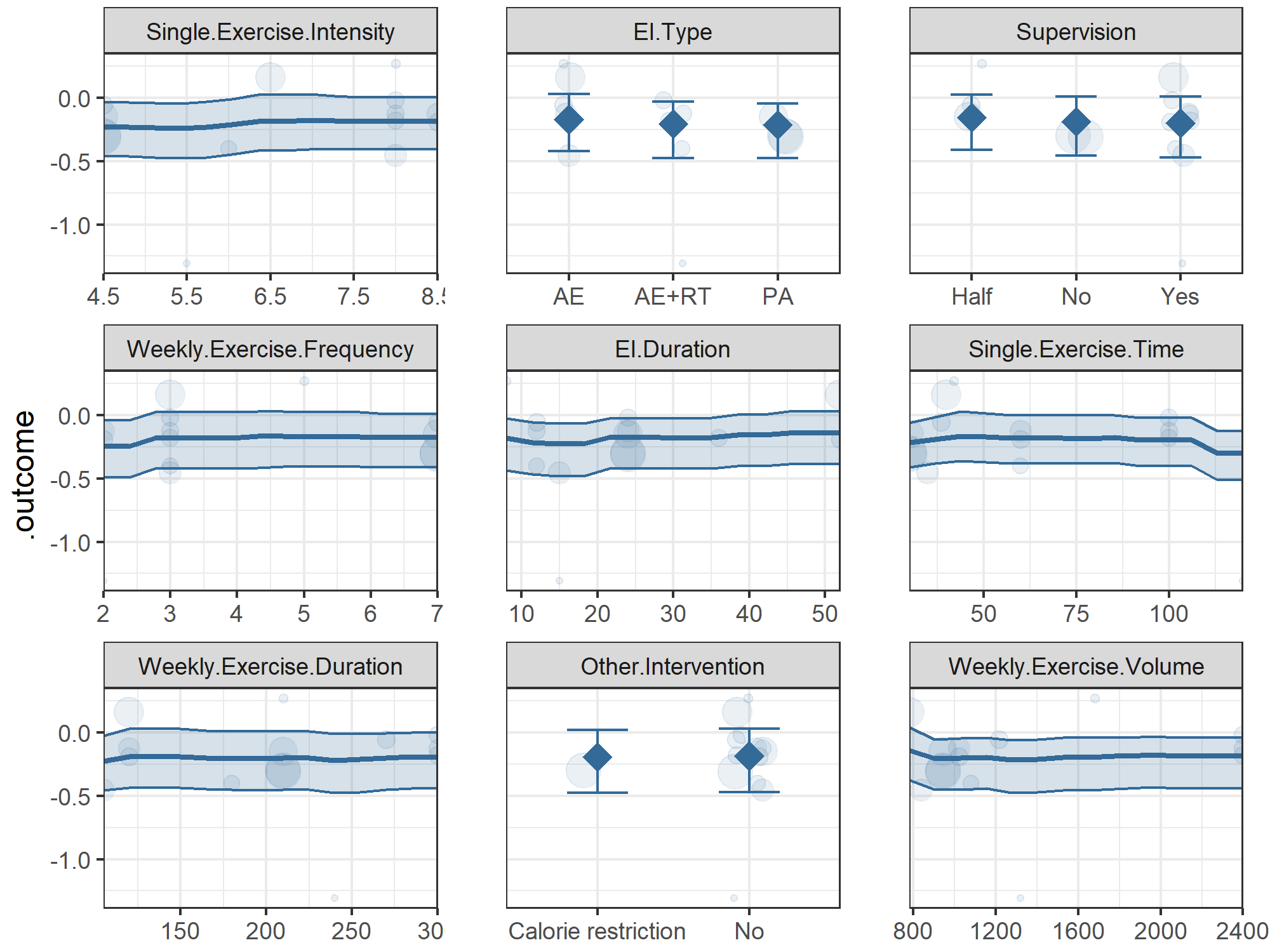


Fig. S63. Partial dependence plot (exercise prescription moderator variables of Triglycerides in BC subgroups)


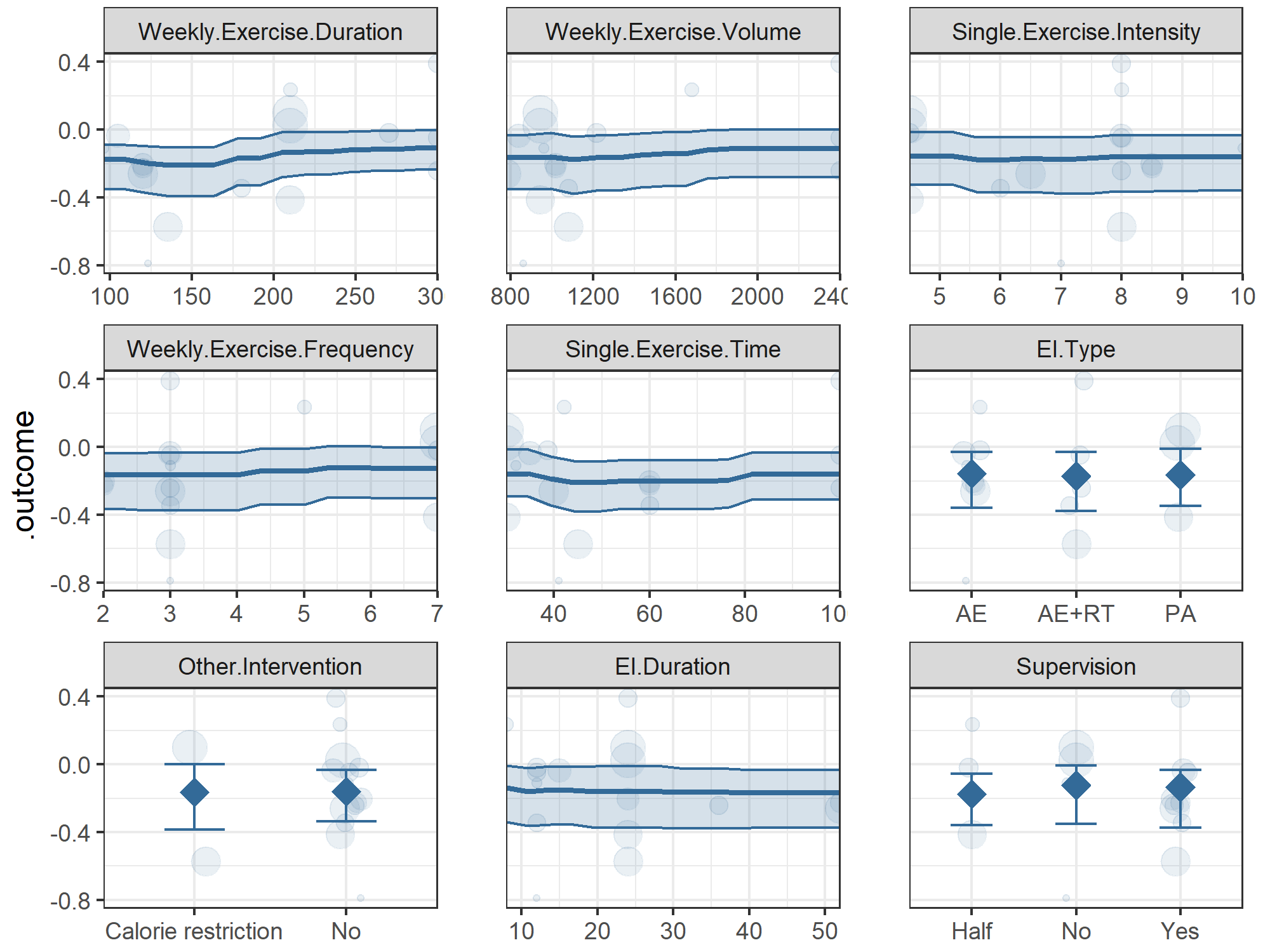


Fig. S64. Partial dependence plot (exercise prescription moderator variables of Total cholesterol in BC subgroups)


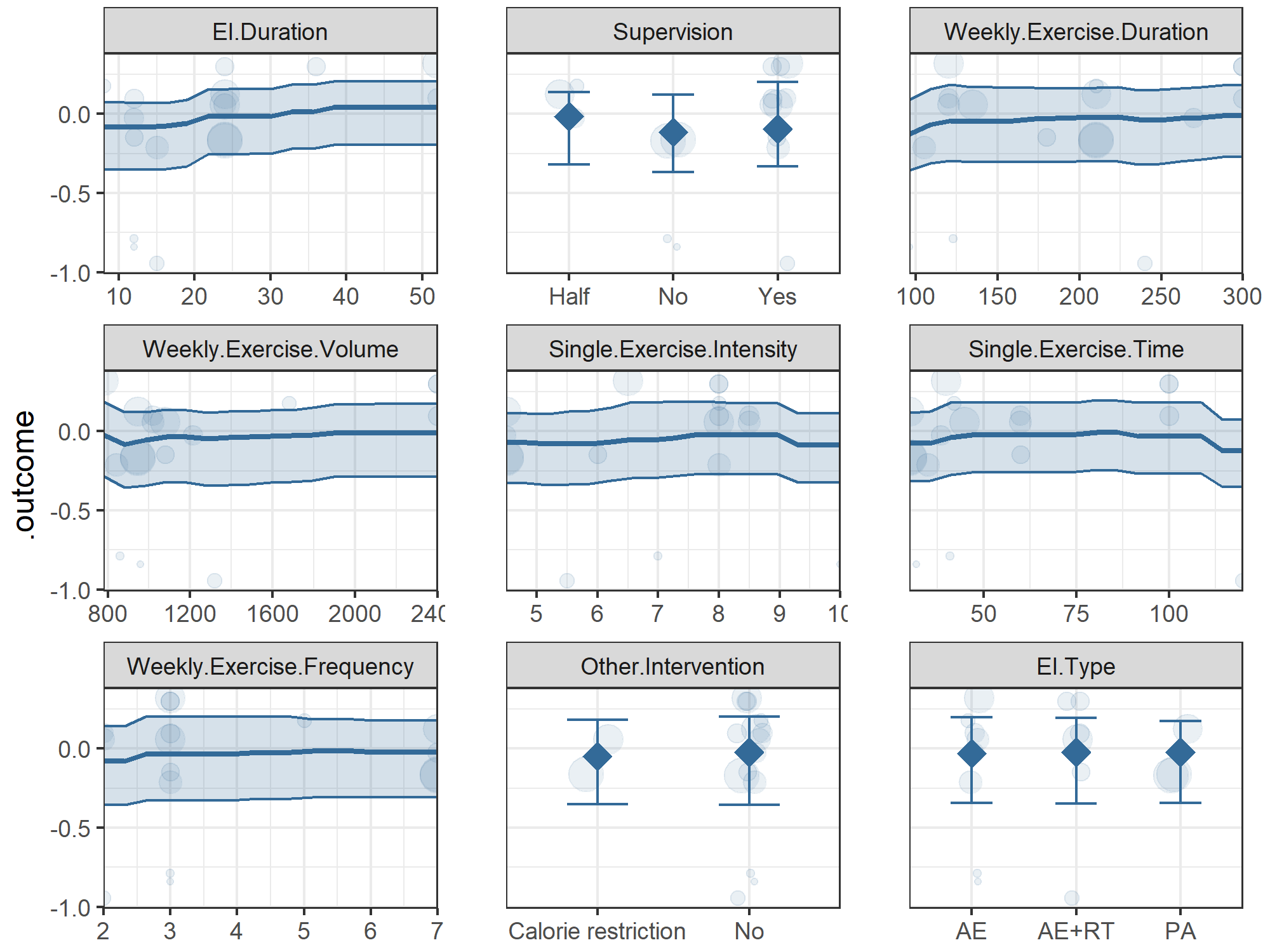


Fig. S65. Partial dependence plot (exercise prescription moderator variables of HDL-C in BC subgroups)


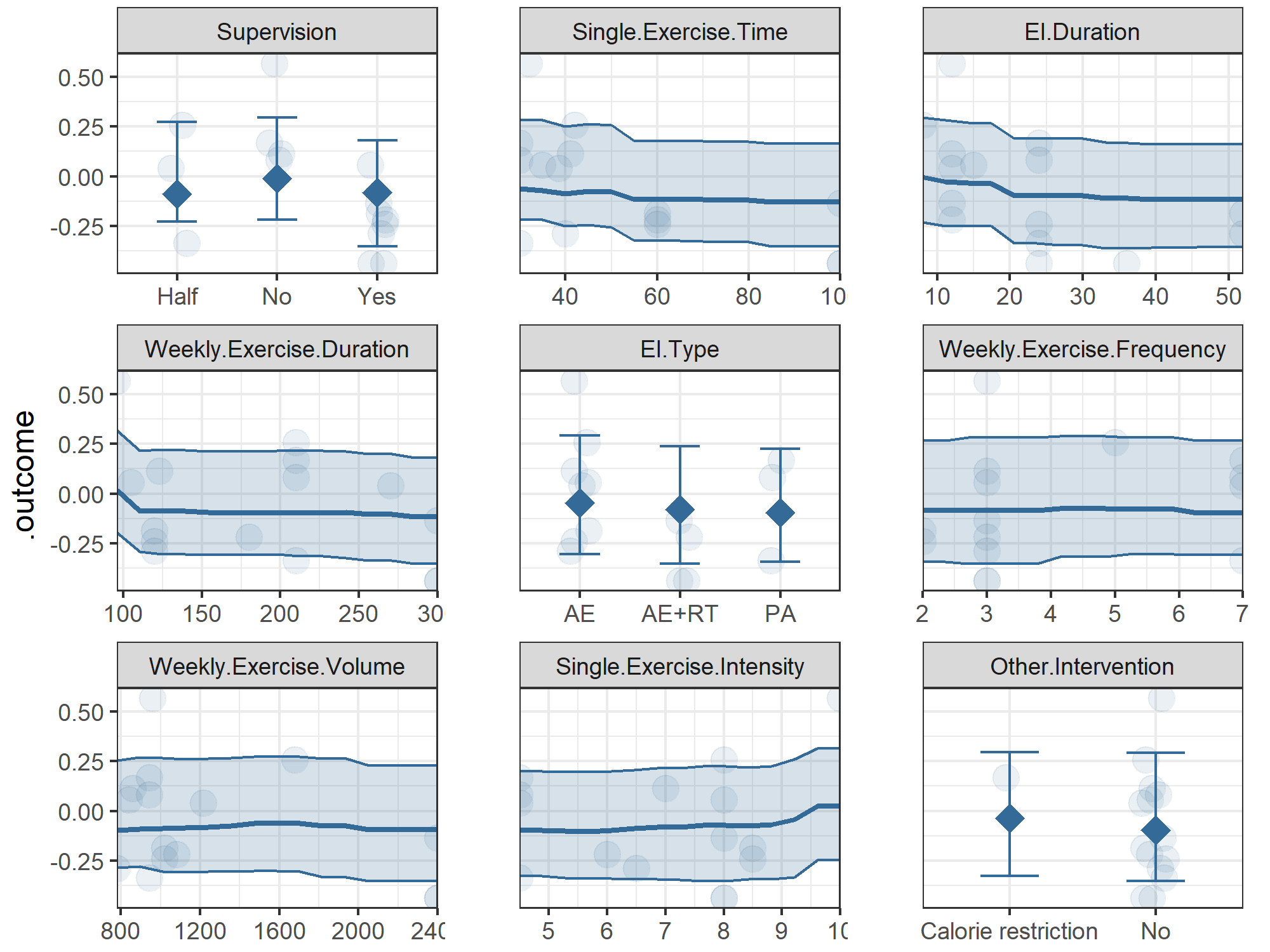


Fig. S66. Partial dependence plot (exercise prescription moderator variables of LDL-C in BC subgroups)


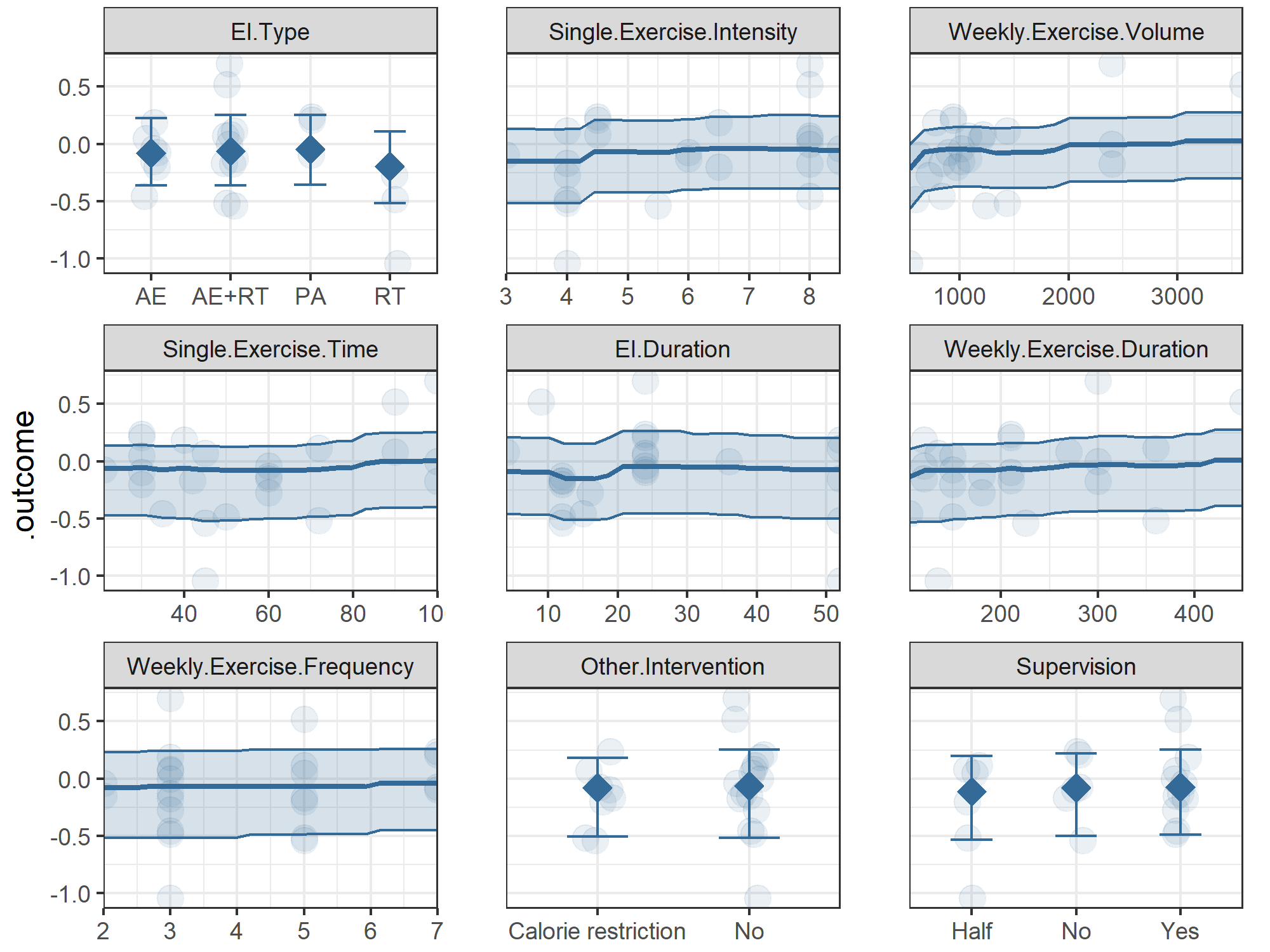


Fig. S67. Partial dependence plot (exercise prescription moderator variables of CRP in BC subgroups)


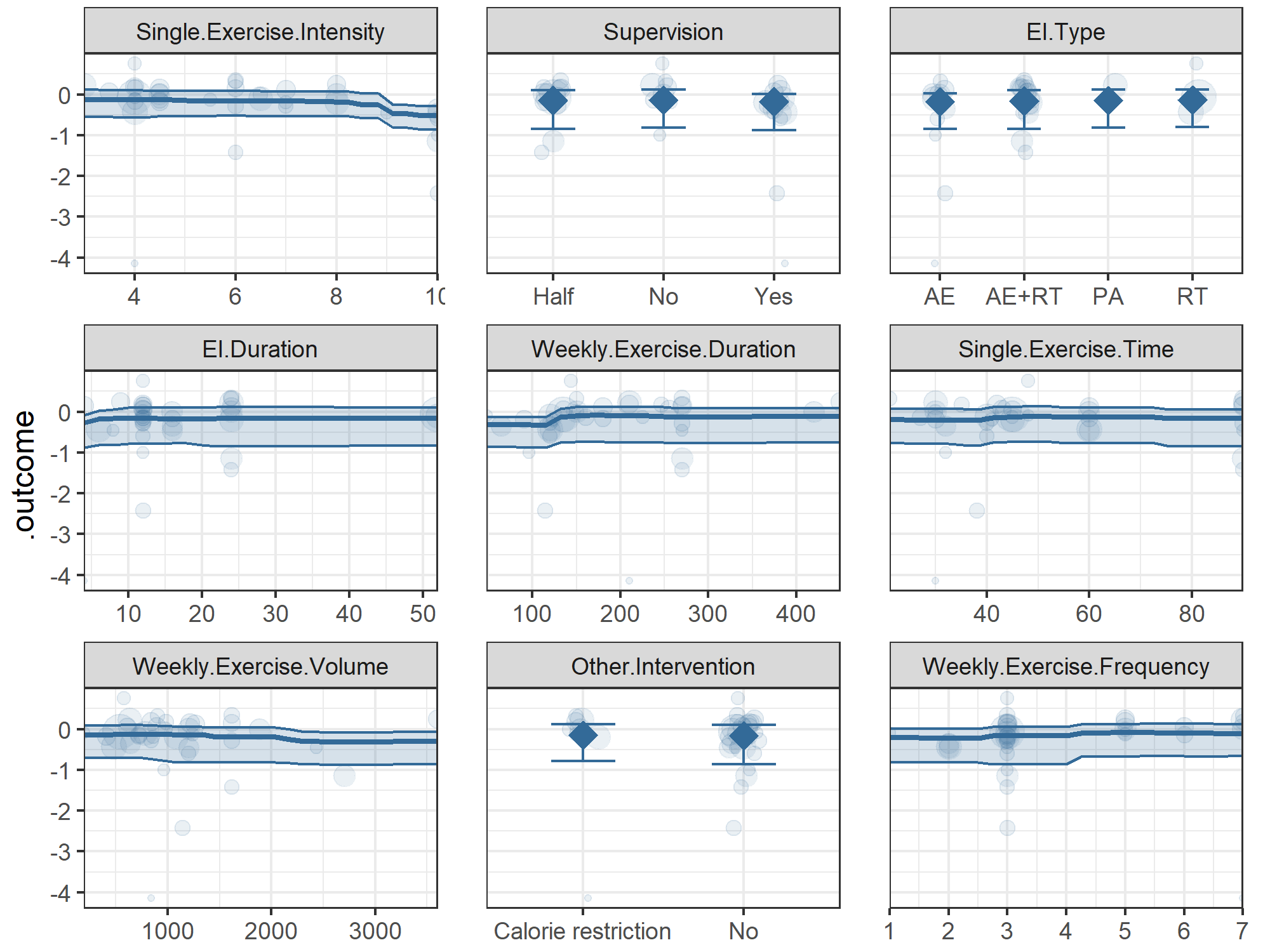


Fig. S68. Partial dependence plot (exercise prescription moderator variables of IL-6 in BC subgroups)


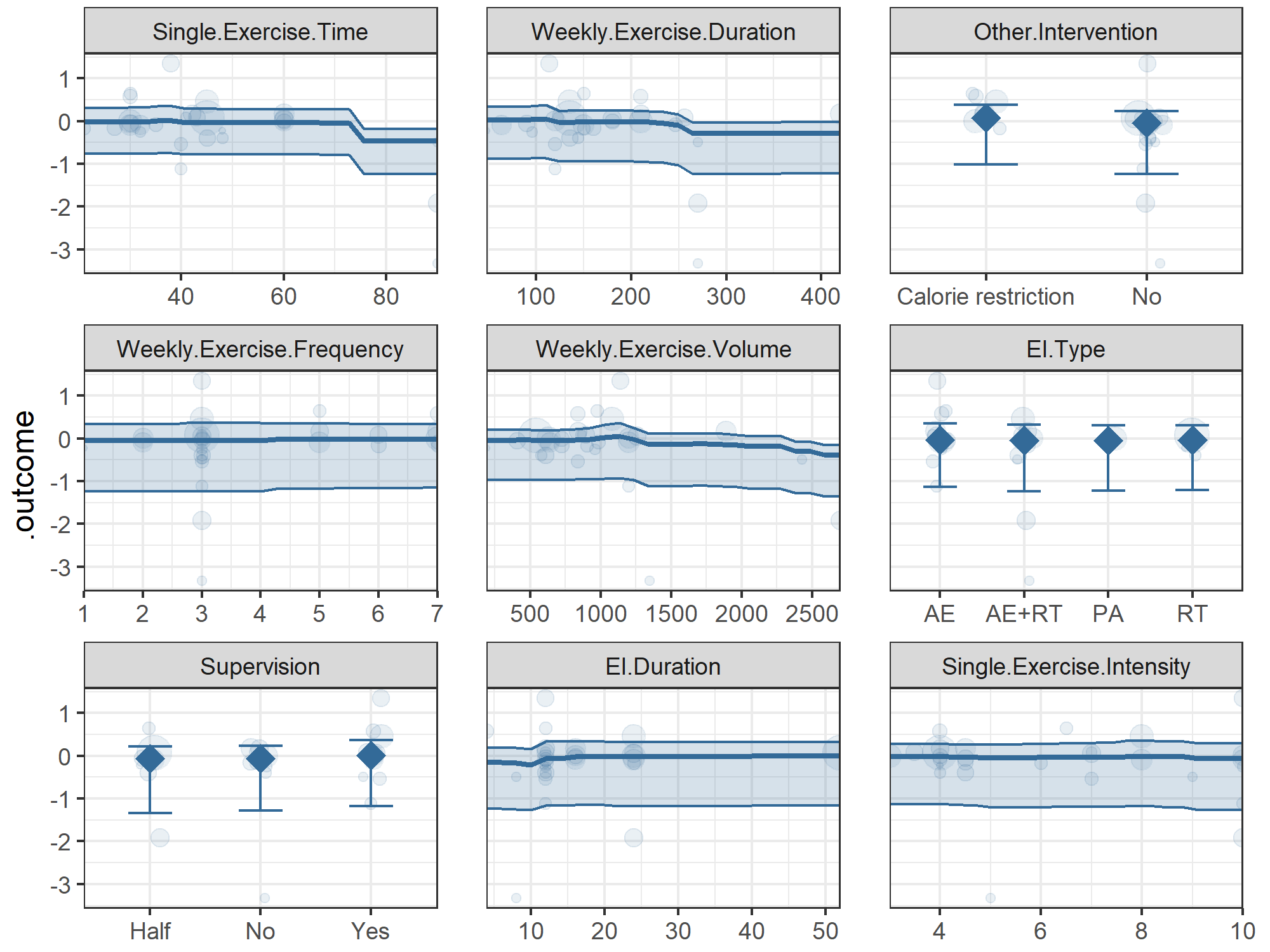


Fig. S69. Partial dependence plot (exercise prescription moderator variables of TNF-α in BC subgroups)


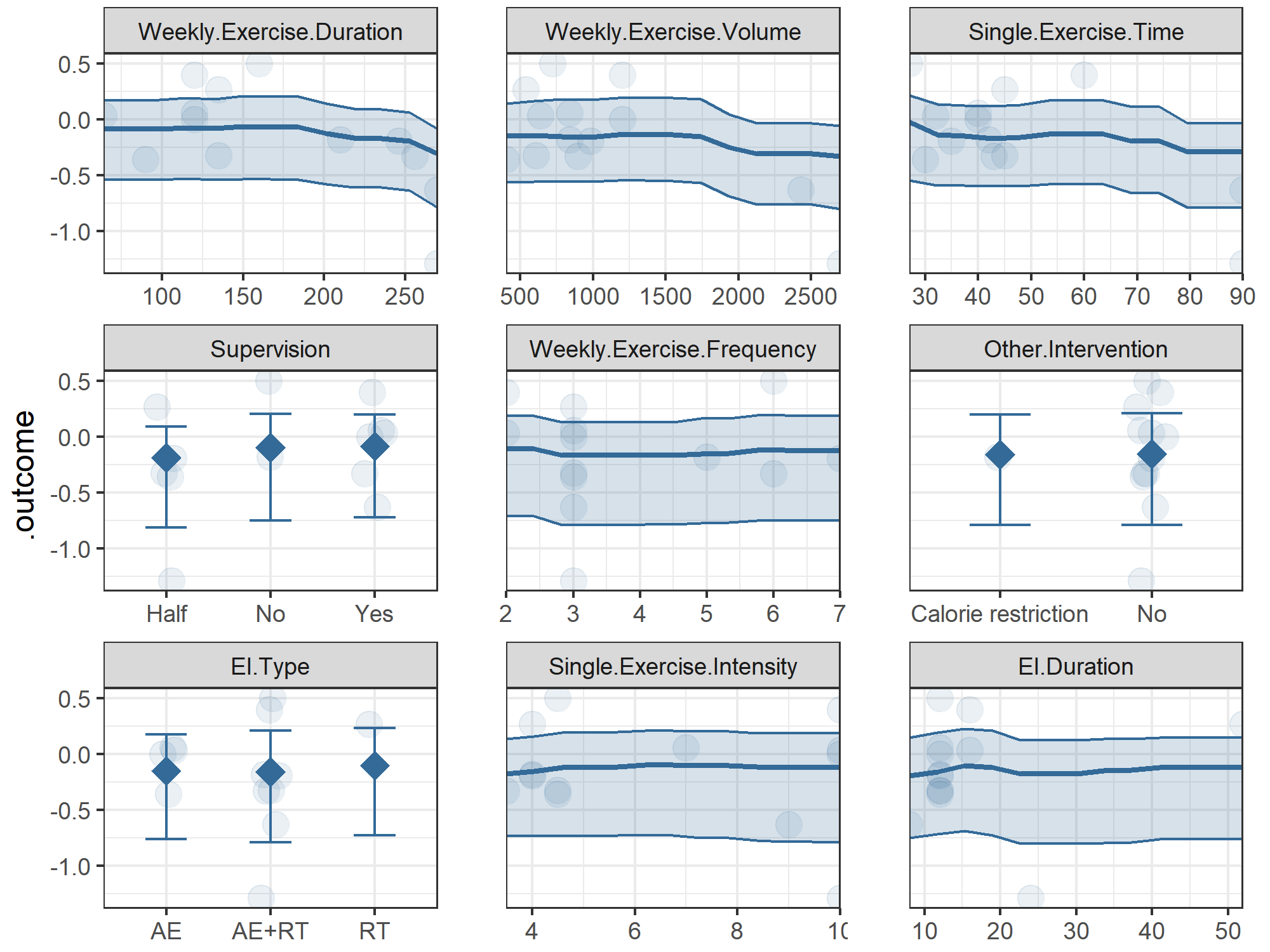


Fig. S70. Partial dependence plot (exercise prescription moderator variables of IL-8 in BC subgroups)


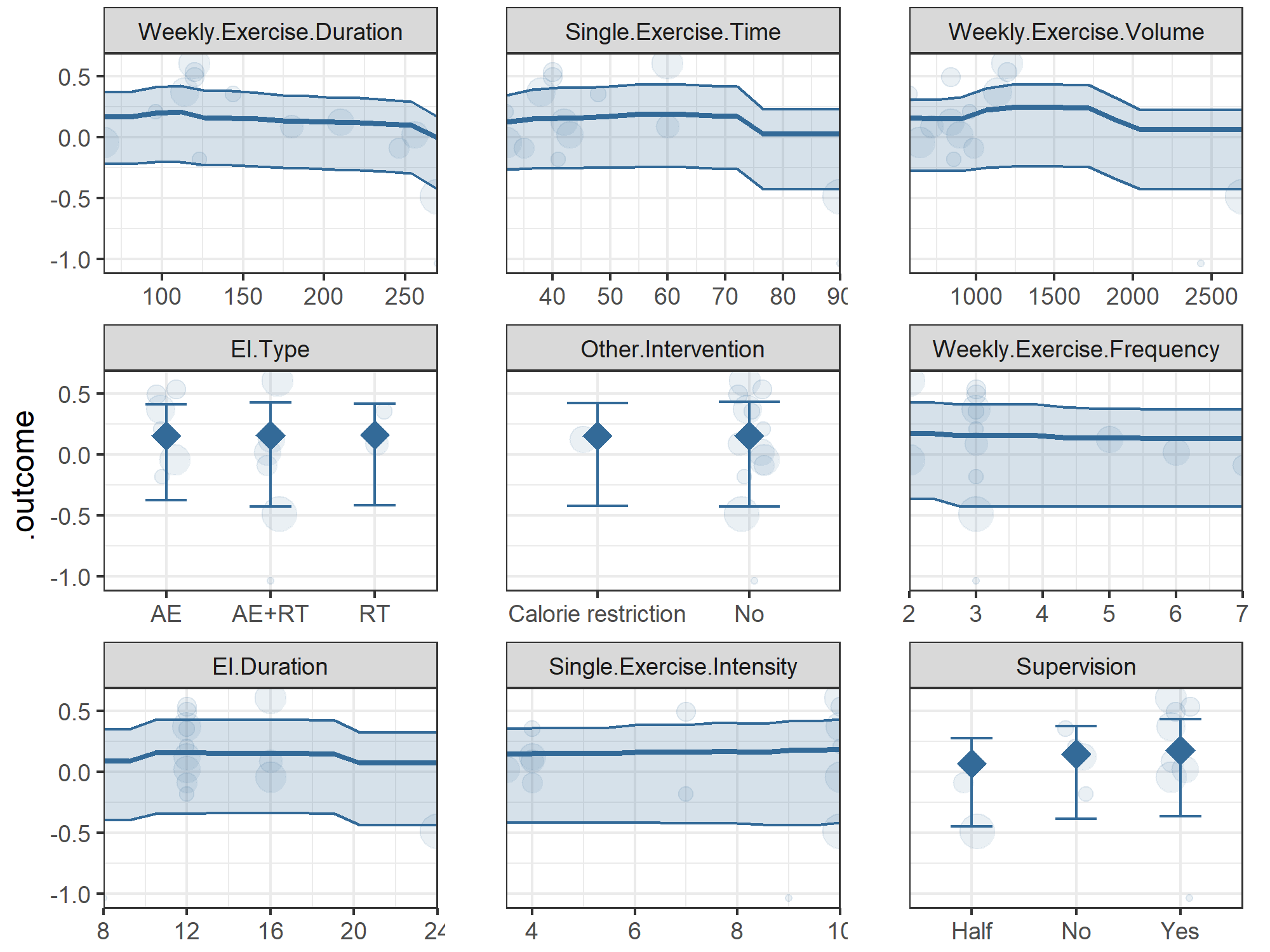


Fig. S71. Partial dependence plot (exercise prescription moderator variables of IL-10 in BC subgroups)

Table S11. Parameters of MetaForest regression models with background moderating variables in breast cancer subgroups

| ID | R²_OOB_ | τ² | Feature Weights | Mtry | min.node.size | R²_CV_ | R²_CV_ SD | RMSE | RMSE SD | MAE | MAE SD |
| --- | --- | --- | --- | --- | --- | --- | --- | --- | --- | --- | --- |
| (a) Exercise Prescription Moderator Variables | | | | | | | | | | | |
| Glucose | -0.19 | 0.01 | fixed | 2.00 | 4.00 | 0.13 | 0.20 | 0.31 | 0.14 | 0.22 | 0.08 |
| Insulin | -0.30 | 0.27 | fixed | 2.00 | 4.00 | 0.18 | 0.19 | 0.64 | 0.16 | 0.48 | 0.11 |
| Adiponectin | -0.07 | 0.03 | random | 2.00 | 4.00 | 0.20 | 0.22 | 0.26 | 0.06 | 0.21 | 0.05 |
| Leptin | -0.01 | 0.07 | fixed | 2.00 | 4.00 | 0.10 | 0.13 | 0.42 | 0.13 | 0.34 | 0.10 |
| HOMA index | 0.07 | 0.00 | unif | 2.00 | 3.00 | 0.29 | 0.27 | 0.33 | 0.11 | 0.25 | 0.09 |
| Triglycerides | -0.22 | 0.01 | fixed | 2.00 | 2.00 | 0.25 | 0.31 | 0.38 | 0.17 | 0.27 | 0.12 |
| Total cholesterol | -0.05 | 0.00 | fixed | 2.00 | 4.00 | 0.15 | 0.20 | 0.31 | 0.08 | 0.25 | 0.07 |
| HDL-C | 0.08 | 0.00 | unif | 2.00 | 2.00 | 0.27 | 0.27 | 0.38 | 0.13 | 0.29 | 0.10 |
| LDL-C | 0.22 | 0.00 | unif | 2.00 | 3.00 | 0.42 | 0.25 | 0.25 | 0.08 | 0.21 | 0.06 |
| CRP | -0.31 | 0.13 | unif | 2.00 | 2.00 | 0.08 | 0.11 | 0.41 | 0.08 | 0.33 | 0.07 |
| IL-6 | -0.13 | 0.45 | fixed | 2.00 | 4.00 | 0.10 | 0.13 | 0.87 | 0.36 | 0.53 | 0.16 |
| TNF-α | -0.13 | 0.61 | fixed | 2.00 | 4.00 | 0.10 | 0.10 | 0.87 | 0.29 | 0.56 | 0.17 |
| IL-8 | -0.03 | 0.18 | unif | 2.00 | 2.00 | 0.32 | 0.31 | 0.45 | 0.19 | 0.34 | 0.14 |
| IL-10 | 0.08 | 0.07 | unif | 2.00 | 3.00 | 0.30 | 0.27 | 0.40 | 0.14 | 0.33 | 0.11 |


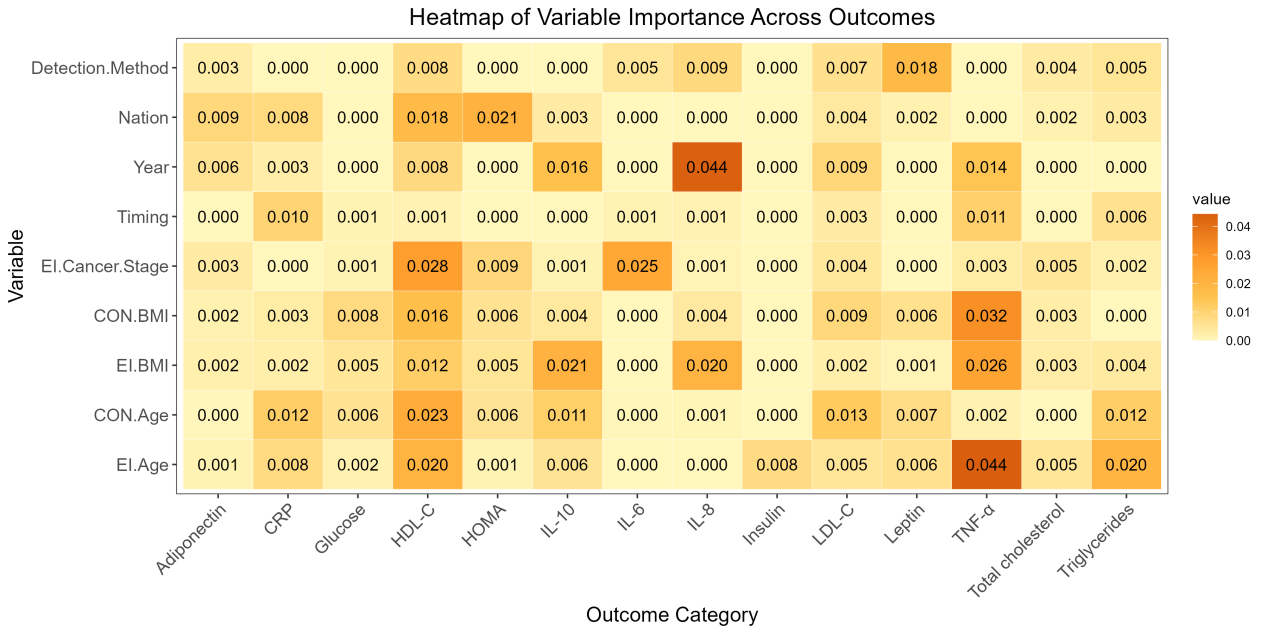


Fig. S72. Heat map of the importance of background moderator variables in BC subgroups


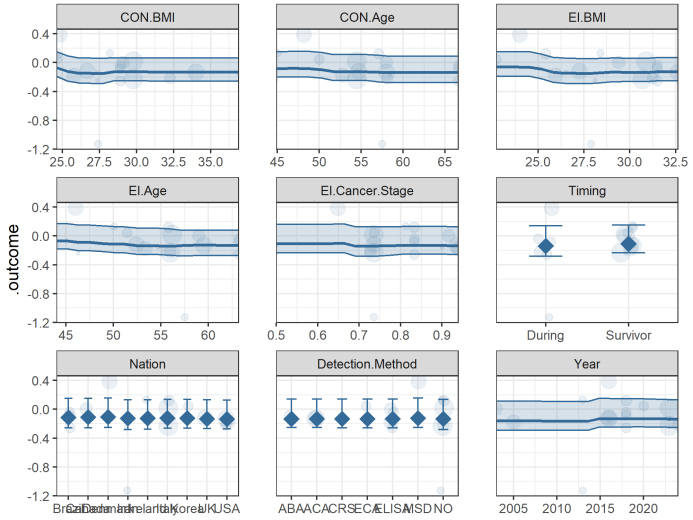


Fig. S73. Partial dependence plot (background moderator variables of Glucose in BC subgroups)


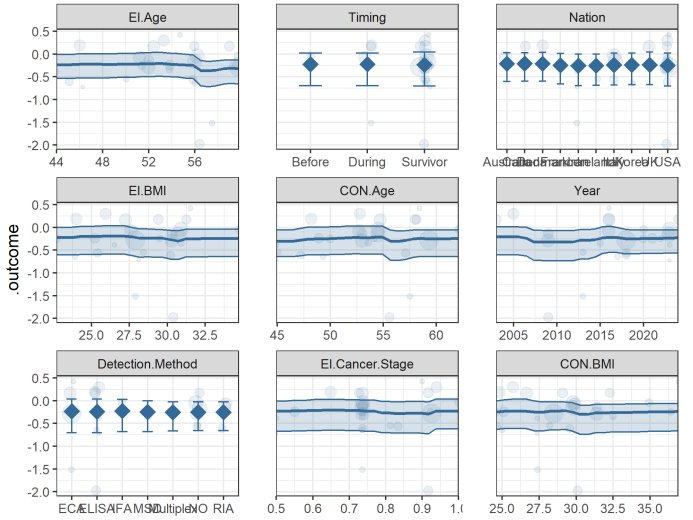


Fig. S74. Partial dependence plot (background moderator variables of Insulin in BC subgroups)


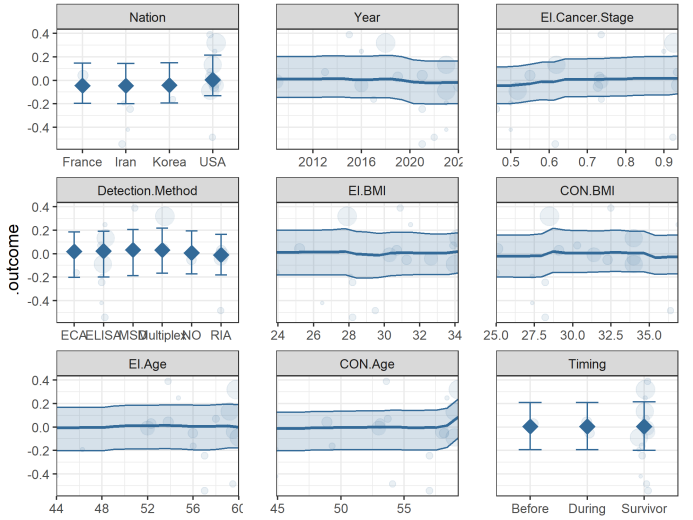


Fig. S75. Partial dependence plot (background moderator variables of Adiponectin in BC subgroups)


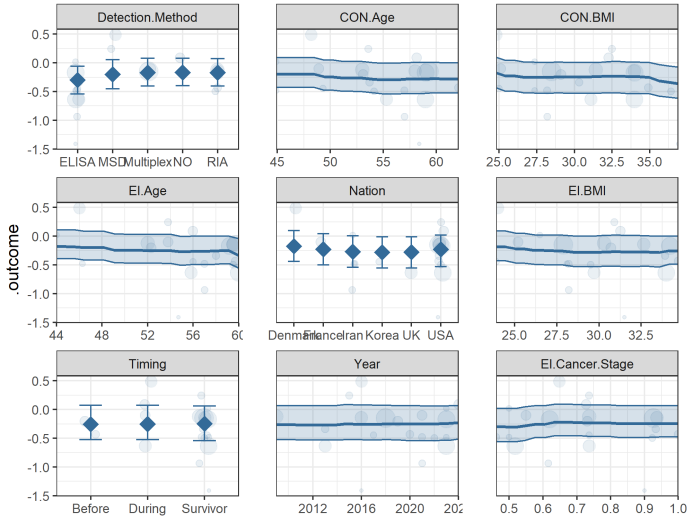


Fig. S76. Partial dependence plot (background moderator variables of Leptin in BC subgroups)


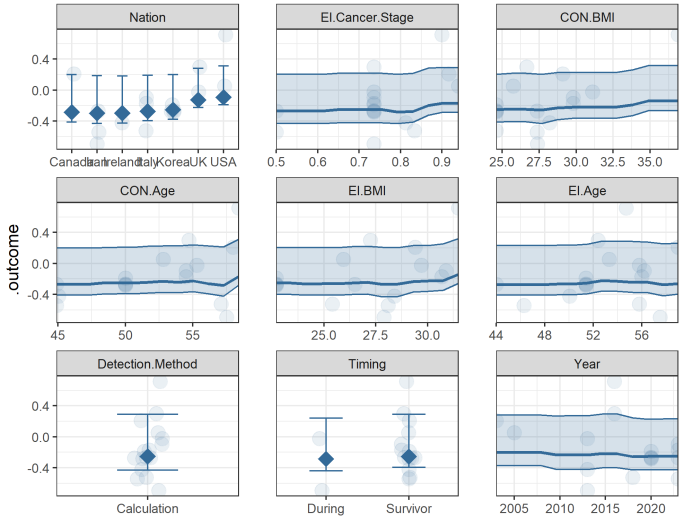


Fig. S77. Partial dependence plot (background moderator variables of HOMA index in BC subgroups)


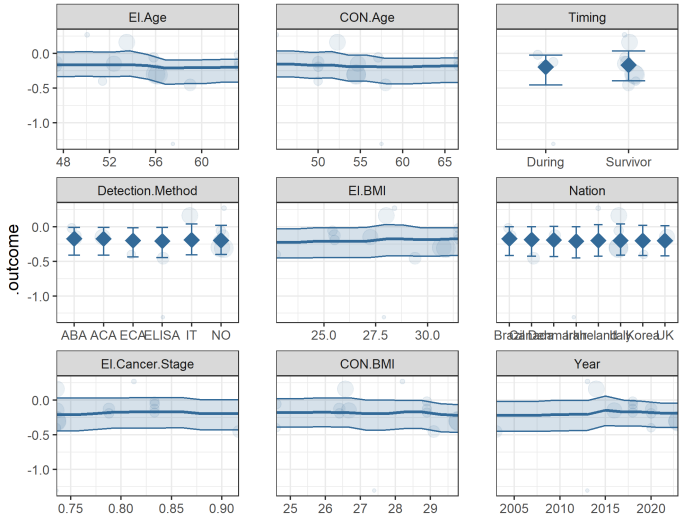


Fig. S78. Partial dependence plot (background moderator variables of Triglycerides in BC subgroups)


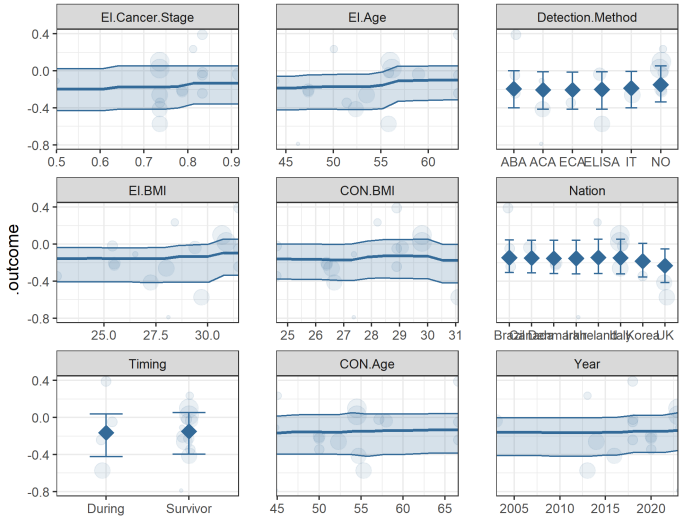


Fig. S79. Partial dependence plot (background moderator variables of Total cholesterol in BC subgroups)


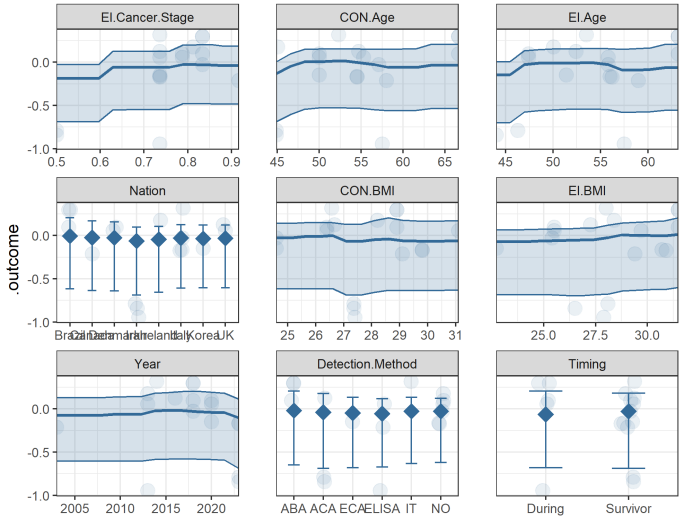


Fig. S80. Partial dependence plot (background moderator variables of HDL-C in BC subgroups)


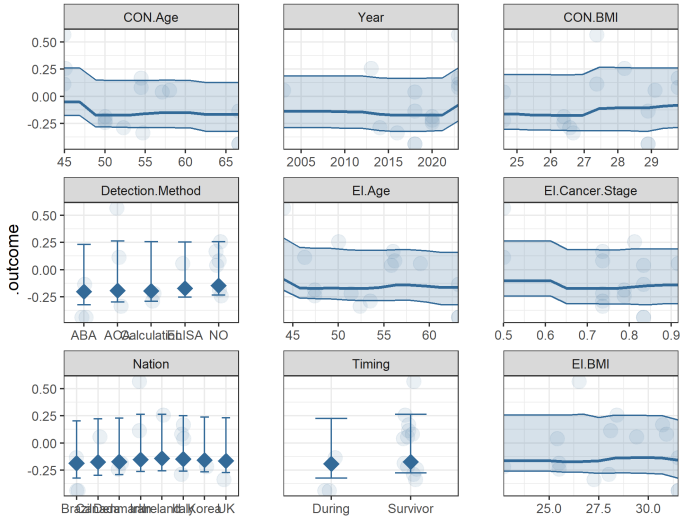


Fig. S81. Partial dependence plot (background moderator variables of LDL-C in BC subgroups)


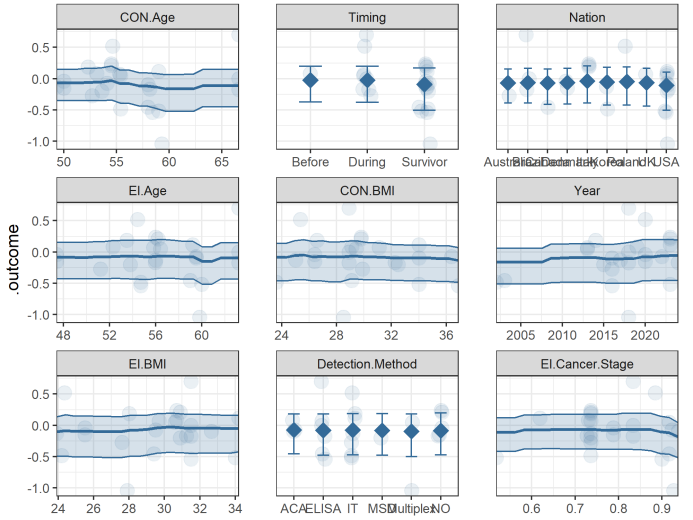


Fig. S82. Partial dependence plot (background moderator variables of CRP in BC subgroups)


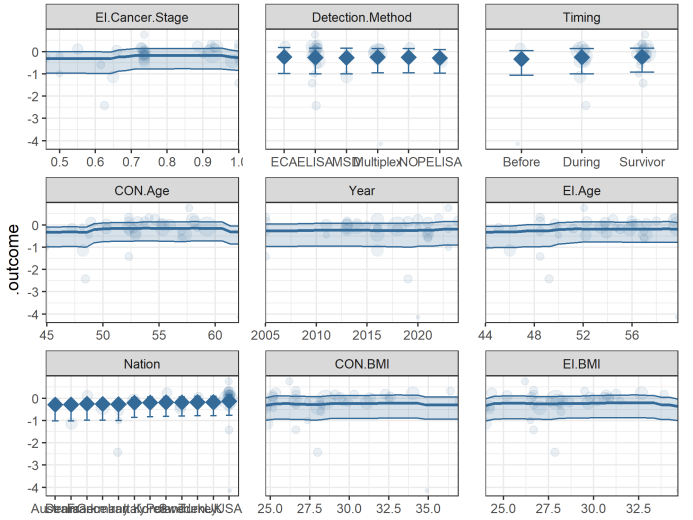


Fig. S83. Partial dependence plot (background moderator variables of IL-6 in BC subgroups)


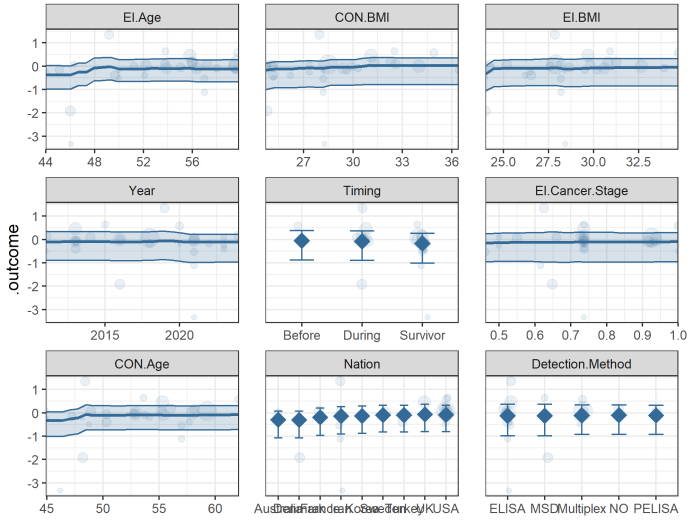


Fig. S84. Partial dependence plot (background moderator variables of TNF-α in BC subgroups)


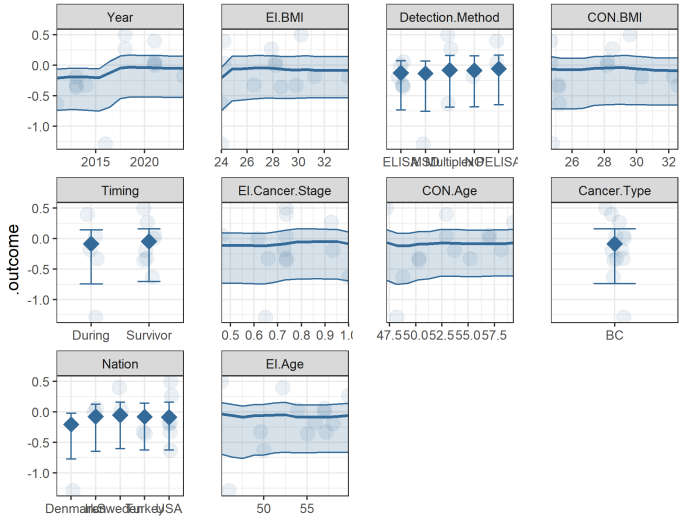


Fig. S85. Partial dependence plot (background moderator variables of IL-8 in BC subgroups)


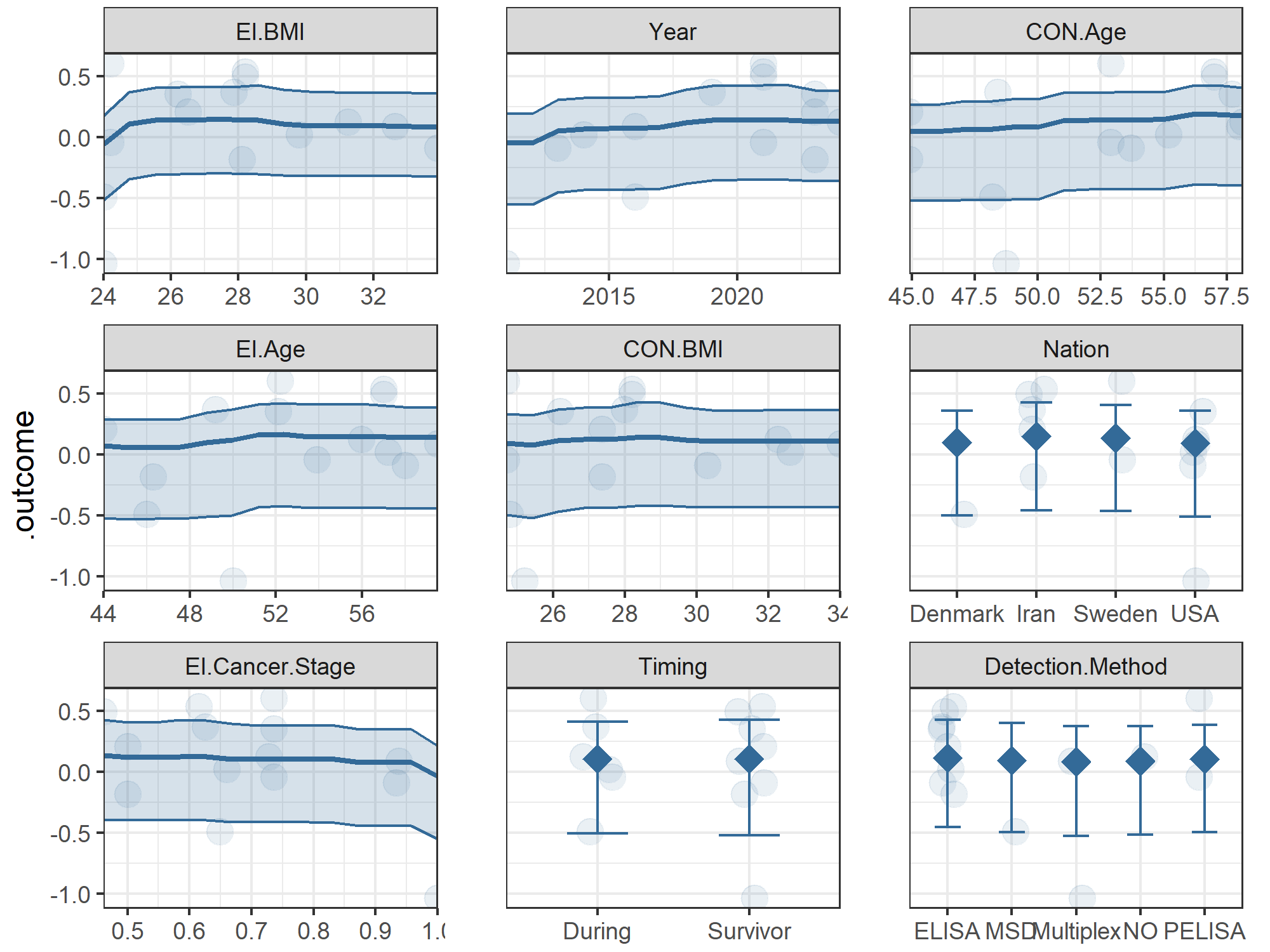


Fig. S86. Partial dependence plot (background moderator variables of IL-10 in BC subgroups)

**Supplementary information 11. Results of MetaForest regression models for subgroups by intervention timing.**


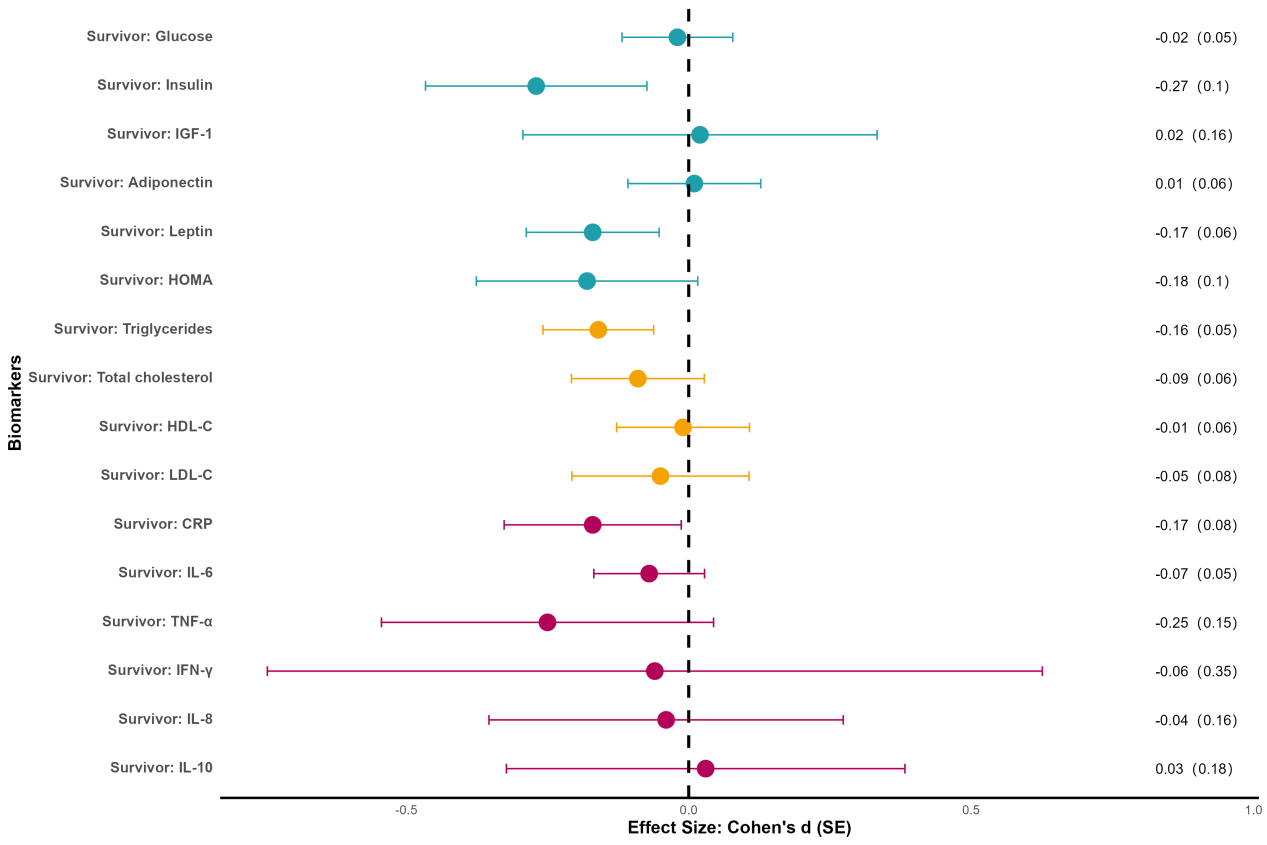


Fig. S87.Forest plot of subgroups by intervention timing

Table S12. RVE model parameters without moderators for subgroups by intervention timing

|  | ES | SE | t | dfs | prob | CI.L | CI.U | sig | I² |
| --- | --- | --- | --- | --- | --- | --- | --- | --- | --- |
| Survivor: Glucose | -0.02 | 0.05 | -0.49 | 11.04 | 0.64 | -0.14 | 0.09 |  | 0.00 |
| Survivor: Insulin | -0.27 | 0.10 | -2.77 | 21.75 | 0.01 | -0.47 | -0.07 | ** | 0.48 |
| Survivor: IGF-1 | 0.02 | 0.16 | 0.13 | 10.49 | 0.90 | -0.33 | 0.37 |  | 0.63 |
| Survivor: Adiponectin | 0.01 | 0.06 | 0.15 | 9.86 | 0.88 | -0.13 | 0.15 |  | 0.00 |
| Survivor: Leptin | -0.17 | 0.06 | -2.99 | 10.85 | 0.01 | -0.30 | -0.04 | ** | 0.11 |
| Survivor: HOMA index | -0.18 | 0.10 | -1.82 | 11.19 | 0.10 | -0.41 | 0.04 | * | 0.32 |
| Survivor: Triglycerides | -0.16 | 0.05 | -2.98 | 8.15 | 0.02 | -0.28 | -0.04 | ** | 0.00 |
| Survivor: Total cholesterol | -0.09 | 0.06 | -1.60 | 8.66 | 0.15 | -0.22 | 0.04 |  | 0.00 |
| Survivor: HDL-C | -0.01 | 0.06 | -0.14 | 8.65 | 0.89 | -0.14 | 0.13 |  | 0.00 |
| Survivor: LDL-C | -0.05 | 0.08 | -0.69 | 7.16 | 0.51 | -0.24 | 0.13 |  | 0.00 |
| Survivor: CRP | -0.17 | 0.08 | -1.99 | 17.12 | 0.06 | -0.35 | 0.01 | * | 0.51 |
| Survivor: IL-6 | -0.07 | 0.05 | -1.38 | 11.12 | 0.19 | -0.19 | 0.04 |  | 0.02 |
| Survivor: TNF-α | -0.25 | 0.15 | -1.61 | 16.13 | 0.13 | -0.57 | 0.08 |  | 0.61 |
| Survivor: IFN-γ | -0.06 | 0.35 | -0.18 | 1.98 | 0.87 | -1.60 | 1.48 |  | 0.55 |
| Survivor: IL-8 | -0.04 | 0.16 | -0.25 | 5.01 | 0.80 | -0.45 | 0.37 |  | 0.35 |
| Survivor: IL-10 | 0.03 | 0.18 | 0.15 | 4.82 | 0.89 | -0.44 | 0.49 |  | 0.25 |

Table S15. Parameters of MetaForest regression models with exercise prescription moderator variables in survivor subgroups

| ID | R²_OOB_ | τ² | Feature Weights | Mtry | min.node.size | R²_CV_ | R²_CV_ SD | RMSE | RMSE SD | MAE | MAE SD |
| --- | --- | --- | --- | --- | --- | --- | --- | --- | --- | --- | --- |
| Glucose | -0.10 | 0.00 | fixed | 2.00 | 4.00 | 0.10 | 0.14 | 0.27 | 0.09 | 0.21 | 0.06 |
| Insulin | -0.24 | 0.18 | fixed | 2.00 | 4.00 | 0.07 | 0.09 | 0.51 | 0.14 | 0.38 | 0.09 |
| IGF-1 | -0.16 | 0.27 | unif | 2.00 | 2.00 | 0.22 | 0.25 | 0.56 | 0.15 | 0.45 | 0.13 |
| Adiponectin | 0.04 | 0.02 | unif | 2.00 | 4.00 | 0.18 | 0.16 | 0.26 | 0.06 | 0.21 | 0.05 |
| Leptin | -0.12 | 0.05 | fixed | 2.00 | 4.00 | 0.11 | 0.15 | 0.41 | 0.13 | 0.32 | 0.08 |
| HOMA index | -0.12 | 0.04 | fixed | 2.00 | 4.00 | 0.13 | 0.17 | 0.36 | 0.10 | 0.27 | 0.08 |
| Triglycerides | -0.15 | 0.00 | fixed | 2.00 | 4.00 | 0.12 | 0.18 | 0.24 | 0.05 | 0.20 | 0.05 |
| Total cholesterol | -0.12 | 0.00 | fixed | 2.00 | 4.00 | 0.14 | 0.18 | 0.27 | 0.06 | 0.21 | 0.05 |
| HDL-C | -0.18 | 0.00 | fixed | 2.00 | 4.00 | 0.12 | 0.15 | 0.34 | 0.09 | 0.27 | 0.07 |
| LDL-C | -0.09 | 0.00 | unif | 2.00 | 4.00 | 0.19 | 0.21 | 0.25 | 0.06 | 0.20 | 0.06 |
| CRP | -0.55 | 0.14 | unif | 2.00 | 4.00 | 0.16 | 0.17 | 0.36 | 0.11 | 0.26 | 0.08 |
| IL-6 | -0.08 | 0.04 | fixed | 2.00 | 4.00 | 0.11 | 0.15 | 0.49 | 0.12 | 0.35 | 0.09 |
| TNF-α | -0.14 | 0.43 | fixed | 2.00 | 4.00 | 0.19 | 0.18 | 0.70 | 0.38 | 0.45 | 0.19 |

Note: R^2^_OOB_: Out-of-Bag R-Squared. τ²: Between-Studies Variance. R^2^_CV_: Cross-Validation R-Squared. R^2^_CV_ SD: Cross-Validation R-Squared Standard Deviation. Mtry: Number of Variables Randomly Sampled as Candidates at Each Split. min.node.size: Minimum Node Size. RMSE: Root Mean Square Error. MASE: Mean Absolute Scaled Error.


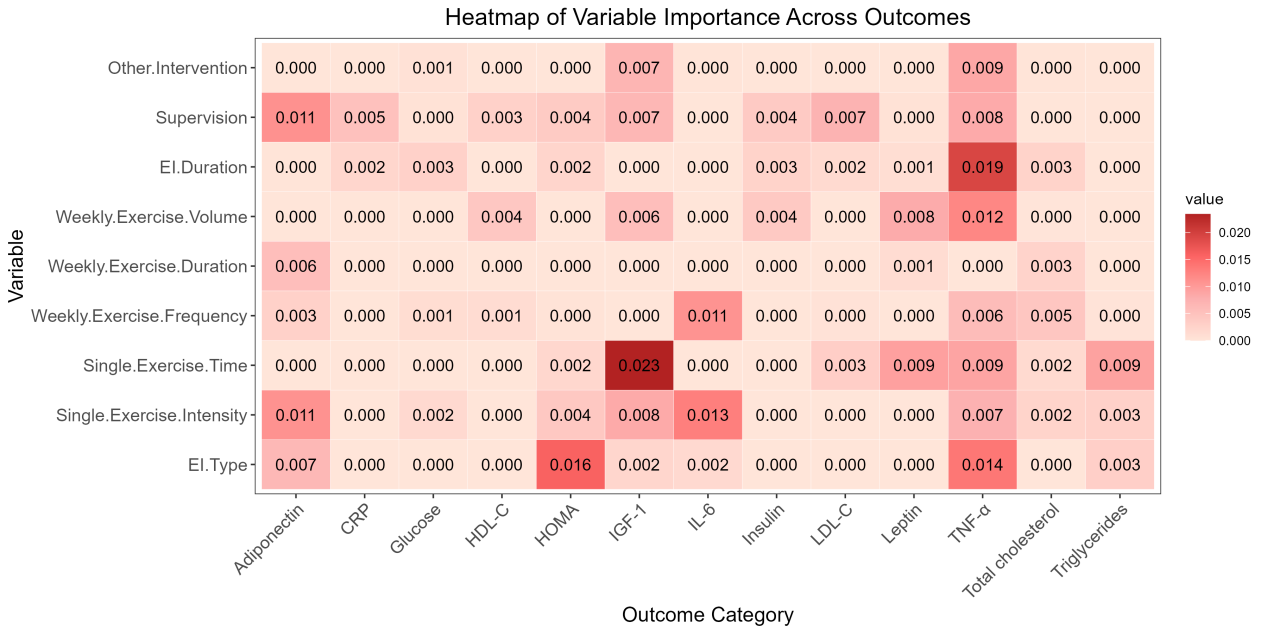


Fig. S88. Heat map of the importance of exercise prescription moderator variables in Survivor subgroups


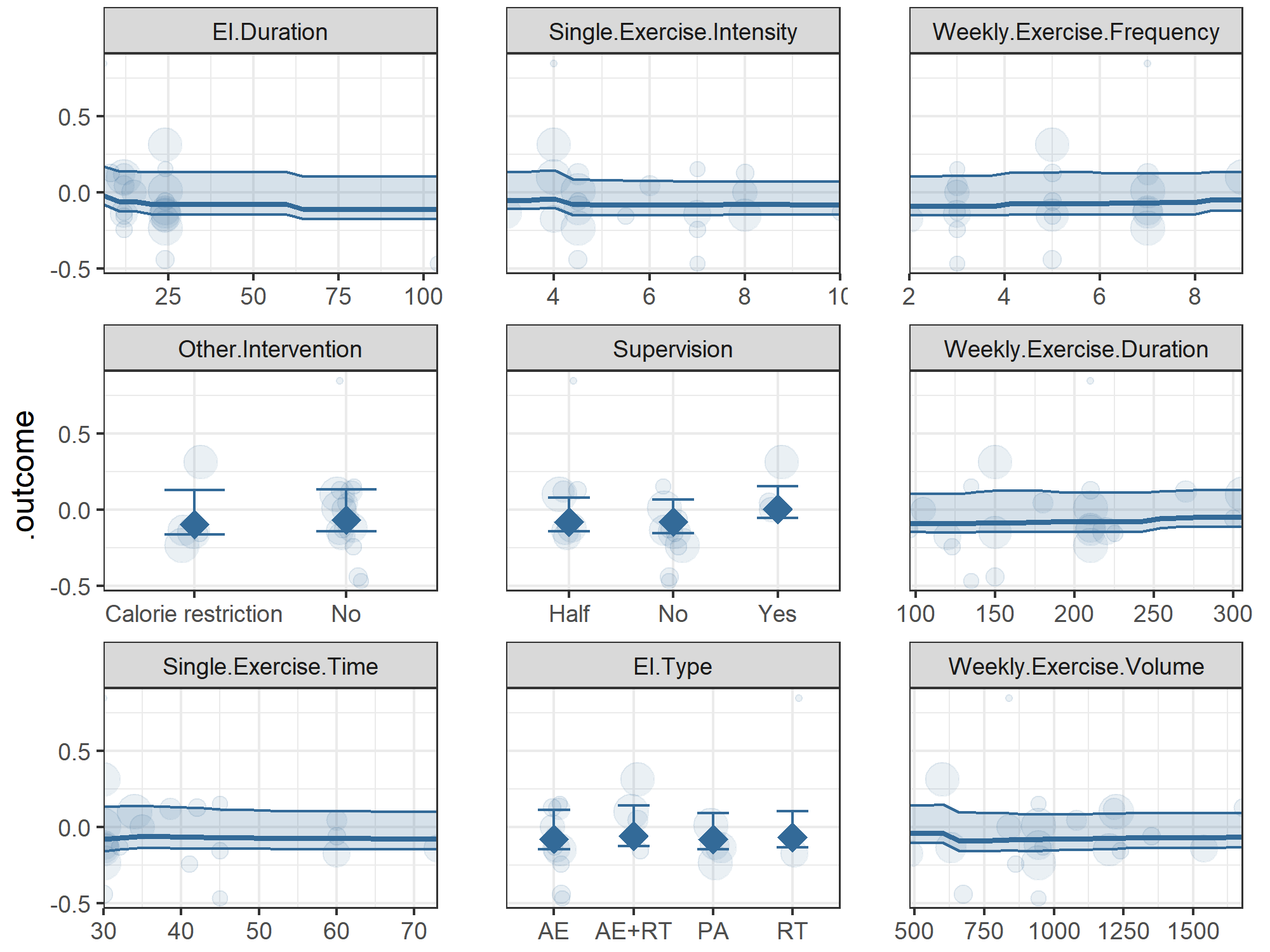


Fig. S89. Partial dependence plot (exercise prescription moderator variables of Glucose in Survivor subgroups)


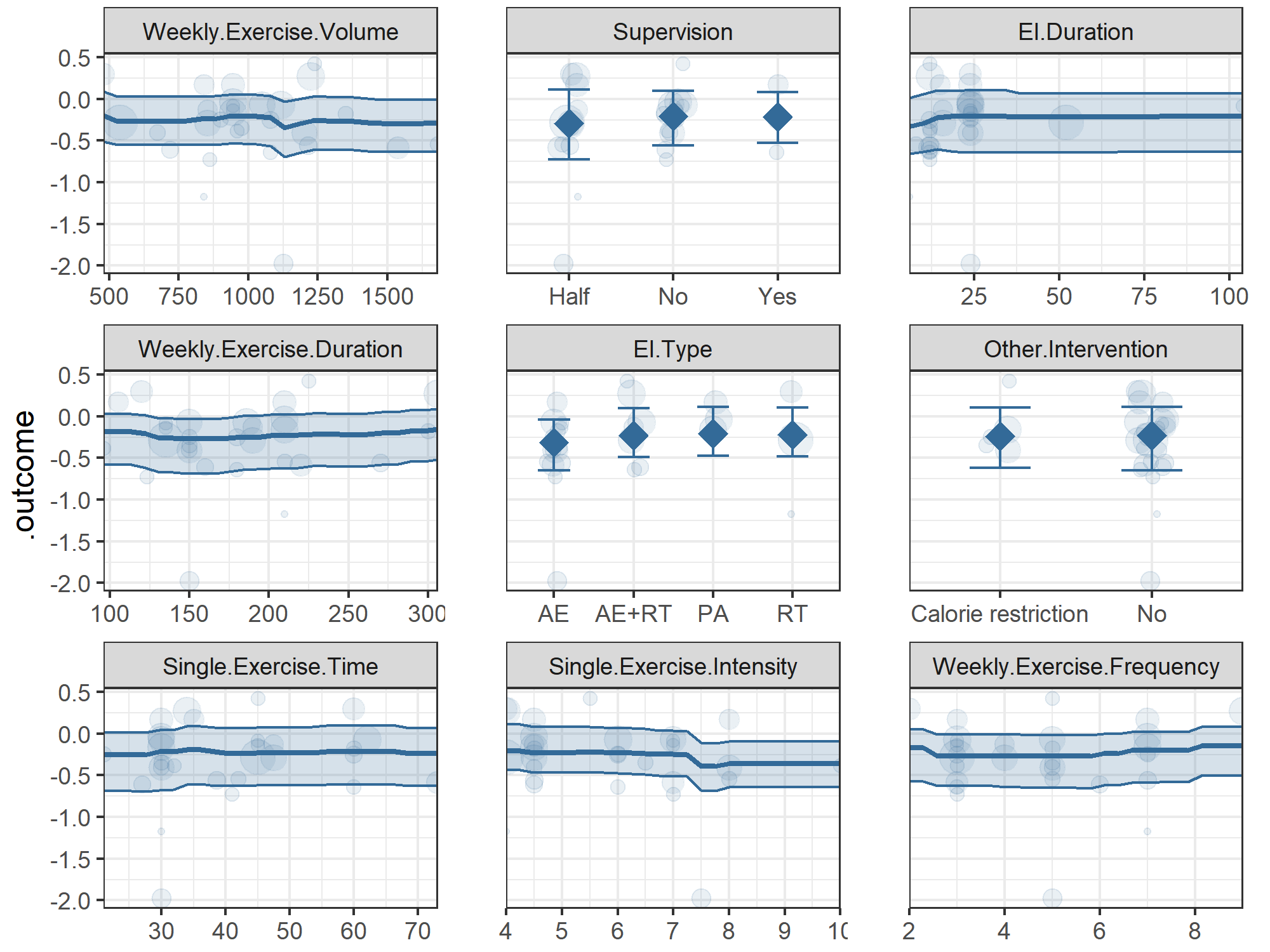


Fig. S90. Partial dependence plot (exercise prescription moderator variables of Insulin in Survivor subgroups)


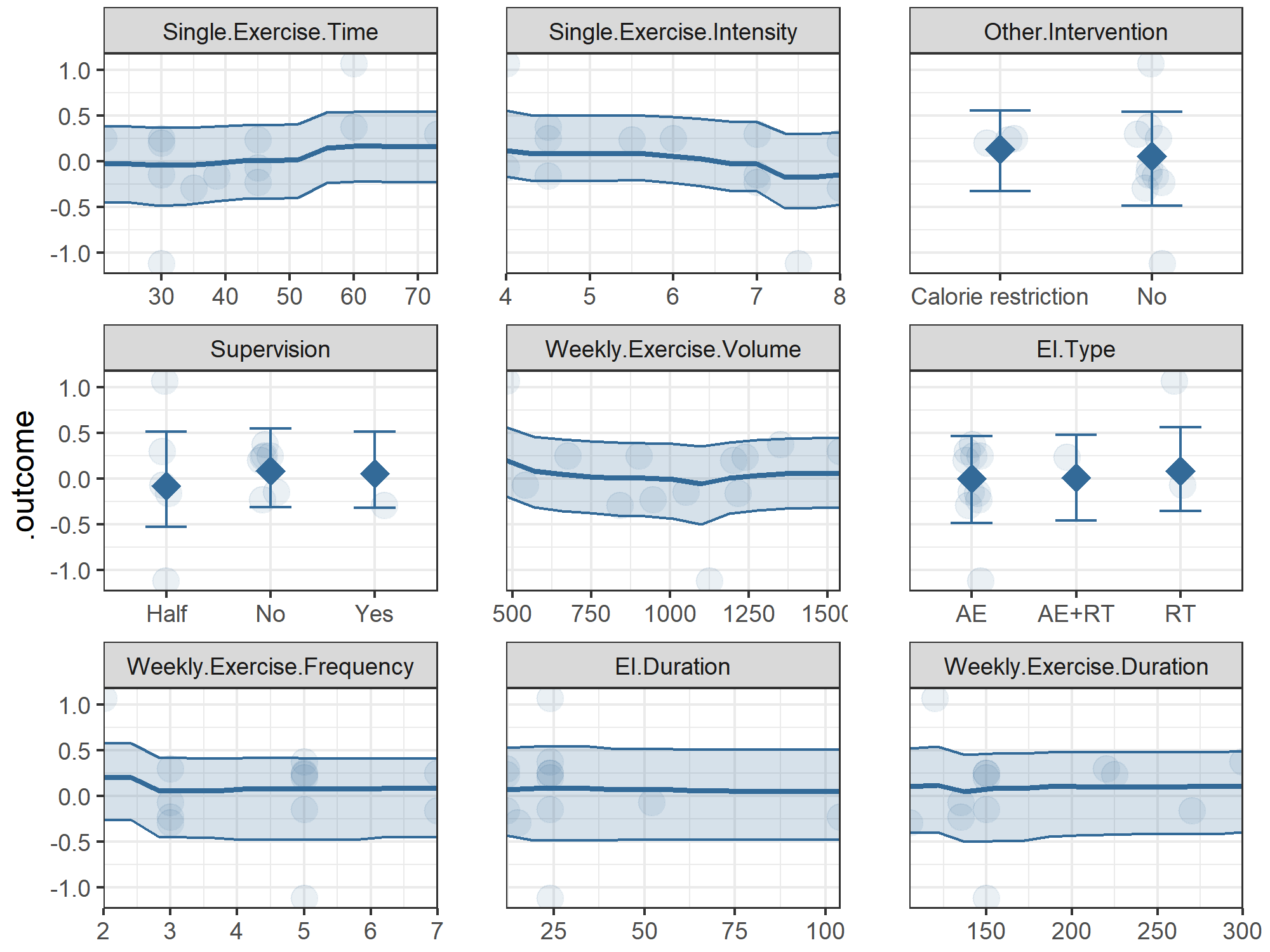


Fig. S91. Partial dependence plot (exercise prescription moderator variables of IGF-1 in Survivor subgroups)


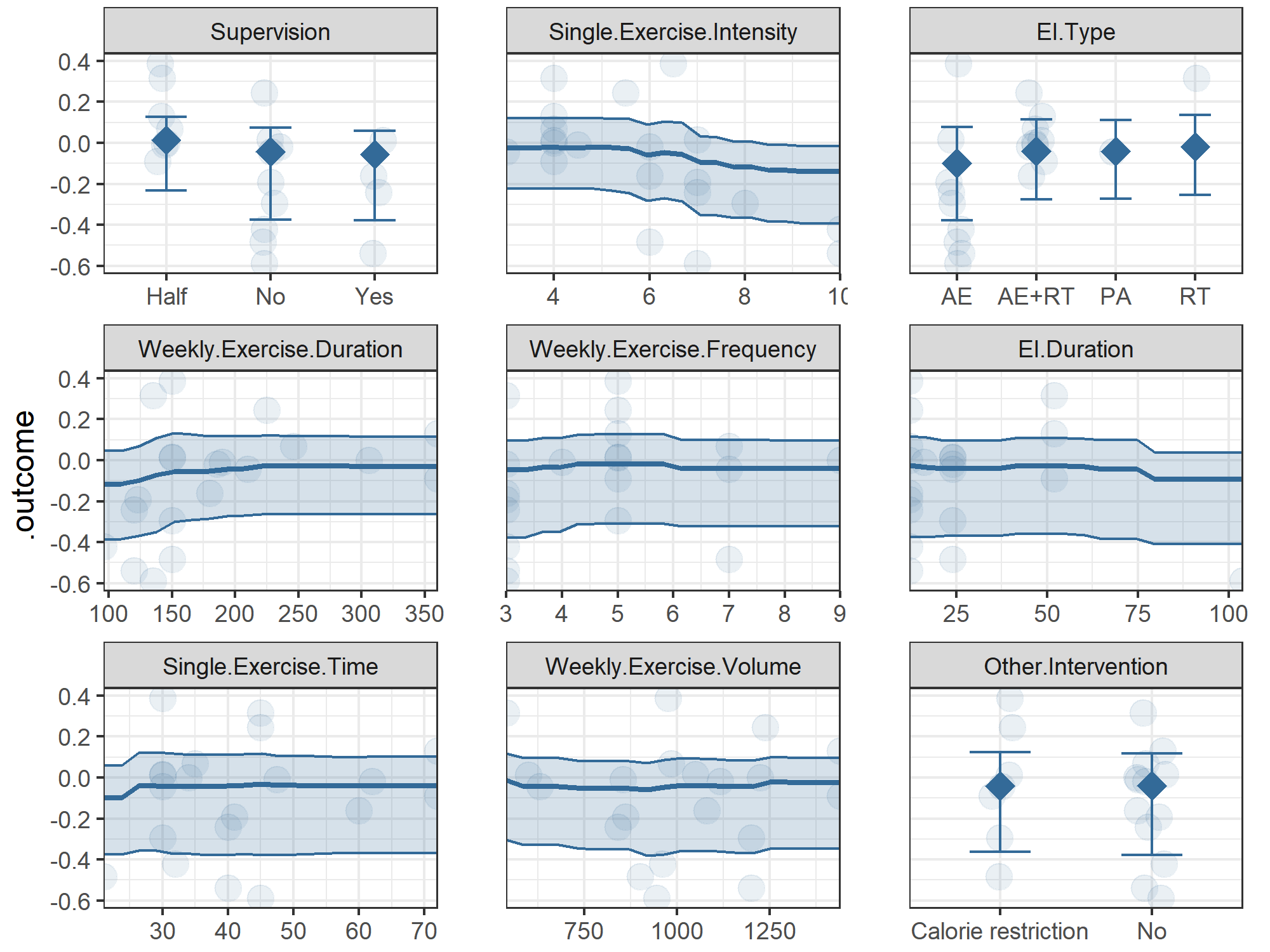


Fig. S92. Partial dependence plot (exercise prescription moderator variables of Adiponectin in Survivor subgroups)


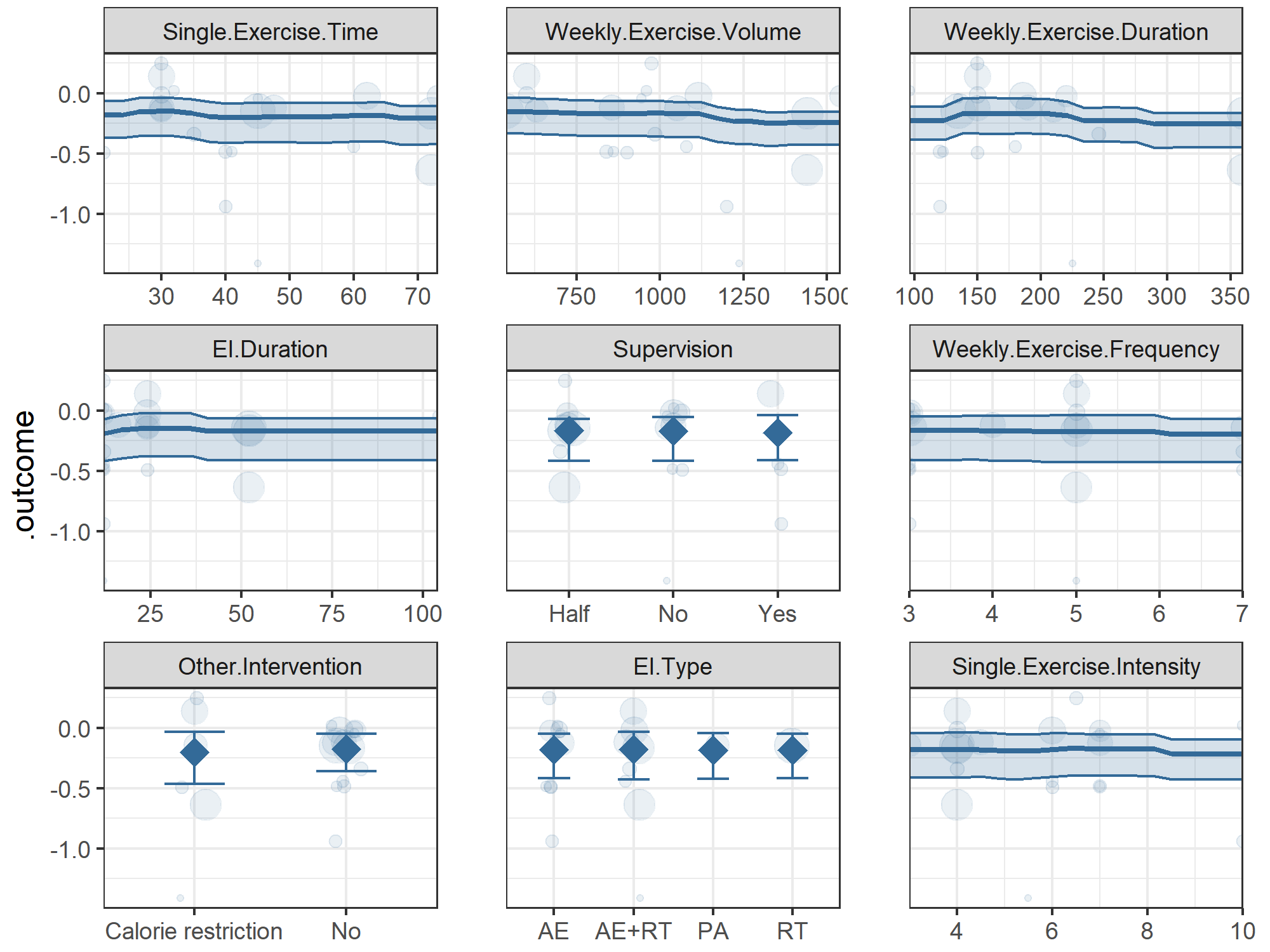


Fig. S93. Partial dependence plot (exercise prescription moderator variables of Leptin in Survivor subgroups)


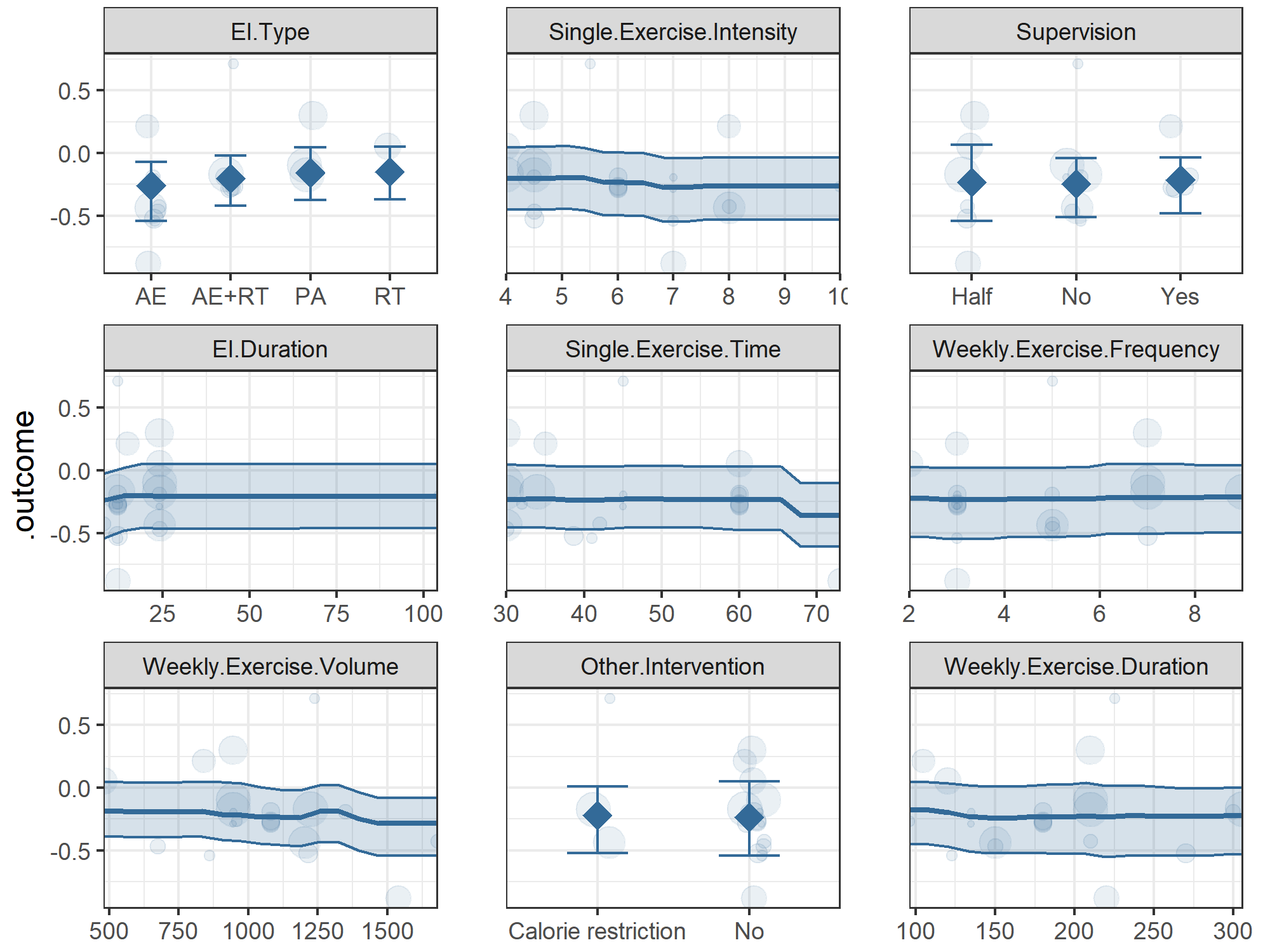


Fig. S94. Partial dependence plot (exercise prescription moderator variables of HOMA index in Survivor subgroups)


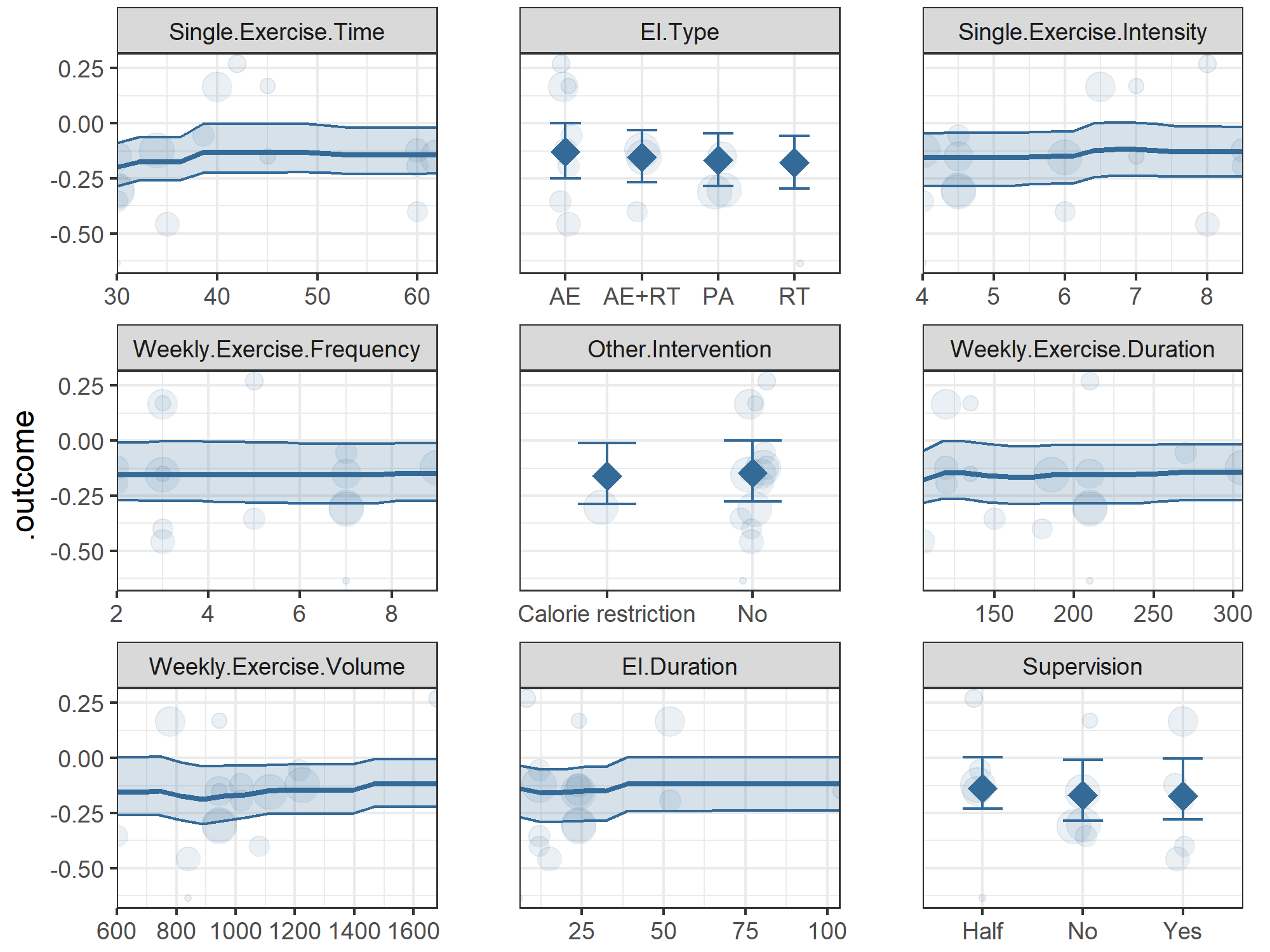


Fig. S95. Partial dependence plot (exercise prescription moderator variables of Triglycerides in Survivor subgroups)


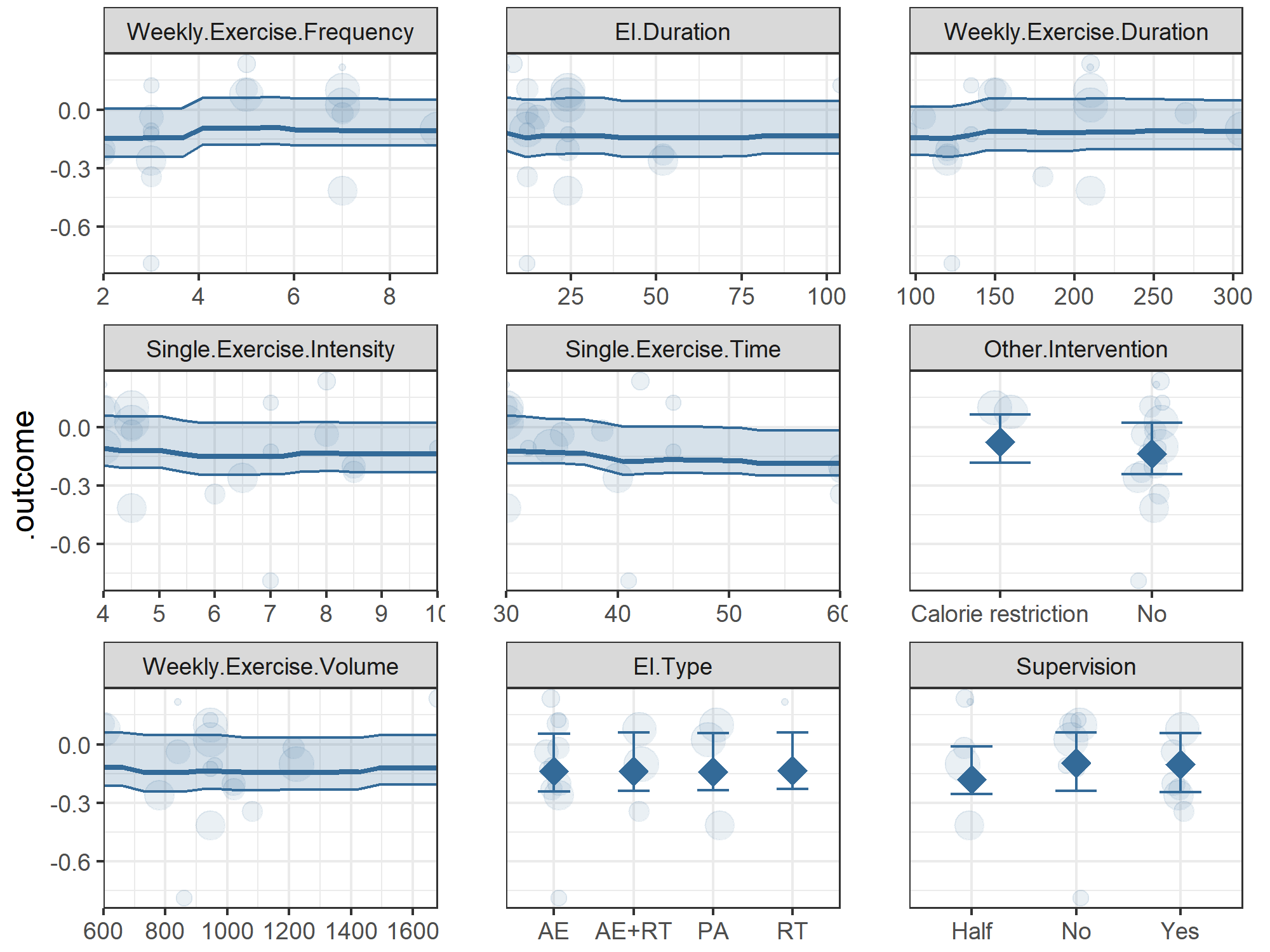


Fig. S96. Partial dependence plot (exercise prescription moderator variables of Total cholesterol in Survivor subgroups)


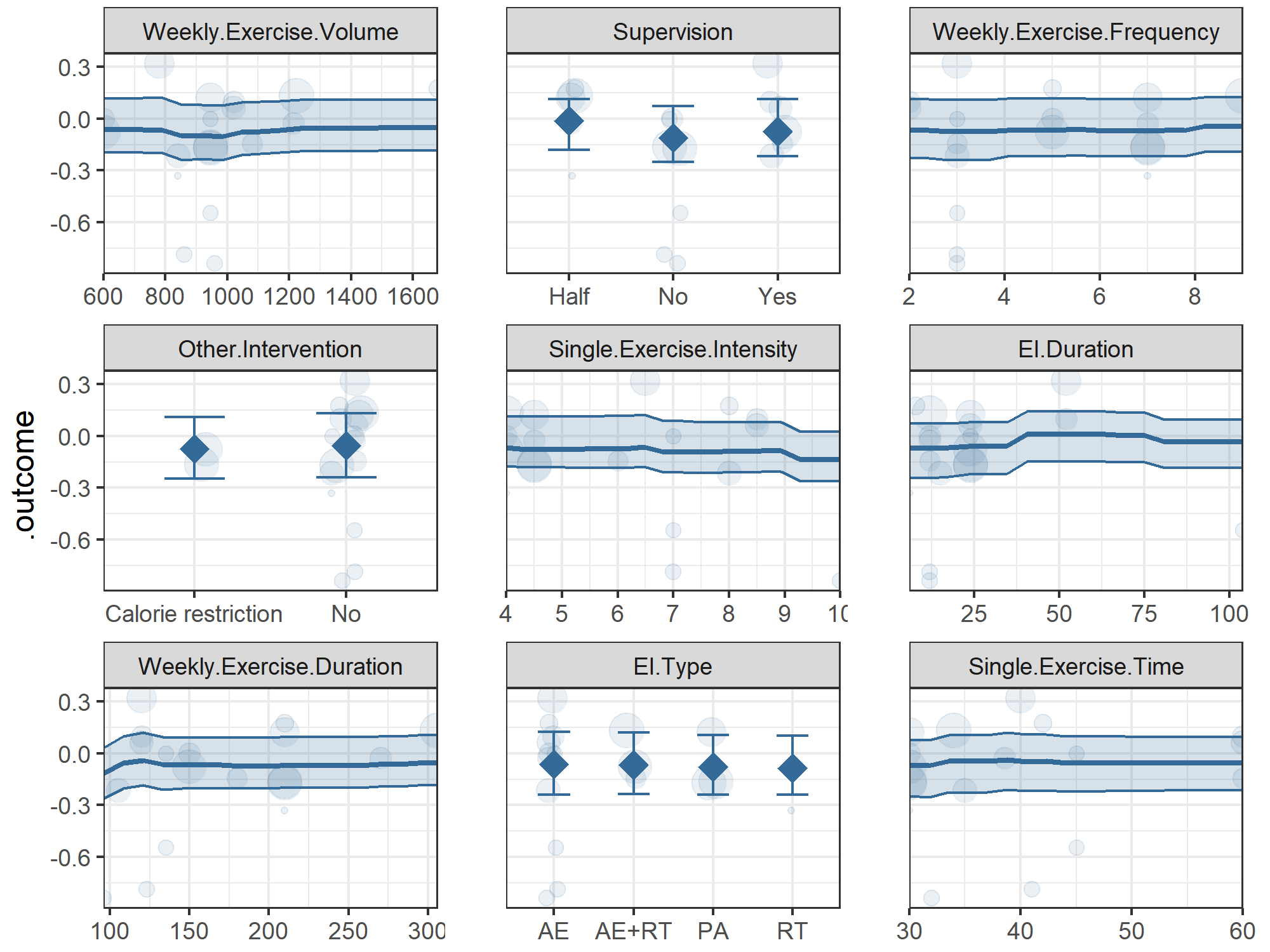


Fig. S97. Partial dependence plot (exercise prescription moderator variables of HDL-C in Survivor subgroups)


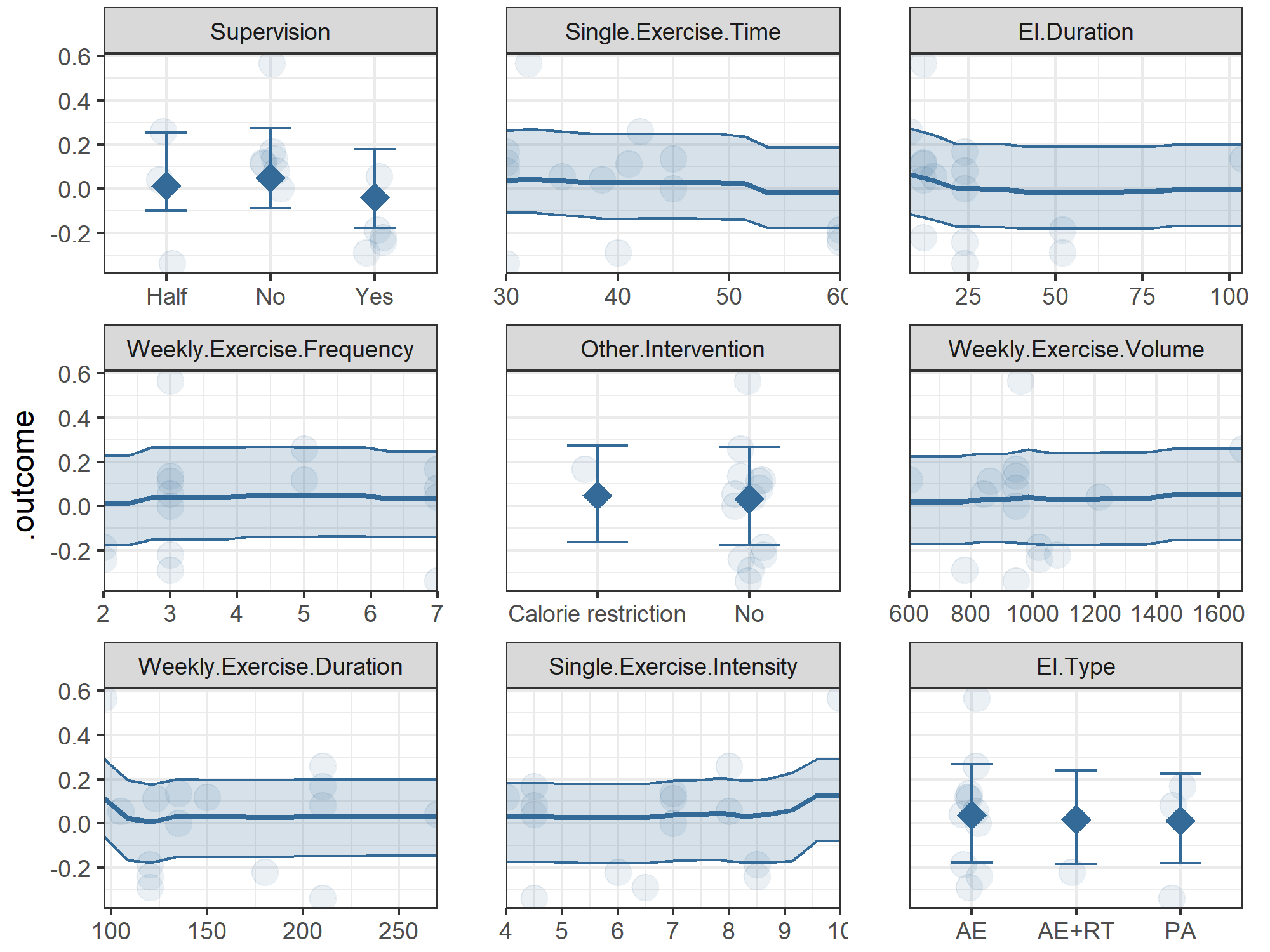


Fig. S98. Partial dependence plot (exercise prescription moderator variables of LDL-C in Survivor subgroups)


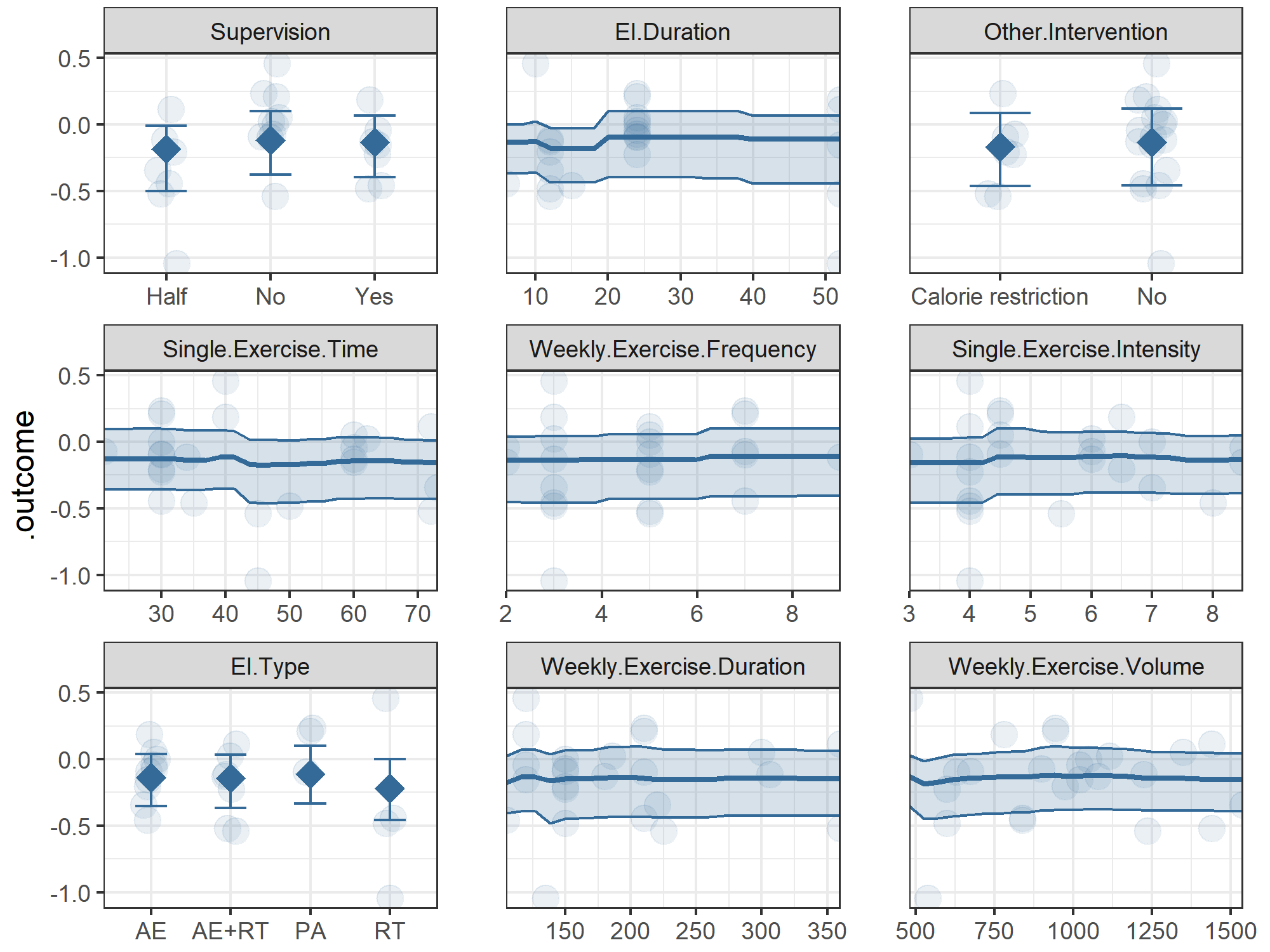


Fig. S99. Partial dependence plot (exercise prescription moderator variables of CRP in Survivor subgroups)

Fig. S100. Partial dependence plot (exercise prescription moderator variables of IL-6 in Survivor subgroups)

Fig. S101. Partial dependence plot (exercise prescription moderator variables of TNF-α in Survivor subgroups)

Table S13. Parameters of MetaForest regression models with background moderating variables in survivor subgroups

| ID | R²_OOB_ | τ² | Feature Weights | Mtry | min.node.size | R²_CV_ | R²_CV_ SD | RMSE | RMSE SD | MAE | MAE SD |
| --- | --- | --- | --- | --- | --- | --- | --- | --- | --- | --- | --- |
| Glucose | -0.26 | 0.01 | random | 2.00 | 4.00 | 0.17 | 0.17 | 0.32 | 0.09 | 0.24 | 0.06 |
| Insulin | -0.32 | 0.19 | fixed | 2.00 | 4.00 | 0.10 | 0.11 | 0.53 | 0.13 | 0.39 | 0.09 |
| Adiponectin | -0.10 | 0.03 | unif | 2.00 | 4.00 | 0.15 | 0.15 | 0.28 | 0.06 | 0.23 | 0.06 |
| Leptin | -0.10 | 0.04 | fixed | 2.00 | 4.00 | 0.10 | 0.11 | 0.41 | 0.12 | 0.32 | 0.08 |
| HOMA index | 0.12 | 0.02 | unif | 2.00 | 2.00 | 0.21 | 0.22 | 0.33 | 0.10 | 0.25 | 0.08 |
| Triglycerides | 0.03 | 0.00 | random | 2.00 | 2.00 | 0.18 | 0.19 | 0.23 | 0.05 | 0.18 | 0.05 |
| Total cholesterol | 0.16 | 0.00 | random | 2.00 | 4.00 | 0.27 | 0.22 | 0.23 | 0.07 | 0.18 | 0.05 |
| HDL-C | 0.03 | 0.00 | unif | 2.00 | 2.00 | 0.33 | 0.30 | 0.30 | 0.10 | 0.25 | 0.08 |
| LDL-C | 0.13 | 0.00 | unif | 2.00 | 4.00 | 0.37 | 0.29 | 0.22 | 0.09 | 0.17 | 0.06 |
| CRP | -0.31 | 0.10 | unif | 2.00 | 4.00 | 0.11 | 0.13 | 0.34 | 0.08 | 0.28 | 0.06 |
| IL-6 | -0.04 | 0.00 | fixed | 2.00 | 4.00 | 0.08 | 0.10 | 0.48 | 0.12 | 0.34 | 0.08 |
| TNF-α | -0.13 | 0.41 | fixed | 2.00 | 4.00 | 0.20 | 0.16 | 0.71 | 0.37 | 0.48 | 0.18 |

Fig. S102. Heat map of the importance of background moderator variables in Survivor subgroups

Fig. S103. Partial dependence plot (background moderator variables of Glucose in Survivor subgroups)

Fig. S104. Partial dependence plot (background moderator variables of Insulin in Survivor subgroups)

Fig. S105. Partial dependence plot (background moderator variables of Adiponectin in Survivor subgroups)

Fig. S106. Partial dependence plot (background moderator variables of Leptin in Survivor subgroups)

Fig. S107. Partial dependence plot (background moderator variables of HOMA index in Survivor subgroups)

Fig. S108. Partial dependence plot (background moderator variables of Triglycerides in Survivor subgroups)

Fig. S109. Partial dependence plot (background moderator variables of Total cholesterol in Survivor subgroups)

Fig. S110. Partial dependence plot (background moderator variables of HDL-C in Survivor subgroups)

Fig. S111. Partial dependence plot (background moderator variables of LDL-C in Survivor subgroups)

Fig. S112. Partial dependence plot (background moderator variables of CRP in Survivor subgroups)

Fig. S113. Partial dependence plot (background moderator variables of IL-6 in Survivor subgroups)

Fig. S114. Partial dependence plot (background moderator variables of TNF-α in Survivor subgroups)

**Supplementary information 12. Results of the MetaForest regression models for subgroups by cancer type and intervention timing.**

Fig. S115. Forest plot of subgroups by cancer type and intervention timing

Table S14. RVE model parameters without moderators for subgroups by cancer type and intervention timing

|  | ES | SE | t | dfs | prob | CI.L | CI.U | sig | I² |
| --- | --- | --- | --- | --- | --- | --- | --- | --- | --- |
| BC During |  |  |  |  |  |  |  |  |  |
| Glucose–Insulin Group | -0.12 | 0.13 | -0.89 | 5.75 | 0.40 | -0.45 | 0.21 |  | 0.51 |
| Lipid Group | -0.43 | 0.29 | -1.48 | 1.96 | 0.27 | -1.72 | 0.84 |  | 0.70 |
| Inflammatory Group | -0.14 | 0.12 | -1.14 | 10.95 | 0.27 | -0.42 | 0.13 |  | 0.79 |
| BC Survivor |  |  |  |  |  |  |  |  |  |
| Glucose–Insulin Group | -0.21 | 0.08 | -2.46 | 18.52 | 0.02 | -0.38 | -0.03 | ** | 0.50 |
| Lipid Group | -0.10 | 0.03 | -2.87 | 5.89 | 0.0 | -0.18 | -0.01 | ** | 0 |
| Inflammatory Group | -0.20 | 0.11 | -1.78 | 22.02 | 0.08 | -0.42 | 0.03 | * | 0.61 |

Table S15. Parameters of MetaForest regression models for inflammatory and obesity markers in BC During and BC Survivor subgroups

| ID | R²_OOB_ | τ² | Feature Weights | Mtry | min.node.size | R²_CV_ | R²_CV_ SD | RMSE | RMSE SD | MAE | MAE SD |
| --- | --- | --- | --- | --- | --- | --- | --- | --- | --- | --- | --- |
| BC During |  |  |  |  |  |  |  |  |  |  |  |
| Glucose–Insulin Group | 0.58 | 0.01 | Unif | 2 | 4 | 0.58 | 0.24 | 0.31 | 0.12 | 0.25 | 0.08 |
| Lipid Group | 0.25 | 0.08 | Unif | 2 | 2 | 0.44 | 0.29 | 0.40 | 0.13 | 0.33 | 0.11 |
| Inflammatory Group | -0.06 | 0.36 | Fixed | 2 | 4 | 0.18 | 0.18 | 0.68 | 0.20 | 0.44 | 0.10 |
| BC Survivor |  |  |  |  |  |  |  |  |  |  |  |
| Glucose–Insulin Group | 0.13 | 0.04 | Fixed | 2 | 2 | 0.13 | 0.13 | 0.41 | 0.07 | 0.29 | 0.04 |
| Lipid Group | -0.12 | 0.00 | Random | 2 | 4 | 0.06 | 0.07 | 0.30 | 0.05 | 0.23 | 0.03 |
| Inflammatory Group | -0.20 | 0.19 | Fixed | 2 | 4 | 0.02 | 0.03 | 0.59 | 0.14 | 0.40 | 0.06 |

Fig. S116. Heat map of the importance of exercise prescription moderator variables in BC During and BC Survivor subgroups

Fig. S117. Partial dependence plot (Effect of exercise prescription moderator variables on the overall effect size of Glucose–Insulin Group in BC During subgroups)

Fig. S118. Partial dependence plot (Effect of exercise prescription moderator variables on the overall effect size of Lipid Group in BC During subgroups)

Fig. S119. Partial dependence plot (Effect of exercise prescription moderator variables on the overall effect size of Inflammatory Group in BC During subgroups)

Fig. S120. Partial dependence plot (Effect of exercise prescription moderator variables on the overall effect size of Glucose–Insulin Group in BC Survivor subgroups)

Fig. S121. Partial dependence plot (Effect of exercise prescription moderator variables on the overall effect size of Lipid Group in BC Survivor subgroups)

Fig. S122. Partial dependence plot (Effect of exercise prescription moderator variables on the overall effect size of Inflammatory Group in BC Survivor subgroups)

**Supplementary information 13. Grading of Recommendations, Assessment, Development and Evaluations (GRADE).**

Table S16. GRADE rating for effect size

| Outcome | Effect Size | SE | Risk of Bias | Imprecision(p) | Indirectness | Inconsistency (I²) | Publication Bias | GRADE Quality |
| --- | --- | --- | --- | --- | --- | --- | --- | --- |
| Glucose | -0.07 | 0.07 | ROB2*:  7/74 High Risk;  24/74 Unclear Risk | 0.29† | Direct | 0.35† | Egger’s p > 0.05 | Very Low*‡ |
| Insulin | -0.24 | 0.08 |  | 0.00 | Direct | 0.49† |  | Low*† |
| IGF-1 | -0.04 | 0.12 |  | 0.75† | Direct | 0.51† |  | Very Low*‡ |
| Adiponectin | 0.01 | 0.05 |  | 0.88† | Direct | 0.00 |  | Low*† |
| Leptin | -0.16 | 0.06 |  | 0.02 | Direct | 0.27† |  | Low*† |
| HOMA | -0.19 | 0.09 |  | 0.06† | Direct | 0.29† |  | Very Low*‡ |
| Triglycerides | -0.16 | 0.06 |  | 0.02 | Direct | 0.20 |  | Moderate* |
| Total cholesterol | -0.11 | 0.05 |  | 0.05 | Direct | 0.00 |  | Moderate* |
| HDL-C | -0.03 | 0.05 |  | 0.49† | Direct | 0.00 |  | Low*† |
| LDL-C | -0.05 | 0.05 |  | 0.38† | Direct | 0.00 |  | Low*† |
| CRP | -0.19 | 0.09 |  | 0.04 | Direct | 0.63† |  | Low*† |
| IL-6 | -0.17 | 0.10 |  | 0.08† | Direct | 0.69† |  | Very Low*‡ |
| TNF-α | -0.22 | 0.13 |  | 0.10† | Direct | 0.74† |  | Very Low*‡ |
| IFN-γ | -0.08 | 0.26 |  | 0.76† | Direct | 0.85† |  | Very Low*‡ |
| IL-8 | -0.16 | 0.15 |  | 0.33† | Direct | 0.63† |  | Very Low*‡ |
| IL-10 | 0.03 | 0.11 |  | 0.81† | Direct | 0.36† |  | Very Low*‡ |

Note: Reasons for downgrading: †Imprecision (1 downgrade), ‡Severe imprecision (2 downgrades), *risk of bias (1 downgrade) , #inconsistency (1 downgrade).

Table S17. GRADE rating for Metaforest

| Outcome | Risk of Bias | Imprecision(p) | Inconsistency | Indirectness | Publication Bias | GRADE Quality |
| --- | --- | --- | --- | --- | --- | --- |
| Glucose | ROB2*:  7/74 High Risk;  24/74 Unclear Risk | NO | Direct |  | Egger’s p > 0.05 | Low* |
| Insulin |  | NO | Direct |  |  | Low* |
| IGF-1 |  | NO | Direct |  |  | Low* |
| Adiponectin |  | NO | Direct |  |  | Low* |
| Leptin |  | NO | Direct |  |  | Low* |
| HOMA |  | NO | Direct |  |  | Low* |
| Triglycerides |  | NO | Direct |  |  | Low* |
| Total cholesterol |  | NO | Direct |  |  | Low* |
| HDL-C |  | NO | Direct |  |  | Low* |
| LDL-C |  | NO | Direct |  |  | Low* |
| CRP |  | NO | Direct |  |  | Low* |
| IL-6 |  | NO | Direct |  |  | Low* |
| TNF-α |  | NO | Direct |  |  | Low* |
| IFN-γ |  | NO | Direct |  |  | Low* |
| IL-8 |  | NO | Direct |  |  | Low* |
| IL-10 |  | NO | Direct |  |  | Low* |

Note: The initial rating was assigned as “Moderate.” Inconsistency was assessed based on model performance differences across the three categories of moderator variables (RMSE, MAE, RMSE SD, and MAE SD). Imprecision was evaluated based on variation in model performance across the three moderator categories (R²CV and R²CV SD). †Imprecision (1 downgrade), ‡Severe imprecision (2 downgrades), *risk of bias (1 downgrade) , #inconsistency (1 downgrade).
